# Exploration of a Polygenic Risk Score for Alcohol Consumption: A Longitudinal Analysis from the ALSPAC Cohort

**DOI:** 10.1371/journal.pone.0167360

**Published:** 2016-11-30

**Authors:** Michelle Taylor, Andrew J. Simpkin, Philip C. Haycock, Frank Dudbridge, Luisa Zuccolo

**Affiliations:** 1 MRC Integrative Epidemiology Unit at the University of Bristol (IEU), University of Bristol, Bristol, United Kingdom; 2 School of Social and Community Medicine, Faculty of Medicine and Dentistry, University of Bristol, Bristol, United Kingdom; 3 UK Centre for Tobacco and Alcohol Studies, School of Experimental Psychology, University of Bristol, Bristol, United Kingdom; 4 Department of Non-communicable Disease Epidemiology, London School of Hygiene and Tropical Medicine, London, United Kingdom; Hunter College, UNITED STATES

## Abstract

**Background:**

Uncertainty remains about the true extent by which alcohol consumption causes a number of health outcomes. Genetic variants, or combinations of variants built into a polygenic risk score (PGRS), can be used in an instrumental variable framework to assess causality between a phenotype and disease outcome of interest, a method known as Mendelian randomisation (MR). We aimed to identify genetic variants involved in the aetiology of alcohol consumption, and develop a PGRS for alcohol.

**Methods:**

Repeated measures of alcohol consumption from mothers and their offspring were collected as part of the Avon Longitudinal Study of Parents and Children. We tested the association between 89 SNPs (identified from either published GWAS data or from functional literature) and repeated measures of alcohol consumption, separately in mothers (from ages 28–48) and offspring (from ages 15–21) who had ever reported drinking. We modelled log units of alcohol using a linear mixed model and calculated beta coefficients for each SNP separately. Cross-validation was used to determine an allelic score for alcohol consumption, and the AVENGEME algorithm employed to estimate variance of the trait explained.

**Results:**

Following correction for multiple testing, one SNP (rs1229984) showed evidence for association with alcohol consumption (β = -0.177, SE = 0.042, *p* = <0.0001) in the mothers. No SNPs showed evidence for association in the offspring after correcting for multiple testing. The optimal allelic score was generated using p-value cut offs of 0.5 and 0.05 for the mothers and offspring respectively. These scores explained 0.3% and 0.7% of the variance.

**Conclusion:**

Our PGRS explains a modest amount of the variance in alcohol consumption and larger sample sizes would be required to use our PGRS in an MR framework.

## Introduction

Alcohol is a leading preventable cause of ill health in Europe [1], with Europeans accounting for more than a quarter of the total worldwide alcohol consumption (despite making up 15% of the global population) [2]. Despite this, uncertainty remains about the true extent by which alcohol consumption in the general population causes a number of health outcomes including type 2 diabetes [3] and cardiovascular disease [4] mainly because of bias in conventional epidemiological studies.

Genetic variants can be used in an instrumental variable framework to improve evidence on causality between an exposure and disease outcome of interest, a method known as Mendelian randomisation (MR) [5]. Details of the rationale and assumptions of MR have been discussed in detail elsewhere [6]. In brief, the allocation of genetic variants is random at conception, therefore the frequency of those variants associated with an exposure of interest should be approximately the same in groups of individuals with different confounding factors. Furthermore, as genotype is determined at conception, it cannot be susceptible to reverse causation [5, 7], which is particularly problematic when studying long-term effects of alcohol use (“sick-quitter effect” [3, 8]). Holmes *et al* (2014) [9] used rs1229984 (a genetic variant in *ADH1B*) in an MR framework to examine the causal impact of alcohol on cardiovascular disease in European populations while other examples can be found in East Asian populations [10–12]. Polygenic risk scores (PGRS) can also be used in an MR framework, accounting for a greater proportion of the variance in the exposure phenotype of interest, thus increasing power. Use of PGRS in MR can avoid or alleviate weak instrument bias [13], which is a common problem in MR. Furthermore, in the instance that researchers wish to use a PGRS in a large cohort that has genotyping availability but no GWAS data, a finite number of SNPs from the PGRS can be easily and cost effectively genotyped for that purpose.

There are clear advantages of using PGRS in MR studies of alcohol consumption, however to date there are no known variants robustly associated with alcohol drinking in populations of European origin, other than the relatively rare ADH1B used by Holmes et al [9]. This is despite estimates of heritability for alcohol use disorders and consumption reaching approximately 50% at their peak [14–17], and linkage and genome wide association studies (GWAS) suggesting a variety of potential loci that might be implicated [18–20]. The majority of GWAS for alcohol phenotypes focus on dependence rather than heaviness of use [21–24], and among the top findings are often alcohol dehydrogenase genes (*ADH)* and aldehyde dehydrogenase (*ALDH2*) [25–27], which have also been reported in candidate gene studies of metabolic reactions following ingestion [28].

We therefore aimed to identify genetic variants likely to play a role in the aetiology of alcohol consumption. Our end goal was to develop a polygenic risk score for average alcohol consumption in the general population, which could be specific to consumption and explain a larger proportion of the variance than the known ADH1B variant. We used a multi-step approach, by [a] Identifying genetic variants that could plausibly be associated with alcohol consumption from genome wide association studies (GWAS) and the functional literature, [b] Estimating their association with alcohol consumption in mothers (heritability is estimated to be higher after college years) and offspring from the Avon Longitudinal Study of Parents and Children (ALSPAC), and [c] Creating PGRSs (based on the initial set of SNPs) and estimating the proportion of variance explained for both mothers and offspring’s phenotypes. We fitted both cross-sectional and longitudinal models, thus taking advantage of the repeated measures of alcohol consumption available at different time points in life to minimise noise in the definition/reporting of alcohol use.

## Methods

### Study Population

Data were taken from the Avon Longitudinal Study of Parents and Children (ALSPAC), a longitudinal study situated in South West England. ALSPAC recruited 14,541 pregnant women between 1991 and 1992, with over 14,062 live births resulting from these pregnancies. Comparison with the 1991 census shows the sample was broadly representative of the British population [29]. Both mothers and offspring have been followed up with a series of questionnaires, clinics and lab-based assessments over the past 25 years, which has allowed for a wide range of phenotypic and biological measures to be collected. Ethical approval was obtained from the ALSPAC Ethics and Law Committee and the Local Research Ethics Committees. Further information of the recruitment process is available elsewhere [29–31]. The study website contains details of all data through a searchable data dictionary [32].

We used data from 10 postal questionnaires completed by the mothers in the cohort over 18 years (ranging from a mean age of 28 at baseline to a mean age of 48 at 18 years post pregnancy) and questionnaires completed by ALSPAC offspring over 6 years (ranging from age 15 to 21 years). All individuals who had available genetic data (outlined below) and answered alcohol related questions at these time points were included in the analysis (Mother N = 1609 to 3912; Offspring N = 2604 to 7989, Table 1).

**Table 1:** Summary information of weekly alcohol consumption across all time points.

Timepoint	Alcohol units per week				
	N	Mean units (SD)	Median units (IQR)	Zeros (%)	Mean age (SD)
**Child** ^**1**^					
**15 years (C)**	3912	2.5 (6.4)	0.5 (0–2)	39	15.1 (0.3)
**16 years (Q)**	3573	9.6 (10.9)	4.5 (0.38–16.5)	9	16.2 (0.4)
**17 years (C)**	3059	10.1 (9.9)	7.5 (1.38–16.5)	5	17.2 (0.4)
**18 years (Q)**	2427	13.3 (11.8)	8.25 (3.75–24)	6	18.2 (0.6)
**21 years (Q)**	2973	8.4 (9.6)	6 (2–8.75)	4	20.5 (0.5)
**All child**	17998	8.3 (10.3)	4.1 (0.6–13.8)	13	16.7 (2.3)
**Mother** ^2^					
**Baseline**	7425	3.6 (6.1)	3.5 (0.5–3.5)	7	28.2 (4.8)
**1 years (Q)**	6641	1.6 (3.3)	0.5 (0.5–1.5)	11	29.5 (4.7)
**2 years (Q)**	6224	2.5 (4.4)	0.5 (0.5–3.5)	13	30.7 (4.6)
**3 years (Q)**	5952	2.8 (4.7)	0.5 (0.5–3.5)	11	32.9 (4.5)
**4 years (Q)**	7989	3.6 (5.6)	1.0 (0–6)	47	34.0 (4.5)
**6 years (Q)**	5601	3.8 (6.1)	3.5 (0.5–3.5)	8	34.2 (4.5)
**7 years (Q)**	7973	4.3 (8.4)	0 (0–6)	52	36.3 (4.5)
**8 years (Q)**	7962	4.1 (8.7)	0 (0–6)	52	37.5 (4.4)
**12 years (Q)**	4379	6.5 (6.9)	5 (1–9)	19	41.5 (4.4)
**18 years (Q)**	2604	6.0 (6.5)	3.8 (1.1–8.3)	0	48.3 (4.3)
**All mother**	67988	3.7 (6.5)	1.1 (0–4)	24	34.0 (6.8)

^1^ All offspring’s time points represent the age at which the questionnaire/clinic was administered.

^2^ All mothers time points correspond to the amount of time since the end of the first pregnancy (i.e. pregnancy enrolled into ALSPAC); baseline corresponds to a questionnaire administered at enrolment into the study that reflects alcohol use *before* pregnancy.

(C) = data collected during a clinic session; (Q) data collected using a postal questionnaire.

### Phenotypic Measures

#### Weekly alcohol consumption (units)

In all alcohol related questions used in this research, participants were informed that “one drink referred to ½ pint of beer/cider, a small (125ml) glass of wine or a single (25ml) measure of spirit”, with each of these drinks containing approximately one UK unit of alcohol. Weekly alcohol consumption was treated as a continuous measure of UK units.

Mothers’ weekly alcohol consumption between zero and three years post pregnancy and at six years post pregnancy were calculated using the question “*How often have you drunk alcoholic drinks*”. Participants selected one of the following responses: “*Never*”; “*Less than 1 glass a week*”; “*At least 1 glass a week*”; “*1–2 glasses every day*”; “*At least 1–9 glasses every day*”; “*At least 10 glasses every day*”. At zero years post pregnancy, this question related to the amount of alcohol consumed *before* the current pregnancy. At four years post pregnancy and between seven and 12 years post pregnancy, weekly alcohol consumption was calculated from self-reported beers/ciders, wines, spirits, other alcohol or low alcohol beverages consumed on each day of the previous week. Weekly alcohol consumption at 18 years post pregnancy was calculated by multiplying the number of days the mother generally drank by the number of drinks consumed on a typical drinking data (S1 Table).

Offspring’s weekly alcohol consumption between ages 15 and 21 years was calculated by multiplying the frequency at which the child drank by the number of drinks consumed on a normal drinking day (S2 Table).

#### Potential covariates

Mothers’ covariates included: Cigarettes per day treated as a continuous measure at one, two, three and six years post pregnancy; age in years; social class (III manual skilled, IV and V unskilled manual or casual workers or those who rely on state for their income/I and II professional occupations and managerial and technical occupations and III non-manual skilled workers); highest level of education (certificate of secondary education/vocational qualification/O level/A level/Degree); cannabis, antidepressant, amphetamine and opiate use in the past year (No/Yes) at one, two, three and six years post pregnancy.

Offspring’s covariates included: Sex; ethnicity (white/other); DSM-IV classification of anxiety, depression, conduct disorder and ADHD at ages 7, 10, 13 and 15 years; Binge eating in the past year at ages 13, 14, 16 and 18 years; antisocial behaviour at ages 11, 13, 14, 15, 18, 19 and 21 years (all coded No/Yes); and mothers highest level of education (certificate of secondary education/vocational qualification/O level/A level/Degree).

### Genetic Measures

ALSPAC offspring were genotyped using the Illumina HumanHap550 quad chip genotyping platforms by 23andMe subcontracting the Wellcome Trust Sanger Institute, Cambridge, UK and the Laboratory Corporation of America, Burlington, NC, US. ALSPAC mothers were genotyped using the Illumina human660W-quad array at Centre National de Génotypage (CNG). Following quality control (individual call rate > 0.97, SNP call rate >0.95, MAF > 0.01, HWE > 1E-7, cryptic relatedness within mothers and within offspring IBD < 0.1, non-European clustering individuals removed) 9237 offspring and 8196 mothers were retained with 477482 SNP genotypes in common between them. SNPs were flipped to forward strand and haplotypes were estimated on the combined sample using ShapeIT (v2.r644). Imputation was performed using Impute V2.2.2 against all 2186 reference haplotypes (including non-Europeans) in the December 2013 release of the 1000 genomes reference haplotypes (Version 1 Phase 3). This gave 8237 eligible offspring and 8196 eligible mothers with available genotype data.

To identify SNPs with some *a priori* evidence of association with alcohol consumption/ potentially associated with alcohol consumption, we searched the NHGRI-EBI GWAS catalogue [33] for published GWAS of alcohol-related phenotypes, using the following search terms: “Alcohol consumption”; “Alcohol dependence”; and “Alcoholism”. 68 SNPs were associated with at least one of the latter phenotypes at a P value < 1.0 x 10^−5^. We supplemented this list with a search for candidate gene or functional studies of alcohol consumption, identifying a further 23 SNPs, bringing the total number of SNPs to 91 (note that three SNPs were identified from functional studies that had already been identified by the GWAS search). We then extracted genotype data for the 91 SNPs from ALSPAC. 31 SNPs were directly genotyped. For the remaining 60 SNPs, imputed genotypes with imputation r^2^>0.8 were available (two SNPs with imputation r2<0.8 were excluded). 89 SNPs were therefore included in the analyses. A full summary of selected SNPs is provided in S3 Table.

### Statistical Analysis

All analyses were conducted using Stata13 [34]. We tested for association between weekly alcohol consumption and the 89 SNPs in both mothers and offspring separately. For alcohol consumption, an abundance of zeros was expected, since no consumption would be reported both by those offspring and mothers who never drink, and by offspring and mothers who had not drank in the preceding week. To analyse cross-sectional and repeated measures of these data we log-transformed units and focussed analysis only on those who had ever reported drinking (i.e. dropping non-drinkers). We modelled log units using a linear mixed model and calculated the beta coefficient of each SNP as a function of the number of copies of the minor allele. The outcome was also assessed cross-sectionally using a log-linear regression at each time point separately. In each model, we adjusted for age and controlled for population stratification using the first 10 principal components. To test for pleiotropic effects, we examined the associations between the 89 SNPs and 48 potential confounders detailed above.

We used the Bonferroni method to correct for multiple testing. Evidence for association was taken at p = 0.00056 (0.05/89) for repeated measures analyses and p = 0.000037 (0.05/number of time points*89) for the cross sectional analyses.

To determine a PGRS for alcohol consumption, we randomly separated the individuals into 80% training and 20% discovery sets. Repeated measures of log units were modelled in the training set, separately for each of the 89 SNPs with beta coefficients, their standard error and corresponding p-values recorded. Using p-value thresholds of 0.01, 0.05, 0.1, 0.2, 0.4 and 0.5 for inclusion, we then created a weighted PGRS for each threshold. These scores were used to predict repeated measures of log-units in the 20% discovery set, with R-squared recorded for the score corresponding to each p-value threshold. The process was repeated five times, and the p-value threshold with the highest R-squared was taken as optimal. This was done independently for mothers and offspring data. Finally, the AVENGEME [35] algorithm was employed to estimate variance of the trait (i.e. alcohol units consumed in a week) explained by the PGRSs.

To demonstrate a possible use of the PGRS, we tested the association between our PGRS on proxy measures for cardiovascular disease [9] which were available both in mothers and in their offspring, namely HDL cholesterol, systolic blood pressure and diastolic blood pressure. These were modelled against the two PGRS while controlling for age at measurement.

### Sensitivity Analysis

The above statistical methods were used to conduct the following sensitivity analyses using the mother’s data: (a) excluding individuals who were pregnant at completion of the questionnaire; and (b) excluding weekly alcohol consumption measures at four, seven, eight and 12 years post pregnancy in the mothers as these questions were phrased differently from the other time points (S1 Table).

## Results

### ALSPAC Mothers

For the ALSPAC mothers, there were 67988 questionnaire responses to units consumed, between the ages of 28 to 48 years. Alcohol consumption increased over the course of the questionnaires and over age (Table 1).

**Fig 1:**
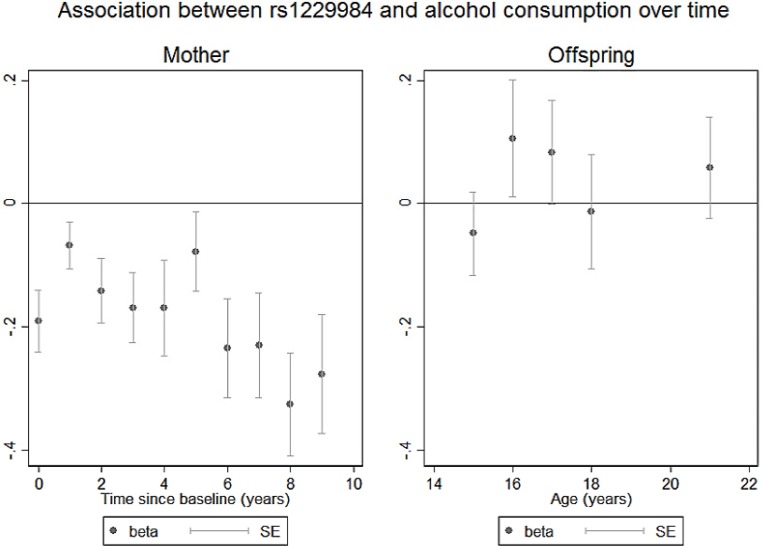
Association between rs1229984 and alcohol consumption over time.

In the repeated measures analysis, six SNPs had a *p* value < 0.05. Following correction for multiple testing, one SNP (rs1229984) showed evidence for association with alcohol consumption (β = -0.177, SE = 0.042, *p* = 0.00002) (Table 2 and S4 Table). In the cross sectional analyses, 27 SNPs had a *p* value < 0.05 at a minimum of one time point. The top ranked SNP was rs1229984 with the alcohol consumption variable measured at 12 years post pregnancy (difference in units per week for each additional copy of the minor allele = -0.326, SE = 0.084, P = 0.0001). This SNP also showed evidence for association (multiple testing threshold) at baseline (difference in units per week for each additional copy of the minor allele = -0.19, SE = 0.051, P = 0.0002) and was consistently negatively associated with alcohol consumption across all time points (Fig 1). (S5 Table). None of the mother’s covariates showed evidence for association with any of individual SNPs following correction for multiple testing (S6 Table).

**Table 2:** Top 20 SNPs for alcohol consumption (ranked by p value) for both mothers and offspring.

Offspring										Mothers									
Rank	SNP	n	Effect size	SE	*t*	p-value	χ^2^	R^2^	Rank in mother’s model	Rank	SNP	n	Effect size	SE	*t*	p-value	χ^2^	R^2^	Rank in offspring’s model
**1**	**rs2188561**	5016	-0.055	0.019	-2.855	0.0043	776.66	0.000995	51	**1**	**rs1229984**	5399	-0.177	0.042	-4.223	0.00002	14899.03	0.0005963	22
**2**	**rs8040009**	5016	0.056	0.021	2.711	0.0067	774.80	0.000284	44	**2**	**rs1497571**	7457	0.024	0.011	2.248	0.0246	20111.04	0.0007266	36
**3**	**rs567926**	5016	-0.081	0.032	-2.548	0.0108	775.92	0.000167	70	**3**	**rs4478858**	7457	-0.024	0.011	-2.221	0.0264	20118.68	0.0002592	84
**4**	**rs2154294**	5016	-0.038	0.016	-2.339	0.0193	775.76	0.000205	77	**4**	**rs6902771**	7457	0.023	0.011	2.154	0.0312	20121.22	0.0005803	34
**5**	**rs11851015**	5016	0.049	0.023	2.099	0.0358	775.65	0.000323	25	**5**	**rs11724320**	7457	-0.023	0.011	-2.007	0.0448	20116.57	0.0004777	67
**6**	**rs4293630**	5016	-0.046	0.023	-1.984	0.0473	774.09	0.000274	33	**6**	**rs1876831**	7457	-0.025	0.013	-1.962	0.0497	20116.56	0.0002741	83
**7**	**rs279861**	5016	-0.059	0.032	-1.834	0.0667	776.89	0.000108	89	**7**	**rs1318937**	7457	0.031	0.016	1.944	0.0520	20124.18	0.0001302	23
**8**	**rs2548145**	5016	-0.029	0.016	-1.807	0.0708	775.12	0.000201	79	**8**	**rs1380131**	7457	0.034	0.019	1.829	0.0674	20121.28	0.0003891	33
**9**	**rs1353621**	5016	0.028	0.017	1.693	0.0904	776.08	0.000389	45	**9**	**rs2100290**	7457	-0.019	0.011	-1.775	0.0759	20110.84	0.0002867	24
**10**	**rs3819197**	5016	-0.031	0.019	-1.668	0.0954	776.65	0.000194	76	**10**	**rs1230165**	7457	0.024	0.014	1.710	0.0872	20119.48	0.0003098	27
**11**	**rs4758317**	5016	-0.027	0.016	-1.644	0.1003	776.67	0.00022	56	**11**	**rs1353899**	7457	-0.023	0.014	-1.704	0.0884	20117.89	0.0003524	53
**12**	**rs1573496**	5016	0.044	0.027	1.631	0.1029	777.31	3.22E-05	20	**12**	**rs750338**	7457	0.022	0.013	1.701	0.0889	20127.06	0.0002061	68
**13**	**rs7590720**	5016	-0.029	0.018	-1.613	0.1068	777.92	0.000219	63	**13**	**rs3131513**	7457	0.018	0.011	1.648	0.0993	20118.56	0.0002538	54
**14**	**rs9512637**	5016	0.027	0.017	1.606	0.1082	776.59	6.93E-05	30	**14**	**rs9656709**	7457	0.017	0.011	1.601	0.1093	20125.56	0.0002202	77
**15**	**rs1908556**	5016	0.036	0.023	1.565	0.1176	776.85	0.000293	49	**15**	**rs4770403**	7457	-0.022	0.014	-1.577	0.1148	20115.18	0.0002771	89
**16**	**rs12311304**	5016	-0.026	0.017	-1.538	0.1241	777.42	0.000319	27	**16**	**rs642899**	7457	0.020	0.013	1.564	0.1177	20111.39	0.0002185	47
**17**	**rs237238**	5016	0.048	0.031	1.514	0.1299	778.00	0.000121	80	**17**	**rs933769**	7457	-0.021	0.014	-1.496	0.1346	20125.24	0.0001654	55
**18**	**rs62202398**	5016	-0.048	0.034	-1.423	0.1547	777.74	0.000192	34	**18**	**rs3764435**	7457	0.015	0.011	1.391	0.1644	20118.54	0.0001952	64
**19**	**rs1800759**	5016	0.023	0.016	1.418	0.1561	777.35	0.000405	26	**19**	**rs59972978**	7457	0.020	0.014	1.385	0.1661	20110.16	0.0001889	29
**20**	**rs1864982**	5016	0.033	0.023	1.409	0.1588	777.53	9.22E-06	57	**20**	**rs1573496**	7457	-0.025	0.018	-1.379	0.1680	20128.82	0.0001741	12
**PGRS**	**6 SNPs**	5016	0.399	0.076	5.230	<0.001	761.62	0.0066	-	**PGRS**	**42 SNPs**	7457	0.553	0.073	7.540	<0.001	19916.96	0.0030	-

Effect sizes explain increase in units of alcohol consumed per week for each additional copy of the minor allele.

### ALSPAC Offspring

Units of alcohol consumed were measured 17998 times over 5 questionnaires during adolescence for the ALSPAC offspring, from age 15 to 20.5. The average number of units peaked at 13 per week at age 18 years, dropping to 8 per week by age 21 years (Table 1).

In the repeated measures analysis, six SNPs had a *p* value < 0.05. Following correction for multiple testing, no SNPs showed evidence for association (Table 2 and S4 Table). In cross sectional analyses, 28 SNPs had a p value < 0.05 at a minimum of one time point. The top ranked SNP in the offspring was rs2228093 with the alcohol consumption variable measures at age 18 years (difference in units per week for each additional copy of the minor allele = -0.105, SE = 0.036, P = 0.004), however this did not meet the *p* value threshold for multiple testing (S5 Table). In contrast to the ALSPAC mothers, rs1229984 did not pass multiple testing thresholds for alcohol consumption in repeated measures analysis and did not show a consistent pattern across all time points tested (Fig 1). None of the offspring’s covariates showed evidence for association with any of individual SNPs following correction for multiple testing (S6 Table).

### Sensitivity Analysis

When excluding non-pregnant women and data from questionnaires at four, seven, eight and 12 years post pregnancy, rs1229984 remained the only SNP associated with weekly alcohol consumption (*excluding pregnant women*: increase in units per week for each additional copy of the minor allele = -0.159, SE = 0.042, P = 0.0001; *excluding questionnaires*: increase in units per week for each additional copy of the minor allele = -0.149, SE = 0.039, P = 0.0001) (S7 Table).

### Polygenic Risk Score

The best allelic score was generated using p value cut offs of 0.5 and 0.05 for the mothers and offspring, respectively, which resulted in including a total of 42 SNPs (out of 89) for mothers and 6 SNPs (out of 89) for offspring. For the ALSPAC mothers, the variance in units of alcohol per week explained was 0.3% (95% CI 0.13% to 0.76%). For the ALSPAC offspring the variance explained was 0.66% (95% CI 0.22% to 1.3%). Neither of the PGRS showed evidence for association with any of the confounders, i.e. there was no evidence to suggest that the PGRS could be violating the second assumption of instrumental variables (that the instrument is independent of the confounders of the original exposure-outcome association) and therefore being invalid as an instrumental variable to proxy for alcohol intake (S6 Table).

When modelling the effect of our PGRS on cardiovascular disease risk factors, there was evidence for association between the offspring PGRS and offspring diastolic blood pressure in the expected direction (beta = 1.61, 95% CI 0.25 to 2.97, p = 0.020). However, our estimates of the association between the offspring PGRS and both HDL cholesterol and systolic blood pressure provided no strong evidence of association (HDL cholesterol: beta = 0.06, 95% CI -0.03 to 0.14, p = 0.177; systolic blood pressure: beta = 1.44, 95% CI -0.84 to 3.72, p = 0.216). Similarly, there was no statistical evidence for association between the mothers PGRS and any of the cardiovascular disease risk factors (HDL cholesterol beta = 0.39, 95% CI -0.10 to 0.17, p = 0.565; systolic blood pressure beta = -0.68, 95% CI -7.05 to 5.69, p = 0.0.835; diastolic blood pressure beta = 2.14, 95% CI -2.34 to 6.62, p = 0.350).

## Discussion

The aim of this study was to develop a polygenic risk score for alcohol consumption, in view of using this to assess the causal impact of alcohol on health related outcomes such as cardiovascular disease. Literature searches of published GWAS and functional studies identified 89 candidate SNPs that had previously shown some evidence of association with alcohol-related phenotypes. Using repeated measures analysis of alcohol behaviour over the course of a 20 year period, we found strong evidence confirming that rs1229984 plays a role in alcohol consumption, confirming previous results [9]. This SNP was associated with a decrease of 0.84 units of alcohol per week, on average. It was found to be associated in cross-sectional analyses of questionnaires measured 20 years apart and effect estimates were stronger in the repeated measures analysis, strengthening the evidence that it relates to alcohol consumption throughout the life course. The PGRS derived through cross-validation only explained a modest proportion of the variance in alcohol consumption (0.3% for mothers and 0.66% for the offspring in our sample).

The score could in principle be used to conduct MR analyses for example in the field of cardiovascular disease (CVD), although large sample sizes would be required. The British Heart Foundation estimates that there are 7m people living with CVD in the UK (~10% of the population) [36], if the odds ratio for alcohol consumption on CVD incidence was 0.75 (OR for CVD mortality used) [37], we would require a sample size of 126,500 (assuming a 1:1 ratio, 80% power, alpha = 0.05 and R^2^ = 0.3% from the mothers PGRS result), or 54,200 (assuming a 1:1 ratio, 80% power, alpha = 0.05 and R^2^ = 0.7% from the offspring’s PGRS result)[38]. Similarly, the incidence odds ratio for coronary heart disease (CHD) is 0.71 [37], with 2.3 million in the UK living with CHD. To perform an MR analysis to examine the effect of alcohol consumption on CHD using the mothers PGRS we would need 89,300 with 38,300 individuals needed for the offspring’s PGRS. The required sample sizes are much larger than those in our sample, therefore our tests of association between the PGRSs and proxy measures of cardiovascular disease are underpowered. As such, we cannot be certain that the lack of associations are representative of null results.

### Strengths and limitations

ALSPAC is a well characterised birth cohort with repeated measures of alcohol use, which have been used in several other studies of substance use [39–41]. Moreover, the available data were collected on mothers and their offspring over the course of 20 years. As such, they are an excellent resource to investigate alcohol behaviour over time. The nature of this dataset allowed for the use of repeated measures to strengthen the phenotype. Furthermore, the wealth of additional data allowed for detailed sensitivity analyses and examining of a wide range of potential covariates to test for pleiotropic effects of the alcohol variants and the derived PGRSs. An additional strength comes from the way our PGRS was constructed. By taking a limited number of SNPs that have previously shown some evidence of association with alcohol behaviours (either from GWAS or functional literature) we have developed a PGRS that has a reduced number of SNPs compared to the numbers that might be required if using p value cut offs from whole GWAS. The advantage to this approach is that the resulting PGRS is less likely to have pleiotropic effects than one from a deep GWAS list. Furthermore, this finite number of SNPs would therefore be more cost effective to genotype and could, therefore, be feasibly used in a study that does not have access to genome wide data.

There are also some limitations which need to be considered when interpreting the results of this study. First, the set of SNPs identified through searches came from GWAS analyses of alcohol dependence [21–24] and so might not show an association with our phenotype (units of alcohol per week). Meanwhile, functional literature reports the role of genetic variants in metabolism, however the effects of these genes are not taken as far as alcohol consumption. Second, alcohol consumption questions were not uniform over time, however sensitivity analyses excluding data from a different version of the questionnaire returned similar results. Third, our data on alcohol consumption are based on self-report and so may be subject to misclassification. However, there are currently no reliable biological alternatives for alcohol use in a general population sample [42], with current biomarkers only being able to identify long term heavy use [43, 44]. One might expect to find that the direction of bias differs in the two populations of mothers and offspring, as mothers might underreport their use (negatively impacting estimates), while offspring might over-report their consumption (positively impacting estimates) [45–47]. Fourth, there was loss to follow up, with greater proportions of missing data in later questionnaires, which reduced statistical power and could lead to selection bias if alcohol consumption is related to the loss to follow up. This drop in sample size also meant that stratifying the offspring analysis by gender would reduce the power, however we did adjust for gender in this analysis. Furthermore, we were unable to examine the association between SNPs and alcohol consumption in adult males (i.e. fathers) as their genetic data was not available. Finally, we found no suitable independent cohort study with life course alcohol consumption data for testing PGRS performance and hence we used cross-validation in ALSPAC. However, it has previously been reported that sample sizes such as those used in this analysis are adequate when using two separate ‘training’ and ‘testing’ samples [48].

### Findings in relation to other research

Burgess and colleagues suggested that variants with known biology are better for use in MR studies [49]. The underlying biology of some of the SNPs (those selected from functional literature) included in our analysis is known, and linked to changes in alcohol metabolism, and as such, would be better for use in a PGRS MR analysis. One SNP (rs1229984 in *ADH1B*) was consistently identified as being associated with alcohol consumption in ALSPAC mothers. This SNP has previously been used in an MR framework, after Holmes and colleagues validated it as a genetic instrument by providing solid evidence for association with various alcohol phenotypes (including units of alcohol per week) in a sample of >200,000 participants [9]. In their estimate, carriers of the minor allele consumed 17.2% fewer units per week than non-carriers, which is very similar to our result of 0.177 fewer log units per week (equivalent to 16.2% fewer units per week).

The set of SNPs included in the mothers PGRS and offspring PGRS were different, possibly due to age and gender effects. Previous literature has suggested that the heritability of alcohol consumption changes across the life course. Estimates start to increase at the age of 15 years and peak in the mid-20’s [14–17]. It is therefore possible that the offspring are so young that their genetic potential to abuse or avoid alcohol is not yet fully expressed. Conversely, the age of the ALSPAC mothers at baseline ranged from 14 to 46 years (mean = 28 years), with these individuals being followed up for 20 years. Since the mothers’ longitudinal analyses cover a wide range of ages across the life course, it is not possible to make assumptions about the impact of age on the PGRS composition or the proportion of the variance it explains, in relation to the offspring’s PGRS. Additionally, gender differences may also have a role if there are systematic differences in alcohol consumption by gender. However, stratifying by gender here would reduce power in the analysis.

PGRS have previously been used in an MR framework to evaluate the causal effect of a number of traits/exposures, with proportions of the variance explained in the trait in the range or 1.5–3% (e.g. BMI: 1.5% - 2.5% [50–52], type 2 diabetes: ~2% [53], schizophrenia: ~3% [54]). However, the proportion of the variance explained by our PGRS is comparable to the variance of age of onset of alcohol consumption explained by a previously reported PGRS [55]. In our analysis, the variation explained by the initial 89 SNPs selected was estimated to be between 0.13% and 0.76% for the ALSPAC mothers. A previous PGRS for tobacco (cigarettes smoked per day) was shown to explain 0.4–0.5% of the variance in glasses of alcohol per week [56], which is comparable in magnitude to the variance explained by our PGRS for alcohol consumption. This provides additional evidence that some genetic risk factors are shared between substances, suggesting that incorrect effect estimates could be introduced through pleiotropy. However, the lack of evidence for association between our PGRSs and potential confounders (including tobacco and other drug use) suggesting minimal evidence for pleiotropy. These comparisons are limited by design differences (i.e. genome wide analysis in previous literature compared to the candidate gene approach here). However, in theory, our selection process identified ‘a-priori’ candidates and we would therefore expect a higher proportion of the variance to be explained in this analysis. This highlights how little we know about the genetic contribution to alcohol consumption.

## Conclusion

The PGRSs developed in our analyses explained a modest proportion of the variance in alcohol consumption for both ALSPAC mothers and offspring. For future MR analyses examining the causal effects of drinking alcohol in the general population, the mothers’ PGRS reported here is most likely a more suitable genetic proxy as it is based on a breadth of ages, although one limitation to this discovery sample is the inclusion of women only. Very large sample sizes, such as those from multi-study consortia, would be required if these PGRSs were to be used as genetic instruments in MR analyses.

## Supporting Information

S1 Table Mothers questionnaire information–Alcohol consumption. (DOCX)

S2 Table Offspring questionnaire information–Alcohol consumption. (DOCX)

S3 Table SNP Information. (DOCX)

S4 Table All results for repeated measures alcohol consumption in ALSPAC mothers and offspring. (DOCX)

S5 Table All results for cross sectional alcohol consumption, mothers (time points M0 to M18) and offspring (times points C15 to C21). (DOCX)

S6 Table Associations between PGRS, individual SNPs and potential confounders (Bonferroni corrected p-value = 0.00011). (DOCX)

S7 Table Sensitivity analysis for repeated measures analysis in ALSPAC mothers. (DOCX)

## References

[1] Rehm J, Shield K, Rehm M, Gmel G, Frick U. Alcohol consumption, alcohol dependence and attributable burden of disease in Europe. Centre for Addiction and Mental Health. 2012.

[2] Organization WH. Global status report on alcohol and health, 2014. 2014.

[3] Koppes LL, Dekker JM, Hendriks HF, Bouter LM, Heine RJ. Moderate Alcohol Consumption Lowers the Risk of Type 2 Diabetes A meta-analysis of prospective observational studies. Diabetes care. 2005;28(3):719–25. 15735217 10.2337/diacare.28.3.719

[4] Glymour M. Alcohol and cardiovascular disease. Bmj. 2014;349:g4334 10.1136/bmj.g4334 25011451

[5] Lawlor DA, Harbord RM, Sterne JA, Timpson N, Davey Smith G. Mendelian randomization: using genes as instruments for making causal inferences in epidemiology. Statistics in medicine. 2008;27(8):1133–63. 10.1002/sim.3034 17886233

[6] Smith GD, Hemani G. Mendelian randomization: genetic anchors for causal inference in epidemiological studies. Human molecular genetics. 2014;23(R1):R89–R98. 10.1093/hmg/ddu328 25064373 PMC4170722

[7] Smith GD, Lawlor DA, Harbord R, Timpson N, Day I, Ebrahim S. Clustered environments and randomized genes: a fundamental distinction between conventional and genetic epidemiology. PLoS medicine. 2007;4(12):e352 PubMed Central PMCID: PMC2121108. 10.1371/journal.pmed.0040352 18076282 PMC2121108

[8] Rehm J, Irving H, Ye Y, Kerr WC, Bond J, Greenfield TK. Are lifetime abstainers the best control group in alcohol epidemiology? On the stability and validity of reported lifetime abstention. American journal of epidemiology. 2008;168(8):866–71. 10.1093/aje/kwn093 18701442 PMC2565735

[9] Holmes MV, Dale CE, Zuccolo L, Silverwood RJ, Guo Y, Ye Z, et al Association between alcohol and cardiovascular disease: Mendelian randomisation analysis based on individual participant data. Bmj. 2014;349:g4164 10.1136/bmj.g4164 25011450 PMC4091648

[10] Irons DE, McGue M, Iacono WG, Oetting WS. Mendelian randomization: a novel test of the gateway hypothesis and models of gene-environment interplay. Development and psychopathology. 2007;19(4):1181–95. 10.1017/S0954579407000612 17931442

[11] Yeung SLA, Jiang C, Cheng KK, Liu B, Zhang W, Lam TH, et al Is aldehyde dehydrogenase 2 a credible genetic instrument for alcohol use in Mendelian randomization analysis in Southern Chinese men? International journal of epidemiology. 2013;42(1):318–28. 10.1093/ije/dys221 23243119

[12] Taylor AE, Lu F, Carslake D, Hu Z, Qian Y, Liu S, et al Exploring causal associations of alcohol with cardiovascular and metabolic risk factors in a Chinese population using Mendelian randomization analysis. Scientific reports. 2015;5. 10.1038/srep14005 PMC4568464 26364564

[13] Burgess S, Thompson SG. Use of allele scores as instrumental variables for Mendelian randomization. International journal of epidemiology. 2013;42(4):1134–44. 10.1093/ije/dyt093 24062299 PMC3780999

[14] Bevilacqua L, Goldman D. Genes and addictions. Clinical pharmacology and therapeutics. 2009;85(4):359 10.1038/clpt.2009.6 19295534 PMC2715956

[15] Verhulst B, Neale M, Kendler K. The heritability of alcohol use disorders: a meta-analysis of twin and adoption studies. Psychological medicine. 2015;45(05):1061–72. 25171596 10.1017/S0033291714002165 PMC4345133

[16] Bergen SE, Gardner CO, Kendler KS. Age-related changes in heritability of behavioral phenotypes over adolescence and young adulthood: a meta-analysis. Twin Research and Human Genetics. 2007;10(03):423–33. 17564500 10.1375/twin.10.3.423

[17] Swan GE, Carmelli D, Rosenman RH, Fabsitz RR, Christian JC. Smoking and alcohol consumption in adult male twins: genetic heritability and shared environmental influences. J Subst Abuse. 1990;2(1):39–50. 2136102 10.1016/s0899-3289(05)80044-6

[18] Li MD, Burmeister M. New insights into the genetics of addiction. Nature Reviews Genetics. 2009;10(4):225–31. 10.1038/nrg2536 19238175 PMC2879628

[19] Edenberg HJ. The genetics of alcohol metabolism: role of alcohol dehydrogenase and aldehyde dehydrogenase variants. Alcohol Research & Health. 2007;30(1):5–14. 17718394 PMC3860432

[20] Prescott C, Sullivan P, Kuo P, Webb B, Vittum J, Patterson De, et al Genomewide linkage study in the Irish affected sib pair study of alcohol dependence: evidence for a susceptibility region for symptoms of alcohol dependence on chromosome 4. Molecular psychiatry. 2006;11(6):603–11. 10.1038/sj.mp.4001811 16534506

[21] Gelernter J, Kranzler H, Sherva R, Almasy L, Koesterer R, Smith A, et al Genome-wide association study of alcohol dependence: significant findings in African-and European-Americans including novel risk loci. Molecular psychiatry. 2014;19(1):41–9. 10.1038/mp.2013.145 24166409 PMC4165335

[22] Bierut LJ, Agrawal A, Bucholz KK, Doheny KF, Laurie C, Pugh E, et al A genome-wide association study of alcohol dependence. Proceedings of the National Academy of Sciences. 2010;107(11):5082–7. 10.1073/pnas.0911109107 PMC2841942 20202923

[23] Treutlein J, Cichon S, Ridinger M, Wodarz N, Soyka M, Zill P, et al Genome-wide association study of alcohol dependence. Archives of general psychiatry. 2009;66(7):773–84. 10.1001/archgenpsychiatry.2009.83 19581569 PMC4229246

[24] Heath AC, Whitfield JB, Martin NG, Pergadia ML, Goate AM, Lind PA, et al A quantitative-trait genome-wide association study of alcoholism risk in the community: findings and implications. Biological psychiatry. 2011;70(6):513–8. 10.1016/j.biopsych.2011.02.028 21529783 PMC3210694

[25] Park BL, Kim JW, Cheong HS, Kim LH, Lee BC, Seo CH, et al Extended genetic effects of ADH cluster genes on the risk of alcohol dependence: from GWAS to replication. Human genetics. 2013;132(6):657–68. 10.1007/s00439-013-1281-8 23456092

[26] Dick DM, Foroud T. Candidate genes for alcohol dependence: a review of genetic evidence from human studies. Alcoholism: Clinical and Experimental Research. 2003;27(5):868–79. 10.1097/01.ALC.0000065436.24221.63 12766633

[27] Treutlein J, Rietschel M. Genome-wide association studies of alcohol dependence and substance use disorders. Current psychiatry reports. 2011;13(2):147–55. 10.1007/s11920-011-0176-4 21253885

[28] Birley AJ, James MR, Dickson PA, Montgomery GW, Heath AC, Martin NG, et al ADH single nucleotide polymorphism associations with alcohol metabolism in vivo. Human molecular genetics. 2009;18(8):1533–42. 10.1093/hmg/ddp060 19193628 PMC2664151

[29] Golding J, Pembrey M, Jones R, Team AS. ALSPAC—the Avon Longitudinal Study of Parents and Children. I. Study methodology. Paediatr Perinat Epidemiol. 2001;15(1):74–87. 11237119 10.1046/j.1365-3016.2001.00325.x

[30] Boyd A, Golding J, Macleod J, Lawlor DA, Fraser A, Henderson J, et al Cohort Profile: the 'children of the 90s'—the index offspring of the Avon Longitudinal Study of Parents and Children. International journal of epidemiology. 2013;42(1):111–27. PubMed Central PMCID: PMC3600618. 10.1093/ije/dys064 22507743 PMC3600618

[31] Fraser A, Macdonald-Wallis C, Tilling K, Boyd A, Golding J, Davey Smith G, et al Cohort Profile: the Avon Longitudinal Study of Parents and Children: ALSPAC mothers cohort. International journal of epidemiology. 2013;42(1):97–110. PubMed Central PMCID: PMC3600619. 10.1093/ije/dys066 22507742 PMC3600619

[32] ALSPAC. Data Dictionary http://www.bris.ac.uk/alspac/researchers/data-access/data-dictionary/; archived at http://www.webcitation.org/6Tgld7Ze02014.

[33] Welter D, MacArthur J, Morales J, Burdett T, Hall P, Junkins H, et al The NHGRI GWAS Catalog, a curated resource of SNP-trait associations. Nucleic acids research. 2014;42(D1):D1001–D6. 24316577 10.1093/nar/gkt1229 PMC3965119

[34] StataCorp. Stata Statistical Software: Release 13. 2013.

[35] Palla L, Dudbridge F. A fast method that uses polygenic scores to estimate the variance explained by genome-wide marker panels and the proportion of variants affecting a trait. The American Journal of Human Genetics. 2015;97(2):250–9. 10.1016/j.ajhg.2015.06.005 26189816 PMC4573448

[36] BHF. Cardiovascular disease statistics 2015: British Heart Foundation; 2015 [09 June 2016]. Available from: https://www.bhf.org.uk/research/heart-statistics/heart-statistics-publications/cardiovascular-disease-statistics-2015.

[37] Ronksley PE, Brien SE, Turner BJ, Mukamal KJ, Ghali WA. Association of alcohol consumption with selected cardiovascular disease outcomes: a systematic review and meta-analysis. Bmj. 2011;342:d671 10.1136/bmj.d671 21343207 PMC3043109

[38] Brion M-JA, Shakhbazov K, Visscher PM. Calculating statistical power in Mendelian randomization studies. International journal of epidemiology. 2013;42(5):1497–501. 10.1093/ije/dyt179 24159078 PMC3807619

[39] Heron J, Macleod J, Munafo MR, Melotti R, Lewis G, Tilling K, et al Patterns of alcohol use in early adolescence predict problem use at age 16. Alcohol and alcoholism. 2012;47(2):169–77. PubMed Central PMCID: PMC3284685. 10.1093/alcalc/agr156 22215001 PMC3284685

[40] Melotti R, Heron J, Hickman M, Macleod J, Araya R, Lewis G, et al Adolescent alcohol and tobacco use and early socioeconomic position: the ALSPAC birth cohort. Pediatrics. 2011;127(4):e948–55. Epub 2011/03/16. 10.1542/peds.2009-3450 21402626

[41] MacArthur GJ, Smith MC, Melotti R, Heron J, Macleod J, Hickman M, et al Patterns of alcohol use and multiple risk behaviour by gender during early and late adolescence: the ALSPAC cohort. Journal of public health (Oxford, England). 2012;34 Suppl 1:i20–30. PubMed Central PMCID: PMC3284864. 10.1093/pubmed/fds006 PMC3284864 22363027

[42] Lees R, Kingston R, Williams T, Henderson G, Lingford-Hughes A, Hickman M. Comparison of Ethyl Glucuronide in Hair with Self-Reported Alcohol Consumption. Alcohol and alcoholism. 2012;47(3):267–72. 10.1093/alcalc/ags010 22336766

[43] Peterson K. Biomarkers for alcohol use and abuse-a summary. Alcohol Research and Health. 2004;28(1):30 19006989 PMC6601655

[44] Achur RN, Freeman WM, Vrana KE. Circulating cytokines as biomarkers of alcohol abuse and alcoholism. Journal of Neuroimmune Pharmacology. 2010;5(1):83–91. 10.1007/s11481-009-9185-z 20020329 PMC2862254

[45] Midanik L. The validity of self‐reported alcohol consumption and alcohol problems: A literature review. British journal of addiction. 1982;77(4):357–82. 6762224 10.1111/j.1360-0443.1982.tb02469.x

[46] Stockwell T, Donath S, Cooper‐Stanbury M, Chikritzhs T, Catalano P, Mateo C. Under‐reporting of alcohol consumption in household surveys: a comparison of quantity–frequency, graduated–frequency and recent recall. Addiction. 2004;99(8):1024–33. 10.1111/j.1360-0443.2004.00815.x 15265099

[47] Edwards AL. The social desirability variable in personality assessment and research1957.

[48] Dudbridge F. Power and predictive accuracy of polygenic risk scores. PLoS genetics. 2013;9(3):e1003348 10.1371/journal.pgen.1003348 23555274 PMC3605113

[49] Burgess S, Butterworth AS, Thompson JR. Beyond Mendelian randomization: how to interpret evidence of shared genetic predictors. Journal of clinical epidemiology. 2016;69:208–16. 10.1016/j.jclinepi.2015.08.001 26291580 PMC4687951

[50] Clarke T, Hall L, Fernandez-Pujals A, MacIntyre D, Thomson P, Hayward C, et al Major depressive disorder and current psychological distress moderate the effect of polygenic risk for obesity on body mass index. Translational psychiatry. 2015;5(6):e592. 26125155 10.1038/tp.2015.83 PMC4490293

[51] Hung C-F, Breen G, Czamara D, Corre T, Wolf C, Kloiber S, et al A genetic risk score combining 32 SNPs is associated with body mass index and improves obesity prediction in people with major depressive disorder. BMC medicine. 2015;13(1):1. 25903154 10.1186/s12916-015-0334-3 PMC4407390

[52] Walter S, Kubzansky LD, Koenen KC, Liang L, Tchetgen Tchetgen EJ, Cornelis MC, et al Revisiting mendelian randomization studies of the effect of body mass index on depression. American Journal of Medical Genetics Part B: Neuropsychiatric Genetics. 2015;168(2):108–15. 10.1002/ajmg.b.32286 PMC4387873 25656382

[53] Shen L, Walter S, Melles RB, Glymour MM, Jorgenson E. Diabetes Pathology and Risk of Primary Open-Angle Glaucoma: Evaluating Causal Mechanisms by Using Genetic Information. American journal of epidemiology. 2016;183(2):147–55. 10.1093/aje/kwv204 26608880 PMC4706681

[54] Taylor AE, Burgess S, Ware JJ, Gage SH, Richards JB, Smith GD, et al Investigating causality in the association between 25 (OH) D and schizophrenia. Scientific reports. 2016;6:26496 10.1038/srep26496 27215954 PMC4877705

[55] Chou Y, Madden P, Bierut L, Heath A, Bucholz K, Agrawal A. Genome-wide polygenic scores for age at onset of alcohol dependence and association with alcohol-related measures. Translational Psychiatry. 2016;22:e761. 10.1038/tp.2016.27 PMC4872451 27003187

[56] Vink JM, Hottenga JJ, de Geus EJ, Willemsen G, Neale MC, Furberg H, et al Polygenic risk scores for smoking: predictors for alcohol and cannabis use? Addiction. 2014;109(7):1141–51. PubMed Central PMCID: PMC4048635. 10.1111/add.12491 24450588 PMC4048635

