# Supplementary material for: Exploration of a Polygenic Risk Score for Alcohol Consumption: A Longitudinal Analysis from the ALSPAC Cohort
Source: PLoS One. 2016 Nov 30;11(11):e0167360. doi: 10.1371/journal.pone.0167360 (PMC5130278; doi:10.1371/journal.pone.0167360)
Supplement: S1 Table — (DOCX) [file pone.0167360.s001.docx]

**S1 Table: Mothers questionnaire information – Alcohol consumption**

| **Time point** | **Question asked** | **Answers given** | **Variable derivation** |
| --- | --- | --- | --- |
| **Baseline** | How often have you drunk alcohol drinks before this pregnancy? | Never, less than one glass a week, at least once or twice a week, between 3 & 6 times a week, 1-2 glasses per day, 3-9 glasses a day, at least 10 glasses a day | Recoded as 0, 0.5, 1.5, 4.5, 10, 42, 70 to provide a conservative measure of units of alcohol consumed per week |
| **1 years (Q)** | How would you describe you alcohol drinking? |  |  |
| **2 years (Q)** | How much alcohol do you drink? |  |  |
| **3 years (Q)** | How much alcohol do you drink? |  |  |
| **4 years (Q)** | During the last week how many of each type of alcoholic drink did you have on each day? | Number of glasses of beer, lager, cider, wine, spirits, low alcohol drinks and other alcoholic drinks on Monday, Tuesday etc | Sum of each drink on each day to provide units consumed in the past week |
| **6 years (Q)** | How much alcohol do you drink? | Never, less than one glass a week, at least once or twice a week, between 3 & 6 times a week, 1-2 glasses per day, 3-9 glasses a day, at least 10 glasses a day | Recoded as 0, 0.5, 1.5, 4.5, 10, 42, 70 to provide a conservative measure of units of alcohol consumed per week |
| **7 years (Q)** | During the last week how many of each type of alcoholic drink did you have on each day? | Number of glasses of beer, lager, cider, wine, spirits, low alcohol drinks and other alcoholic drinks on Monday, Tuesday etc | Sum of each drink on each day to provide units consumed in the past week |
| **8 years (Q)** |  |  |  |
| **12 years (Q)** |  |  |  |
| **18 years (Q)** | 1. How often do you have a drink containing alcohol 2. How many drinks containing alcohol do you have on a typical drinking day? | 1. Never, monthly or less, 2-4 times a month, 2-3 times a week, 4 or more times a week 2. 1-2, 3-4, 5-6, 7-9, 10 or more | 1. Recoded to 0, 0.25, 0.75, 2.5, 4 to represent number of days consumed alcohol per week 2. Recoded as 1.5, 3.5, 5.5, 8, 10 3. Multiply (a) and (b) to obtained measure of weekly units per week |

All mothers time points correspond to the amount of time since the end of the first pregnancy (i.e. pregnancy enrolled into ALSPAC); baseline corresponds to a questionnaire administered at enrolment into the study that reflects alcohol use *before* pregnancy

At all time points, participants were informed that a glass is the equivalent of a single measure (1oz) of spirit, ½ pint of beer or cider or a small glass (125ml) of wine.

(Q) data collected using a postal questionnaire
