# Supplementary material for: Exploration of a Polygenic Risk Score for Alcohol Consumption: A Longitudinal Analysis from the ALSPAC Cohort
Source: PLoS One. 2016 Nov 30;11(11):e0167360. doi: 10.1371/journal.pone.0167360 (PMC5130278; doi:10.1371/journal.pone.0167360)
Supplement: S2 Table — (DOCX) [file pone.0167360.s002.docx]

**S2 Table: Offspring questionnaire information – Alcohol consumption**

| **Time point** | **Question asked** | **Answer given** | **Variable derivation** |
| --- | --- | --- | --- |
| **15 years (C)** | 1. How regularly have you had a drink containing alcohol? 2. How many drinks do you have on a typical drinking day | 1. Never, Tried once or twice, used sometimes never now, less than once p/w, 1/2 days p/w, >2 days p/w, every day 2. Participant provided a number | 1. Recoded as 0, 0, 0, 0.5, 1.5, 4, 7 2. Multiplied by (a) to get a measure of units consumed per week |
| **16 years (Q)** | 1. How often do you have a drink containing alcohol? 2. How many units containing alcohol do you have on a typical day when you are drinking? | 1. Never, monthly or less, 2-4 times a month, 2-3 times a week, 4+ times a week 2. 1-2, 3-4, 5-6, 7-9, 10+ | 1. Recoded to 0, 0.25, 0.75, 2.5, 4 to represent number of days consumed alcohol per week 2. Recoded as 1.5, 3.5, 5.5, 8, 10 3. Multiply (a) and (b) to obtained measure of weekly units per week |
| **17 years (C)** |  |  |  |
| **18 years (Q)** |  |  |  |
| **21 years (Q)** |  |  |  |

All offspring’s time points represent the age at which the questionnaire/clinic was administered

At all time points, participants were informed that a glass is the equivalent of a single measure (1oz) of spirit, ½ pint of beer or cider or a small glass (125ml) of wine.

(C) = data collected during a clinic session; (Q) data collected using a postal questionnaire
