# Supplementary material for: Exploration of a Polygenic Risk Score for Alcohol Consumption: A Longitudinal Analysis from the ALSPAC Cohort
Source: PLoS One. 2016 Nov 30;11(11):e0167360. doi: 10.1371/journal.pone.0167360 (PMC5130278; doi:10.1371/journal.pone.0167360)
Supplement: S3 Table — (DOCX) [file pone.0167360.s003.docx]

**S3 Table: SNP information**

GWAS hit = evidence for association between SNP and a non-alcohol related phenotype. Determined by searching of the NHGRI-EBI GWAS Catalog (1)

| **SNP** | **Gene** | **Chromosome** | **Position** | **Effect Allele** | **Effect allele frequency** | **Reference** | **Type of reference** | **Included in mother’s PGRS?** | **Included in offspring’s PGRS?** | **GWAS hit (for PGRS SNPs only)** |
| --- | --- | --- | --- | --- | --- | --- | --- | --- | --- | --- |
| rs3131513 | *Intergenic* | 1 | 24866664 | G | 0.393 | (2) | GWAS | ✓ |  | - |
| rs4478858 | SERINC2 | 1 | 31411078 | C | 0.430 | (3) | GWAS | ✓ |  | - |
| rs195204 | *Intergenic* | 1 | 115191470 | C | 0.245 | (4) | GWAS |  |  |  |
| rs6701037 | *Intergenic* | 1 | 175150943 | C | 0.465 | (5) | GWAS |  |  |  |
| rs7553212 | ESRRG | 1 | 216565446 | C | 0.332 | (6) | GWAS |  |  |  |
| rs3738443 | *Intergenic* | 1 | 247184887 | G | 0.169 | (7) | GWAS |  |  |  |
| rs2100290 | *Intergenic* | 2 | 79405221 | T | 0.499 | (2) | GWAS | ✓ |  | (a) |
| rs9636231 | *Intergenic* | 2 | 138914385 | A | 0.291 | (8) | GWAS |  |  |  |
| rs6716455 | *Intergenic* | 2 | 150269889 | A | 0.133 | (4) | GWAS |  |  |  |
| rs67031482 | PLCL1 | 2 | 198046442 | A | 0.488 | (6) | GWAS |  |  |  |
| rs1344694 | *Intergenic* | 2 | 216028914 | C | 0.335 | (9) | GWAS |  |  |  |
| rs7590720 | PECR | 2 | 216033935 | T | 0.292 | (10) | GWAS |  |  |  |
| rs12472151 | TRPM8 | 2 | 233944048 | G | 0.047 | (2) | GWAS |  |  |  |
| rs1318937 | SH3BP5-AS1 | 3 | 15253857 | A | 0.131 | (8) | GWAS | ✓ |  | (b) |
| rs9871864 | *Intergenic* | 3 | 20311274 | G | 0.459 | (10) | GWAS | ✓ |  | - |
| rs9825310 | *Intergenic* | 3 | 32198627 | A | 0.472 | (9) | GWAS |  |  |  |
| rs2369955 | *Intergenic* | 3 | 132974744 | C | 0.129 | (4) | GWAS |  |  |  |
| rs1353899 | LINC00578 | 3 | 177511191 | G | 0.198 | (6) | GWAS | ✓ |  | (a) |
| rs3930234 | *Intergenic* | 3 | 184498342 | T | 0.159 | (4) | GWAS |  |  |  |
| rs1000579 | *Intergenic* | 4 | 4717767 | G | 0.393 | (9) | GWAS |  |  |  |
| rs4543123 | *Intergenic* | 4 | 38790903 | G | 0.233 | (6) | GWAS |  |  |  |
| rs1497571 | GABRG1 | 4 | 46057233 | T | 0.0478 | (11) | Functional | ✓ |  | - |
| rs567926 | *Intergenic* | 4 | 46239752 | G | 0.437 | (12-17) | Functional |  | ✓ | - |
| rs279861 | GABRA2 | 4 | 46311308 | C | 0.445 | (12-14, 16-21) | Functional |  |  |  |
| rs1109501 | MUC7 | 4 | 70463773 | A | 0.256 | (4) | GWAS |  |  |  |
| rs1908556 | *Intergenic* | 4 | 89500549 | G | 0.138 | (19) | Functional |  |  |  |
| rs1230165 | *Intergenic* | 4 | 99065222 | G | 0.185 | (22) | Functional | ✓ |  | No |
| rs1800759 | ADH4 | 4 | 99144358 | T | 0.390 | (23-25) | Functional | ✓ |  | No |
| rs3762894 | ADH4 | 4 | 99144933 | C | 0.164 | (22, 24) | Functional |  |  |  |
| rs3819197 | ADH1A | 4 | 99279352 | T | 0.241 | (22, 24) | Functional |  |  |  |
| rs1042026 | ADH1B | 4 | 99307309 | C | 0.293 | (22, 24, 26, 27) | Functional | ✓ |  | (c) |
| rs1229984 | ADH1B | 4 | 99318162 | T | 0.032 | (6, 28-30) | GWAS/ Functional | ✓ |  | (d) |
| rs1353621 | ADH1B | 4 | 99320418 | C | 0.372 | (24, 27) | Functional |  |  |  |
| rs1789891 | *Intergenic* | 4 | 99329262 | A | 0.166 | (9) | GWAS | ✓ |  | (e) |
| rs284786 | ADH7 | 4 | 99412820 | A | 0.309 | (24, 26) | Functional |  |  |  |
| rs1573496 | ADH7 | 4 | 99428512 | G | 0.098 | (26, 27, 31) | Functional | ✓ |  | - |
| rs4440177 | *Intergenic* | 4 | 162321901 | A | 0.325 | (2) | GWAS |  |  |  |
| rs11724320 | NPY1R | 4 | 163346771 | T | 0.346 | (32) | Functional | ✓ |  | - |
| rs2548145 | *Intergenic* | 5 | 40134675 | A | 0.480 | (4) | GWAS |  |  |  |
| rs13160562 | ERAP1 | 5 | 96775667 | A | 0.311 | (10) | GWAS | ✓ |  | - |
| rs1864982 | PPP2R2B | 5 | 146941260 | T | 0.131 | (10) | GWAS |  |  |  |
| rs2140418 | ANKS1A | 6 | 35007638 | T | 0.191 | (4) | GWAS | ✓ |  | - |
| rs2380220 | *Intergenic* | 6 | 95519536 | T | 0.148 | (10) | GWAS |  |  |  |
| rs6902771 | ESR1 | 6 | 151836746 | C | 0.547 | (10) | GWAS | ✓ |  | - |
| rs9656709 | *Intergenic* | 7 | 55656752 | C | 0.489 | (10) | GWAS | ✓ |  | (a) |
| rs6943555 | AUTS2 | 7 | 70341037 | A | 0.252 | (33) | GWAS | ✓ |  | - |
| rs237238 | HIP1 | 7 | 75582098 | G | 0.069 | (4) | GWAS |  |  |  |
| rs2188561 | SLC26A4 | 7 | 107695613 | A | 0.222 | (6) | GWAS |  | ✓ | (a) |
| rs10253361 | *Intergenic* | 7 | 121635175 | C | 0.446 | (4) | GWAS |  |  |  |
| rs1824024 | CHRM2 | 7 | 136958947 | C | 0.324 | (34-36) | Functional | ✓ |  | - |
| rs420817 | CHRM2 | 7 | 137002656 | T | 0.494 | (35) | Functional |  |  |  |
| rs804292 | NEIL2 | 8 | 11786406 | G | 0.254 | (2) | GWAS |  |  |  |
| rs13259667 | *Intergenic* | 8 | 77413649 | A | 0.078 | (10) | GWAS | ✓ |  | - |
| rs36061340 | *Intergenic* | 8 | 100795002 | T | 0.062 | (6) | GWAS |  |  |  |
| rs59677118 | *Intergenic* | 9 | 14441679 | A | 0.092 | (6) | GWAS |  |  |  |
| rs2228093 | ALDH1B1 | 9 | 38396005 | T | .151 | (37) | Functional |  |  |  |
| rs3764435 | ALDH1A1 | 9 | 72901960 | A | 0.492 | (38) | Functional | ✓ |  | - |
| rs2303317 | ALDH1A1 | 9 | 72927026 | G | 0.482 | (38) | Functional | ✓ |  | - |
| rs10908907 | *Intergenic* | 9 | 89634669 | A | 0.247 | (4) | GWAS |  |  |  |
| rs4758317 | LMO1 | 11 | 8229264 | C | 0.448 | (6) | GWAS |  |  |  |
| rs750338 | PKNOX2 | 11 | 125302697 | G | 0.224 | (5) | GWAS | ✓ |  | - |
| rs10893366 | PKNOX2 | 11 | 125308507 | T | 0.167 | (8) | GWAS | ✓ |  | - |
| rs1793257 | OPCML | 11 | 132714224 | T | 0.036 | (2) | GWAS |  |  |  |
| rs12311304 | *Intergenic* | 12 | 15236703 | C | 0.322 | (10) | GWAS | ✓ |  | - |
| rs4761097 | *Intergenic* | 12 | 84793732 | G | 0.448 | (10) | GWAS |  |  |  |
| rs10849915 | CCDC63 | 12 | 110895818 | C | 0.345 | (39) | GWAS | ✓ |  | (a) |
| rs886205 | ALDH2 | 12 | 111766623 | G | 0.178 | (22) | Functional |  |  |  |
| rs4770403 | SGCG | 13 | 23180988 | A | 0.191 | (9) | GWAS | ✓ |  | - |
| rs9512637 | *Intergenic* | 13 | 27346474 | T | 0.361 | (4) | GWAS | ✓ |  | - |
| rs642899 | STARD13 | 13 | 33106717 | G | 0.230 | (10) | GWAS | ✓ |  | - |
| rs9556711 | MBNL2 | 13 | 97364162 | A | 0.061 | (4) | GWAS | ✓ |  | - |
| rs2154294 | *Intergenic* | 14 | 42186072 | A | 0.481 | (4) | GWAS |  | ✓ | - |
| rs1380131 | *Intergenic* | 14 | 53606140 | T | 0.092 | (4) | GWAS | ✓ |  | - |
| rs11851015 | EXOC5 | 14 | 57202815 | G | 0.134 | (6) | GWAS | ✓ | ✓ | (a) |
| rs7144649 | *Intergenic* | 14 | 57355498 | G | 0.224 | (6) | GWAS |  |  |  |
| rs36563 | *Intergenic* | 14 | 70885931 | T | 0.152 | (10) | GWAS | ✓ |  | - |
| rs2810114 | PCNX | 14 | 70928887 | C | 0.274 | (9) | GWAS | ✓ |  | - |
| rs8040009 | C15orf32 | 15 | 92501109 | C | 0.201 | (4) | GWAS | ✓ | ✓ | (f) |
| rs933769 | *Intergenic* | 15 | 95509513 | C | 0.182 | (4) | GWAS | ✓ |  | - |
| rs8062326 | *Intergenic* | 16 | 19144694 | A | 0.031 | (40) | GWAS |  |  |  |
| rs242938 | CRHR1 | 17 | 45818570 | A | 0.064 | (41, 42) | Functional | ✓ |  | - |
| rs1876831 | CRHR1 | 17 | 45830379 | T | 0.239 | (41, 42) | Functional | ✓ |  | - |
| rs768048 | DCC | 18 | 52759028 | T | 0.135 | (4) | GWAS |  |  |  |
| rs62202398 | *Intergenic* | 20 | 48172481 | A | 0.063 | (6) | GWAS | ✓ |  | (a) |
| rs59972978 | *Intergenic* | 20 | 57483299 | T | 0.185 | (6) | GWAS | ✓ |  | (a) |
| rs2827312 | *Intergenic* | 21 | 22259356 | T | 0.375 | (4) | GWAS |  |  |  |
| rs4293630 | *Intergenic* | 21 | 46025877 | G | 0.134 | (4) | GWAS | ✓ | ✓ | - |
| rs16985179 | *Intergenic* | 22 | 27633571 | T | 0.096 | (6, 22) | GWAS/ Functional |  |  |  |
| rs12388359 | *Intergenic* | X | 10263651 | T | 0.133 | (10, 22) | GWAS/ Functional |  |  |  |

(a) Diastolic blood pressure alcohol interaction; (b) Alcohol and nicotine co-dependence; (c) Oesophageal cancer (alcohol interaction); (d) Upper aerodigestive tract cancers oesophageal cancer; (e) Conduct disorder (interaction with high maternal expressed emotion)

(f) Bipolar disorder (Chinese population only)

**S3 Table references:**

1. Welter D, MacArthur J, Morales J, Burdett T, Hall P, Junkins H, et al. The NHGRI GWAS Catalog, a curated resource of SNP-trait associations. Nucleic acids research. 2014;42(D1):D1001-D6.

2. McGue M, Zhang Y, Miller MB, Basu S, Vrieze S, Hicks B, et al. A genome-wide association study of behavioral disinhibition. Behavior genetics. 2013;43(5):363-73.

3. Zuo L, Wang K, Zhang X-Y, Krystal JH, Li C-SR, Zhang F, et al. NKAIN1–SERINC2 is a functional, replicable and genome-wide significant risk gene region specific for alcohol dependence in subjects of European descent. Drug and alcohol dependence. 2013;129(3):254-64.

4. Heath AC, Whitfield JB, Martin NG, Pergadia ML, Goate AM, Lind PA, et al. A quantitative-trait genome-wide association study of alcoholism risk in the community: findings and implications. Biological psychiatry. 2011;70(6):513-8.

5. Wang K-S, Liu X, Zhang Q, Pan Y, Aragam N, Zeng M. A meta-analysis of two genome-wide association studies identifies 3 new loci for alcohol dependence. Journal of psychiatric research. 2011;45(11):1419-25.

6. Kapoor M, Wang J-C, Wetherill L, Le N, Bertelsen S, Hinrichs AL, et al. A meta-analysis of two genome-wide association studies to identify novel loci for maximum number of alcoholic drinks. Human genetics. 2013;132(10):1141-51.

7. Kendler KS, Kalsi G, Holmans PA, Sanders AR, Aggen SH, Dick DM, et al. Genomewide association analysis of symptoms of alcohol dependence in the molecular genetics of schizophrenia (MGS2) control sample. Alcoholism: Clinical and Experimental Research. 2011;35(5):963-75.

8. Zuo L, Gelernter J, Zhang CK, Zhao H, Lu L, Kranzler HR, et al. Genome-wide association study of alcohol dependence implicates KIAA0040 on chromosome 1q. Neuropsychopharmacology : official publication of the American College of Neuropsychopharmacology. 2012;37(2):557-66.

9. Frank J, Cichon S, Treutlein J, Ridinger M, Mattheisen M, Hoffmann P, et al. Genome‐wide significant association between alcohol dependence and a variant in the ADH gene cluster. Addiction biology. 2012;17(1):171-80.

10. Treutlein J, Cichon S, Ridinger M, Wodarz N, Soyka M, Zill P, et al. Genome-wide association study of alcohol dependence. Archives of general psychiatry. 2009;66(7):773-84.

11. Ray LA, Hutchison KE. Associations among GABRG1, level of response to alcohol, and drinking behaviors. Alcoholism: Clinical and Experimental Research. 2009;33(8):1382-90.

12. Fehr C, Sander T, Tadic A, Lenzen KP, Anghelescu I, Klawe C, et al. Confirmation of association of the GABRA2 gene with alcohol dependence by subtype-specific analysis. Psychiatric genetics. 2006;16(1):9-17.

13. Covault J, Gelernter J, Hesselbrock V, Nellissery M, Kranzler HR. Allelic and haplotypic association of GABRA2 with alcohol dependence. American Journal of Medical Genetics Part B: Neuropsychiatric Genetics. 2004;129(1):104-9.

14. Lappalainen J, Krupitsky E, Remizov M, Pchelina S, Taraskina A, Zvartau E, et al. Association Between Alcoholism and γ‐Amino Butyric Acid α2 Receptor Subtype in a Russian Population. Alcoholism: Clinical and Experimental Research. 2005;29(4):493-8.

15. Covault J, Gelernter J, Jensen K, Anton R, Kranzler HR. Markers in the 5′-region of GABRG1 associate to alcohol dependence and are in linkage disequilibrium with markers in the adjacent GABRA2 gene. Neuropsychopharmacology : official publication of the American College of Neuropsychopharmacology. 2008;33(4):837-48.

16. Soyka M, Preuss U, Hesselbrock V, Zill P, Koller G, Bondy B. GABA-A2 receptor subunit gene (GABRA2) polymorphisms and risk for alcohol dependence. Journal of psychiatric research. 2008;42(3):184-91.

17. Lind PA, MacGregor S, Montgomery GW, Heath AC, Martin NG, Whitfield JB. Effects of GABRA2 variation on physiological, psychomotor and subjective responses in the alcohol challenge twin study. Twin Research and Human Genetics. 2008;11(02):174-82.

18. Edenberg HJ, Dick DM, Xuei X, Tian H, Almasy L, Bauer LO, et al. Variations in GABRA2, encoding the α2 subunit of the GABA A receptor, are associated with alcohol dependence and with brain oscillations. The American Journal of Human Genetics. 2004;74(4):705-14.

19. Dick DM, Bierut L, Hinrichs A, Fox L, Bucholz KK, Kramer J, et al. The role of GABRA2 in risk for conduct disorder and alcohol and drug dependence across developmental stages. Behavior genetics. 2006;36(4):577-90.

20. Philibert RA, Gunter TD, Beach SR, Brody GH, Hollenbeck N, Andersen A, et al. The role of GABRA2 on risk for alcohol, nicotine and cannabis dependence in the Iowa adoption studies. Psychiatric genetics. 2009;19(2):91.

21. Haughey H, Ray L, Finan P, Villanueva R, Niculescu M, Hutchison K. Human γ‐aminobutyric acid A receptor α2 gene moderates the acute effects of alcohol and brain mRNA expression. Genes, Brain and Behavior. 2008;7(4):447-54.

22. Macgregor S, Lind PA, Bucholz KK, Hansell NK, Madden PA, Richter MM, et al. Associations of ADH and ALDH2 gene variation with self report alcohol reactions, consumption and dependence: an integrated analysis. Human molecular genetics. 2009;18(3):580-93.

23. Luo X, Kranzler HR, Zuo L, Lappalainen J, Yang B-z, Gelernter J. ADH4 gene variation is associated with alcohol dependence and drug dependence in European Americans: results from HWD tests and case–control association studies. Neuropsychopharmacology : official publication of the American College of Neuropsychopharmacology. 2006;31(5):1085-95.

24. Edenberg HJ, Xuei X, Chen H-J, Tian H, Wetherill LF, Dick DM, et al. Association of alcohol dehydrogenase genes with alcohol dependence: a comprehensive analysis. Human molecular genetics. 2006;15(9):1539-49.

25. Guindalini C, Scivoletto S, Ferreira RG, Breen G, Zilberman M, Peluso MA, et al. Association of genetic variants in alcohol dehydrogenase 4 with alcohol dependence in Brazilian patients. Am J Psychiat. 2005;162(5):1005-7.

26. Luo X, Kranzler HR, Zuo L, Wang S, Schork NJ, Gelernter J. Diplotype trend regression analysis of the ADH gene cluster and the ALDH2 gene: multiple significant associations with alcohol dependence. The American Journal of Human Genetics. 2006;78(6):973-87.

27. Kuo PH, Kalsi G, Prescott CA, Hodgkinson CA, Goldman D, Van Den Oord EJ, et al. Association of ADH and ALDH genes with alcohol dependence in the Irish Affected Sib Pair Study of alcohol dependence (IASPSAD) sample. Alcoholism: Clinical and Experimental Research. 2008;32(5):785-95.

28. Park BL, Kim JW, Cheong HS, Kim LH, Lee BC, Seo CH, et al. Extended genetic effects of ADH cluster genes on the risk of alcohol dependence: from GWAS to replication. Human genetics. 2013;132(6):657-68.

29. Hashibe M, Boffetta P, Zaridze D, Shangina O, Szeszenia-Dabrowska N, Mates D, et al. Evidence for an important role of alcohol-and aldehyde-metabolizing genes in cancers of the upper aerodigestive tract. Cancer Epidemiology Biomarkers & Prevention. 2006;15(4):696-703.

30. Wall TL, Shea SH, Luczak SE, Cook TA, Carr LG. Genetic associations of alcohol dehydrogenase with alcohol use disorders and endophenotypes in white college students. J Abnorm Psychol. 2005;114(3):456.

31. Hashibe M, McKay JD, Curado MP, Oliveira JC, Koifman S, Koifman R, et al. Multiple ADH genes are associated with upper aerodigestive cancers. Nature genetics. 2008;40(6):707-9.

32. Elbers CC, de Kovel CG, van der Schouw YT, Meijboom JR, Bauer F, Grobbee DE, et al. Variants in neuropeptide Y receptor 1 and 5 are associated with nutrient-specific food intake and are under recent selection in Europeans. PloS one. 2009;4(9):e7070.

33. Schumann G, Coin LJ, Lourdusamy A, Charoen P, Berger KH, Stacey D, et al. Genome-wide association and genetic functional studies identify autism susceptibility candidate 2 gene (AUTS2) in the regulation of alcohol consumption. Proceedings of the National Academy of Sciences. 2011;108(17):7119-24.

34. Wang JC, Hinrichs AL, Stock H, Budde J, Allen R, Bertelsen S, et al. Evidence of common and specific genetic effects: association of the muscarinic acetylcholine receptor M2 (CHRM2) gene with alcohol dependence and major depressive syndrome. Human molecular genetics. 2004;13(17):1903-11.

35. Luo X, Kranzler HR, Zuo L, Wang S, Blumberg HP, Gelernter J. CHRM2 gene predisposes to alcohol dependence, drug dependence and affective disorders: results from an extended case–control structured association study. Human Molecular Genetics. 2005;14(16):2421-34.

36. Luo X, Kranzler HR, Zuo L, Zhang H, Wang S, Gelernter J. CHRM2 variation predisposes to personality traits of agreeableness and conscientiousness. Human Molecular Genetics. 2007;16(13):1557-68.

37. Husemoen LLN, Fenger M, Friedrich N, Tolstrup JS, Beenfeldt Fredriksen S, Linneberg A. The association of ADH and ALDH gene variants with alcohol drinking habits and cardiovascular disease risk factors. Alcoholism: Clinical and Experimental Research. 2008;32(11):1984-91.

38. Liu J, Zhou Z, Hodgkinson CA, Yuan Q, Shen PH, Mulligan CJ, et al. Haplotype‐Based Study of the Association of Alcohol‐Metabolizing Genes With Alcohol Dependence in Four Independent Populations. Alcoholism: Clinical and Experimental Research. 2011;35(2):304-16.

39. Baik I, Cho NH, Kim SH, Han B-G, Shin C. Genome-wide association studies identify genetic loci related to alcohol consumption in Korean men. The American journal of clinical nutrition. 2011;93(4):809-16.

40. Lydall GJ, Bass NJ, McQuillin A, Lawrence J, Anjorin A, Kandaswamy R, et al. Confirmation of prior evidence of genetic susceptibility to alcoholism in a genome wide association study of comorbid alcoholism and bipolar disorder. Psychiatric genetics. 2011;21(6):294.

41. Treutlein J, Kissling C, Frank J, Wiemann S, Dong L, Depner M, et al. Genetic association of the human corticotropin releasing hormone receptor 1 (CRHR1) with binge drinking and alcohol intake patterns in two independent samples. Molecular psychiatry. 2006;11(6):594-602.

42. Blomeyer D, Treutlein J, Esser G, Schmidt MH, Schumann G, Laucht M. Interaction between CRHR1 gene and stressful life events predicts adolescent heavy alcohol use. Biological psychiatry. 2008;63(2):146-51.
