# Supplementary material for: Exploration of a Polygenic Risk Score for Alcohol Consumption: A Longitudinal Analysis from the ALSPAC Cohort
Source: PLoS One. 2016 Nov 30;11(11):e0167360. doi: 10.1371/journal.pone.0167360 (PMC5130278; doi:10.1371/journal.pone.0167360)
Supplement: S4 Table — (DOCX) [file pone.0167360.s004.docx]

**S4 Table: All results for repeated measures alcohol consumption in ALSPAC mothers and offspring**

| **ALSPAC Mothers** | | | | | | | **ALSPAC offspring** | | | | | | |
| --- | --- | --- | --- | --- | --- | --- | --- | --- | --- | --- | --- | --- | --- |
| **Rank** | **SNP** | **Effect size** | **SE** | ***t*** | **p-value** | **Rank in offspring** | **Rank** | **SNP** | **Effect size** | **SE** | ***t*** | **p-value** | **Rank in mothers** |
| **1** | **rs1229984** | -0.177 | 0.042 | -4.223 | 2.40E-05 | 22 | **1** | **rs2188561** | -0.055 | 0.019 | -2.855 | 0.0043 | 51 |
| **2** | **rs1497571** | 0.024 | 0.011 | 2.248 | 0.0246 | 36 | **2** | **rs8040009** | 0.056 | 0.021 | 2.711 | 0.0067 | 44 |
| **3** | **rs4478858** | -0.024 | 0.011 | -2.221 | 0.0264 | 84 | **3** | **rs567926** | -0.081 | 0.032 | -2.548 | 0.0108 | 70 |
| **4** | **rs6902771** | 0.023 | 0.011 | 2.154 | 0.0312 | 34 | **4** | **rs2154294** | -0.038 | 0.016 | -2.339 | 0.0193 | 77 |
| **5** | **rs11724320** | -0.023 | 0.011 | -2.007 | 0.0448 | 67 | **5** | **rs11851015** | 0.049 | 0.023 | 2.099 | 0.0358 | 25 |
| **6** | **rs1876831** | -0.025 | 0.013 | -1.962 | 0.0497 | 83 | **6** | **rs4293630** | -0.046 | 0.023 | -1.984 | 0.0473 | 33 |
| **7** | **rs1318937** | 0.031 | 0.016 | 1.944 | 0.0520 | 23 | **7** | **rs279861** | -0.059 | 0.032 | -1.834 | 0.0667 | 89 |
| **8** | **rs1380131** | 0.034 | 0.019 | 1.829 | 0.0674 | 33 | **8** | **rs2548145** | -0.029 | 0.016 | -1.807 | 0.0708 | 79 |
| **9** | **rs2100290** | -0.019 | 0.011 | -1.775 | 0.0759 | 24 | **9** | **rs1353621** | 0.028 | 0.017 | 1.693 | 0.0904 | 45 |
| **10** | **rs1230165** | 0.024 | 0.014 | 1.710 | 0.0872 | 27 | **10** | **rs3819197** | -0.031 | 0.019 | -1.668 | 0.0954 | 76 |
| **11** | **rs1353899** | -0.023 | 0.014 | -1.704 | 0.0884 | 53 | **11** | **rs4758317** | -0.027 | 0.016 | -1.644 | 0.1003 | 56 |
| **12** | **rs750338** | 0.022 | 0.013 | 1.701 | 0.0889 | 68 | **12** | **rs1573496** | 0.044 | 0.027 | 1.631 | 0.1029 | 20 |
| **13** | **rs3131513** | 0.018 | 0.011 | 1.648 | 0.0993 | 54 | **13** | **rs7590720** | -0.029 | 0.018 | -1.613 | 0.1068 | 63 |
| **14** | **rs9656709** | 0.017 | 0.011 | 1.601 | 0.1093 | 77 | **14** | **rs9512637** | 0.027 | 0.017 | 1.606 | 0.1082 | 30 |
| **15** | **rs4770403** | -0.022 | 0.014 | -1.577 | 0.1148 | 89 | **15** | **rs1908556** | 0.036 | 0.023 | 1.565 | 0.1176 | 49 |
| **16** | **rs642899** | 0.020 | 0.013 | 1.564 | 0.1177 | 47 | **16** | **rs12311304** | -0.026 | 0.017 | -1.538 | 0.1241 | 27 |
| **17** | **rs933769** | -0.021 | 0.014 | -1.496 | 0.1346 | 55 | **17** | **rs237238** | 0.048 | 0.031 | 1.514 | 0.1299 | 80 |
| **18** | **rs3764435** | 0.015 | 0.011 | 1.391 | 0.1644 | 64 | **18** | **rs62202398** | -0.048 | 0.034 | -1.423 | 0.1547 | 34 |
| **19** | **rs59972978** | 0.020 | 0.014 | 1.385 | 0.1661 | 29 | **19** | **rs1800759** | 0.023 | 0.016 | 1.418 | 0.1561 | 26 |
| **20** | **rs1573496** | -0.025 | 0.018 | -1.379 | 0.1680 | 12 | **20** | **rs1864982** | 0.033 | 0.023 | 1.409 | 0.1588 | 57 |
| **21** | **rs9556711** | 0.031 | 0.023 | 1.354 | 0.1756 | 78 | **21** | **rs1229984** | 0.068 | 0.051 | 1.328 | 0.1842 | 1 |
| **22** | **rs1789891** | 0.019 | 0.015 | 1.291 | 0.1969 | 62 | **22** | **rs1318937** | 0.031 | 0.024 | 1.312 | 0.1895 | 7 |
| **23** | **rs6943555** | -0.016 | 0.012 | -1.283 | 0.1997 | 48 | **23** | **rs2100290** | 0.020 | 0.016 | 1.272 | 0.2033 | 9 |
| **24** | **rs11851015** | -0.020 | 0.016 | -1.229 | 0.2190 | 18 | **24** | **rs2228093** | -0.031 | 0.024 | -1.271 | 0.2037 | 58 |
| **25** | **rs1800759** | 0.013 | 0.011 | 1.199 | 0.2305 | 5 | **25** | **rs242938** | 0.040 | 0.032 | 1.246 | 0.2128 | 29 |
| **26** | **rs12311304** | -0.014 | 0.012 | -1.174 | 0.2404 | 20 | **26** | **rs1230165** | 0.024 | 0.020 | 1.157 | 0.2471 | 10 |
| **27** | **rs13160562** | -0.013 | 0.011 | -1.145 | 0.2521 | 16 | **27** | **rs7144649** | 0.021 | 0.019 | 1.113 | 0.2659 | 53 |
| **28** | **rs242938** | -0.025 | 0.022 | -1.127 | 0.2597 | 70 | **28** | **rs59972978** | 0.024 | 0.021 | 1.107 | 0.2681 | 19 |
| **29** | **rs9512637** | -0.012 | 0.011 | -1.111 | 0.2667 | 26 | **29** | **rs13259667** | -0.033 | 0.030 | -1.104 | 0.2695 | 35 |
| **30** | **rs2140418** | -0.014 | 0.014 | -1.019 | 0.3084 | 14 | **30** | **rs768048** | 0.025 | 0.024 | 1.058 | 0.2898 | 81 |
| **31** | **rs2303317** | 0.011 | 0.011 | 1.001 | 0.3169 | 32 | **31** | **rs2140418** | -0.021 | 0.021 | -1.040 | 0.2981 | 31 |
| **32** | **rs4293630** | -0.016 | 0.016 | -0.978 | 0.3280 | 39 | **32** | **rs1380131** | 0.029 | 0.028 | 1.039 | 0.2990 | 8 |
| **33** | **rs62202398** | -0.021 | 0.022 | -0.950 | 0.3420 | 6 | **33** | **rs6902771** | -0.016 | 0.016 | -1.016 | 0.3097 | 4 |
| **34** | **rs13259667** | -0.019 | 0.020 | -0.940 | 0.3472 | 19 | **34** | **rs195204** | 0.019 | 0.018 | 1.003 | 0.3159 | 48 |
| **35** | **rs10893366** | 0.013 | 0.015 | 0.920 | 0.3578 | 30 | **35** | **rs1497571** | -0.017 | 0.018 | -0.925 | 0.3548 | 2 |
| **36** | **rs1824024** | 0.011 | 0.012 | 0.902 | 0.3671 | 56 | **36** | **rs3762894** | -0.019 | 0.021 | -0.897 | 0.3695 | 68 |
| **37** | **rs1042026** | -0.010 | 0.012 | -0.834 | 0.4044 | 45 | **37** | **rs10253361** | -0.014 | 0.016 | -0.889 | 0.3743 | 60 |
| **38** | **rs10849915** | 0.009 | 0.011 | 0.807 | 0.4196 | 40 | **38** | **rs2303317** | 0.014 | 0.016 | 0.882 | 0.3777 | 32 |
| **39** | **rs2810114** | -0.009 | 0.012 | -0.772 | 0.4404 | 82 | **39** | **rs1042026** | -0.015 | 0.018 | -0.876 | 0.3813 | 38 |
| **40** | **rs36563** | -0.011 | 0.015 | -0.760 | 0.4471 | 57 | **40** | **rs886205** | -0.018 | 0.021 | -0.862 | 0.3887 | 64 |
| **41** | **rs9871864** | 0.008 | 0.011 | 0.714 | 0.4750 | 72 | **41** | **rs9871864** | -0.013 | 0.016 | -0.841 | 0.4002 | 42 |
| **42** | **rs4543123** | -0.009 | 0.013 | -0.701 | 0.4835 | 42 | **42** | **rs4761097** | -0.013 | 0.016 | -0.835 | 0.4037 | 71 |
| **43** | **rs8040009** | -0.010 | 0.014 | -0.699 | 0.4844 | 73 | **43** | **rs2369955** | -0.020 | 0.025 | -0.832 | 0.4055 | 90 |
| **44** | **rs1353621** | 0.007 | 0.011 | 0.670 | 0.5027 | 2 | **44** | **rs1824024** | -0.013 | 0.017 | -0.786 | 0.4316 | 37 |
| **45** | **rs9825310** | 0.007 | 0.011 | 0.644 | 0.5194 | 9 | **45** | **rs36061340** | -0.025 | 0.033 | -0.757 | 0.4489 | 59 |
| **46** | **rs10908907** | -0.008 | 0.012 | -0.641 | 0.5212 | 80 | **46** | **rs642899** | 0.014 | 0.019 | 0.722 | 0.4705 | 16 |
| **47** | **rs195204** | 0.008 | 0.012 | 0.628 | 0.5301 | 61 | **47** | **rs6943555** | 0.013 | 0.019 | 0.705 | 0.4809 | 23 |
| **48** | **rs1908556** | -0.009 | 0.016 | -0.591 | 0.5542 | 35 | **48** | **rs1344694** | -0.012 | 0.017 | -0.695 | 0.4868 | 55 |
| **49** | **rs12388359** | 0.009 | 0.016 | 0.576 | 0.5645 | 15 | **49** | **rs1109501** | -0.012 | 0.018 | -0.675 | 0.4995 | 84 |
| **50** | **rs2188561** | -0.006 | 0.013 | -0.483 | 0.6292 | 71 | **50** | **rs2380220** | 0.015 | 0.022 | 0.668 | 0.5039 | 75 |
| **51** | **rs7553212** | 0.005 | 0.011 | 0.479 | 0.6317 | 1 | **51** | **rs6716455** | 0.015 | 0.024 | 0.628 | 0.5301 | 88 |
| **52** | **rs7144649** | 0.006 | 0.013 | 0.473 | 0.6361 | 75 | **52** | **rs1353899** | -0.012 | 0.020 | -0.604 | 0.5460 | 11 |
| **53** | **rs284786** | 0.005 | 0.012 | 0.463 | 0.6434 | 28 | **53** | **rs3131513** | -0.010 | 0.016 | -0.587 | 0.5570 | 13 |
| **54** | **rs1344694** | -0.005 | 0.012 | -0.452 | 0.6513 | 85 | **54** | **rs933769** | -0.012 | 0.021 | -0.566 | 0.5713 | 17 |
| **55** | **rs4758317** | -0.005 | 0.011 | -0.448 | 0.6545 | 49 | **55** | **rs10893366** | -0.011 | 0.021 | -0.515 | 0.6065 | 36 |
| **56** | **rs1864982** | -0.007 | 0.016 | -0.426 | 0.6700 | 11 | **56** | **rs2810114** | 0.009 | 0.018 | 0.513 | 0.6081 | 40 |
| **57** | **rs2228093** | -0.007 | 0.016 | -0.415 | 0.6780 | 21 | **57** | **rs59677118** | -0.014 | 0.028 | -0.484 | 0.6282 | 66 |
| **58** | **rs36061340** | 0.009 | 0.023 | 0.390 | 0.6968 | 25 | **58** | **rs9636231** | 0.008 | 0.018 | 0.459 | 0.6461 | 78 |
| **59** | **rs10253361** | 0.004 | 0.011 | 0.373 | 0.7094 | 46 | **59** | **rs67031482** | -0.007 | 0.016 | -0.439 | 0.6605 | 67 |
| **60** | **rs6701037** | 0.004 | 0.011 | 0.372 | 0.7101 | 38 | **60** | **rs10908907** | 0.008 | 0.018 | 0.416 | 0.6772 | 47 |
| **61** | **rs8062326** | -0.011 | 0.031 | -0.356 | 0.7220 | 63 | **61** | **rs1789891** | -0.008 | 0.022 | -0.368 | 0.7132 | 22 |
| **62** | **rs7590720** | -0.004 | 0.012 | -0.338 | 0.7353 | 76 | **62** | **rs6701037** | -0.006 | 0.016 | -0.359 | 0.7198 | 61 |
| **63** | **rs886205** | -0.005 | 0.014 | -0.332 | 0.7396 | 13 | **63** | **rs3764435** | -0.006 | 0.016 | -0.353 | 0.7240 | 18 |
| **64** | **rs804292** | 0.004 | 0.013 | 0.329 | 0.7418 | 41 | **64** | **rs2827312** | -0.006 | 0.018 | -0.351 | 0.7259 | 73 |
| **65** | **rs59677118** | 0.006 | 0.019 | 0.301 | 0.7636 | 86 | **65** | **rs420817** | 0.005 | 0.016 | 0.321 | 0.7486 | 86 |
| **66** | **rs67031482** | 0.003 | 0.011 | 0.287 | 0.7744 | 58 | **66** | **rs11724320** | -0.005 | 0.017 | -0.311 | 0.7562 | 5 |
| **67** | **rs3762894** | -0.004 | 0.015 | -0.283 | 0.7769 | 60 | **67** | **rs750338** | -0.006 | 0.019 | -0.306 | 0.7594 | 12 |
| **68** | **rs1793257** | -0.008 | 0.029 | -0.282 | 0.7777 | 37 | **68** | **rs12472151** | 0.012 | 0.038 | 0.304 | 0.7609 | 82 |
| **69** | **rs567926** | 0.003 | 0.011 | 0.266 | 0.7901 | 87 | **69** | **rs13160562** | 0.005 | 0.017 | 0.303 | 0.7615 | 28 |
| **70** | **rs4761097** | 0.003 | 0.011 | 0.263 | 0.7924 | 3 | **70** | **rs12388359** | 0.005 | 0.019 | 0.281 | 0.7789 | 50 |
| **71** | **rs4440177** | -0.003 | 0.011 | -0.250 | 0.8023 | 43 | **71** | **rs36563** | -0.006 | 0.022 | -0.278 | 0.7806 | 41 |
| **72** | **rs2827312** | 0.003 | 0.012 | 0.248 | 0.8043 | 90 | **72** | **rs4543123** | -0.005 | 0.019 | -0.277 | 0.7815 | 43 |
| **73** | **rs3930234** | 0.004 | 0.015 | 0.242 | 0.8091 | 65 | **73** | **rs16985179** | 0.007 | 0.027 | 0.265 | 0.7913 | 83 |
| **74** | **rs2380220** | -0.004 | 0.015 | -0.240 | 0.8105 | 79 | **74** | **rs7553212** | 0.004 | 0.017 | 0.264 | 0.7915 | 52 |
| **75** | **rs3819197** | -0.003 | 0.013 | -0.233 | 0.8155 | 51 | **75** | **rs8062326** | -0.012 | 0.047 | -0.259 | 0.7954 | 62 |
| **76** | **rs2154294** | 0.002 | 0.011 | 0.231 | 0.8173 | 10 | **76** | **rs9656709** | 0.004 | 0.016 | 0.249 | 0.8037 | 14 |
| **77** | **rs9636231** | -0.003 | 0.012 | -0.227 | 0.8206 | 4 | **77** | **rs9556711** | -0.007 | 0.033 | -0.203 | 0.8391 | 21 |
| **78** | **rs2548145** | -0.002 | 0.011 | -0.188 | 0.8512 | 59 | **78** | **rs3930234** | -0.004 | 0.022 | -0.194 | 0.8463 | 74 |
| **79** | **rs237238** | -0.003 | 0.021 | -0.154 | 0.8776 | 8 | **79** | **rs9825310** | -0.003 | 0.016 | -0.170 | 0.8649 | 46 |
| **80** | **rs768048** | 0.002 | 0.016 | 0.139 | 0.8891 | 17 | **80** | **rs3738443** | -0.003 | 0.021 | -0.154 | 0.8775 | 85 |
| **81** | **rs12472151** | -0.003 | 0.026 | -0.131 | 0.8956 | 31 | **81** | **rs10849915** | 0.002 | 0.017 | 0.132 | 0.8947 | 39 |
| **82** | **rs16985179** | -0.002 | 0.019 | -0.118 | 0.9058 | 69 | **82** | **rs1876831** | 0.004 | 0.032 | 0.113 | 0.9102 | 6 |
| **83** | **rs1109501** | -0.001 | 0.012 | -0.098 | 0.9221 | 74 | **83** | **rs4478858** | 0.002 | 0.016 | 0.106 | 0.9153 | 3 |
| **84** | **rs3738443** | 0.001 | 0.014 | 0.081 | 0.9352 | 50 | **84** | **rs284786** | 0.002 | 0.017 | 0.102 | 0.9191 | 54 |
| **85** | **rs420817** | 0.001 | 0.011 | 0.081 | 0.9356 | 81 | **85** | **rs804292** | -0.001 | 0.018 | -0.082 | 0.9348 | 65 |
| **86** | **rs1000579** | 0.000 | 0.011 | 0.041 | 0.9674 | 66 | **86** | **rs1793257** | -0.003 | 0.042 | -0.075 | 0.9405 | 69 |
| **87** | **rs6716455** | -0.001 | 0.016 | -0.033 | 0.9736 | 88 | **87** | **rs1000579** | 0.000 | 0.016 | -0.025 | 0.9802 | 87 |
| **88** | **rs279861** | 0.000 | 0.011 | -0.008 | 0.9934 | 52 | **88** | **rs4770403** | 0.000 | 0.020 | 0.015 | 0.9878 | 15 |
| **89** | **rs2369955** | 0.000 | 0.016 | -0.006 | 0.9952 | 7 | **89** | **rs4440177** | 0.000 | 0.017 | 0.011 | 0.9916 | 72 |
