# Supplementary material for: Exploration of a Polygenic Risk Score for Alcohol Consumption: A Longitudinal Analysis from the ALSPAC Cohort
Source: PLoS One. 2016 Nov 30;11(11):e0167360. doi: 10.1371/journal.pone.0167360 (PMC5130278; doi:10.1371/journal.pone.0167360)
Supplement: S5 Table — (DOCX) [file pone.0167360.s005.docx]

**S5 Table: All results for cross sectional alcohol consumption, mothers (time points M0 to M18) and offspring (times points C15 to C21)**

| **Time Point** | **SNP** | **Effect size** | **SE** | **t** | **p-value** |
| --- | --- | --- | --- | --- | --- |
| C15 | **rs2303317** | 0.0238153 | 0.020225 | 1.17753 | 0.238984 |
| C15 | **rs933769** | -0.0186973 | 0.026015 | -0.71872 | 0.472314 |
| C15 | **rs4758317** | -0.0303247 | 0.020534 | -1.47681 | 0.139727 |
| C15 | **rs10893366** | 0.0423635 | 0.026828 | 1.579062 | 0.114322 |
| C15 | **rs11851015** | 0.0597821 | 0.029768 | 2.00825 | 0.044617 |
| C15 | **rs1824024** | -0.00386 | 0.021546 | -0.17916 | 0.857816 |
| C15 | **rs3819197** | -0.0525767 | 0.023645 | -2.22356 | 0.026178 |
| C15 | **rs3764435** | -0.0110181 | 0.020066 | -0.5491 | 0.582936 |
| C15 | **rs12311304** | -0.0280597 | 0.02177 | -1.2889 | 0.197432 |
| C15 | **rs1353621** | 0.0436859 | 0.021042 | 2.076113 | 0.037884 |
| C15 | **rs10849915** | 0.0080353 | 0.021627 | 0.371546 | 0.710231 |
| C15 | **rs6902771** | -0.0322674 | 0.02028 | -1.59108 | 0.111591 |
| C15 | **rs4543123** | -0.0235909 | 0.02405 | -0.98092 | 0.326633 |
| C15 | **rs2140418** | -0.0462955 | 0.026309 | -1.75971 | 0.078458 |
| C15 | **rs9871864** | -0.0360975 | 0.020217 | -1.78549 | 0.074182 |
| C15 | **rs3930234** | -0.011939 | 0.028142 | -0.42424 | 0.671391 |
| C15 | **rs1497571** | -0.0155359 | 0.023134 | -0.67156 | 0.501865 |
| C15 | **rs9636231** | 0.0166471 | 0.022448 | 0.741589 | 0.458336 |
| C15 | **rs3762894** | -0.0243461 | 0.026687 | -0.91227 | 0.361626 |
| C15 | **rs1864982** | 0.0193361 | 0.029528 | 0.654833 | 0.512576 |
| C15 | **rs242938** | 0.0919543 | 0.041165 | 2.233801 | 0.025496 |
| C15 | **rs567926** | -0.0723333 | 0.040653 | -1.77929 | 0.075193 |
| C15 | **rs6701037** | -0.0322503 | 0.020426 | -1.57886 | 0.114367 |
| C15 | **rs1229984** | -0.0485061 | 0.068193 | -0.71131 | 0.476894 |
| C15 | **rs2188561** | -0.04416 | 0.02416 | -1.82781 | 0.067578 |
| C15 | **rs1908556** | 0.0244805 | 0.029212 | 0.838021 | 0.402019 |
| C15 | **rs1109501** | -0.037188 | 0.02325 | -1.59946 | 0.109718 |
| C15 | **rs3131513** | 0.0038727 | 0.020716 | 0.186938 | 0.851709 |
| C15 | **rs67031482** | 0.0000215 | 0.020272 | 0.001058 | 0.999156 |
| C15 | **rs59972978** | 0.0306573 | 0.027061 | 1.132911 | 0.257252 |
| C15 | **rs59677118** | 0.0453897 | 0.03614 | 1.255942 | 0.209137 |
| C15 | **rs2154294** | -0.0239378 | 0.020447 | -1.17074 | 0.241705 |
| C15 | **rs12388359** | 0.0198992 | 0.024069 | 0.82676 | 0.408373 |
| C15 | **rs13259667** | -0.0593031 | 0.038386 | -1.54493 | 0.122364 |
| C15 | **rs2810114** | 0.0192175 | 0.022657 | 0.848187 | 0.396334 |
| C15 | **rs4770403** | -0.0088536 | 0.026027 | -0.34017 | 0.73373 |
| C15 | **rs750338** | 0.0245018 | 0.024205 | 1.012263 | 0.311412 |
| C15 | **rs6943555** | 0.011706 | 0.023644 | 0.495084 | 0.620541 |
| C15 | **rs6716455** | -0.0156874 | 0.030952 | -0.50684 | 0.612269 |
| C15 | **rs1230165** | 0.0144351 | 0.025868 | 0.558028 | 0.576825 |
| C15 | **rs9825310** | -0.0182286 | 0.020237 | -0.90077 | 0.367713 |
| C15 | **rs195204** | 0.0500582 | 0.023307 | 2.147751 | 0.031734 |
| C15 | **rs12472151** | 0.0265874 | 0.048303 | 0.550432 | 0.582023 |
| C15 | **rs4293630** | -0.0471608 | 0.029621 | -1.59213 | 0.111356 |
| C15 | **rs2827312** | -0.0240297 | 0.022448 | -1.07046 | 0.284414 |
| C15 | **rs7590720** | 0.0124585 | 0.022407 | 0.556016 | 0.5782 |
| C15 | **rs279861** | -0.0206265 | 0.041455 | -0.49756 | 0.618796 |
| C15 | **rs62202398** | 0.0280663 | 0.043192 | 0.649807 | 0.515817 |
| C15 | **rs2228093** | -0.0146534 | 0.031015 | -0.47246 | 0.636602 |
| C15 | **rs7144649** | 0.0601754 | 0.024304 | 2.475907 | 0.01329 |
| C15 | **rs7553212** | -0.0180652 | 0.021288 | -0.84863 | 0.396087 |
| C15 | **rs1789891** | 0.0057948 | 0.027475 | 0.210909 | 0.832959 |
| C15 | **rs1344694** | 0.0357671 | 0.021741 | 1.645182 | 0.099932 |
| C15 | **rs4440177** | -0.0360776 | 0.021537 | -1.67511 | 0.093912 |
| C15 | **rs16985179** | -0.0052778 | 0.035036 | -0.15064 | 0.88026 |
| C15 | **rs1318937** | 0.0093536 | 0.030371 | 0.307975 | 0.758101 |
| C15 | **rs36061340** | -0.0399102 | 0.041952 | -0.95134 | 0.341432 |
| C15 | **rs284786** | -0.0123442 | 0.021985 | -0.56148 | 0.574471 |
| C15 | **rs420817** | 0.0320691 | 0.020249 | 1.583717 | 0.113258 |
| C15 | **rs9656709** | 0.0079396 | 0.020594 | 0.385533 | 0.699843 |
| C15 | **rs1353899** | -0.009246 | 0.025232 | -0.36645 | 0.714031 |
| C15 | **rs2380220** | -0.0059832 | 0.028633 | -0.20896 | 0.834479 |
| C15 | **rs8062326** | -0.0702467 | 0.059199 | -1.18662 | 0.235379 |
| C15 | **rs11724320** | -0.0442153 | 0.021586 | -2.04833 | 0.040528 |
| C15 | **rs3738443** | -0.0156997 | 0.027405 | -0.57288 | 0.566728 |
| C15 | **rs9556711** | -0.0246849 | 0.041952 | -0.58841 | 0.556258 |
| C15 | **rs9512637** | -0.0103996 | 0.021195 | -0.49066 | 0.623666 |
| C15 | **rs804292** | 0.0023708 | 0.022906 | 0.103499 | 0.917567 |
| C15 | **rs4761097** | -0.0049073 | 0.02033 | -0.24138 | 0.809259 |
| C15 | **rs886205** | 0.0037905 | 0.026543 | 0.142807 | 0.886442 |
| C15 | **rs237238** | 0.0405843 | 0.03978 | 1.020213 | 0.307627 |
| C15 | **rs768048** | 0.0374675 | 0.030251 | 1.238537 | 0.215517 |
| C15 | **rs1800759** | 0.0092394 | 0.020456 | 0.451671 | 0.651506 |
| C15 | **rs36563** | 0.0004027 | 0.028428 | 0.014166 | 0.988698 |
| C15 | **rs1793257** | 0.0233069 | 0.053003 | 0.439732 | 0.660132 |
| C15 | **rs2369955** | -0.0445902 | 0.031032 | -1.43693 | 0.150738 |
| C15 | **rs1000579** | 0.0197238 | 0.020839 | 0.946483 | 0.343902 |
| C15 | **rs2100290** | 0.0303085 | 0.020144 | 1.50459 | 0.132429 |
| C15 | **rs1573496** | 0.0027089 | 0.034078 | 0.07949 | 0.936643 |
| C15 | **rs10253361** | 0.0050759 | 0.020515 | 0.247421 | 0.804583 |
| C15 | **rs642899** | 0.0007213 | 0.024055 | 0.029984 | 0.97608 |
| C15 | **rs1876831** | -0.003632 | 0.039855 | -0.09113 | 0.92739 |
| C15 | **rs8040009** | 0.0452618 | 0.026503 | 1.707809 | 0.087672 |
| C15 | **rs1042026** | -0.0481974 | 0.022186 | -2.17245 | 0.029822 |
| C15 | **rs2548145** | -0.00561 | 0.020189 | -0.27787 | 0.781111 |
| C15 | **rs13160562** | 0.0114647 | 0.022294 | 0.514253 | 0.607075 |
| C15 | **rs1380131** | -0.0341655 | 0.035137 | -0.97236 | 0.33087 |
| C15 | **rs10908907** | 0.0005353 | 0.023247 | 0.023028 | 0.981628 |
| C15 | **rs4478858** | -0.0167957 | 0.020671 | -0.81251 | 0.416499 |
| C16 | **rs1000579** | 0.0178242 | 0.029825 | 0.59762 | 0.550094 |
| C16 | **rs2827312** | -0.0306043 | 0.031839 | -0.96122 | 0.336441 |
| C16 | **rs1344694** | -0.0049389 | 0.031382 | -0.15738 | 0.874943 |
| C16 | **rs2140418** | -0.0379904 | 0.037594 | -1.01055 | 0.31223 |
| C16 | **rs36563** | 0.0074224 | 0.040924 | 0.181372 | 0.856076 |
| C16 | **rs642899** | 0.036874 | 0.033879 | 1.088419 | 0.27641 |
| C16 | **rs768048** | 0.0015038 | 0.043383 | 0.034663 | 0.972348 |
| C16 | **rs4440177** | 0.0051524 | 0.030962 | 0.166408 | 0.867836 |
| C16 | **rs284786** | 0.0346 | 0.031619 | 1.094297 | 0.273825 |
| C16 | **rs36061340** | -0.0156453 | 0.059163 | -0.26444 | 0.791438 |
| C16 | **rs1353621** | -0.0094109 | 0.030012 | -0.31357 | 0.753844 |
| C16 | **rs567926** | -0.1285028 | 0.05692 | -2.25761 | 0.02397 |
| C16 | **rs2548145** | -0.056051 | 0.029136 | -1.92378 | 0.054382 |
| C16 | **rs7144649** | 0.0340103 | 0.034511 | 0.985505 | 0.324376 |
| C16 | **rs9656709** | 0.0195504 | 0.02921 | 0.669295 | 0.503307 |
| C16 | **rs2188561** | -0.081996 | 0.034772 | -2.35813 | 0.018367 |
| C16 | **rs6943555** | 0.0233044 | 0.033601 | 0.693567 | 0.487954 |
| C16 | **rs242938** | 0.0814036 | 0.05774 | 1.409827 | 0.158591 |
| C16 | **rs1109501** | -0.0658172 | 0.033592 | -1.95933 | 0.050075 |
| C16 | **rs237238** | 0.0423568 | 0.056553 | 0.748972 | 0.453874 |
| C16 | **rs4543123** | 0.0269998 | 0.034658 | 0.779036 | 0.435959 |
| C16 | **rs4478858** | 0.0363976 | 0.029329 | 1.241031 | 0.214594 |
| C16 | **rs10908907** | 0.0255065 | 0.033483 | 0.761766 | 0.4462 |
| C16 | **rs2100290** | 0.0410738 | 0.028856 | 1.423409 | 0.154618 |
| C16 | **rs3131513** | -0.0156669 | 0.029358 | -0.53365 | 0.593582 |
| C16 | **rs1497571** | 0.0172821 | 0.032504 | 0.531693 | 0.594939 |
| C16 | **rs1318937** | 0.0516444 | 0.043069 | 1.199108 | 0.230486 |
| C16 | **rs67031482** | -0.0142803 | 0.029104 | -0.49066 | 0.62367 |
| C16 | **rs1793257** | -0.035792 | 0.075298 | -0.47534 | 0.634548 |
| C16 | **rs13160562** | -0.0086399 | 0.031777 | -0.27189 | 0.785707 |
| C16 | **rs420817** | -0.0134053 | 0.028933 | -0.46332 | 0.643136 |
| C16 | **rs2303317** | 0.0420303 | 0.028964 | 1.45114 | 0.146741 |
| C16 | **rs4761097** | -0.0442305 | 0.028756 | -1.53812 | 0.12402 |
| C16 | **rs1908556** | 0.0180321 | 0.041074 | 0.439015 | 0.660651 |
| C16 | **rs3819197** | -0.0186908 | 0.034444 | -0.54264 | 0.587376 |
| C16 | **rs7590720** | -0.0370332 | 0.032289 | -1.14694 | 0.251409 |
| C16 | **rs12311304** | -0.0664738 | 0.03138 | -2.11838 | 0.034143 |
| C16 | **rs10849915** | -0.0204145 | 0.030699 | -0.66499 | 0.506059 |
| C16 | **rs11724320** | 0.0049156 | 0.030506 | 0.161137 | 0.871986 |
| C16 | **rs1864982** | 0.0622112 | 0.04209 | 1.478054 | 0.139393 |
| C16 | **rs804292** | 0.0128769 | 0.032447 | 0.396865 | 0.691467 |
| C16 | **rs8062326** | -0.0456998 | 0.084678 | -0.53969 | 0.589409 |
| C16 | **rs4293630** | -0.055029 | 0.042091 | -1.30738 | 0.191083 |
| C16 | **rs2380220** | 0.040916 | 0.040233 | 1.016979 | 0.309164 |
| C16 | **rs6902771** | -0.0638075 | 0.028685 | -2.22441 | 0.026121 |
| C16 | **rs16985179** | 0.0000778 | 0.049878 | 0.001559 | 0.998756 |
| C16 | **rs6716455** | 0.0399557 | 0.044034 | 0.907383 | 0.364204 |
| C16 | **rs933769** | -0.0327772 | 0.037275 | -0.87934 | 0.379215 |
| C16 | **rs2228093** | 0.0007614 | 0.043712 | 0.017419 | 0.986102 |
| C16 | **rs2154294** | -0.0582422 | 0.0294 | -1.98101 | 0.04759 |
| C16 | **rs11851015** | 0.0396227 | 0.042206 | 0.9388 | 0.347833 |
| C16 | **rs3762894** | -0.0235467 | 0.038362 | -0.6138 | 0.539348 |
| C16 | **rs1800759** | 0.0422159 | 0.029326 | 1.439534 | 0.149999 |
| C16 | **rs195204** | -0.0032745 | 0.033871 | -0.09668 | 0.922984 |
| C16 | **rs12388359** | 0.0346582 | 0.035328 | 0.981053 | 0.326567 |
| C16 | **rs1789891** | 0.0174444 | 0.039932 | 0.436856 | 0.662216 |
| C16 | **rs3930234** | -0.0258403 | 0.040275 | -0.6416 | 0.521134 |
| C16 | **rs4758317** | -0.0467458 | 0.029409 | -1.58951 | 0.111945 |
| C16 | **rs2810114** | 0.0374881 | 0.032514 | 1.152981 | 0.248918 |
| C16 | **rs3738443** | -0.0331259 | 0.038621 | -0.85771 | 0.391053 |
| C16 | **rs1573496** | 0.0497631 | 0.048117 | 1.0342 | 0.301043 |
| C16 | **rs1229984** | 0.1063491 | 0.094997 | 1.119506 | 0.262924 |
| C16 | **rs9871864** | -0.0088165 | 0.029131 | -0.30264 | 0.762161 |
| C16 | **rs12472151** | -0.0499951 | 0.069721 | -0.71708 | 0.473325 |
| C16 | **rs4770403** | -0.0211118 | 0.037159 | -0.56815 | 0.569934 |
| C16 | **rs7553212** | 0.0335925 | 0.030256 | 1.110277 | 0.26688 |
| C16 | **rs279861** | -0.1475546 | 0.058979 | -2.50181 | 0.012356 |
| C16 | **rs13259667** | -0.0625201 | 0.053389 | -1.17102 | 0.241589 |
| C16 | **rs62202398** | -0.0336224 | 0.061529 | -0.54645 | 0.584755 |
| C16 | **rs1042026** | -0.0102333 | 0.031956 | -0.32023 | 0.748796 |
| C16 | **rs1824024** | -0.0224628 | 0.031096 | -0.72238 | 0.470061 |
| C16 | **rs1380131** | 0.031705 | 0.050285 | 0.630505 | 0.528364 |
| C16 | **rs1230165** | 0.0241463 | 0.036866 | 0.654971 | 0.512487 |
| C16 | **rs9512637** | 0.0311816 | 0.030488 | 1.02274 | 0.306431 |
| C16 | **rs1876831** | 0.0112677 | 0.057079 | 0.197407 | 0.843509 |
| C16 | **rs9825310** | -0.0097132 | 0.029307 | -0.33143 | 0.740322 |
| C16 | **rs1353899** | 0.0198194 | 0.036308 | 0.545863 | 0.58516 |
| C16 | **rs59677118** | -0.0659634 | 0.051136 | -1.28997 | 0.19706 |
| C16 | **rs6701037** | 0.0163659 | 0.029161 | 0.561228 | 0.574642 |
| C16 | **rs3764435** | 0.0027862 | 0.028882 | 0.09647 | 0.923148 |
| C16 | **rs10893366** | -0.0533749 | 0.039298 | -1.35822 | 0.174394 |
| C16 | **rs8040009** | 0.0522595 | 0.037918 | 1.378212 | 0.168138 |
| C16 | **rs9556711** | -0.013995 | 0.059986 | -0.2333 | 0.815525 |
| C16 | **rs10253361** | -0.0320431 | 0.02897 | -1.10607 | 0.268695 |
| C16 | **rs9636231** | 0.0037884 | 0.032324 | 0.117203 | 0.906699 |
| C16 | **rs886205** | -0.0300512 | 0.037655 | -0.79807 | 0.424829 |
| C16 | **rs750338** | -0.0533258 | 0.03526 | -1.51238 | 0.130437 |
| C16 | **rs59972978** | 0.0348636 | 0.038682 | 0.901299 | 0.367429 |
| C16 | **rs2369955** | -0.0248523 | 0.044816 | -0.55454 | 0.579207 |
| C17 | **rs12388359** | -0.0037773 | 0.033211 | -0.11374 | 0.909447 |
| C17 | **rs1824024** | -0.0683734 | 0.028746 | -2.3785 | 0.017383 |
| C17 | **rs1864982** | 0.0602111 | 0.040049 | 1.50345 | 0.132723 |
| C17 | **rs11724320** | -0.0128946 | 0.028769 | -0.44822 | 0.653995 |
| C17 | **rs2827312** | -0.0121934 | 0.029852 | -0.40846 | 0.682938 |
| C17 | **rs1318937** | 0.053803 | 0.039784 | 1.352392 | 0.17625 |
| C17 | **rs1344694** | 0.0130498 | 0.029182 | 0.447187 | 0.65474 |
| C17 | **rs2154294** | -0.0271206 | 0.027173 | -0.99808 | 0.318239 |
| C17 | **rs2188561** | -0.0722253 | 0.032371 | -2.23116 | 0.025671 |
| C17 | **rs1229984** | 0.0829414 | 0.084521 | 0.981316 | 0.326437 |
| C17 | **rs59972978** | 0.0302323 | 0.036131 | 0.836741 | 0.402738 |
| C17 | **rs7553212** | -0.026371 | 0.028178 | -0.93587 | 0.349341 |
| C17 | **rs67031482** | -0.0327862 | 0.027215 | -1.2047 | 0.228319 |
| C17 | **rs8040009** | 0.0372837 | 0.035109 | 1.061952 | 0.288257 |
| C17 | **rs1573496** | 0.0662119 | 0.045669 | 1.449833 | 0.147105 |
| C17 | **rs13259667** | -0.0622937 | 0.050637 | -1.23021 | 0.218618 |
| C17 | **rs420817** | 0.0420173 | 0.027092 | 1.550924 | 0.12092 |
| C17 | **rs6701037** | 0.0263675 | 0.027251 | 0.967585 | 0.333252 |
| C17 | **rs10849915** | -0.0028003 | 0.029114 | -0.09619 | 0.923373 |
| C17 | **rs4293630** | -0.0901354 | 0.03946 | -2.28424 | 0.022357 |
| C17 | **rs36061340** | 0.0258016 | 0.058341 | 0.44226 | 0.658301 |
| C17 | **rs1789891** | -0.0403873 | 0.036847 | -1.09609 | 0.273039 |
| C17 | **rs3762894** | -0.0326156 | 0.035683 | -0.91403 | 0.3607 |
| C17 | **rs804292** | 0.0391836 | 0.030303 | 1.29305 | 0.195994 |
| C17 | **rs3764435** | 0.0008771 | 0.027058 | 0.032417 | 0.97414 |
| C17 | **rs6716455** | -0.05615 | 0.041161 | -1.36415 | 0.172519 |
| C17 | **rs13160562** | -0.0088237 | 0.029925 | -0.29486 | 0.7681 |
| C17 | **rs284786** | 0.0024501 | 0.029544 | 0.082929 | 0.933908 |
| C17 | **rs10253361** | -0.0364208 | 0.027303 | -1.33397 | 0.182213 |
| C17 | **rs1800759** | 0.0285596 | 0.027478 | 1.039377 | 0.298629 |
| C17 | **rs1908556** | 0.0577964 | 0.038873 | 1.486802 | 0.137067 |
| C17 | **rs11851015** | 0.0795678 | 0.040209 | 1.978853 | 0.047833 |
| C17 | **rs1380131** | 0.09918 | 0.047542 | 2.086154 | 0.036965 |
| C17 | **rs9512637** | 0.0635188 | 0.028415 | 2.235435 | 0.025389 |
| C17 | **rs10908907** | 0.0245525 | 0.031651 | 0.775717 | 0.437916 |
| C17 | **rs4770403** | -0.0053256 | 0.034422 | -0.15472 | 0.877045 |
| C17 | **rs2140418** | 0.0134927 | 0.035077 | 0.38466 | 0.70049 |
| C17 | **rs9871864** | 0.0244837 | 0.027294 | 0.897044 | 0.369695 |
| C17 | **rs4761097** | 0.0163003 | 0.027165 | 0.600046 | 0.548476 |
| C17 | **rs237238** | 0.0975832 | 0.052849 | 1.846468 | 0.064824 |
| C17 | **rs36563** | 0.0118971 | 0.038033 | 0.312812 | 0.754424 |
| C17 | **rs12472151** | -0.0312438 | 0.06559 | -0.47635 | 0.633827 |
| C17 | **rs6902771** | -0.0075229 | 0.026948 | -0.27917 | 0.780115 |
| C17 | **rs242938** | 0.0275458 | 0.05404 | 0.509732 | 0.610239 |
| C17 | **rs2369955** | 0.0065056 | 0.04182 | 0.155562 | 0.876378 |
| C17 | **rs1876831** | -0.1018931 | 0.053628 | -1.89999 | 0.057435 |
| C17 | **rs7590720** | 0.0064462 | 0.03007 | 0.214374 | 0.830256 |
| C17 | **rs62202398** | -0.0661843 | 0.057144 | -1.15821 | 0.246779 |
| C17 | **rs4758317** | -0.0227117 | 0.027588 | -0.82325 | 0.410363 |
| C17 | **rs10893366** | 0.0092802 | 0.035794 | 0.259264 | 0.795432 |
| C17 | **rs2100290** | 0.0352335 | 0.027251 | 1.292938 | 0.196033 |
| C17 | **rs3819197** | -0.0384539 | 0.03181 | -1.20885 | 0.226721 |
| C17 | **rs933769** | -0.0315203 | 0.035066 | -0.89888 | 0.368718 |
| C17 | **rs567926** | -0.0758301 | 0.054239 | -1.39809 | 0.162087 |
| C17 | **rs2228093** | -0.0224814 | 0.040966 | -0.54879 | 0.583153 |
| C17 | **rs2810114** | 0.0157532 | 0.030111 | 0.523179 | 0.60085 |
| C17 | **rs9556711** | -0.0100862 | 0.057237 | -0.17622 | 0.860121 |
| C17 | **rs3738443** | -0.0537586 | 0.035627 | -1.50894 | 0.131314 |
| C17 | **rs8062326** | 0.0570223 | 0.078215 | 0.729041 | 0.465976 |
| C17 | **rs642899** | 0.0048399 | 0.032319 | 0.149756 | 0.880957 |
| C17 | **rs1042026** | -0.0175199 | 0.029977 | -0.58446 | 0.558913 |
| C17 | **rs4478858** | 0.0495718 | 0.027779 | 1.784522 | 0.074339 |
| C17 | **rs1353621** | 0.0334342 | 0.028157 | 1.18744 | 0.235054 |
| C17 | **rs1109501** | 0.0182873 | 0.030992 | 0.590062 | 0.555149 |
| C17 | **rs2303317** | -0.0028042 | 0.02725 | -0.10291 | 0.918038 |
| C17 | **rs2548145** | -0.0615169 | 0.027166 | -2.26448 | 0.023544 |
| C17 | **rs2380220** | -0.0101963 | 0.038809 | -0.26273 | 0.792757 |
| C17 | **rs1497571** | -0.0640031 | 0.030765 | -2.0804 | 0.037489 |
| C17 | **rs7144649** | 0.0264605 | 0.032151 | 0.823014 | 0.4105 |
| C17 | **rs279861** | -0.0469187 | 0.055689 | -0.84251 | 0.399503 |
| C17 | **rs16985179** | -0.100035 | 0.046346 | -2.15843 | 0.030895 |
| C17 | **rs768048** | -0.0012866 | 0.040031 | -0.03214 | 0.974361 |
| C17 | **rs4543123** | 0.0104949 | 0.031952 | 0.328453 | 0.742569 |
| C17 | **rs750338** | -0.0136576 | 0.03222 | -0.42388 | 0.671653 |
| C17 | **rs1793257** | 0.0337959 | 0.074118 | 0.455975 | 0.648408 |
| C17 | **rs4440177** | 0.0238536 | 0.028532 | 0.836029 | 0.403139 |
| C17 | **rs3131513** | -0.005316 | 0.027525 | -0.19313 | 0.846854 |
| C17 | **rs886205** | -0.0352829 | 0.035716 | -0.98787 | 0.323217 |
| C17 | **rs12311304** | 0.0125395 | 0.029449 | 0.425806 | 0.670249 |
| C17 | **rs1000579** | -0.0129444 | 0.027844 | -0.46489 | 0.642011 |
| C17 | **rs9636231** | 0.0190827 | 0.030008 | 0.635917 | 0.524831 |
| C17 | **rs1353899** | -0.0278345 | 0.033841 | -0.82251 | 0.410789 |
| C17 | **rs9656709** | 0.012613 | 0.027506 | 0.458552 | 0.646556 |
| C17 | **rs195204** | 0.0118377 | 0.031063 | 0.381086 | 0.70314 |
| C17 | **rs9825310** | 0.0153184 | 0.027316 | 0.560782 | 0.574946 |
| C17 | **rs1230165** | 0.0061691 | 0.034945 | 0.176539 | 0.859871 |
| C17 | **rs59677118** | -0.0495705 | 0.048795 | -1.0159 | 0.309676 |
| C17 | **rs6943555** | 0.0239117 | 0.031483 | 0.75951 | 0.447547 |
| C17 | **rs3930234** | -0.0140081 | 0.037642 | -0.37214 | 0.709786 |
| C18 | **rs1344694** | -0.0270773 | 0.031893 | -0.84902 | 0.395872 |
| C18 | **rs4293630** | -0.0386521 | 0.042816 | -0.90275 | 0.366656 |
| C18 | **rs1229984** | -0.0131044 | 0.092791 | -0.14123 | 0.887692 |
| C18 | **rs3819197** | -0.0160506 | 0.034388 | -0.46676 | 0.640675 |
| C18 | **rs1042026** | 0.0099968 | 0.032155 | 0.31089 | 0.755884 |
| C18 | **rs9636231** | 0.0619826 | 0.033139 | 1.870381 | 0.061431 |
| C18 | **rs59677118** | -0.0461643 | 0.050951 | -0.90606 | 0.364906 |
| C18 | **rs16985179** | 0.0322747 | 0.050803 | 0.635288 | 0.525241 |
| C18 | **rs11851015** | 0.0522302 | 0.04315 | 1.210431 | 0.226114 |
| C18 | **rs2303317** | 0.0472143 | 0.029385 | 1.606734 | 0.108113 |
| C18 | **rs36563** | -0.0182715 | 0.041784 | -0.43728 | 0.661907 |
| C18 | **rs933769** | 0.00065 | 0.037443 | 0.017359 | 0.986151 |
| C18 | **rs10893366** | -0.0516831 | 0.039113 | -1.32139 | 0.186373 |
| C18 | **rs1380131** | 0.0225009 | 0.0513 | 0.438612 | 0.660943 |
| C18 | **rs567926** | -0.06578 | 0.057736 | -1.13932 | 0.25457 |
| C18 | **rs3738443** | -0.0235617 | 0.039108 | -0.60248 | 0.546852 |
| C18 | **rs2827312** | 0.0069105 | 0.032608 | 0.211925 | 0.832165 |
| C18 | **rs67031482** | -0.0309313 | 0.029364 | -1.05338 | 0.292167 |
| C18 | **rs4478858** | -0.0358403 | 0.029984 | -1.19531 | 0.231965 |
| C18 | **rs642899** | 0.0145674 | 0.034434 | 0.423053 | 0.672256 |
| C18 | **rs1353621** | 0.0692248 | 0.031143 | 2.222779 | 0.026231 |
| C18 | **rs1318937** | 0.0365027 | 0.04457 | 0.818994 | 0.41279 |
| C18 | **rs804292** | -0.0407475 | 0.033259 | -1.22517 | 0.220511 |
| C18 | **rs242938** | -0.0059703 | 0.058742 | -0.10164 | 0.919046 |
| C18 | **rs6902771** | 0.0251576 | 0.029454 | 0.854134 | 0.393031 |
| C18 | **rs886205** | -0.0089239 | 0.039099 | -0.22824 | 0.81946 |
| C18 | **rs2369955** | 0.0160847 | 0.045677 | 0.352142 | 0.724732 |
| C18 | **rs420817** | -0.0344662 | 0.029282 | -1.17706 | 0.239173 |
| C18 | **rs2100290** | -0.0260362 | 0.029242 | -0.89036 | 0.373272 |
| C18 | **rs6701037** | -0.0130048 | 0.029616 | -0.43912 | 0.660575 |
| C18 | **rs2548145** | -0.0382239 | 0.029618 | -1.29056 | 0.196858 |
| C18 | **rs1353899** | -0.0259805 | 0.037187 | -0.69864 | 0.48478 |
| C18 | **rs10849915** | -0.0013814 | 0.030818 | -0.04482 | 0.964248 |
| C18 | **rs1000579** | 0.0383644 | 0.030234 | 1.2689 | 0.204477 |
| C18 | **rs9871864** | -0.0217633 | 0.029657 | -0.73384 | 0.463049 |
| C18 | **rs6716455** | 0.0363432 | 0.044575 | 0.815323 | 0.414888 |
| C18 | **rs9512637** | 0.0329089 | 0.030926 | 1.064113 | 0.287277 |
| C18 | **rs750338** | -0.008158 | 0.035481 | -0.22993 | 0.81815 |
| C18 | **rs1497571** | -0.035632 | 0.033092 | -1.07677 | 0.281584 |
| C18 | **rs4761097** | -0.0327783 | 0.02932 | -1.11795 | 0.263587 |
| C18 | **rs1800759** | 0.0522696 | 0.029815 | 1.753145 | 0.079577 |
| C18 | **rs2154294** | -0.0192981 | 0.029946 | -0.64443 | 0.519299 |
| C18 | **rs1793257** | 0.0883312 | 0.077347 | 1.142016 | 0.253448 |
| C18 | **rs12472151** | 0.0163346 | 0.071764 | 0.227616 | 0.819945 |
| C18 | **rs1908556** | 0.0365178 | 0.042223 | 0.864882 | 0.387104 |
| C18 | **rs2140418** | 0.0174551 | 0.038204 | 0.456896 | 0.647746 |
| C18 | **rs9825310** | 0.0258364 | 0.029888 | 0.864453 | 0.387339 |
| C18 | **rs13160562** | 0.0003104 | 0.032111 | 0.009668 | 0.992286 |
| C18 | **rs4758317** | 0.0045887 | 0.030151 | 0.152191 | 0.879036 |
| C18 | **rs8062326** | 0.0062104 | 0.088674 | 0.070036 | 0.944165 |
| C18 | **rs2810114** | -0.0177518 | 0.032865 | -0.54014 | 0.589101 |
| C18 | **rs195204** | 0.0501325 | 0.03479 | 1.440999 | 0.149585 |
| C18 | **rs1230165** | 0.1068266 | 0.037468 | 2.851167 | 0.004356 |
| C18 | **rs9556711** | 0.1782245 | 0.062408 | 2.855807 | 0.004293 |
| C18 | **rs3762894** | -0.0216371 | 0.038674 | -0.55948 | 0.575836 |
| C18 | **rs1789891** | -0.0220154 | 0.040221 | -0.54737 | 0.584128 |
| C18 | **rs1876831** | 0.1019466 | 0.057047 | 1.787072 | 0.073926 |
| C18 | **rs284786** | -0.0109146 | 0.032019 | -0.34088 | 0.733193 |
| C18 | **rs12311304** | -0.0113286 | 0.031707 | -0.35729 | 0.720878 |
| C18 | **rs768048** | 0.0220485 | 0.043522 | 0.506609 | 0.612429 |
| C18 | **rs2380220** | 0.0471772 | 0.041763 | 1.129655 | 0.258622 |
| C18 | **rs4440177** | -0.0107883 | 0.03107 | -0.34722 | 0.728422 |
| C18 | **rs4543123** | 0.0453531 | 0.035349 | 1.283019 | 0.199486 |
| C18 | **rs3131513** | -0.0103635 | 0.030218 | -0.34296 | 0.731628 |
| C18 | **rs4770403** | 0.0033587 | 0.038527 | 0.087179 | 0.930529 |
| C18 | **rs7144649** | 0.0189979 | 0.035216 | 0.53947 | 0.589563 |
| C18 | **rs3930234** | -0.0407024 | 0.040819 | -0.99715 | 0.318694 |
| C18 | **rs10253361** | -0.0106979 | 0.029655 | -0.36075 | 0.71829 |
| C18 | **rs1824024** | 0.0100726 | 0.031738 | 0.317372 | 0.750962 |
| C18 | **rs3764435** | 0.0005326 | 0.029251 | 0.01821 | 0.985472 |
| C18 | **rs9656709** | -0.0131477 | 0.029998 | -0.43829 | 0.661178 |
| C18 | **rs13259667** | -0.0088293 | 0.053881 | -0.16387 | 0.869835 |
| C18 | **rs7590720** | -0.0261897 | 0.033001 | -0.79359 | 0.427432 |
| C18 | **rs237238** | -0.0196734 | 0.059788 | -0.32905 | 0.742117 |
| C18 | **rs6943555** | -0.0198962 | 0.034509 | -0.57655 | 0.564244 |
| C18 | **rs10908907** | -0.0539688 | 0.034145 | -1.58057 | 0.113977 |
| C18 | **rs1864982** | -0.0257582 | 0.043743 | -0.58886 | 0.555958 |
| C18 | **rs7553212** | 0.0175573 | 0.031328 | 0.560437 | 0.575181 |
| C18 | **rs279861** | 0.0016633 | 0.059133 | 0.028128 | 0.97756 |
| C18 | **rs12388359** | 0.0275873 | 0.037353 | 0.738555 | 0.460177 |
| C18 | **rs1573496** | 0.0195197 | 0.049864 | 0.391463 | 0.695455 |
| C18 | **rs2228093** | -0.0702822 | 0.045138 | -1.55705 | 0.119458 |
| C18 | **rs1109501** | 0.0446313 | 0.034325 | 1.300258 | 0.193513 |
| C18 | **rs62202398** | -0.0798268 | 0.061745 | -1.29286 | 0.196061 |
| C18 | **rs8040009** | 0.0844783 | 0.038022 | 2.221851 | 0.026293 |
| C18 | **rs2188561** | 0.0039114 | 0.035544 | 0.110044 | 0.912375 |
| C18 | **rs36061340** | -0.038714 | 0.060082 | -0.64436 | 0.519345 |
| C18 | **rs11724320** | 0.0593876 | 0.031292 | 1.89785 | 0.057716 |
| C18 | **rs59972978** | -0.0033279 | 0.039325 | -0.08463 | 0.932559 |
| C21 | **rs11851015** | 0.0083494 | 0.036055 | 0.231571 | 0.816871 |
| C21 | **rs9656709** | -0.0010199 | 0.024867 | -0.04102 | 0.967284 |
| C21 | **rs12311304** | -0.0001424 | 0.026548 | -0.00536 | 0.99572 |
| C21 | **rs3930234** | -0.0140913 | 0.034087 | -0.4134 | 0.679315 |
| C21 | **rs1229984** | 0.0585844 | 0.081791 | 0.716267 | 0.473826 |
| C21 | **rs886205** | 0.0294795 | 0.032127 | 0.917582 | 0.358838 |
| C21 | **rs62202398** | -0.140132 | 0.052954 | -2.64628 | 0.008138 |
| C21 | **rs8040009** | 0.0566167 | 0.032155 | 1.760731 | 0.078284 |
| C21 | **rs242938** | -0.0004584 | 0.050067 | -0.00915 | 0.992696 |
| C21 | **rs1824024** | -0.0113922 | 0.026521 | -0.42955 | 0.667521 |
| C21 | **rs13160562** | 0.0295237 | 0.026709 | 1.105391 | 0.26899 |
| C21 | **rs6902771** | -0.0005456 | 0.024438 | -0.02232 | 0.982189 |
| C21 | **rs642899** | 0.0114893 | 0.028845 | 0.398317 | 0.690396 |
| C21 | **rs11724320** | -0.0120041 | 0.02603 | -0.46117 | 0.644676 |
| C21 | **rs3819197** | -0.0266411 | 0.029184 | -0.91288 | 0.361305 |
| C21 | **rs7590720** | -0.05828 | 0.027465 | -2.12202 | 0.033837 |
| C21 | **rs279861** | -0.0368779 | 0.050186 | -0.73483 | 0.462443 |
| C21 | **rs1573496** | 0.0433207 | 0.042228 | 1.025885 | 0.304946 |
| C21 | **rs284786** | 0.0238769 | 0.027055 | 0.882522 | 0.377495 |
| C21 | **rs804292** | -0.0159063 | 0.027809 | -0.57199 | 0.567327 |
| C21 | **rs2228093** | -0.1054285 | 0.036809 | -2.86418 | 0.004181 |
| C21 | **rs1344694** | -0.048911 | 0.026718 | -1.83066 | 0.067151 |
| C21 | **rs2548145** | -0.0363767 | 0.024546 | -1.48196 | 0.138352 |
| C21 | **rs4293630** | -0.0405665 | 0.036368 | -1.11546 | 0.264654 |
| C21 | **rs2100290** | -3.10E-06 | 0.02459 | -0.00013 | 0.999899 |
| C21 | **rs1793257** | -0.0218957 | 0.063559 | -0.3445 | 0.730473 |
| C21 | **rs12388359** | 0.0200145 | 0.030021 | 0.666676 | 0.504979 |
| C21 | **rs4440177** | 0.0401613 | 0.026013 | 1.543904 | 0.122612 |
| C21 | **rs1000579** | -0.0156503 | 0.025191 | -0.62126 | 0.534428 |
| C21 | **rs16985179** | 0.0731951 | 0.042628 | 1.71705 | 0.08597 |
| C21 | **rs4478858** | -0.0206683 | 0.024831 | -0.83235 | 0.405212 |
| C21 | **rs7144649** | -0.044733 | 0.029498 | -1.51649 | 0.129395 |
| C21 | **rs1864982** | 0.0355899 | 0.035899 | 0.991378 | 0.321501 |
| C21 | **rs10253361** | 0.0075426 | 0.024844 | 0.303594 | 0.761437 |
| C21 | **rs1789891** | 0.0014211 | 0.033685 | 0.042188 | 0.966349 |
| C21 | **rs1042026** | -0.012219 | 0.027513 | -0.44412 | 0.656956 |
| C21 | **rs933769** | -0.0337598 | 0.032108 | -1.05145 | 0.293055 |
| C21 | **rs237238** | 0.0098112 | 0.04877 | 0.201174 | 0.840562 |
| C21 | **rs6716455** | 0.0773503 | 0.03669 | 2.108201 | 0.035014 |
| C21 | **rs9512637** | 0.0238164 | 0.025772 | 0.924111 | 0.355429 |
| C21 | **rs3131513** | -0.0311216 | 0.025295 | -1.23035 | 0.218567 |
| C21 | **rs6943555** | 0.0322995 | 0.028828 | 1.120404 | 0.262542 |
| C21 | **rs2154294** | -0.0654621 | 0.024784 | -2.64133 | 0.008258 |
| C21 | **rs8062326** | 0.0225837 | 0.070765 | 0.319136 | 0.749623 |
| C21 | **rs2810114** | -0.0258871 | 0.027394 | -0.94499 | 0.344663 |
| C21 | **rs1230165** | 0.003512 | 0.031281 | 0.112271 | 0.910609 |
| C21 | **rs750338** | -0.003124 | 0.029344 | -0.10646 | 0.915216 |
| C21 | **rs10893366** | -0.0306312 | 0.032802 | -0.93382 | 0.350397 |
| C21 | **rs2380220** | 0.0376612 | 0.034478 | 1.092338 | 0.274684 |
| C21 | **rs59677118** | -0.0290729 | 0.043633 | -0.6663 | 0.505219 |
| C21 | **rs10849915** | 0.0365174 | 0.026071 | 1.400701 | 0.161304 |
| C21 | **rs4770403** | 0.0425553 | 0.03148 | 1.351807 | 0.176437 |
| C21 | **rs3738443** | 0.040687 | 0.032648 | 1.246231 | 0.21268 |
| C21 | **rs195204** | 0.0309406 | 0.029004 | 1.066758 | 0.286081 |
| C21 | **rs1318937** | -0.0095844 | 0.036862 | -0.26001 | 0.794856 |
| C21 | **rs2369955** | 0.0032509 | 0.038006 | 0.085536 | 0.931836 |
| C21 | **rs67031482** | 0.0113476 | 0.02482 | 0.4572 | 0.647527 |
| C21 | **rs36563** | -0.0195311 | 0.033878 | -0.57652 | 0.564263 |
| C21 | **rs768048** | 0.0049316 | 0.037131 | 0.132814 | 0.894341 |
| C21 | **rs2303317** | -0.0297477 | 0.024489 | -1.21473 | 0.224468 |
| C21 | **rs567926** | -0.0254123 | 0.049366 | -0.51477 | 0.606712 |
| C21 | **rs1908556** | 0.0400228 | 0.035088 | 1.140648 | 0.254017 |
| C21 | **rs1497571** | -0.0009494 | 0.027857 | -0.03408 | 0.972813 |
| C21 | **rs420817** | -0.0146782 | 0.024444 | -0.60048 | 0.548185 |
| C21 | **rs9636231** | -0.0181974 | 0.027594 | -0.65948 | 0.509588 |
| C21 | **rs4761097** | -0.0430297 | 0.024427 | -1.76156 | 0.078144 |
| C21 | **rs7553212** | 0.0169042 | 0.025929 | 0.651953 | 0.514432 |
| C21 | **rs2188561** | -0.0190858 | 0.029553 | -0.64582 | 0.518395 |
| C21 | **rs1380131** | 0.025992 | 0.043553 | 0.596784 | 0.550651 |
| C21 | **rs2827312** | 0.0132446 | 0.027287 | 0.485384 | 0.627404 |
| C21 | **rs2140418** | -0.035533 | 0.031764 | -1.11866 | 0.263286 |
| C21 | **rs1800759** | 0.0044741 | 0.024847 | 0.180067 | 0.8571 |
| C21 | **rs59972978** | 0.0188558 | 0.03277 | 0.575399 | 0.565021 |
| C21 | **rs1109501** | 0.019219 | 0.028823 | 0.666799 | 0.5049 |
| C21 | **rs4543123** | -0.0083997 | 0.028965 | -0.28999 | 0.771824 |
| C21 | **rs9825310** | -0.0224419 | 0.024802 | -0.90485 | 0.365543 |
| C21 | **rs1876831** | 0.0502157 | 0.048715 | 1.030813 | 0.302628 |
| C21 | **rs13259667** | -0.0347304 | 0.046542 | -0.74622 | 0.455536 |
| C21 | **rs10908907** | -0.0134493 | 0.028113 | -0.47841 | 0.632361 |
| C21 | **rs3764435** | -0.0242487 | 0.0246 | -0.9857 | 0.324279 |
| C21 | **rs9871864** | -0.0029313 | 0.024857 | -0.11793 | 0.906127 |
| C21 | **rs1353899** | -0.0032789 | 0.030724 | -0.10672 | 0.91501 |
| C21 | **rs4758317** | -0.0168809 | 0.025236 | -0.66893 | 0.503541 |
| C21 | **rs12472151** | 0.1104415 | 0.060236 | 1.833493 | 0.066729 |
| C21 | **rs36061340** | -0.0016202 | 0.050551 | -0.03205 | 0.974432 |
| C21 | **rs3762894** | -0.0233537 | 0.032988 | -0.70794 | 0.478982 |
| C21 | **rs1353621** | 0.0387152 | 0.025746 | 1.50371 | 0.132656 |
| C21 | **rs9556711** | -0.0749524 | 0.050472 | -1.48505 | 0.137532 |
| C21 | **rs6701037** | -0.0037067 | 0.025057 | -0.14793 | 0.882396 |
| M0 | **rs1000579** | 0.0009935 | 0.013151 | 0.075547 | 0.939779 |
| M0 | **rs2827312** | -0.0074042 | 0.0142 | -0.52144 | 0.60206 |
| M0 | **rs7144649** | -0.0237431 | 0.015617 | -1.52035 | 0.128423 |
| M0 | **rs2548145** | -0.0040662 | 0.012976 | -0.31337 | 0.754 |
| M0 | **rs7553212** | 0.0132065 | 0.013822 | 0.955479 | 0.339336 |
| M0 | **rs9512637** | -0.0025665 | 0.013409 | -0.1914 | 0.848214 |
| M0 | **rs2188561** | 0.0094729 | 0.015417 | 0.614431 | 0.538931 |
| M0 | **rs59972978** | 0.0231567 | 0.017211 | 1.345468 | 0.178474 |
| M0 | **rs6716455** | -0.0047899 | 0.018682 | -0.25639 | 0.797648 |
| M0 | **rs1353621** | 0.012916 | 0.013399 | 0.96399 | 0.335051 |
| M0 | **rs13160562** | -0.0178373 | 0.013829 | -1.28983 | 0.19711 |
| M0 | **rs13259667** | -0.0002412 | 0.024419 | -0.00988 | 0.992121 |
| M0 | **rs3131513** | 0.0268902 | 0.013267 | 2.026859 | 0.042677 |
| M0 | **rs1109501** | 0.0285816 | 0.014916 | 1.916171 | 0.055343 |
| M0 | **rs2140418** | -0.0093542 | 0.016362 | -0.57169 | 0.567535 |
| M0 | **rs6701037** | 0.016105 | 0.01305 | 1.234112 | 0.217161 |
| M0 | **rs284786** | 0.0050678 | 0.014141 | 0.358378 | 0.720061 |
| M0 | **rs2810114** | -0.0278101 | 0.014574 | -1.90819 | 0.056367 |
| M0 | **rs36061340** | 0.0085248 | 0.027373 | 0.311435 | 0.75547 |
| M0 | **rs16985179** | -0.0092002 | 0.022751 | -0.40439 | 0.685925 |
| M0 | **rs9556711** | 0.03833 | 0.027371 | 1.400376 | 0.161401 |
| M0 | **rs2380220** | 0.0183059 | 0.018314 | 0.999575 | 0.317516 |
| M0 | **rs12311304** | -0.0089747 | 0.013967 | -0.64258 | 0.520498 |
| M0 | **rs10908907** | -0.0179492 | 0.014947 | -1.20083 | 0.229818 |
| M0 | **rs804292** | -0.0093707 | 0.015134 | -0.61918 | 0.535795 |
| M0 | **rs36563** | -0.0283482 | 0.01817 | -1.56016 | 0.118721 |
| M0 | **rs12388359** | 0.0211657 | 0.019261 | 1.098872 | 0.271824 |
| M0 | **rs1800759** | -0.0030509 | 0.013235 | -0.23052 | 0.817686 |
| M0 | **rs4770403** | -0.0395688 | 0.016608 | -2.38245 | 0.017198 |
| M0 | **rs8062326** | -0.0403129 | 0.036959 | -1.09075 | 0.275382 |
| M0 | **rs1230165** | 0.0165373 | 0.016604 | 0.996002 | 0.319249 |
| M0 | **rs1353899** | -0.0214514 | 0.016467 | -1.30272 | 0.192669 |
| M0 | **rs6943555** | -0.0235935 | 0.014964 | -1.57664 | 0.114878 |
| M0 | **rs1042026** | -0.0040685 | 0.014279 | -0.28492 | 0.775702 |
| M0 | **rs1573496** | -0.0014623 | 0.021819 | -0.06702 | 0.946567 |
| M0 | **rs59677118** | 0.0034603 | 0.022646 | 0.152801 | 0.878555 |
| M0 | **rs3738443** | -0.0094419 | 0.017329 | -0.54487 | 0.585841 |
| M0 | **rs4293630** | -0.0289255 | 0.019166 | -1.50922 | 0.131242 |
| M0 | **rs2154294** | 0.013178 | 0.012981 | 1.015179 | 0.310021 |
| M0 | **rs750338** | 0.0276893 | 0.015595 | 1.775499 | 0.075816 |
| M0 | **rs1318937** | 0.0294473 | 0.019148 | 1.537855 | 0.124084 |
| M0 | **rs2100290** | -0.0355752 | 0.012939 | -2.74939 | 0.005971 |
| M0 | **rs10849915** | -0.0034679 | 0.013736 | -0.25246 | 0.800684 |
| M0 | **rs8040009** | 0.0068631 | 0.016931 | 0.405351 | 0.685219 |
| M0 | **rs3764435** | 0.0129687 | 0.013187 | 0.983419 | 0.325401 |
| M0 | **rs242938** | -0.0447663 | 0.027011 | -1.65736 | 0.097446 |
| M0 | **rs9656709** | 0.0116093 | 0.013088 | 0.887054 | 0.37505 |
| M0 | **rs9871864** | -0.0054328 | 0.013088 | -0.41509 | 0.678075 |
| M0 | **rs62202398** | -0.0479517 | 0.026754 | -1.79232 | 0.073082 |
| M0 | **rs10893366** | 0.0146552 | 0.017504 | 0.837263 | 0.402445 |
| M0 | **rs768048** | -0.0117174 | 0.018975 | -0.61752 | 0.536891 |
| M0 | **rs1380131** | 0.0222225 | 0.022585 | 0.983935 | 0.325147 |
| M0 | **rs4758317** | 0.011973 | 0.013259 | 0.903042 | 0.366504 |
| M0 | **rs6902771** | 0.0058975 | 0.012924 | 0.456322 | 0.648159 |
| M0 | **rs237238** | -0.0073764 | 0.02591 | -0.28469 | 0.775882 |
| M0 | **rs279861** | -0.0056638 | 0.013115 | -0.43187 | 0.665835 |
| M0 | **rs1793257** | 0.020424 | 0.03511 | 0.581721 | 0.560755 |
| M0 | **rs3819197** | 0.0085087 | 0.015185 | 0.560326 | 0.575257 |
| M0 | **rs195204** | 0.0076379 | 0.01501 | 0.508845 | 0.610861 |
| M0 | **rs2303317** | 0.0043423 | 0.013069 | 0.332248 | 0.739702 |
| M0 | **rs9825310** | -0.0024836 | 0.013043 | -0.19041 | 0.848985 |
| M0 | **rs7590720** | 0.0031694 | 0.014329 | 0.221193 | 0.824942 |
| M0 | **rs1229984** | -0.1910053 | 0.05051 | -3.78156 | 0.000156 |
| M0 | **rs11724320** | -0.0220191 | 0.013813 | -1.59412 | 0.110909 |
| M0 | **rs1789891** | 0.0199797 | 0.017548 | 1.138584 | 0.254877 |
| M0 | **rs2228093** | 0.0311346 | 0.019188 | 1.622595 | 0.104676 |
| M0 | **rs642899** | 0.0362376 | 0.015392 | 2.354331 | 0.018556 |
| M0 | **rs1497571** | 0.017002 | 0.013009 | 1.306922 | 0.191239 |
| M0 | **rs3762894** | 0.0203845 | 0.017543 | 1.161983 | 0.245243 |
| M0 | **rs1908556** | -0.007202 | 0.018709 | -0.38496 | 0.700268 |
| M0 | **rs4543123** | -0.004548 | 0.015443 | -0.2945 | 0.768373 |
| M0 | **rs2369955** | -0.0291346 | 0.019578 | -1.48813 | 0.136716 |
| M0 | **rs1344694** | 0.0029344 | 0.013857 | 0.211767 | 0.832289 |
| M0 | **rs1824024** | -0.0015725 | 0.014058 | -0.11186 | 0.910933 |
| M0 | **rs1864982** | -0.0217544 | 0.019538 | -1.11346 | 0.265512 |
| M0 | **rs886205** | -0.0178087 | 0.017045 | -1.04478 | 0.296125 |
| M0 | **rs4440177** | 0.010898 | 0.013736 | 0.793412 | 0.427538 |
| M0 | **rs933769** | -0.0199515 | 0.016818 | -1.18633 | 0.235491 |
| M0 | **rs9636231** | -0.0060464 | 0.01445 | -0.41843 | 0.67563 |
| M0 | **rs10253361** | 0.0028319 | 0.013009 | 0.217687 | 0.827673 |
| M0 | **rs67031482** | -0.0083303 | 0.01304 | -0.63885 | 0.522921 |
| M0 | **rs567926** | -0.0052671 | 0.013117 | -0.40156 | 0.688004 |
| M0 | **rs420817** | 0.0085734 | 0.012986 | 0.660189 | 0.509132 |
| M0 | **rs11851015** | -0.025743 | 0.019229 | -1.33877 | 0.180645 |
| M0 | **rs3930234** | 0.0199941 | 0.018155 | 1.101281 | 0.270774 |
| M0 | **rs1876831** | -0.0399422 | 0.015393 | -2.59481 | 0.009465 |
| M0 | **rs4478858** | -0.02555 | 0.013022 | -1.96207 | 0.049754 |
| M0 | **rs12472151** | -0.0091186 | 0.031118 | -0.29303 | 0.769497 |
| M0 | **rs4761097** | -0.0039738 | 0.013028 | -0.30502 | 0.760348 |
| M1 | **rs62202398** | -0.0238769 | 0.020179 | -1.18327 | 0.236702 |
| M1 | **rs2154294** | 0.0042842 | 0.009774 | 0.438343 | 0.661138 |
| M1 | **rs1380131** | 0.0161397 | 0.017164 | 0.94035 | 0.347038 |
| M1 | **rs1573496** | 0.0039477 | 0.016317 | 0.241946 | 0.808822 |
| M1 | **rs1042026** | -0.0158314 | 0.010746 | -1.47321 | 0.140695 |
| M1 | **rs2188561** | 0.0104854 | 0.01163 | 0.901595 | 0.367272 |
| M1 | **rs12472151** | 0.0188266 | 0.023359 | 0.805954 | 0.420269 |
| M1 | **rs642899** | 0.020437 | 0.011604 | 1.761245 | 0.078197 |
| M1 | **rs279861** | -0.020283 | 0.009908 | -2.04713 | 0.040645 |
| M1 | **rs2380220** | -0.0055791 | 0.01376 | -0.40546 | 0.685141 |
| M1 | **rs886205** | 0.0059083 | 0.012784 | 0.462153 | 0.643972 |
| M1 | **rs10253361** | 0.0133036 | 0.009807 | 1.356552 | 0.174924 |
| M1 | **rs9825310** | 0.0039703 | 0.009849 | 0.403097 | 0.686877 |
| M1 | **rs36563** | -0.0228905 | 0.01366 | -1.67577 | 0.093782 |
| M1 | **rs3764435** | 0.0098505 | 0.009944 | 0.990642 | 0.321861 |
| M1 | **rs9871864** | 0.0010957 | 0.009891 | 0.110779 | 0.911792 |
| M1 | **rs4478858** | -0.0019426 | 0.009821 | -0.1978 | 0.8432 |
| M1 | **rs4758317** | 0.0041944 | 0.010011 | 0.41898 | 0.675231 |
| M1 | **rs284786** | -0.0096956 | 0.010692 | -0.90684 | 0.36449 |
| M1 | **rs11851015** | -0.0083355 | 0.014394 | -0.5791 | 0.56252 |
| M1 | **rs6902771** | 0.0111471 | 0.009745 | 1.143929 | 0.252653 |
| M1 | **rs4770403** | -0.0248531 | 0.012539 | -1.98209 | 0.047469 |
| M1 | **rs11724320** | -0.0165211 | 0.010428 | -1.58437 | 0.113109 |
| M1 | **rs804292** | 0.0076983 | 0.011434 | 0.673289 | 0.500764 |
| M1 | **rs2810114** | -0.0100055 | 0.010974 | -0.91176 | 0.361893 |
| M1 | **rs13160562** | -0.014886 | 0.010446 | -1.42504 | 0.154146 |
| M1 | **rs2303317** | 0.0077856 | 0.00984 | 0.791214 | 0.428819 |
| M1 | **rs1353621** | 0.0145558 | 0.010145 | 1.434726 | 0.151365 |
| M1 | **rs1824024** | 0.0049989 | 0.010608 | 0.471253 | 0.63746 |
| M1 | **rs3930234** | 0.0077299 | 0.013609 | 0.568016 | 0.570024 |
| M1 | **rs1230165** | 0.0097262 | 0.012559 | 0.774465 | 0.438656 |
| M1 | **rs1109501** | -0.0166246 | 0.011258 | -1.47676 | 0.139741 |
| M1 | **rs9556711** | 0.0348377 | 0.020679 | 1.684711 | 0.092044 |
| M1 | **rs933769** | -0.0111775 | 0.012693 | -0.88064 | 0.378514 |
| M1 | **rs67031482** | 0.0067566 | 0.009821 | 0.687987 | 0.491461 |
| M1 | **rs1876831** | -0.0119432 | 0.011581 | -1.03129 | 0.302407 |
| M1 | **rs10849915** | 0.0034453 | 0.010358 | 0.332621 | 0.739421 |
| M1 | **rs1344694** | -0.0043151 | 0.010399 | -0.41495 | 0.678175 |
| M1 | **rs1800759** | 0.0113732 | 0.009965 | 1.141356 | 0.253722 |
| M1 | **rs6943555** | -0.0112183 | 0.011302 | -0.99261 | 0.320899 |
| M1 | **rs1000579** | 0.0101349 | 0.009915 | 1.022154 | 0.306708 |
| M1 | **rs2548145** | 0.0034592 | 0.00981 | 0.352625 | 0.72437 |
| M1 | **rs8062326** | -0.0237375 | 0.028178 | -0.84242 | 0.399552 |
| M1 | **rs36061340** | 0.0167883 | 0.020581 | 0.815708 | 0.414667 |
| M1 | **rs9512637** | -0.0073463 | 0.010095 | -0.72774 | 0.466774 |
| M1 | **rs2140418** | -0.0040896 | 0.012341 | -0.33138 | 0.74036 |
| M1 | **rs59677118** | 0.0127899 | 0.016972 | 0.753583 | 0.4511 |
| M1 | **rs2369955** | 0.0105398 | 0.014705 | 0.716773 | 0.473514 |
| M1 | **rs59972978** | 0.0194996 | 0.012978 | 1.502502 | 0.132968 |
| M1 | **rs9636231** | 0.0012879 | 0.010939 | 0.117736 | 0.906277 |
| M1 | **rs1864982** | 0.0002218 | 0.01464 | 0.015148 | 0.987915 |
| M1 | **rs750338** | 0.0025836 | 0.011756 | 0.219765 | 0.826054 |
| M1 | **rs237238** | 0.016045 | 0.019464 | 0.824336 | 0.409749 |
| M1 | **rs10893366** | -0.0020197 | 0.013201 | -0.153 | 0.878402 |
| M1 | **rs1497571** | -0.0035685 | 0.009832 | -0.36294 | 0.716653 |
| M1 | **rs6701037** | 0.0066771 | 0.009828 | 0.679395 | 0.496888 |
| M1 | **rs195204** | 0.0023139 | 0.011317 | 0.204473 | 0.837984 |
| M1 | **rs2100290** | -0.0119819 | 0.009776 | -1.2256 | 0.220348 |
| M1 | **rs1229984** | -0.0681675 | 0.038041 | -1.79196 | 0.073139 |
| M1 | **rs2228093** | 0.0036959 | 0.014579 | 0.253512 | 0.799873 |
| M1 | **rs1318937** | 0.0124081 | 0.014583 | 0.85086 | 0.394847 |
| M1 | **rs567926** | -0.0197853 | 0.009916 | -1.99537 | 0.046003 |
| M1 | **rs4543123** | -0.0038403 | 0.01166 | -0.32934 | 0.741897 |
| M1 | **rs4761097** | -0.0066046 | 0.009829 | -0.67194 | 0.501621 |
| M1 | **rs12311304** | -0.0075826 | 0.010533 | -0.71992 | 0.471575 |
| M1 | **rs3131513** | 0.0132953 | 0.010025 | 1.326172 | 0.184783 |
| M1 | **rs2827312** | 0.0070766 | 0.010718 | 0.660237 | 0.509102 |
| M1 | **rs4293630** | -0.0048587 | 0.014474 | -0.3357 | 0.737098 |
| M1 | **rs420817** | -0.0044596 | 0.00982 | -0.45412 | 0.64974 |
| M1 | **rs7553212** | -0.0006204 | 0.010354 | -0.05992 | 0.952222 |
| M1 | **rs10908907** | 0.0022638 | 0.011266 | 0.200941 | 0.840745 |
| M1 | **rs9656709** | -0.0028496 | 0.009883 | -0.28833 | 0.773094 |
| M1 | **rs1353899** | 0.0007433 | 0.012453 | 0.059693 | 0.9524 |
| M1 | **rs1789891** | 0.0070599 | 0.013198 | 0.534936 | 0.592694 |
| M1 | **rs7144649** | -0.0071242 | 0.011766 | -0.60549 | 0.544852 |
| M1 | **rs6716455** | -0.0071798 | 0.014127 | -0.50822 | 0.611298 |
| M1 | **rs8040009** | -0.0077547 | 0.012768 | -0.60736 | 0.543613 |
| M1 | **rs4440177** | 0.0110161 | 0.010346 | 1.06477 | 0.28698 |
| M1 | **rs13259667** | 0.0023568 | 0.018391 | 0.128152 | 0.898029 |
| M1 | **rs16985179** | 0.0151829 | 0.01714 | 0.885829 | 0.37571 |
| M1 | **rs242938** | -0.009846 | 0.020213 | -0.4871 | 0.626185 |
| M1 | **rs3819197** | -0.0119626 | 0.011414 | -1.04803 | 0.294626 |
| M1 | **rs768048** | 0.0041255 | 0.014244 | 0.289622 | 0.772105 |
| M1 | **rs7590720** | -0.0067971 | 0.010716 | -0.63427 | 0.525903 |
| M1 | **rs12388359** | 0.0013766 | 0.01449 | 0.095007 | 0.92431 |
| M1 | **rs1793257** | -0.012321 | 0.026162 | -0.47095 | 0.637678 |
| M1 | **rs3762894** | -0.0153045 | 0.013168 | -1.16229 | 0.245119 |
| M1 | **rs1908556** | -0.0110277 | 0.014144 | -0.7797 | 0.43557 |
| M1 | **rs3738443** | -0.0125818 | 0.013068 | -0.96278 | 0.335657 |
| M2 | **rs886205** | -0.0036452 | 0.018049 | -0.20196 | 0.839949 |
| M2 | **rs3738443** | 0.0309967 | 0.018463 | 1.678856 | 0.09318 |
| M2 | **rs9556711** | 0.0014331 | 0.029333 | 0.048854 | 0.961035 |
| M2 | **rs9636231** | -0.0064579 | 0.015323 | -0.42146 | 0.673417 |
| M2 | **rs3764435** | 0.0102998 | 0.013964 | 0.737601 | 0.460757 |
| M2 | **rs4758317** | 0.0207014 | 0.014048 | 1.473612 | 0.140586 |
| M2 | **rs9512637** | -0.0010637 | 0.014203 | -0.07489 | 0.9403 |
| M2 | **rs4543123** | 0.0035334 | 0.01644 | 0.214929 | 0.829823 |
| M2 | **rs6716455** | -0.0012136 | 0.019919 | -0.06093 | 0.951416 |
| M2 | **rs6943555** | -0.0185699 | 0.015806 | -1.17486 | 0.240052 |
| M2 | **rs10253361** | -0.0002281 | 0.013773 | -0.01656 | 0.986785 |
| M2 | **rs1353621** | 0.0049687 | 0.014203 | 0.349837 | 0.726461 |
| M2 | **rs2548145** | 0.0121427 | 0.01384 | 0.877385 | 0.380278 |
| M2 | **rs4293630** | 0.0089147 | 0.020305 | 0.43905 | 0.660625 |
| M2 | **rs67031482** | 0.0217075 | 0.013873 | 1.564694 | 0.117655 |
| M2 | **rs1800759** | 0.0222299 | 0.014048 | 1.582388 | 0.113561 |
| M2 | **rs1318937** | 0.0184108 | 0.020479 | 0.898998 | 0.368654 |
| M2 | **rs59677118** | -0.013544 | 0.023953 | -0.56545 | 0.571768 |
| M2 | **rs7590720** | -0.0063384 | 0.015192 | -0.41723 | 0.676512 |
| M2 | **rs11724320** | -0.0248345 | 0.014632 | -1.69729 | 0.089642 |
| M2 | **rs8040009** | 0.0165533 | 0.017979 | 0.920712 | 0.357201 |
| M2 | **rs9656709** | 0.0106868 | 0.013905 | 0.768541 | 0.442166 |
| M2 | **rs3930234** | 0.0048554 | 0.019123 | 0.253906 | 0.799569 |
| M2 | **rs3131513** | 0.0146077 | 0.014042 | 1.040265 | 0.298217 |
| M2 | **rs3819197** | -0.0396793 | 0.015953 | -2.48734 | 0.01287 |
| M2 | **rs1380131** | 0.0046334 | 0.02405 | 0.192653 | 0.84723 |
| M2 | **rs237238** | -0.0224287 | 0.027252 | -0.82301 | 0.410505 |
| M2 | **rs279861** | -0.0200928 | 0.013873 | -1.44836 | 0.147517 |
| M2 | **rs1042026** | -0.0384688 | 0.01506 | -2.55435 | 0.010639 |
| M2 | **rs195204** | 0.0147225 | 0.015926 | 0.924453 | 0.35525 |
| M2 | **rs7144649** | 0.0144868 | 0.016512 | 0.877379 | 0.380281 |
| M2 | **rs1793257** | 0.0203069 | 0.036748 | 0.552594 | 0.580541 |
| M2 | **rs1229984** | -0.1414479 | 0.052578 | -2.69024 | 0.00714 |
| M2 | **rs1876831** | -0.0171515 | 0.016259 | -1.05488 | 0.29148 |
| M2 | **rs933769** | -0.0346661 | 0.01775 | -1.95299 | 0.050821 |
| M2 | **rs2188561** | -0.0124654 | 0.016377 | -0.76116 | 0.446559 |
| M2 | **rs2380220** | 0.0259434 | 0.019349 | 1.340819 | 0.179979 |
| M2 | **rs12472151** | 0.0361774 | 0.033032 | 1.09521 | 0.273425 |
| M2 | **rs6902771** | 0.0203976 | 0.013678 | 1.491246 | 0.135897 |
| M2 | **rs11851015** | -0.0078524 | 0.020075 | -0.39116 | 0.695678 |
| M2 | **rs4770403** | -0.0432884 | 0.01763 | -2.45543 | 0.014072 |
| M2 | **rs4761097** | 0.0193071 | 0.01383 | 1.396052 | 0.162699 |
| M2 | **rs1344694** | -0.0103698 | 0.014715 | -0.70471 | 0.480991 |
| M2 | **rs1864982** | -0.0037454 | 0.020643 | -0.18144 | 0.856026 |
| M2 | **rs62202398** | -0.0241531 | 0.028322 | -0.8528 | 0.393769 |
| M2 | **rs2810114** | 0.0141085 | 0.015469 | 0.912042 | 0.361747 |
| M2 | **rs1000579** | 0.0034381 | 0.013936 | 0.246715 | 0.805129 |
| M2 | **rs2140418** | -0.008608 | 0.017476 | -0.49256 | 0.622324 |
| M2 | **rs2100290** | -0.0190423 | 0.013756 | -1.38428 | 0.166273 |
| M2 | **rs768048** | 0.0328598 | 0.019959 | 1.646384 | 0.099685 |
| M2 | **rs1824024** | -0.0096184 | 0.014942 | -0.64374 | 0.519746 |
| M2 | **rs13259667** | -0.0035288 | 0.025897 | -0.13626 | 0.891614 |
| M2 | **rs4478858** | -0.0214352 | 0.01382 | -1.55105 | 0.12089 |
| M2 | **rs9871864** | 0.0108617 | 0.013832 | 0.785282 | 0.432288 |
| M2 | **rs1573496** | -0.0002071 | 0.022908 | -0.00904 | 0.992788 |
| M2 | **rs36563** | 0.0135502 | 0.019319 | 0.701384 | 0.483064 |
| M2 | **rs10908907** | 0.0031269 | 0.015834 | 0.19748 | 0.843452 |
| M2 | **rs10893366** | -0.0181821 | 0.018643 | -0.97528 | 0.329423 |
| M2 | **rs59972978** | 0.0250119 | 0.018284 | 1.367944 | 0.17133 |
| M2 | **rs12388359** | 0.0139753 | 0.020508 | 0.681474 | 0.495571 |
| M2 | **rs8062326** | -0.0037002 | 0.039448 | -0.0938 | 0.92527 |
| M2 | **rs750338** | 0.0000423 | 0.016598 | 0.002551 | 0.997964 |
| M2 | **rs1789891** | 0.0118702 | 0.018559 | 0.639604 | 0.52243 |
| M2 | **rs1497571** | 0.0191462 | 0.013747 | 1.392738 | 0.163699 |
| M2 | **rs2369955** | 0.0067485 | 0.020687 | 0.326224 | 0.744255 |
| M2 | **rs1353899** | -0.0017552 | 0.017584 | -0.09982 | 0.92049 |
| M2 | **rs10849915** | 0.0026961 | 0.01458 | 0.184917 | 0.853295 |
| M2 | **rs2303317** | 0.0047267 | 0.01382 | 0.342023 | 0.732333 |
| M2 | **rs16985179** | -0.0160927 | 0.023948 | -0.67198 | 0.501599 |
| M2 | **rs567926** | -0.019192 | 0.013886 | -1.38215 | 0.166926 |
| M2 | **rs36061340** | 0.0090489 | 0.028808 | 0.314111 | 0.753437 |
| M2 | **rs12311304** | -0.0201565 | 0.014802 | -1.36174 | 0.173281 |
| M2 | **rs6701037** | 0.0017661 | 0.013803 | 0.127953 | 0.898186 |
| M2 | **rs13160562** | -0.0209899 | 0.014617 | -1.43597 | 0.15101 |
| M2 | **rs1908556** | -0.0013296 | 0.01981 | -0.06712 | 0.94649 |
| M2 | **rs2827312** | 0.0034192 | 0.01507 | 0.226889 | 0.82051 |
| M2 | **rs642899** | 0.0138655 | 0.016238 | 0.853876 | 0.393174 |
| M2 | **rs1109501** | -0.0134464 | 0.015857 | -0.84796 | 0.396461 |
| M2 | **rs4440177** | 0.0185917 | 0.01454 | 1.278619 | 0.201031 |
| M2 | **rs284786** | -0.0104227 | 0.014978 | -0.69586 | 0.486514 |
| M2 | **rs242938** | -0.0204472 | 0.028425 | -0.71935 | 0.471928 |
| M2 | **rs420817** | 0.0069302 | 0.013813 | 0.501732 | 0.615856 |
| M2 | **rs9825310** | -0.0019922 | 0.013876 | -0.14357 | 0.885838 |
| M2 | **rs1230165** | 0.0373715 | 0.017741 | 2.106506 | 0.03516 |
| M2 | **rs2154294** | -0.0019645 | 0.01373 | -0.14308 | 0.886231 |
| M2 | **rs804292** | 0.0203144 | 0.016014 | 1.268515 | 0.204614 |
| M2 | **rs3762894** | -0.0274771 | 0.018408 | -1.49268 | 0.135522 |
| M2 | **rs2228093** | -0.0053148 | 0.020398 | -0.26056 | 0.794432 |
| M2 | **rs7553212** | 0.0124476 | 0.014426 | 0.86285 | 0.38822 |
| M3 | **rs1800759** | 0.003948 | 0.01532 | 0.257699 | 0.796639 |
| M3 | **rs1230165** | 0.0299877 | 0.019415 | 1.544551 | 0.122455 |
| M3 | **rs242938** | -0.0234262 | 0.03113 | -0.75253 | 0.451733 |
| M3 | **rs2369955** | -0.0089586 | 0.022671 | -0.39515 | 0.692731 |
| M3 | **rs3764435** | 0.03927 | 0.015183 | 2.586398 | 0.009699 |
| M3 | **rs12388359** | 0.0099449 | 0.022291 | 0.446147 | 0.655491 |
| M3 | **rs2154294** | -0.0106477 | 0.014906 | -0.71434 | 0.475015 |
| M3 | **rs1876831** | -0.0293334 | 0.01767 | -1.66012 | 0.096891 |
| M3 | **rs1864982** | -0.009826 | 0.022603 | -0.43472 | 0.663769 |
| M3 | **rs62202398** | -0.025723 | 0.030924 | -0.83181 | 0.405516 |
| M3 | **rs12472151** | 0.0390456 | 0.036535 | 1.068718 | 0.285197 |
| M3 | **rs237238** | 0.0032449 | 0.029854 | 0.108694 | 0.913445 |
| M3 | **rs6701037** | 0.0111759 | 0.015049 | 0.742642 | 0.457699 |
| M3 | **rs2228093** | -0.0229104 | 0.022121 | -1.03567 | 0.300356 |
| M3 | **rs67031482** | 0.006954 | 0.015152 | 0.458949 | 0.646271 |
| M3 | **rs2827312** | -0.0108534 | 0.01641 | -0.66138 | 0.508367 |
| M3 | **rs9512637** | -0.0047556 | 0.015446 | -0.3079 | 0.758161 |
| M3 | **rs195204** | -0.0180162 | 0.017342 | -1.03888 | 0.298862 |
| M3 | **rs1344694** | -0.0221387 | 0.01601 | -1.3828 | 0.166726 |
| M3 | **rs642899** | -0.004551 | 0.017736 | -0.2566 | 0.797487 |
| M3 | **rs1318937** | 0.0717426 | 0.022294 | 3.218023 | 0.001291 |
| M3 | **rs7144649** | 0.0000181 | 0.017854 | 0.001015 | 0.99919 |
| M3 | **rs1353621** | -0.0014798 | 0.015396 | -0.09612 | 0.923427 |
| M3 | **rs4758317** | 0.0111372 | 0.015264 | 0.729629 | 0.465617 |
| M3 | **rs4440177** | 0.0117212 | 0.015822 | 0.74083 | 0.458797 |
| M3 | **rs1824024** | 0.0074452 | 0.016247 | 0.458239 | 0.64678 |
| M3 | **rs1042026** | -0.0195065 | 0.016368 | -1.19174 | 0.233362 |
| M3 | **rs2100290** | -0.0158733 | 0.014998 | -1.05839 | 0.289879 |
| M3 | **rs2140418** | -0.0246062 | 0.019016 | -1.29395 | 0.195681 |
| M3 | **rs933769** | -0.0037195 | 0.019303 | -0.19269 | 0.847203 |
| M3 | **rs567926** | 0.0070744 | 0.015135 | 0.467415 | 0.640203 |
| M3 | **rs7590720** | -0.0217225 | 0.016518 | -1.31509 | 0.18848 |
| M3 | **rs284786** | 0.0073448 | 0.016353 | 0.449139 | 0.653332 |
| M3 | **rs1908556** | -0.0208511 | 0.021544 | -0.96785 | 0.333118 |
| M3 | **rs4478858** | -0.0244847 | 0.015062 | -1.62564 | 0.104027 |
| M3 | **rs9636231** | -0.0198526 | 0.016681 | -1.19014 | 0.233993 |
| M3 | **rs11724320** | -0.0106436 | 0.015965 | -0.66668 | 0.504979 |
| M3 | **rs3131513** | 0.0175985 | 0.01531 | 1.1495 | 0.25035 |
| M3 | **rs886205** | -0.0030516 | 0.019665 | -0.15518 | 0.87668 |
| M3 | **rs9656709** | -0.0015684 | 0.015173 | -0.10337 | 0.917672 |
| M3 | **rs59972978** | 0.0321704 | 0.019927 | 1.614408 | 0.106439 |
| M3 | **rs279861** | 0.0045142 | 0.015114 | 0.298684 | 0.765181 |
| M3 | **rs1109501** | -0.0123022 | 0.01725 | -0.71318 | 0.475737 |
| M3 | **rs59677118** | -0.0013474 | 0.026322 | -0.05119 | 0.959175 |
| M3 | **rs10893366** | 0.0080944 | 0.020341 | 0.397946 | 0.69067 |
| M3 | **rs420817** | 0.008991 | 0.015004 | 0.599223 | 0.549024 |
| M3 | **rs1789891** | 0.0352263 | 0.020275 | 1.737466 | 0.082305 |
| M3 | **rs3819197** | -0.0269004 | 0.017381 | -1.54766 | 0.121704 |
| M3 | **rs12311304** | -0.0040728 | 0.016143 | -0.25229 | 0.800819 |
| M3 | **rs2303317** | 0.0274776 | 0.015046 | 1.82622 | 0.067817 |
| M3 | **rs16985179** | -0.0241379 | 0.026064 | -0.92609 | 0.354399 |
| M3 | **rs9556711** | 0.0434426 | 0.031919 | 1.361048 | 0.173499 |
| M3 | **rs6716455** | -0.005886 | 0.021688 | -0.27139 | 0.786091 |
| M3 | **rs2188561** | -0.0182272 | 0.017803 | -1.02384 | 0.305912 |
| M3 | **rs9871864** | 0.0022395 | 0.015069 | 0.148619 | 0.881854 |
| M3 | **rs3738443** | -0.0044842 | 0.020022 | -0.22396 | 0.822785 |
| M3 | **rs768048** | 0.0035801 | 0.021562 | 0.166038 | 0.868127 |
| M3 | **rs13160562** | -0.0192511 | 0.015983 | -1.20446 | 0.22841 |
| M3 | **rs8062326** | 0.0052339 | 0.04384 | 0.119386 | 0.90497 |
| M3 | **rs10908907** | -0.0129894 | 0.017229 | -0.75394 | 0.450886 |
| M3 | **rs4293630** | -0.0112919 | 0.022047 | -0.51218 | 0.608528 |
| M3 | **rs6943555** | -0.009062 | 0.01715 | -0.5284 | 0.597223 |
| M3 | **rs2810114** | -0.0088115 | 0.016924 | -0.52064 | 0.602616 |
| M3 | **rs36563** | -0.0106535 | 0.021156 | -0.50357 | 0.614567 |
| M3 | **rs3762894** | -0.01111 | 0.020062 | -0.5538 | 0.579718 |
| M3 | **rs4770403** | -0.0093277 | 0.01911 | -0.48811 | 0.625472 |
| M3 | **rs9825310** | 0.0223999 | 0.015093 | 1.484109 | 0.13778 |
| M3 | **rs1353899** | -0.0150485 | 0.019202 | -0.7837 | 0.433214 |
| M3 | **rs2380220** | -0.0110925 | 0.020928 | -0.53003 | 0.59609 |
| M3 | **rs1000579** | 0.0100218 | 0.015151 | 0.661475 | 0.508308 |
| M3 | **rs2548145** | 0.0042942 | 0.015007 | 0.286148 | 0.774765 |
| M3 | **rs1573496** | 0.0215748 | 0.024812 | 0.86954 | 0.384552 |
| M3 | **rs10849915** | 0.0039319 | 0.015912 | 0.247105 | 0.804827 |
| M3 | **rs8040009** | -0.0137416 | 0.019555 | -0.70272 | 0.482227 |
| M3 | **rs3930234** | 0.0116226 | 0.020999 | 0.553471 | 0.579941 |
| M3 | **rs750338** | 0.0126761 | 0.018109 | 0.700006 | 0.483923 |
| M3 | **rs6902771** | 0.0249363 | 0.014855 | 1.678611 | 0.093228 |
| M3 | **rs7553212** | 0.0015753 | 0.015723 | 0.100195 | 0.92019 |
| M3 | **rs1229984** | -0.1690324 | 0.057346 | -2.94761 | 0.003202 |
| M3 | **rs4543123** | -0.0242022 | 0.01788 | -1.35356 | 0.175877 |
| M3 | **rs11851015** | -0.0169311 | 0.02187 | -0.77417 | 0.438832 |
| M3 | **rs4761097** | 0.0048995 | 0.015063 | 0.325279 | 0.74497 |
| M3 | **rs1793257** | 0.0167952 | 0.040139 | 0.418429 | 0.675634 |
| M3 | **rs10253361** | 0.0001514 | 0.014975 | 0.010113 | 0.991931 |
| M3 | **rs1497571** | 0.0272275 | 0.01493 | 1.823662 | 0.068203 |
| M3 | **rs804292** | 0.0321381 | 0.017498 | 1.836687 | 0.066256 |
| M3 | **rs36061340** | -0.0119556 | 0.031516 | -0.37935 | 0.70443 |
| M3 | **rs1380131** | 0.0331705 | 0.026119 | 1.269987 | 0.204089 |
| M3 | **rs13259667** | 0.0393532 | 0.028151 | 1.397915 | 0.162139 |
| M4 | **rs16985179** | 0.0155269 | 0.034791 | 0.446298 | 0.655382 |
| M4 | **rs2188561** | -0.0170004 | 0.023731 | -0.71637 | 0.473762 |
| M4 | **rs2154294** | -0.0143311 | 0.01988 | -0.7209 | 0.470971 |
| M4 | **rs9556711** | 0.0335881 | 0.042428 | 0.79165 | 0.428565 |
| M4 | **rs3131513** | 0.0370211 | 0.020537 | 1.802629 | 0.071447 |
| M4 | **rs1380131** | 0.0608499 | 0.034925 | 1.742306 | 0.081455 |
| M4 | **rs1353621** | -0.0097915 | 0.020669 | -0.47372 | 0.6357 |
| M4 | **rs1229984** | -0.1698636 | 0.077086 | -2.20355 | 0.027556 |
| M4 | **rs12388359** | -0.0469053 | 0.029853 | -1.5712 | 0.116136 |
| M4 | **rs237238** | -0.0689138 | 0.039964 | -1.72438 | 0.084639 |
| M4 | **rs1230165** | -0.0064224 | 0.02594 | -0.24758 | 0.804456 |
| M4 | **rs567926** | 0.0318693 | 0.020261 | 1.572977 | 0.115724 |
| M4 | **rs8040009** | -0.0393253 | 0.026122 | -1.50543 | 0.132213 |
| M4 | **rs2140418** | -0.0224556 | 0.025444 | -0.88256 | 0.377472 |
| M4 | **rs2380220** | -0.043061 | 0.028169 | -1.52866 | 0.126349 |
| M4 | **rs1344694** | 0.0152961 | 0.021393 | 0.715022 | 0.474596 |
| M4 | **rs3764435** | 0.0190331 | 0.020256 | 0.939644 | 0.3474 |
| M4 | **rs59972978** | 0.0369116 | 0.026663 | 1.384359 | 0.166249 |
| M4 | **rs6716455** | 0.0371093 | 0.028936 | 1.282457 | 0.199682 |
| M4 | **rs1497571** | 0.0336906 | 0.019971 | 1.686964 | 0.09161 |
| M4 | **rs1573496** | -0.0389796 | 0.033148 | -1.17591 | 0.239631 |
| M4 | **rs750338** | 0.0095157 | 0.024256 | 0.392299 | 0.694838 |
| M4 | **rs59677118** | 0.0006906 | 0.035398 | 0.01951 | 0.984434 |
| M4 | **rs2810114** | 0.0131959 | 0.022696 | 0.581415 | 0.560961 |
| M4 | **rs1109501** | 0.0155193 | 0.023206 | 0.668766 | 0.503645 |
| M4 | **rs9825310** | 0.0077081 | 0.020238 | 0.380881 | 0.703292 |
| M4 | **rs36563** | 0.0131946 | 0.028415 | 0.464352 | 0.642396 |
| M4 | **rs9871864** | 0.0289318 | 0.020252 | 1.428602 | 0.153119 |
| M4 | **rs7144649** | 0.0195594 | 0.023891 | 0.818693 | 0.412962 |
| M4 | **rs11724320** | -0.0379981 | 0.021397 | -1.77585 | 0.075758 |
| M4 | **rs1864982** | -0.0042026 | 0.030195 | -0.13918 | 0.889308 |
| M4 | **rs1318937** | 0.0476426 | 0.029832 | 1.597017 | 0.110262 |
| M4 | **rs4478858** | -0.0342762 | 0.020217 | -1.69546 | 0.089988 |
| M4 | **rs62202398** | 0.0225725 | 0.041606 | 0.542535 | 0.58745 |
| M4 | **rs8062326** | -0.0095783 | 0.058246 | -0.16445 | 0.86938 |
| M4 | **rs3738443** | 0.012077 | 0.026685 | 0.452581 | 0.65085 |
| M4 | **rs2827312** | 0.0275511 | 0.021962 | 1.254469 | 0.209672 |
| M4 | **rs1789891** | 0.0224092 | 0.02712 | 0.826301 | 0.408633 |
| M4 | **rs242938** | -0.0476087 | 0.041775 | -1.13964 | 0.254437 |
| M4 | **rs1876831** | -0.0630032 | 0.023627 | -2.66658 | 0.007663 |
| M4 | **rs804292** | 0.0118343 | 0.023521 | 0.503149 | 0.61486 |
| M4 | **rs4758317** | -0.0228422 | 0.020409 | -1.11925 | 0.263035 |
| M4 | **rs10908907** | -0.0246293 | 0.023045 | -1.06876 | 0.285179 |
| M4 | **rs2548145** | 0.0114444 | 0.020097 | 0.569466 | 0.56904 |
| M4 | **rs9512637** | 0.0018281 | 0.020714 | 0.088254 | 0.929675 |
| M4 | **rs4770403** | -0.0235641 | 0.025573 | -0.92145 | 0.356818 |
| M4 | **rs3762894** | 0.0127021 | 0.026702 | 0.47569 | 0.634295 |
| M4 | **rs768048** | 0.0172917 | 0.028943 | 0.597444 | 0.550211 |
| M4 | **rs2369955** | 0.0188653 | 0.03026 | 0.623447 | 0.532991 |
| M4 | **rs279861** | 0.0248463 | 0.020243 | 1.227385 | 0.219678 |
| M4 | **rs10849915** | 0.0124419 | 0.021214 | 0.586509 | 0.557533 |
| M4 | **rs1000579** | -0.0119948 | 0.020245 | -0.59249 | 0.553519 |
| M4 | **rs11851015** | -0.0129237 | 0.029313 | -0.44089 | 0.659294 |
| M4 | **rs67031482** | 0.0197903 | 0.020264 | 0.976608 | 0.328763 |
| M4 | **rs10253361** | 0.0223973 | 0.020013 | 1.119148 | 0.263077 |
| M4 | **rs420817** | -0.0078845 | 0.020011 | -0.394 | 0.693581 |
| M4 | **rs7553212** | -0.0015557 | 0.021031 | -0.07397 | 0.941033 |
| M4 | **rs12472151** | 0.043783 | 0.049244 | 0.889107 | 0.373946 |
| M4 | **rs195204** | 0.0151434 | 0.023176 | 0.653407 | 0.513494 |
| M4 | **rs1800759** | 0.0044545 | 0.020522 | 0.217057 | 0.828164 |
| M4 | **rs886205** | -0.0108116 | 0.026245 | -0.41195 | 0.680373 |
| M4 | **rs4761097** | 0.0083485 | 0.020202 | 0.413255 | 0.67942 |
| M4 | **rs642899** | 0.0186504 | 0.023833 | 0.782543 | 0.433896 |
| M4 | **rs284786** | 0.0115413 | 0.021825 | 0.528814 | 0.596934 |
| M4 | **rs13259667** | 0.0047164 | 0.03777 | 0.124873 | 0.900624 |
| M4 | **rs933769** | -0.0211399 | 0.025748 | -0.82102 | 0.411635 |
| M4 | **rs4440177** | -0.0085488 | 0.021134 | -0.4045 | 0.685847 |
| M4 | **rs1042026** | -0.0058241 | 0.021767 | -0.26757 | 0.78903 |
| M4 | **rs10893366** | 0.0141629 | 0.027294 | 0.518894 | 0.603835 |
| M4 | **rs3819197** | 0.0062942 | 0.023116 | 0.272283 | 0.785405 |
| M4 | **rs1824024** | 0.0225041 | 0.021703 | 1.0369 | 0.299782 |
| M4 | **rs1353899** | -0.0337626 | 0.025699 | -1.31376 | 0.188928 |
| M4 | **rs7590720** | 0.0148331 | 0.022017 | 0.673714 | 0.500493 |
| M4 | **rs12311304** | -0.0181232 | 0.021614 | -0.83849 | 0.401758 |
| M4 | **rs36061340** | 0.0047952 | 0.041791 | 0.114742 | 0.90865 |
| M4 | **rs3930234** | 0.0059839 | 0.028071 | 0.213173 | 0.831192 |
| M4 | **rs4543123** | -0.0054175 | 0.02398 | -0.22592 | 0.821267 |
| M4 | **rs1908556** | 0.0256053 | 0.028776 | 0.889814 | 0.373566 |
| M4 | **rs1793257** | 0.0076505 | 0.053752 | 0.14233 | 0.886819 |
| M4 | **rs2228093** | 0.000487 | 0.02938 | 0.016577 | 0.986775 |
| M4 | **rs9656709** | 0.0194176 | 0.020249 | 0.958933 | 0.337593 |
| M4 | **rs6943555** | -0.0144401 | 0.022831 | -0.63249 | 0.527065 |
| M4 | **rs2100290** | -0.0349924 | 0.019986 | -1.75083 | 0.079975 |
| M4 | **rs2303317** | 0.0071967 | 0.02008 | 0.358398 | 0.720045 |
| M4 | **rs4293630** | 0.0021793 | 0.02944 | 0.074024 | 0.940992 |
| M4 | **rs13160562** | -0.0091961 | 0.021386 | -0.43002 | 0.667184 |
| M4 | **rs6701037** | 0.004522 | 0.020096 | 0.225019 | 0.821965 |
| M4 | **rs9636231** | 0.0085756 | 0.022417 | 0.382551 | 0.702053 |
| M4 | **rs6902771** | 0.023126 | 0.019846 | 1.165289 | 0.243902 |
| M5 | **rs750338** | 0.0159443 | 0.020067 | 0.794541 | 0.426881 |
| M5 | **rs567926** | 0.005806 | 0.01689 | 0.343753 | 0.731032 |
| M5 | **rs1824024** | 0.0176891 | 0.018021 | 0.9816 | 0.326297 |
| M5 | **rs3738443** | -0.0089206 | 0.022214 | -0.40157 | 0.688002 |
| M5 | **rs11851015** | -0.0134859 | 0.024456 | -0.55144 | 0.581333 |
| M5 | **rs9512637** | -0.0043466 | 0.017175 | -0.25308 | 0.800205 |
| M5 | **rs2303317** | 0.013645 | 0.016831 | 0.810694 | 0.417542 |
| M5 | **rs933769** | -0.0049633 | 0.021704 | -0.22869 | 0.819112 |
| M5 | **rs8062326** | -0.0358764 | 0.04776 | -0.75118 | 0.452542 |
| M5 | **rs3764435** | 0.0210138 | 0.017001 | 1.236002 | 0.216458 |
| M5 | **rs16985179** | -0.0102278 | 0.029291 | -0.34918 | 0.726956 |
| M5 | **rs2140418** | 0.0277787 | 0.02126 | 1.306601 | 0.191348 |
| M5 | **rs1497571** | 0.0311648 | 0.016839 | 1.850787 | 0.0642 |
| M5 | **rs13160562** | -0.0325884 | 0.017745 | -1.83648 | 0.066287 |
| M5 | **rs7590720** | -0.0040404 | 0.018544 | -0.21788 | 0.827523 |
| M5 | **rs3762894** | -0.0268817 | 0.022205 | -1.21062 | 0.226042 |
| M5 | **rs804292** | -0.0161058 | 0.019683 | -0.81826 | 0.413211 |
| M5 | **rs3930234** | 0.0137756 | 0.023315 | 0.590855 | 0.554617 |
| M5 | **rs886205** | 0.007797 | 0.021922 | 0.355676 | 0.722083 |
| M5 | **rs4293630** | -0.0146827 | 0.024429 | -0.60103 | 0.547821 |
| M5 | **rs2380220** | 0.0006996 | 0.023412 | 0.029883 | 0.976161 |
| M5 | **rs7144649** | 0.0266623 | 0.020049 | 1.32989 | 0.183555 |
| M5 | **rs6943555** | -0.012133 | 0.019271 | -0.6296 | 0.528954 |
| M5 | **rs2369955** | 0.0335148 | 0.02495 | 1.343263 | 0.179187 |
| M5 | **rs62202398** | -0.0015791 | 0.034338 | -0.04599 | 0.96332 |
| M5 | **rs1353899** | -0.0158741 | 0.021224 | -0.74794 | 0.454496 |
| M5 | **rs6902771** | 0.0345673 | 0.016586 | 2.084141 | 0.037147 |
| M5 | **rs1318937** | 0.064909 | 0.024328 | 2.66803 | 0.00763 |
| M5 | **rs59972978** | 0.0468567 | 0.022127 | 2.11764 | 0.034206 |
| M5 | **rs2188561** | 0.0020649 | 0.019814 | 0.104219 | 0.916995 |
| M5 | **rs6701037** | -0.0097675 | 0.016762 | -0.58271 | 0.560086 |
| M5 | **rs12311304** | -0.0123633 | 0.018079 | -0.68384 | 0.494078 |
| M5 | **rs1800759** | 0.0197837 | 0.017008 | 1.163209 | 0.244745 |
| M5 | **rs420817** | -0.0117812 | 0.016709 | -0.70507 | 0.480764 |
| M5 | **rs1876831** | -0.0058721 | 0.019768 | -0.29706 | 0.766422 |
| M5 | **rs4761097** | -0.0020778 | 0.016818 | -0.12355 | 0.901673 |
| M5 | **rs6716455** | 0.0288014 | 0.023926 | 1.203784 | 0.228673 |
| M5 | **rs1344694** | -0.0073815 | 0.018019 | -0.40964 | 0.682068 |
| M5 | **rs195204** | -0.0050444 | 0.019307 | -0.26127 | 0.793888 |
| M5 | **rs1864982** | 0.0193368 | 0.025069 | 0.771361 | 0.440493 |
| M5 | **rs4543123** | -0.0051407 | 0.019873 | -0.25868 | 0.795884 |
| M5 | **rs1000579** | -0.0023535 | 0.016839 | -0.13976 | 0.888847 |
| M5 | **rs10893366** | 0.0125117 | 0.02272 | 0.550683 | 0.581851 |
| M5 | **rs7553212** | -0.0016876 | 0.017564 | -0.09608 | 0.923457 |
| M5 | **rs284786** | 0.0002054 | 0.018261 | 0.011249 | 0.991025 |
| M5 | **rs3131513** | 0.0108801 | 0.017078 | 0.637095 | 0.524063 |
| M5 | **rs36563** | 0.0222834 | 0.023407 | 0.952002 | 0.341096 |
| M5 | **rs8040009** | 0.0006672 | 0.021926 | 0.030431 | 0.975723 |
| M5 | **rs1573496** | -0.0158101 | 0.027891 | -0.56686 | 0.570807 |
| M5 | **rs2154294** | 0.0047841 | 0.0167 | 0.286473 | 0.774516 |
| M5 | **rs768048** | 0.0444645 | 0.024244 | 1.834013 | 0.066652 |
| M5 | **rs4478858** | 0.0037131 | 0.016788 | 0.221172 | 0.824959 |
| M5 | **rs13259667** | -0.0629177 | 0.031496 | -1.99766 | 0.045754 |
| M5 | **rs67031482** | 0.002367 | 0.016885 | 0.14018 | 0.888518 |
| M5 | **rs9556711** | -0.0283512 | 0.036092 | -0.78553 | 0.432142 |
| M5 | **rs2100290** | 0.0007583 | 0.0167 | 0.04541 | 0.963781 |
| M5 | **rs59677118** | -0.0004149 | 0.029277 | -0.01417 | 0.988692 |
| M5 | **rs1109501** | -0.0279546 | 0.01927 | -1.45069 | 0.146866 |
| M5 | **rs11724320** | 0.0015952 | 0.017702 | 0.090112 | 0.928198 |
| M5 | **rs10849915** | 0.0300085 | 0.017708 | 1.694595 | 0.090152 |
| M5 | **rs9871864** | 0.0167428 | 0.016909 | 0.99018 | 0.322086 |
| M5 | **rs4440177** | -0.0036953 | 0.017569 | -0.21033 | 0.833414 |
| M5 | **rs10253361** | -0.0006057 | 0.016687 | -0.0363 | 0.971046 |
| M5 | **rs242938** | -0.0112443 | 0.034882 | -0.32236 | 0.747183 |
| M5 | **rs4770403** | -0.0346536 | 0.021602 | -1.60421 | 0.108668 |
| M5 | **rs1353621** | 0.0048615 | 0.017281 | 0.281326 | 0.77846 |
| M5 | **rs12472151** | 0.0207658 | 0.039832 | 0.52133 | 0.602137 |
| M5 | **rs12388359** | 0.0257317 | 0.024635 | 1.044502 | 0.296253 |
| M5 | **rs1042026** | -0.0216525 | 0.018332 | -1.18116 | 0.237541 |
| M5 | **rs2228093** | 0.0001929 | 0.024611 | 0.007837 | 0.993747 |
| M5 | **rs279861** | 0.0015415 | 0.016898 | 0.091222 | 0.927316 |
| M5 | **rs237238** | -0.017023 | 0.033374 | -0.51007 | 0.610001 |
| M5 | **rs2827312** | 0.0109023 | 0.01835 | 0.594126 | 0.552428 |
| M5 | **rs36061340** | 0.0161151 | 0.035106 | 0.459047 | 0.6462 |
| M5 | **rs642899** | 0.0268644 | 0.019831 | 1.354687 | 0.175517 |
| M5 | **rs2548145** | 0.0069447 | 0.016757 | 0.414429 | 0.67856 |
| M5 | **rs3819197** | -0.0106172 | 0.01938 | -0.54783 | 0.583807 |
| M5 | **rs2810114** | 0.0048191 | 0.018783 | 0.256571 | 0.79751 |
| M5 | **rs1908556** | -0.0364547 | 0.024131 | -1.51072 | 0.13086 |
| M5 | **rs4758317** | 0.0115696 | 0.017065 | 0.677983 | 0.497783 |
| M5 | **rs10908907** | -0.0129848 | 0.019125 | -0.67896 | 0.497162 |
| M5 | **rs1793257** | -0.021845 | 0.04507 | -0.48469 | 0.627897 |
| M5 | **rs9656709** | 0.0260362 | 0.016997 | 1.531784 | 0.125576 |
| M5 | **rs9636231** | 0.0068792 | 0.018717 | 0.367541 | 0.713216 |
| M5 | **rs1380131** | 0.034843 | 0.028577 | 1.219289 | 0.222734 |
| M5 | **rs1789891** | 0.0446901 | 0.022753 | 1.964158 | 0.049512 |
| M5 | **rs1230165** | 0.022343 | 0.021546 | 1.037007 | 0.299733 |
| M5 | **rs1229984** | -0.0777296 | 0.064266 | -1.20949 | 0.226475 |
| M5 | **rs9825310** | 0.0032523 | 0.016875 | 0.192736 | 0.847166 |
| M7 | **rs1109501** | 0.0077944 | 0.024307 | 0.320667 | 0.748463 |
| M7 | **rs59677118** | 0.0101877 | 0.036702 | 0.277577 | 0.781337 |
| M7 | **rs242938** | 0.0069476 | 0.043873 | 0.158355 | 0.874177 |
| M7 | **rs9636231** | -0.0213315 | 0.023527 | -0.90668 | 0.364576 |
| M7 | **rs11851015** | -0.0379975 | 0.030884 | -1.23034 | 0.218572 |
| M7 | **rs1230165** | 0.0363844 | 0.027289 | 1.333322 | 0.182426 |
| M7 | **rs1497571** | 0.0466875 | 0.021189 | 2.203404 | 0.027566 |
| M7 | **rs1229984** | -0.2347168 | 0.080928 | -2.90033 | 0.003728 |
| M7 | **rs7590720** | -0.0112918 | 0.023339 | -0.48382 | 0.628516 |
| M7 | **rs16985179** | -0.0017721 | 0.036893 | -0.04803 | 0.961689 |
| M7 | **rs768048** | 0.0148827 | 0.030651 | 0.485559 | 0.62728 |
| M7 | **rs3764435** | -0.0036656 | 0.021336 | -0.1718 | 0.863595 |
| M7 | **rs279861** | 0.0337599 | 0.021282 | 1.586333 | 0.112664 |
| M7 | **rs1824024** | 0.0421035 | 0.022861 | 1.841711 | 0.065518 |
| M7 | **rs10908907** | -0.031273 | 0.024167 | -1.29406 | 0.195646 |
| M7 | **rs1800759** | 0.0443757 | 0.021417 | 2.072004 | 0.038265 |
| M7 | **rs9512637** | -0.0280311 | 0.021787 | -1.28659 | 0.198236 |
| M7 | **rs1000579** | -0.0233739 | 0.021334 | -1.09561 | 0.273249 |
| M7 | **rs567926** | 0.0337264 | 0.021332 | 1.581054 | 0.113866 |
| M7 | **rs420817** | -0.0181944 | 0.021087 | -0.86283 | 0.388229 |
| M7 | **rs10849915** | -0.00062 | 0.022416 | -0.02766 | 0.977933 |
| M7 | **rs750338** | 0.0410508 | 0.025427 | 1.614454 | 0.106429 |
| M7 | **rs2100290** | -0.0033831 | 0.021029 | -0.16088 | 0.872186 |
| M7 | **rs4758317** | -0.018808 | 0.021552 | -0.87269 | 0.382834 |
| M7 | **rs10893366** | 0.0184053 | 0.02867 | 0.641982 | 0.520885 |
| M7 | **rs2303317** | -0.0063106 | 0.021214 | -0.29748 | 0.766102 |
| M7 | **rs12472151** | -0.0025884 | 0.051255 | -0.0505 | 0.959723 |
| M7 | **rs6902771** | 0.0008305 | 0.021038 | 0.039477 | 0.96851 |
| M7 | **rs2548145** | 0.0028629 | 0.021139 | 0.13543 | 0.892272 |
| M7 | **rs36061340** | -0.0501409 | 0.044489 | -1.12703 | 0.259729 |
| M7 | **rs6701037** | -0.0179253 | 0.021123 | -0.8486 | 0.396104 |
| M7 | **rs3131513** | 0.0140613 | 0.021649 | 0.649503 | 0.516014 |
| M7 | **rs67031482** | 0.0046958 | 0.021368 | 0.219762 | 0.826057 |
| M7 | **rs2369955** | 0.0394993 | 0.031555 | 1.251759 | 0.210658 |
| M7 | **rs6943555** | -0.0143471 | 0.024356 | -0.58907 | 0.555815 |
| M7 | **rs7553212** | -0.0066707 | 0.022324 | -0.29882 | 0.765079 |
| M7 | **rs1353899** | -0.0338931 | 0.027058 | -1.25259 | 0.210356 |
| M7 | **rs2380220** | 0.0028906 | 0.029581 | 0.097718 | 0.922156 |
| M7 | **rs9656709** | 0.0267626 | 0.021394 | 1.250966 | 0.210947 |
| M7 | **rs3930234** | 0.0320362 | 0.029459 | 1.087487 | 0.276822 |
| M7 | **rs9825310** | 0.0199177 | 0.02115 | 0.941739 | 0.346326 |
| M7 | **rs2188561** | 0.0204591 | 0.024984 | 0.818902 | 0.412842 |
| M7 | **rs1344694** | -0.0222989 | 0.02264 | -0.98492 | 0.324664 |
| M7 | **rs4293630** | -0.0164854 | 0.031184 | -0.52866 | 0.597044 |
| M7 | **rs1908556** | 0.0143559 | 0.030605 | 0.469078 | 0.639014 |
| M7 | **rs2228093** | -0.0462508 | 0.031068 | -1.48872 | 0.136562 |
| M7 | **rs195204** | 0.0059806 | 0.024381 | 0.245294 | 0.806229 |
| M7 | **rs1793257** | -0.0620022 | 0.055988 | -1.10743 | 0.268108 |
| M7 | **rs2154294** | 0.0012672 | 0.021024 | 0.060276 | 0.951936 |
| M7 | **rs4543123** | 0.0021678 | 0.025007 | 0.086687 | 0.93092 |
| M7 | **rs7144649** | 0.0049858 | 0.025383 | 0.196423 | 0.844279 |
| M7 | **rs284786** | -0.0293373 | 0.023071 | -1.27163 | 0.203505 |
| M7 | **rs3738443** | 0.0030927 | 0.028087 | 0.110112 | 0.91232 |
| M7 | **rs2810114** | 0.0092172 | 0.023641 | 0.389884 | 0.696623 |
| M7 | **rs1573496** | -0.0663365 | 0.0348 | -1.90622 | 0.056622 |
| M7 | **rs886205** | -0.0212927 | 0.027714 | -0.7683 | 0.442306 |
| M7 | **rs9556711** | 0.0037504 | 0.045219 | 0.082938 | 0.933901 |
| M7 | **rs1876831** | -0.0248282 | 0.024987 | -0.99366 | 0.32039 |
| M7 | **rs804292** | -0.0179043 | 0.024689 | -0.7252 | 0.468332 |
| M7 | **rs1380131** | 0.0314825 | 0.036636 | 0.859338 | 0.390154 |
| M7 | **rs642899** | 0.0224984 | 0.025028 | 0.898934 | 0.368688 |
| M7 | **rs6716455** | -0.0201465 | 0.03068 | -0.65667 | 0.511395 |
| M7 | **rs13160562** | -0.0161726 | 0.022365 | -0.72314 | 0.469597 |
| M7 | **rs11724320** | -0.0089433 | 0.022497 | -0.39753 | 0.690975 |
| M7 | **rs4478858** | -0.0328754 | 0.021083 | -1.55933 | 0.118918 |
| M7 | **rs3762894** | -0.0221368 | 0.028148 | -0.78644 | 0.431611 |
| M7 | **rs2827312** | 0.0131372 | 0.023075 | 0.569321 | 0.569138 |
| M7 | **rs59972978** | 0.0253707 | 0.027753 | 0.914168 | 0.360629 |
| M7 | **rs4761097** | 0.0261149 | 0.021117 | 1.236658 | 0.216214 |
| M7 | **rs13259667** | -0.0304554 | 0.039628 | -0.76852 | 0.442177 |
| M7 | **rs2140418** | -0.0007509 | 0.026561 | -0.02827 | 0.977446 |
| M7 | **rs3819197** | 0.0107903 | 0.024358 | 0.442983 | 0.657778 |
| M7 | **rs36563** | 0.0157116 | 0.029357 | 0.535201 | 0.592511 |
| M7 | **rs9871864** | 0.0103098 | 0.021331 | 0.483332 | 0.62886 |
| M7 | **rs237238** | 0.0119433 | 0.04175 | 0.286066 | 0.774828 |
| M7 | **rs8040009** | -0.0354275 | 0.027785 | -1.27508 | 0.202283 |
| M7 | **rs12388359** | 0.014669 | 0.031418 | 0.4669 | 0.640572 |
| M7 | **rs4440177** | -0.011549 | 0.022311 | -0.51763 | 0.604716 |
| M7 | **rs4770403** | -0.0229992 | 0.027092 | -0.84893 | 0.395923 |
| M7 | **rs10253361** | 0.0137581 | 0.021086 | 0.652467 | 0.5141 |
| M7 | **rs62202398** | 0.0033551 | 0.043702 | 0.076772 | 0.938805 |
| M7 | **rs933769** | -0.0348032 | 0.027468 | -1.26703 | 0.205144 |
| M7 | **rs1789891** | 0.018978 | 0.028591 | 0.663781 | 0.50683 |
| M7 | **rs8062326** | -0.0503807 | 0.0595 | -0.84673 | 0.397143 |
| M7 | **rs1042026** | -0.0099565 | 0.022981 | -0.43325 | 0.664833 |
| M7 | **rs1318937** | 0.0484865 | 0.031118 | 1.558144 | 0.119199 |
| M7 | **rs1353621** | 0.0112463 | 0.021749 | 0.517094 | 0.605091 |
| M7 | **rs1864982** | -0.0202379 | 0.031816 | -0.63609 | 0.524716 |
| M7 | **rs12311304** | -0.0247741 | 0.022737 | -1.08959 | 0.275893 |
| M8 | **rs36061340** | 0.0545956 | 0.046323 | 1.178577 | 0.238567 |
| M8 | **rs2228093** | -0.0236987 | 0.032033 | -0.73982 | 0.459411 |
| M8 | **rs284786** | 0.0090298 | 0.024112 | 0.374503 | 0.708031 |
| M8 | **rs13259667** | -0.0505641 | 0.041209 | -1.22702 | 0.219815 |
| M8 | **rs12388359** | 0.0358505 | 0.032971 | 1.087323 | 0.276894 |
| M8 | **rs11724320** | -0.0273955 | 0.023415 | -1.17001 | 0.241995 |
| M8 | **rs10253361** | 0.0089228 | 0.021964 | 0.406255 | 0.684555 |
| M8 | **rs1353899** | -0.0753171 | 0.028402 | -2.65184 | 0.008005 |
| M8 | **rs9556711** | 0.024909 | 0.046911 | 0.530988 | 0.595427 |
| M8 | **rs9636231** | 0.0409874 | 0.024602 | 1.665991 | 0.095715 |
| M8 | **rs1497571** | 0.035282 | 0.022228 | 1.587291 | 0.112447 |
| M8 | **rs1230165** | 0.0276242 | 0.028429 | 0.971709 | 0.331195 |
| M8 | **rs237238** | -0.0246358 | 0.043275 | -0.56929 | 0.569162 |
| M8 | **rs1353621** | 0.0229211 | 0.022779 | 1.006243 | 0.314299 |
| M8 | **rs1824024** | 0.040357 | 0.023815 | 1.694587 | 0.090154 |
| M8 | **rs36563** | -0.0149059 | 0.030506 | -0.48862 | 0.625112 |
| M8 | **rs933769** | -0.0404132 | 0.028358 | -1.4251 | 0.154128 |
| M8 | **rs1229984** | -0.2304593 | 0.08489 | -2.71481 | 0.006631 |
| M8 | **rs4758317** | -0.030622 | 0.022461 | -1.36335 | 0.172771 |
| M8 | **rs6716455** | -0.0427952 | 0.031914 | -1.34094 | 0.179939 |
| M8 | **rs2827312** | 0.0379211 | 0.024153 | 1.570017 | 0.116411 |
| M8 | **rs1380131** | 0.0387106 | 0.038179 | 1.013933 | 0.310615 |
| M8 | **rs2154294** | 0.000285 | 0.021874 | 0.013029 | 0.989605 |
| M8 | **rs7553212** | 0.0084781 | 0.023178 | 0.36578 | 0.71453 |
| M8 | **rs7144649** | 0.0290919 | 0.026627 | 1.092556 | 0.274589 |
| M8 | **rs3131513** | 0.0281027 | 0.02269 | 1.238538 | 0.215517 |
| M8 | **rs2810114** | -0.0024561 | 0.02454 | -0.10008 | 0.920278 |
| M8 | **rs6902771** | 0.0188811 | 0.022069 | 0.855567 | 0.392237 |
| M8 | **rs10893366** | 0.0149952 | 0.030074 | 0.49861 | 0.618055 |
| M8 | **rs279861** | 0.0230499 | 0.022136 | 1.04127 | 0.29775 |
| M8 | **rs4478858** | -0.0608607 | 0.022014 | -2.76461 | 0.005699 |
| M8 | **rs2369955** | 0.0002208 | 0.033013 | 0.00669 | 0.994662 |
| M8 | **rs10908907** | -0.0338759 | 0.02518 | -1.34533 | 0.17852 |
| M8 | **rs1789891** | 0.0400478 | 0.029754 | 1.345968 | 0.178313 |
| M8 | **rs1000579** | -0.0262153 | 0.022191 | -1.18133 | 0.237473 |
| M8 | **rs4440177** | -0.0181274 | 0.023095 | -0.7849 | 0.43251 |
| M8 | **rs59677118** | 0.0247167 | 0.038357 | 0.644384 | 0.519326 |
| M8 | **rs1573496** | -0.0802535 | 0.036021 | -2.22794 | 0.025885 |
| M8 | **rs9656709** | 0.0110603 | 0.022295 | 0.496099 | 0.619825 |
| M8 | **rs420817** | -0.0175383 | 0.022008 | -0.7969 | 0.42551 |
| M8 | **rs1864982** | 0.0391756 | 0.033028 | 1.186129 | 0.235571 |
| M8 | **rs12311304** | -0.0368212 | 0.02379 | -1.54776 | 0.12168 |
| M8 | **rs3762894** | -0.0098895 | 0.029585 | -0.33427 | 0.738173 |
| M8 | **rs750338** | 0.0316001 | 0.026692 | 1.183882 | 0.23646 |
| M8 | **rs2100290** | -0.0085776 | 0.022025 | -0.38945 | 0.696943 |
| M8 | **rs6701037** | -0.0109349 | 0.021966 | -0.49782 | 0.618611 |
| M8 | **rs1800759** | 0.0181049 | 0.022377 | 0.80908 | 0.418469 |
| M8 | **rs13160562** | 0.0004461 | 0.023428 | 0.019042 | 0.984808 |
| M8 | **rs3930234** | 0.0081228 | 0.030702 | 0.264567 | 0.791343 |
| M8 | **rs8062326** | -0.0195638 | 0.061069 | -0.32036 | 0.748698 |
| M8 | **rs9825310** | 0.0129428 | 0.022125 | 0.58499 | 0.558555 |
| M8 | **rs886205** | -0.0266819 | 0.028865 | -0.92438 | 0.355287 |
| M8 | **rs2303317** | 0.0385537 | 0.022103 | 1.744299 | 0.081107 |
| M8 | **rs9871864** | 0.018447 | 0.02223 | 0.829837 | 0.406631 |
| M8 | **rs804292** | 0.0308812 | 0.025658 | 1.20356 | 0.22876 |
| M8 | **rs2140418** | 0.0009361 | 0.027763 | 0.033716 | 0.973104 |
| M8 | **rs62202398** | -0.0334706 | 0.044895 | -0.74553 | 0.45595 |
| M8 | **rs642899** | 0.0463504 | 0.026161 | 1.771729 | 0.07644 |
| M8 | **rs2548145** | -0.012561 | 0.022032 | -0.57013 | 0.568588 |
| M8 | **rs3764435** | 0.0427201 | 0.022191 | 1.925089 | 0.054218 |
| M8 | **rs1793257** | -0.0499646 | 0.057847 | -0.86374 | 0.387729 |
| M8 | **rs4761097** | 0.0122348 | 0.022097 | 0.553686 | 0.579794 |
| M8 | **rs2188561** | -0.0276461 | 0.026137 | -1.05774 | 0.290176 |
| M8 | **rs12472151** | 0.0290788 | 0.053032 | 0.548323 | 0.58347 |
| M8 | **rs16985179** | -0.0056345 | 0.03817 | -0.14762 | 0.882645 |
| M8 | **rs1344694** | -0.0163598 | 0.023653 | -0.69166 | 0.48915 |
| M8 | **rs8040009** | -0.0085736 | 0.029009 | -0.29555 | 0.767573 |
| M8 | **rs67031482** | 0.0129828 | 0.022382 | 0.580044 | 0.561885 |
| M8 | **rs1109501** | -0.0199267 | 0.025471 | -0.78232 | 0.434029 |
| M8 | **rs768048** | 0.0447317 | 0.031864 | 1.403821 | 0.160372 |
| M8 | **rs195204** | 0.0034577 | 0.025527 | 0.135455 | 0.892252 |
| M8 | **rs3738443** | 0.0135767 | 0.029401 | 0.461782 | 0.644238 |
| M8 | **rs6943555** | -0.0328742 | 0.025394 | -1.29458 | 0.195464 |
| M8 | **rs1908556** | -0.0106407 | 0.032127 | -0.33121 | 0.740487 |
| M8 | **rs567926** | 0.0290492 | 0.022203 | 1.30834 | 0.190758 |
| M8 | **rs2380220** | -0.0481129 | 0.030909 | -1.55658 | 0.11957 |
| M8 | **rs7590720** | -0.0111246 | 0.024392 | -0.45607 | 0.648337 |
| M8 | **rs59972978** | -0.0138174 | 0.028963 | -0.47707 | 0.633312 |
| M8 | **rs3819197** | 0.0012253 | 0.025747 | 0.047591 | 0.962043 |
| M8 | **rs242938** | 0.0100382 | 0.045962 | 0.218404 | 0.827115 |
| M8 | **rs4293630** | -0.0302634 | 0.032551 | -0.92972 | 0.352516 |
| M8 | **rs4543123** | -0.0150858 | 0.026141 | -0.57709 | 0.563882 |
| M8 | **rs1042026** | -0.0155543 | 0.024185 | -0.64313 | 0.52014 |
| M8 | **rs11851015** | -0.0227909 | 0.032224 | -0.70727 | 0.4794 |
| M8 | **rs10849915** | 0.0167461 | 0.023438 | 0.714476 | 0.474933 |
| M8 | **rs4770403** | -0.0084309 | 0.028271 | -0.29822 | 0.765535 |
| M8 | **rs1318937** | 0.0373497 | 0.032428 | 1.151763 | 0.249419 |
| M8 | **rs1876831** | -0.0255087 | 0.025989 | -0.98152 | 0.326338 |
| M8 | **rs9512637** | -0.0204464 | 0.022764 | -0.89819 | 0.369086 |
| M12 | **rs59677118** | 0.0239787 | 0.036577 | 0.655565 | 0.512104 |
| M12 | **rs2100290** | -0.0275299 | 0.021241 | -1.29607 | 0.19495 |
| M12 | **rs4293630** | -0.0512761 | 0.031658 | -1.61968 | 0.1053 |
| M12 | **rs3762894** | 0.021982 | 0.028285 | 0.777171 | 0.437058 |
| M12 | **rs2827312** | 0.0046002 | 0.023309 | 0.197356 | 0.843549 |
| M12 | **rs1344694** | -0.0251792 | 0.022699 | -1.10926 | 0.267318 |
| M12 | **rs3131513** | 0.0287151 | 0.02182 | 1.315994 | 0.188176 |
| M12 | **rs13259667** | -0.0572049 | 0.03985 | -1.43551 | 0.151142 |
| M12 | **rs62202398** | -0.0149968 | 0.043159 | -0.34748 | 0.728233 |
| M12 | **rs59972978** | -0.015329 | 0.027768 | -0.55204 | 0.580922 |
| M12 | **rs6902771** | 0.0134028 | 0.021083 | 0.635728 | 0.524954 |
| M12 | **rs1789891** | 0.0623588 | 0.028567 | 2.182875 | 0.029045 |
| M12 | **rs2380220** | 0.0155729 | 0.030144 | 0.51662 | 0.605421 |
| M12 | **rs2369955** | 0.0034115 | 0.032034 | 0.106496 | 0.915189 |
| M12 | **rs2810114** | -0.0227832 | 0.023614 | -0.96482 | 0.334636 |
| M12 | **rs237238** | -0.0297607 | 0.042066 | -0.70747 | 0.479272 |
| M12 | **rs9556711** | 0.0793064 | 0.045409 | 1.746507 | 0.080723 |
| M12 | **rs1353899** | -0.0296231 | 0.027374 | -1.08215 | 0.279185 |
| M12 | **rs6943555** | -0.0302753 | 0.02453 | -1.2342 | 0.217129 |
| M12 | **rs36563** | -0.0298552 | 0.029346 | -1.01734 | 0.308992 |
| M12 | **rs4478858** | -0.0277489 | 0.021256 | -1.30544 | 0.191744 |
| M12 | **rs7590720** | -0.0241856 | 0.023487 | -1.02976 | 0.303125 |
| M12 | **rs2140418** | -0.002276 | 0.026938 | -0.08449 | 0.932665 |
| M12 | **rs284786** | 0.0356881 | 0.023041 | 1.54892 | 0.121401 |
| M12 | **rs2154294** | -0.0241491 | 0.021038 | -1.14787 | 0.251022 |
| M12 | **rs1380131** | 0.0335049 | 0.036991 | 0.905767 | 0.365059 |
| M12 | **rs3764435** | 0.0193068 | 0.021396 | 0.902347 | 0.366873 |
| M12 | **rs36061340** | 0.0261276 | 0.044204 | 0.591065 | 0.554477 |
| M12 | **rs1318937** | -0.0148941 | 0.031139 | -0.47832 | 0.632424 |
| M12 | **rs3819197** | -0.004599 | 0.024553 | -0.18731 | 0.851417 |
| M12 | **rs67031482** | -0.0263913 | 0.021438 | -1.23105 | 0.218303 |
| M12 | **rs6701037** | 0.0244189 | 0.021245 | 1.149402 | 0.25039 |
| M12 | **rs9512637** | -0.0188524 | 0.021844 | -0.86306 | 0.388107 |
| M12 | **rs12388359** | 0.0236802 | 0.031914 | 0.741995 | 0.458091 |
| M12 | **rs1109501** | -0.0305518 | 0.02444 | -1.25009 | 0.211266 |
| M12 | **rs9656709** | 0.0025932 | 0.021637 | 0.119851 | 0.904601 |
| M12 | **rs1229984** | -0.3263028 | 0.084182 | -3.87618 | 0.000106 |
| M12 | **rs933769** | -0.0379202 | 0.027499 | -1.37898 | 0.1679 |
| M12 | **rs4543123** | -0.0182673 | 0.025408 | -0.71896 | 0.472164 |
| M12 | **rs1353621** | 0.0203058 | 0.021771 | 0.932693 | 0.350979 |
| M12 | **rs9871864** | 0.0181893 | 0.021404 | 0.84982 | 0.395425 |
| M12 | **rs16985179** | 0.0102071 | 0.036829 | 0.277148 | 0.781667 |
| M12 | **rs11724320** | -0.0014542 | 0.022676 | -0.06413 | 0.948869 |
| M12 | **rs750338** | 0.01127 | 0.025414 | 0.443452 | 0.657439 |
| M12 | **rs1864982** | 0.0287441 | 0.031676 | 0.907453 | 0.364167 |
| M12 | **rs4440177** | -0.0402904 | 0.022368 | -1.80128 | 0.071659 |
| M12 | **rs886205** | 0.0017514 | 0.027891 | 0.062794 | 0.949931 |
| M12 | **rs1000579** | -0.0050106 | 0.021502 | -0.23303 | 0.815741 |
| M12 | **rs4758317** | -0.0157579 | 0.021732 | -0.7251 | 0.468391 |
| M12 | **rs2303317** | 0.0044216 | 0.021163 | 0.208937 | 0.834497 |
| M12 | **rs1042026** | -0.0099841 | 0.023222 | -0.42994 | 0.66724 |
| M12 | **rs768048** | 0.0403036 | 0.030867 | 1.305716 | 0.191649 |
| M12 | **rs4761097** | 0.0364412 | 0.021379 | 1.704565 | 0.088276 |
| M12 | **rs3738443** | -0.003787 | 0.028433 | -0.13319 | 0.894044 |
| M12 | **rs13160562** | -0.0167253 | 0.022637 | -0.73886 | 0.45999 |
| M12 | **rs1573496** | -0.0773148 | 0.034767 | -2.22382 | 0.02616 |
| M12 | **rs6716455** | -0.0266898 | 0.030862 | -0.86481 | 0.387142 |
| M12 | **rs1497571** | 0.052529 | 0.021327 | 2.463086 | 0.013775 |
| M12 | **rs195204** | 0.0080287 | 0.024419 | 0.328783 | 0.74232 |
| M12 | **rs1824024** | 0.0184899 | 0.022945 | 0.805847 | 0.420331 |
| M12 | **rs7553212** | 0.0171398 | 0.022323 | 0.767796 | 0.442609 |
| M12 | **rs12472151** | -0.0513637 | 0.050272 | -1.02171 | 0.306916 |
| M12 | **rs279861** | 0.0354368 | 0.021164 | 1.674382 | 0.094056 |
| M12 | **rs12311304** | -0.0034733 | 0.023023 | -0.15086 | 0.880085 |
| M12 | **rs10253361** | 0.010109 | 0.021123 | 0.478586 | 0.632233 |
| M12 | **rs8062326** | 0.0581256 | 0.060364 | 0.962922 | 0.335587 |
| M12 | **rs2188561** | -0.0485014 | 0.025374 | -1.91148 | 0.055944 |
| M12 | **rs9825310** | 0.0031271 | 0.021391 | 0.146189 | 0.883772 |
| M12 | **rs9636231** | -0.0091959 | 0.023644 | -0.38893 | 0.697329 |
| M12 | **rs3930234** | 0.0008754 | 0.030029 | 0.029152 | 0.976744 |
| M12 | **rs8040009** | -0.0416768 | 0.027662 | -1.50663 | 0.131907 |
| M12 | **rs420817** | -0.005726 | 0.021163 | -0.27057 | 0.786723 |
| M12 | **rs804292** | 0.0035943 | 0.024707 | 0.145478 | 0.884334 |
| M12 | **rs7144649** | 0.008265 | 0.025423 | 0.325099 | 0.745107 |
| M12 | **rs567926** | 0.0412884 | 0.021246 | 1.943339 | 0.051975 |
| M12 | **rs10849915** | 0.0148039 | 0.022567 | 0.655999 | 0.511825 |
| M12 | **rs1230165** | 0.0525542 | 0.027195 | 1.932488 | 0.053299 |
| M12 | **rs1876831** | -0.0051859 | 0.024875 | -0.20848 | 0.834855 |
| M12 | **rs4770403** | 0.0032872 | 0.027274 | 0.120524 | 0.904068 |
| M12 | **rs242938** | -0.0587295 | 0.043945 | -1.33643 | 0.181409 |
| M12 | **rs10908907** | -0.0123604 | 0.024531 | -0.50388 | 0.614347 |
| M12 | **rs10893366** | 0.0222173 | 0.028536 | 0.77856 | 0.436239 |
| M12 | **rs2548145** | 0.0115405 | 0.021267 | 0.542645 | 0.587374 |
| M12 | **rs642899** | 0.0532757 | 0.02493 | 2.136982 | 0.032599 |
| M12 | **rs1908556** | -0.0060649 | 0.030663 | -0.19779 | 0.843206 |
| M12 | **rs1800759** | 0.0161973 | 0.021452 | 0.755047 | 0.450221 |
| M12 | **rs1793257** | -0.0458412 | 0.055737 | -0.82246 | 0.410814 |
| M12 | **rs11851015** | -0.0348237 | 0.030936 | -1.12568 | 0.2603 |
| M12 | **rs2228093** | -0.0406312 | 0.031415 | -1.29336 | 0.195889 |
| M18 | **rs8062326** | -0.0508528 | 0.069007 | -0.73692 | 0.461169 |
| M18 | **rs1353621** | 0.0512407 | 0.025274 | 2.027437 | 0.042618 |
| M18 | **rs7144649** | 0.033034 | 0.029438 | 1.122147 | 0.2618 |
| M18 | **rs1573496** | 0.004438 | 0.040278 | 0.110185 | 0.912262 |
| M18 | **rs2303317** | -0.004718 | 0.024492 | -0.19264 | 0.847244 |
| M18 | **rs1344694** | -0.0659334 | 0.026251 | -2.51168 | 0.012016 |
| M18 | **rs7590720** | -0.0744784 | 0.027428 | -2.71541 | 0.006619 |
| M18 | **rs4293630** | -0.051985 | 0.036145 | -1.43822 | 0.150371 |
| M18 | **rs1380131** | 0.0328773 | 0.042654 | 0.770796 | 0.440828 |
| M18 | **rs242938** | -0.0067406 | 0.051567 | -0.13072 | 0.896 |
| M18 | **rs4440177** | -0.0181716 | 0.025917 | -0.70114 | 0.483218 |
| M18 | **rs2188561** | -0.0217277 | 0.029171 | -0.74485 | 0.456364 |
| M18 | **rs1800759** | 0.018944 | 0.025194 | 0.751929 | 0.452094 |
| M18 | **rs2380220** | 0.0256305 | 0.034109 | 0.751427 | 0.452396 |
| M18 | **rs3930234** | 0.0835334 | 0.034156 | 2.445657 | 0.014459 |
| M18 | **rs4761097** | -0.0428481 | 0.024439 | -1.75328 | 0.079555 |
| M18 | **rs9825310** | 0.0360953 | 0.024784 | 1.456398 | 0.145283 |
| M18 | **rs4478858** | -0.035459 | 0.024425 | -1.45177 | 0.146565 |
| M18 | **rs768048** | -0.046044 | 0.03499 | -1.3159 | 0.188207 |
| M18 | **rs6701037** | -0.0227371 | 0.024691 | -0.92085 | 0.357127 |
| M18 | **rs2100290** | -0.0198086 | 0.024714 | -0.8015 | 0.422842 |
| M18 | **rs3131513** | 0.0206911 | 0.025227 | 0.82021 | 0.412096 |
| M18 | **rs4543123** | -0.0009221 | 0.029606 | -0.03115 | 0.975152 |
| M18 | **rs886205** | 0.0009728 | 0.031941 | 0.030458 | 0.975702 |
| M18 | **rs1497571** | 0.0414373 | 0.024524 | 1.689677 | 0.09109 |
| M18 | **rs12388359** | -0.0185605 | 0.036824 | -0.50404 | 0.614234 |
| M18 | **rs9512637** | -0.0502659 | 0.025699 | -1.95599 | 0.050467 |
| M18 | **rs933769** | -0.0018409 | 0.032257 | -0.05707 | 0.954489 |
| M18 | **rs1230165** | 0.0668707 | 0.031668 | 2.111647 | 0.034717 |
| M18 | **rs2810114** | -0.0457806 | 0.027255 | -1.67974 | 0.093008 |
| M18 | **rs6902771** | -0.0098815 | 0.024283 | -0.40694 | 0.684054 |
| M18 | **rs6716455** | -0.0481117 | 0.036144 | -1.33112 | 0.183149 |
| M18 | **rs3819197** | 0.0064939 | 0.028913 | 0.224604 | 0.822288 |
| M18 | **rs10908907** | -0.0075689 | 0.028398 | -0.26653 | 0.789828 |
| M18 | **rs1229984** | -0.2768306 | 0.096431 | -2.87075 | 0.004095 |
| M18 | **rs9636231** | -0.0038629 | 0.027333 | -0.14133 | 0.887613 |
| M18 | **rs1864982** | 0.0252951 | 0.03637 | 0.695498 | 0.486743 |
| M18 | **rs36563** | -0.0417065 | 0.033793 | -1.23417 | 0.217139 |
| M18 | **rs284786** | 0.0332841 | 0.026728 | 1.245284 | 0.213027 |
| M18 | **rs7553212** | -0.007298 | 0.02575 | -0.28342 | 0.776858 |
| M18 | **rs1876831** | -0.0024402 | 0.028582 | -0.08537 | 0.931965 |
| M18 | **rs1109501** | -0.0325584 | 0.027945 | -1.16511 | 0.243975 |
| M18 | **rs2228093** | -0.0410008 | 0.036458 | -1.1246 | 0.26076 |
| M18 | **rs2154294** | -0.0120011 | 0.024391 | -0.49202 | 0.622704 |
| M18 | **rs279861** | 0.0278835 | 0.024536 | 1.136418 | 0.255782 |
| M18 | **rs8040009** | -0.0163433 | 0.032186 | -0.50777 | 0.611611 |
| M18 | **rs59972978** | -0.0009814 | 0.032738 | -0.02998 | 0.976085 |
| M18 | **rs1318937** | 0.0473313 | 0.036121 | 1.310365 | 0.190073 |
| M18 | **rs13259667** | -0.0368515 | 0.045844 | -0.80385 | 0.421486 |
| M18 | **rs1789891** | 0.0211757 | 0.032735 | 0.646883 | 0.517708 |
| M18 | **rs10849915** | 0.0056753 | 0.026069 | 0.217703 | 0.82766 |
| M18 | **rs6943555** | -0.0292074 | 0.028225 | -1.0348 | 0.300763 |
| M18 | **rs804292** | 0.0152688 | 0.028867 | 0.528933 | 0.596852 |
| M18 | **rs67031482** | 0.0203112 | 0.024874 | 0.816552 | 0.414184 |
| M18 | **rs12311304** | -0.0390255 | 0.026506 | -1.47231 | 0.140938 |
| M18 | **rs4758317** | -0.0184995 | 0.024848 | -0.74451 | 0.456569 |
| M18 | **rs642899** | 0.0654676 | 0.028907 | 2.264774 | 0.023527 |
| M18 | **rs11851015** | 0.0218276 | 0.035313 | 0.618114 | 0.5365 |
| M18 | **rs195204** | 0.0146097 | 0.028179 | 0.518462 | 0.604136 |
| M18 | **rs2140418** | -0.0483054 | 0.031017 | -1.5574 | 0.119377 |
| M18 | **rs1000579** | -0.0172086 | 0.024909 | -0.69086 | 0.489653 |
| M18 | **rs1793257** | 0.0226743 | 0.066172 | 0.342657 | 0.731857 |
| M18 | **rs237238** | 0.0011381 | 0.049233 | 0.023117 | 0.981557 |
| M18 | **rs9556711** | 0.0259633 | 0.052336 | 0.496088 | 0.619832 |
| M18 | **rs62202398** | 0.0449142 | 0.049085 | 0.915022 | 0.36018 |
| M18 | **rs1042026** | 0.0144927 | 0.027073 | 0.535329 | 0.592422 |
| M18 | **rs3764435** | -0.0004635 | 0.024641 | -0.01881 | 0.984993 |
| M18 | **rs10893366** | 0.0121528 | 0.033502 | 0.362746 | 0.716795 |
| M18 | **rs11724320** | -0.0376221 | 0.025889 | -1.45322 | 0.146162 |
| M18 | **rs420817** | 0.0319648 | 0.024674 | 1.295481 | 0.195154 |
| M18 | **rs13160562** | 0.0259279 | 0.026435 | 0.980839 | 0.326672 |
| M18 | **rs750338** | -0.006837 | 0.029905 | -0.22863 | 0.819159 |
| M18 | **rs59677118** | -0.0024606 | 0.042936 | -0.05731 | 0.9543 |
| M18 | **rs9656709** | 0.0500916 | 0.025074 | 1.997738 | 0.045745 |
| M18 | **rs36061340** | 0.0574424 | 0.05183 | 1.108293 | 0.267735 |
| M18 | **rs3738443** | -0.0128185 | 0.032565 | -0.39362 | 0.693859 |
| M18 | **rs10253361** | 0.0360245 | 0.024654 | 1.461188 | 0.143964 |
| M18 | **rs2369955** | 0.0700059 | 0.03724 | 1.87988 | 0.060124 |
| M18 | **rs1824024** | 0.0035363 | 0.02677 | 0.132098 | 0.894907 |
| M18 | **rs567926** | 0.0367371 | 0.024587 | 1.494178 | 0.135129 |
| M18 | **rs1908556** | -0.0316202 | 0.035535 | -0.88983 | 0.373555 |
| M18 | **rs4770403** | -0.0629912 | 0.032258 | -1.95271 | 0.050854 |
| M18 | **rs1353899** | -0.0361508 | 0.031653 | -1.14209 | 0.253416 |
| M18 | **rs9871864** | -0.0121864 | 0.024922 | -0.48898 | 0.624857 |
| M18 | **rs2548145** | -0.0111779 | 0.024708 | -0.4524 | 0.650984 |
| M18 | **rs2827312** | 0.0134311 | 0.027054 | 0.49646 | 0.61957 |
| M18 | **rs3762894** | -0.0017773 | 0.032548 | -0.05461 | 0.956453 |
| M18 | **rs12472151** | -0.0488042 | 0.059423 | -0.82131 | 0.41147 |
| M18 | **rs16985179** | 0.0222927 | 0.04287 | 0.520014 | 0.603054 |
