# Supplementary material for: Exploration of a Polygenic Risk Score for Alcohol Consumption: A Longitudinal Analysis from the ALSPAC Cohort
Source: PLoS One. 2016 Nov 30;11(11):e0167360. doi: 10.1371/journal.pone.0167360 (PMC5130278; doi:10.1371/journal.pone.0167360)
Supplement: S6 Table — (DOCX) [file pone.0167360.s006.docx]

**S6 Table: Associations between PGRS, individuals SNPs and potential confounders (Bonferroni corrected p-value = 0.00011)**

| **Confounder** | **SNP/Risk Score** | **OR/Beta** | **SE** | **p value** |
| --- | --- | --- | --- | --- |
| **Mothers** | | | | |
| amphetamine use at 2 years post baseline | Mums PGRS | 10.61968 | 18.13765 | 0.166549 |
|  | rs9512637 | 2.134412 | 0.54162 | 0.002809 |
|  | rs11724320 | 0.447043 | 0.145527 | 0.013391 |
|  | rs59972978 | 1.936256 | 0.554477 | 0.021033 |
|  | rs279861 | 0.584461 | 0.160203 | 0.050072 |
|  | rs567926 | 0.612216 | 0.167377 | 0.072696 |
|  | rs9556711 | 1.996924 | 0.805372 | 0.086374 |
|  | rs13160562 | 0.603247 | 0.186405 | 0.101907 |
|  | rs2228093 | 1.688002 | 0.544843 | 0.104799 |
|  | rs59677118 | 0.3023 | 0.225203 | 0.108297 |
|  | rs9825310 | 0.661167 | 0.174967 | 0.117939 |
|  | rs1864982 | 0.450288 | 0.234059 | 0.124794 |
|  | rs13259667 | 1.767941 | 0.680088 | 0.138532 |
|  | rs10253361 | 0.679419 | 0.179862 | 0.144277 |
|  | rs67031482 | 1.449608 | 0.374391 | 0.150543 |
|  | rs1344694 | 1.439028 | 0.373681 | 0.161028 |
|  | rs16985179 | 0.418566 | 0.263876 | 0.167133 |
|  | rs4761097 | 1.415219 | 0.359586 | 0.171687 |
|  | rs1824024 | 1.427221 | 0.375675 | 0.176552 |
|  | rs10893366 | 1.468083 | 0.450016 | 0.210357 |
|  | rs1908556 | 0.560098 | 0.260012 | 0.211804 |
|  | rs1789891 | 1.450175 | 0.444723 | 0.22551 |
|  | rs7553212 | 0.709845 | 0.205426 | 0.236325 |
|  | rs195204 | 0.67681 | 0.223983 | 0.238174 |
|  | rs2380220 | 0.603997 | 0.260599 | 0.242579 |
|  | rs36563 | 0.606966 | 0.260079 | 0.243932 |
|  | rs3764435 | 0.741597 | 0.19293 | 0.250508 |
|  | rs420817 | 1.337376 | 0.343432 | 0.257606 |
|  | rs12472151 | 0.323768 | 0.327808 | 0.265352 |
|  | rs9871864 | 1.329071 | 0.341008 | 0.267535 |
|  | rs3819197 | 0.695921 | 0.229915 | 0.272513 |
|  | rs2810114 | 0.712822 | 0.221907 | 0.276848 |
|  | rs804292 | 0.709294 | 0.230271 | 0.290045 |
|  | rs8040009 | 1.372338 | 0.419938 | 0.300969 |
|  | rs284786 | 0.737411 | 0.218734 | 0.304457 |
|  | rs4293630 | 1.410268 | 0.47223 | 0.304579 |
|  | rs7590720 | 1.30941 | 0.34871 | 0.311413 |
|  | rs3762894 | 0.670281 | 0.266859 | 0.314973 |
|  | rs12311304 | 0.75465 | 0.218993 | 0.332021 |
|  | rs642899 | 0.726817 | 0.241266 | 0.336434 |
|  | rs7144649 | 0.733657 | 0.245226 | 0.354141 |
|  | rs11851015 | 0.678154 | 0.292601 | 0.368046 |
|  | rs3930234 | 1.336196 | 0.433703 | 0.371896 |
|  | rs1042026 | 0.784904 | 0.231942 | 0.412446 |
|  | rs8062326 | 1.599997 | 0.94429 | 0.425819 |
|  | rs1876831 | 0.781394 | 0.252446 | 0.445144 |
|  | rs3738443 | 0.750809 | 0.282483 | 0.446202 |
|  | rs1573496 | 1.323297 | 0.499881 | 0.458356 |
|  | rs12388359 | 1.281659 | 0.446286 | 0.476057 |
|  | rs6701037 | 0.835072 | 0.215254 | 0.484412 |
|  | rs886205 | 1.23248 | 0.383884 | 0.502159 |
|  | rs1353899 | 0.796846 | 0.277103 | 0.513732 |
|  | rs4440177 | 1.180084 | 0.309131 | 0.527314 |
|  | rs1800759 | 0.846959 | 0.224095 | 0.530149 |
|  | rs1380131 | 1.287163 | 0.522358 | 0.53391 |
|  | rs6716455 | 0.792727 | 0.318136 | 0.562735 |
|  | rs242938 | 1.30258 | 0.613508 | 0.574625 |
|  | rs768048 | 0.800338 | 0.321991 | 0.579857 |
|  | rs2303317 | 0.887944 | 0.226383 | 0.641105 |
|  | rs9656709 | 1.121724 | 0.288259 | 0.654882 |
|  | rs10849915 | 0.888113 | 0.244511 | 0.66648 |
|  | rs933769 | 0.867414 | 0.300543 | 0.681422 |
|  | rs2369955 | 0.855769 | 0.346201 | 0.700231 |
|  | rs1497571 | 0.9068 | 0.232051 | 0.702232 |
|  | rs1318937 | 1.143983 | 0.412341 | 0.709003 |
|  | rs2154294 | 1.091179 | 0.276744 | 0.730807 |
|  | rs750338 | 1.106958 | 0.328849 | 0.732311 |
|  | rs4478858 | 1.086127 | 0.275641 | 0.744768 |
|  | rs1353621 | 1.080572 | 0.282303 | 0.766763 |
|  | rs1109501 | 0.916887 | 0.273837 | 0.771406 |
|  | rs9636231 | 1.084236 | 0.302063 | 0.771589 |
|  | rs1000579 | 1.067806 | 0.272873 | 0.797388 |
|  | rs6943555 | 1.055537 | 0.304017 | 0.851143 |
|  | rs4758317 | 0.954559 | 0.248927 | 0.85846 |
|  | rs237238 | 0.919331 | 0.480596 | 0.872178 |
|  | rs10908907 | 0.962253 | 0.284656 | 0.89651 |
|  | rs36061340 | 1.066446 | 0.55525 | 0.901665 |
|  | rs1230165 | 0.962363 | 0.318964 | 0.907851 |
|  | rs6902771 | 0.973434 | 0.246921 | 0.915465 |
|  | rs4543123 | 0.972696 | 0.295286 | 0.927341 |
|  | rs3131513 | 0.977242 | 0.25542 | 0.929815 |
|  | rs4770403 | 1.027687 | 0.331901 | 0.932608 |
|  | rs2827312 | 0.984503 | 0.274545 | 0.955337 |
|  | rs2548145 | 1.010171 | 0.257769 | 0.968365 |
|  | rs2140418 | 0.989412 | 0.317524 | 0.973539 |
|  | rs2188561 | 1.008084 | 0.30497 | 0.978768 |
|  | rs1229984 | 1.020539 | 1.038331 | 0.984057 |
|  | rs62202398 | 1.00883 | 0.526894 | 0.98657 |
|  | rs2100290 | 0.99879 | 0.254554 | 0.99621 |
| amphetamine use at 3 years post baseline | Mums PGRS | 1.695145 | 2.660163 | 0.736636 |
|  | rs1353621 | 0.470763 | 0.132807 | 0.007572 |
|  | rs1109501 | 1.884184 | 0.455465 | 0.008776 |
|  | rs2100290 | 0.538634 | 0.131755 | 0.011425 |
|  | rs1000579 | 0.513695 | 0.138279 | 0.013339 |
|  | rs3762894 | 1.88339 | 0.484688 | 0.013894 |
|  | rs3738443 | 1.870343 | 0.486075 | 0.015987 |
|  | rs1042026 | 1.754055 | 0.412118 | 0.016771 |
|  | rs11724320 | 0.576002 | 0.160209 | 0.047331 |
|  | rs13259667 | 0.160865 | 0.162159 | 0.069892 |
|  | rs4770403 | 0.518193 | 0.194706 | 0.080181 |
|  | rs237238 | 0.180546 | 0.182221 | 0.08988 |
|  | rs67031482 | 1.461229 | 0.346438 | 0.109655 |
|  | rs750338 | 0.611896 | 0.200551 | 0.13396 |
|  | rs16985179 | 0.430545 | 0.244661 | 0.138086 |
|  | rs1800759 | 0.692002 | 0.173805 | 0.142689 |
|  | rs10908907 | 1.43108 | 0.35565 | 0.149228 |
|  | rs4293630 | 1.533042 | 0.456377 | 0.151226 |
|  | rs3819197 | 1.422485 | 0.353979 | 0.156728 |
|  | rs6716455 | 0.553253 | 0.235986 | 0.165209 |
|  | rs2140418 | 1.445707 | 0.386705 | 0.1682 |
|  | rs1230165 | 0.620996 | 0.220418 | 0.179507 |
|  | rs1789891 | 0.604655 | 0.227322 | 0.180834 |
|  | rs12311304 | 0.697172 | 0.189179 | 0.183731 |
|  | rs2548145 | 0.734876 | 0.174972 | 0.19573 |
|  | rs2188561 | 0.668824 | 0.210648 | 0.201558 |
|  | rs2228093 | 0.602931 | 0.249466 | 0.221394 |
|  | rs6701037 | 0.747931 | 0.178505 | 0.223622 |
|  | rs10253361 | 0.75067 | 0.179509 | 0.230414 |
|  | rs59972978 | 1.399192 | 0.395945 | 0.235233 |
|  | rs284786 | 1.322734 | 0.322397 | 0.251151 |
|  | rs4440177 | 1.311921 | 0.311749 | 0.253241 |
|  | rs642899 | 1.321103 | 0.339443 | 0.278461 |
|  | rs2154294 | 1.283236 | 0.29909 | 0.284628 |
|  | rs1793257 | 0.354725 | 0.358588 | 0.305246 |
|  | rs2380220 | 0.684096 | 0.25807 | 0.314222 |
|  | rs3930234 | 1.346329 | 0.398502 | 0.315043 |
|  | rs6943555 | 1.277756 | 0.321924 | 0.330626 |
|  | rs8040009 | 1.303255 | 0.370049 | 0.350916 |
|  | rs1876831 | 0.756975 | 0.226262 | 0.351601 |
|  | rs1864982 | 0.704582 | 0.281379 | 0.380602 |
|  | rs7553212 | 1.230125 | 0.293186 | 0.384847 |
|  | rs8062326 | 0.422554 | 0.424174 | 0.390811 |
|  | rs10849915 | 0.807822 | 0.207621 | 0.406336 |
|  | rs12388359 | 1.300186 | 0.412832 | 0.408379 |
|  | rs4758317 | 0.820358 | 0.198063 | 0.412127 |
|  | rs12472151 | 0.553408 | 0.399463 | 0.412402 |
|  | rs59677118 | 0.708769 | 0.333187 | 0.464015 |
|  | rs768048 | 0.766631 | 0.287164 | 0.478038 |
|  | rs933769 | 0.79393 | 0.258876 | 0.47913 |
|  | rs9656709 | 1.169998 | 0.275985 | 0.505676 |
|  | rs4478858 | 0.856626 | 0.203057 | 0.513851 |
|  | rs36563 | 0.796527 | 0.283006 | 0.521986 |
|  | rs9636231 | 0.857482 | 0.230092 | 0.566645 |
|  | rs1318937 | 0.811249 | 0.304051 | 0.576761 |
|  | rs1824024 | 1.139987 | 0.28185 | 0.596168 |
|  | rs4761097 | 1.128506 | 0.262285 | 0.602952 |
|  | rs7144649 | 0.861854 | 0.250917 | 0.609595 |
|  | rs6902771 | 1.116018 | 0.258211 | 0.635195 |
|  | rs279861 | 0.898932 | 0.213299 | 0.653405 |
|  | rs9825310 | 0.903674 | 0.212752 | 0.667036 |
|  | rs10893366 | 0.877436 | 0.289744 | 0.692138 |
|  | rs1497571 | 1.093362 | 0.253775 | 0.700567 |
|  | rs11851015 | 0.872815 | 0.31226 | 0.703774 |
|  | rs886205 | 1.107766 | 0.328552 | 0.730038 |
|  | rs62202398 | 0.837228 | 0.43364 | 0.731594 |
|  | rs2303317 | 1.071735 | 0.249096 | 0.765648 |
|  | rs3131513 | 0.932624 | 0.224086 | 0.771582 |
|  | rs1229984 | 0.759704 | 0.770423 | 0.786389 |
|  | rs567926 | 0.938172 | 0.221992 | 0.787375 |
|  | rs2810114 | 1.06936 | 0.276414 | 0.795298 |
|  | rs1573496 | 1.099387 | 0.410734 | 0.79979 |
|  | rs36061340 | 0.87951 | 0.456408 | 0.804589 |
|  | rs1353899 | 1.067898 | 0.31252 | 0.822388 |
|  | rs9556711 | 0.906421 | 0.469689 | 0.849615 |
|  | rs804292 | 0.949582 | 0.260131 | 0.850212 |
|  | rs9871864 | 0.957124 | 0.224597 | 0.851857 |
|  | rs2369955 | 1.064412 | 0.366596 | 0.856176 |
|  | rs242938 | 1.08567 | 0.50831 | 0.860639 |
|  | rs9512637 | 0.962872 | 0.233735 | 0.876141 |
|  | rs2827312 | 1.036755 | 0.263496 | 0.887062 |
|  | rs420817 | 1.030379 | 0.240355 | 0.897917 |
|  | rs1380131 | 1.045115 | 0.41841 | 0.912234 |
|  | rs1344694 | 1.026646 | 0.255825 | 0.915952 |
|  | rs7590720 | 1.025216 | 0.263631 | 0.922851 |
|  | rs3764435 | 0.983385 | 0.23133 | 0.943218 |
|  | rs1908556 | 0.979405 | 0.3317 | 0.951004 |
|  | rs13160562 | 1.00163 | 0.248105 | 0.994753 |
|  | rs4543123 | 1.000379 | 0.276651 | 0.998907 |
|  | rs195204 | 0.999977 | 0.269788 | 0.999931 |
| amphetamine use at 4 years post baseline | Mums PGRS | 3.545164 | 5.041587 | 0.373499 |
|  | rs7553212 | 1.611475 | 0.342149 | 0.02462 |
|  | rs2154294 | 1.618453 | 0.347841 | 0.025077 |
|  | rs9512637 | 1.53339 | 0.323261 | 0.042585 |
|  | rs1789891 | 1.526982 | 0.382739 | 0.091262 |
|  | rs1000579 | 0.691459 | 0.157633 | 0.105573 |
|  | rs3762894 | 0.583652 | 0.203791 | 0.123047 |
|  | rs804292 | 1.42112 | 0.32685 | 0.126498 |
|  | rs2369955 | 0.566275 | 0.22469 | 0.151799 |
|  | rs1864982 | 0.568972 | 0.225497 | 0.154768 |
|  | rs8062326 | 1.854539 | 0.858269 | 0.182013 |
|  | rs12388359 | 1.429782 | 0.397403 | 0.198339 |
|  | rs9656709 | 1.317256 | 0.283569 | 0.200543 |
|  | rs2188561 | 1.325268 | 0.31067 | 0.229627 |
|  | rs279861 | 0.76964 | 0.168379 | 0.231383 |
|  | rs9636231 | 0.740056 | 0.186198 | 0.231517 |
|  | rs6902771 | 1.284807 | 0.270069 | 0.233173 |
|  | rs567926 | 0.77024 | 0.169047 | 0.234261 |
|  | rs4440177 | 1.285621 | 0.276901 | 0.243417 |
|  | rs3930234 | 0.682624 | 0.228416 | 0.25385 |
|  | rs9556711 | 0.542211 | 0.320488 | 0.300403 |
|  | rs1229984 | 1.813848 | 1.081859 | 0.318117 |
|  | rs1876831 | 1.258194 | 0.297676 | 0.331656 |
|  | rs3131513 | 0.807825 | 0.180022 | 0.338241 |
|  | rs13160562 | 1.227422 | 0.267478 | 0.347046 |
|  | rs2548145 | 0.818307 | 0.174549 | 0.347193 |
|  | rs1824024 | 1.227141 | 0.27254 | 0.356724 |
|  | rs2228093 | 1.301095 | 0.375502 | 0.36177 |
|  | rs1353899 | 0.766923 | 0.225735 | 0.367283 |
|  | rs16985179 | 0.68473 | 0.292548 | 0.375377 |
|  | rs59972978 | 0.770027 | 0.235093 | 0.392018 |
|  | rs6716455 | 1.24309 | 0.352981 | 0.443486 |
|  | rs4543123 | 1.20161 | 0.288266 | 0.443927 |
|  | rs750338 | 0.81563 | 0.220374 | 0.450688 |
|  | rs1908556 | 0.779084 | 0.260595 | 0.455473 |
|  | rs12311304 | 0.840301 | 0.198487 | 0.461357 |
|  | rs1793257 | 0.591002 | 0.4267 | 0.466339 |
|  | rs11851015 | 0.785196 | 0.265415 | 0.474363 |
|  | rs1109501 | 1.17179 | 0.27485 | 0.499115 |
|  | rs36563 | 0.80785 | 0.258758 | 0.505299 |
|  | rs242938 | 0.710042 | 0.365926 | 0.5064 |
|  | rs36061340 | 0.718732 | 0.371074 | 0.522373 |
|  | rs10849915 | 1.149554 | 0.253744 | 0.527767 |
|  | rs642899 | 1.164206 | 0.281291 | 0.529178 |
|  | rs8040009 | 0.832479 | 0.243492 | 0.530758 |
|  | rs2827312 | 0.863449 | 0.204496 | 0.535306 |
|  | rs886205 | 1.16676 | 0.30852 | 0.559712 |
|  | rs6701037 | 0.88514 | 0.188779 | 0.567272 |
|  | rs11724320 | 0.878234 | 0.201729 | 0.571888 |
|  | rs284786 | 1.134701 | 0.256937 | 0.576791 |
|  | rs62202398 | 1.241565 | 0.494125 | 0.586668 |
|  | rs10893366 | 0.851073 | 0.258177 | 0.595017 |
|  | rs9825310 | 0.896271 | 0.191641 | 0.608534 |
|  | rs2810114 | 0.882711 | 0.215841 | 0.609902 |
|  | rs768048 | 0.865526 | 0.278952 | 0.654084 |
|  | rs933769 | 1.12525 | 0.297964 | 0.655854 |
|  | rs2380220 | 1.131769 | 0.324146 | 0.665605 |
|  | rs1344694 | 1.096471 | 0.244416 | 0.679494 |
|  | rs1318937 | 1.129671 | 0.339686 | 0.685121 |
|  | rs7144649 | 1.104826 | 0.272586 | 0.686176 |
|  | rs4761097 | 0.918854 | 0.195816 | 0.691284 |
|  | rs10908907 | 0.914122 | 0.228015 | 0.718865 |
|  | rs3764435 | 1.077711 | 0.230162 | 0.726019 |
|  | rs7590720 | 1.083801 | 0.248988 | 0.726119 |
|  | rs1353621 | 1.078227 | 0.232118 | 0.726441 |
|  | rs4770403 | 0.914096 | 0.254169 | 0.746672 |
|  | rs2303317 | 1.069321 | 0.225965 | 0.751111 |
|  | rs10253361 | 0.935009 | 0.198415 | 0.751495 |
|  | rs59677118 | 0.884914 | 0.344057 | 0.753168 |
|  | rs4293630 | 1.099052 | 0.331233 | 0.753988 |
|  | rs1573496 | 1.109166 | 0.371527 | 0.757083 |
|  | rs9871864 | 0.936078 | 0.200583 | 0.757874 |
|  | rs4478858 | 1.063398 | 0.2246 | 0.771024 |
|  | rs1042026 | 0.941551 | 0.220334 | 0.796895 |
|  | rs1497571 | 1.055224 | 0.223067 | 0.799277 |
|  | rs195204 | 0.95057 | 0.236349 | 0.838445 |
|  | rs2140418 | 1.054196 | 0.278286 | 0.841532 |
|  | rs1230165 | 1.051664 | 0.282972 | 0.851492 |
|  | rs6943555 | 1.043905 | 0.250132 | 0.857682 |
|  | rs4758317 | 1.038446 | 0.223366 | 0.860775 |
|  | rs1800759 | 1.036925 | 0.22358 | 0.866452 |
|  | rs2100290 | 0.966193 | 0.204617 | 0.870996 |
|  | rs3819197 | 1.036705 | 0.251902 | 0.882064 |
|  | rs1380131 | 0.953182 | 0.356571 | 0.898007 |
|  | rs420817 | 1.026125 | 0.21662 | 0.902769 |
|  | rs12472151 | 0.944815 | 0.49038 | 0.912908 |
|  | rs237238 | 0.974855 | 0.418268 | 0.95267 |
|  | rs13259667 | 1.012459 | 0.400679 | 0.97504 |
|  | rs67031482 | 1.005565 | 0.213733 | 0.97917 |
|  | rs3738443 | 1.00331 | 0.282295 | 0.990628 |
| amphetamine use at 6 years post baseline | Mums PGRS | 2.02059 | 2.731434 | 0.60283 |
|  | rs7590720 | 1.781933 | 0.365791 | 0.004889 |
|  | rs1789891 | 0.374426 | 0.147579 | 0.012689 |
|  | rs1344694 | 1.664105 | 0.340379 | 0.012778 |
|  | rs1109501 | 1.638455 | 0.347411 | 0.019878 |
|  | rs2369955 | 0.345858 | 0.159443 | 0.021276 |
|  | rs16985179 | 0.399678 | 0.201972 | 0.069551 |
|  | rs1042026 | 1.431974 | 0.296446 | 0.082847 |
|  | rs36061340 | 1.76339 | 0.594925 | 0.0927 |
|  | rs3819197 | 1.427073 | 0.305339 | 0.096493 |
|  | rs9871864 | 1.383022 | 0.280617 | 0.110004 |
|  | rs933769 | 0.614026 | 0.189541 | 0.11411 |
|  | rs4543123 | 1.414291 | 0.31068 | 0.11458 |
|  | rs12388359 | 0.570329 | 0.211445 | 0.129863 |
|  | rs1824024 | 1.336409 | 0.278721 | 0.1644 |
|  | rs9636231 | 0.72336 | 0.174406 | 0.179213 |
|  | rs4293630 | 0.632033 | 0.222077 | 0.191624 |
|  | rs6701037 | 0.772942 | 0.158248 | 0.2084 |
|  | rs9825310 | 0.786038 | 0.161596 | 0.241576 |
|  | rs3738443 | 1.331166 | 0.325194 | 0.241618 |
|  | rs2380220 | 0.693236 | 0.223127 | 0.254984 |
|  | rs886205 | 1.314864 | 0.319892 | 0.260531 |
|  | rs13160562 | 0.783201 | 0.17649 | 0.278182 |
|  | rs8062326 | 1.633615 | 0.746297 | 0.282674 |
|  | rs9656709 | 1.240733 | 0.253933 | 0.291913 |
|  | rs642899 | 1.252928 | 0.282212 | 0.316793 |
|  | rs11851015 | 1.308681 | 0.352041 | 0.317283 |
|  | rs7553212 | 1.226678 | 0.251837 | 0.31965 |
|  | rs6902771 | 1.2187 | 0.243858 | 0.322935 |
|  | rs284786 | 0.798353 | 0.183597 | 0.327442 |
|  | rs2188561 | 0.7977 | 0.203738 | 0.376183 |
|  | rs3131513 | 0.830376 | 0.174838 | 0.377341 |
|  | rs1353899 | 0.786563 | 0.217335 | 0.384907 |
|  | rs2827312 | 0.821606 | 0.186985 | 0.387922 |
|  | rs279861 | 0.837273 | 0.172592 | 0.388911 |
|  | rs1876831 | 1.211176 | 0.275381 | 0.399423 |
|  | rs1908556 | 0.781272 | 0.248682 | 0.438067 |
|  | rs750338 | 0.823647 | 0.21085 | 0.448524 |
|  | rs4770403 | 0.820746 | 0.225699 | 0.472539 |
|  | rs804292 | 0.838599 | 0.205743 | 0.47309 |
|  | rs10893366 | 0.823196 | 0.24075 | 0.505883 |
|  | rs11724320 | 1.148947 | 0.240095 | 0.506415 |
|  | rs62202398 | 1.261045 | 0.472421 | 0.535835 |
|  | rs1800759 | 1.127202 | 0.228257 | 0.554315 |
|  | rs12472151 | 1.288775 | 0.553542 | 0.554753 |
|  | rs36563 | 1.167642 | 0.308535 | 0.557511 |
|  | rs6716455 | 1.172921 | 0.322463 | 0.561813 |
|  | rs59677118 | 0.802663 | 0.308849 | 0.567805 |
|  | rs1230165 | 0.856911 | 0.23339 | 0.570733 |
|  | rs1864982 | 0.833802 | 0.268391 | 0.572301 |
|  | rs242938 | 0.789319 | 0.364776 | 0.608698 |
|  | rs1497571 | 1.107681 | 0.222732 | 0.611033 |
|  | rs1229984 | 0.601346 | 0.608097 | 0.615008 |
|  | rs13259667 | 1.188538 | 0.420018 | 0.62501 |
|  | rs9556711 | 1.209868 | 0.479737 | 0.630902 |
|  | rs2140418 | 1.119126 | 0.27431 | 0.64611 |
|  | rs567926 | 0.911164 | 0.186524 | 0.649498 |
|  | rs4758317 | 0.913981 | 0.188536 | 0.662809 |
|  | rs2100290 | 0.918314 | 0.184759 | 0.671893 |
|  | rs1793257 | 0.780791 | 0.46209 | 0.675866 |
|  | rs4440177 | 1.090715 | 0.227158 | 0.676723 |
|  | rs237238 | 1.159531 | 0.437496 | 0.694839 |
|  | rs1573496 | 1.124163 | 0.359842 | 0.714637 |
|  | rs1000579 | 0.930196 | 0.190397 | 0.7237 |
|  | rs4761097 | 1.069827 | 0.213977 | 0.735766 |
|  | rs7144649 | 1.083035 | 0.256161 | 0.735926 |
|  | rs59972978 | 1.075989 | 0.28195 | 0.779858 |
|  | rs195204 | 0.939356 | 0.222402 | 0.791597 |
|  | rs10253361 | 1.052805 | 0.210956 | 0.797326 |
|  | rs2303317 | 0.953567 | 0.190602 | 0.811986 |
|  | rs2810114 | 0.947563 | 0.215307 | 0.812623 |
|  | rs2228093 | 0.933552 | 0.284182 | 0.821298 |
|  | rs9512637 | 0.959483 | 0.200165 | 0.842841 |
|  | rs6943555 | 0.958593 | 0.222935 | 0.85571 |
|  | rs10908907 | 1.035951 | 0.236338 | 0.876962 |
|  | rs3930234 | 0.959468 | 0.272822 | 0.884305 |
|  | rs1380131 | 0.965848 | 0.339712 | 0.9213 |
|  | rs420817 | 0.981071 | 0.195734 | 0.923689 |
|  | rs10849915 | 0.980723 | 0.209715 | 0.927469 |
|  | rs3762894 | 0.978899 | 0.264979 | 0.937201 |
|  | rs2154294 | 1.015066 | 0.20267 | 0.9403 |
|  | rs2548145 | 0.985437 | 0.198975 | 0.942081 |
|  | rs12311304 | 0.985855 | 0.213388 | 0.947523 |
|  | rs1318937 | 0.98258 | 0.291281 | 0.952729 |
|  | rs3764435 | 0.989474 | 0.199819 | 0.958209 |
|  | rs8040009 | 1.010007 | 0.264271 | 0.969642 |
|  | rs4478858 | 1.003592 | 0.201307 | 0.98574 |
|  | rs67031482 | 0.997641 | 0.202269 | 0.990707 |
|  | rs768048 | 1.003111 | 0.290784 | 0.991452 |
|  | rs1353621 | 0.998249 | 0.207172 | 0.993263 |
| antidepressant use at 2 years post baseline | Mums PGRS | 1.123828 | 0.657128 | 0.841753 |
|  | rs7590720 | 1.328698 | 0.120944 | 0.001795 |
|  | rs1344694 | 1.280954 | 0.114908 | 0.005777 |
|  | rs420817 | 1.26525 | 0.110428 | 0.007025 |
|  | rs12388359 | 1.344378 | 0.158554 | 0.0121 |
|  | rs1824024 | 0.805774 | 0.078631 | 0.0269 |
|  | rs10893366 | 1.270033 | 0.139003 | 0.028957 |
|  | rs59677118 | 1.334825 | 0.180409 | 0.032614 |
|  | rs13259667 | 1.348621 | 0.197128 | 0.040743 |
|  | rs195204 | 1.198362 | 0.115578 | 0.060624 |
|  | rs1380131 | 0.729779 | 0.125396 | 0.066755 |
|  | rs1864982 | 0.777457 | 0.10974 | 0.074527 |
|  | rs10253361 | 1.160378 | 0.100276 | 0.085204 |
|  | rs750338 | 1.180778 | 0.118098 | 0.096623 |
|  | rs567926 | 0.865028 | 0.076793 | 0.102415 |
|  | rs67031482 | 1.136797 | 0.098869 | 0.140422 |
|  | rs2154294 | 0.880748 | 0.07658 | 0.14417 |
|  | rs16985179 | 0.787472 | 0.129941 | 0.147629 |
|  | rs10849915 | 1.136187 | 0.102887 | 0.158555 |
|  | rs2810114 | 0.868925 | 0.087107 | 0.161059 |
|  | rs36563 | 0.839432 | 0.107504 | 0.171721 |
|  | rs1318937 | 1.17796 | 0.14396 | 0.180189 |
|  | rs2140418 | 1.145329 | 0.120381 | 0.196705 |
|  | rs9656709 | 1.118661 | 0.098013 | 0.20061 |
|  | rs8040009 | 1.147514 | 0.125526 | 0.208436 |
|  | rs3738443 | 0.862389 | 0.104644 | 0.222429 |
|  | rs6716455 | 1.150908 | 0.137906 | 0.240805 |
|  | rs242938 | 0.803432 | 0.157176 | 0.263244 |
|  | rs12472151 | 0.780907 | 0.178174 | 0.278421 |
|  | rs1800759 | 0.907897 | 0.081046 | 0.27907 |
|  | rs279861 | 0.909742 | 0.080284 | 0.283768 |
|  | rs642899 | 0.898078 | 0.095006 | 0.309549 |
|  | rs237238 | 0.837611 | 0.154437 | 0.336513 |
|  | rs6902771 | 0.921359 | 0.079906 | 0.344956 |
|  | rs2827312 | 1.092817 | 0.10274 | 0.34512 |
|  | rs4293630 | 0.890901 | 0.118743 | 0.386088 |
|  | rs9871864 | 1.078166 | 0.094189 | 0.388956 |
|  | rs11851015 | 1.109589 | 0.136329 | 0.397341 |
|  | rs36061340 | 0.850743 | 0.165751 | 0.406725 |
|  | rs3819197 | 0.924113 | 0.095211 | 0.44368 |
|  | rs4543123 | 1.077427 | 0.108689 | 0.459746 |
|  | rs933769 | 1.084807 | 0.119572 | 0.460201 |
|  | rs4478858 | 1.063837 | 0.092173 | 0.475089 |
|  | rs9636231 | 1.070345 | 0.102011 | 0.475672 |
|  | rs886205 | 0.923218 | 0.107164 | 0.491293 |
|  | rs804292 | 1.06977 | 0.106502 | 0.498124 |
|  | rs1497571 | 0.944127 | 0.082217 | 0.509109 |
|  | rs59972978 | 0.929401 | 0.109285 | 0.533514 |
|  | rs13160562 | 0.94472 | 0.088487 | 0.543765 |
|  | rs4758317 | 1.054734 | 0.093325 | 0.547006 |
|  | rs1908556 | 1.071827 | 0.131135 | 0.570747 |
|  | rs1789891 | 1.065435 | 0.121936 | 0.579703 |
|  | rs2188561 | 0.943927 | 0.099081 | 0.582482 |
|  | rs1229984 | 0.821257 | 0.29928 | 0.588943 |
|  | rs1042026 | 0.952276 | 0.091455 | 0.610629 |
|  | rs1230165 | 1.05733 | 0.116094 | 0.611653 |
|  | rs8062326 | 0.87398 | 0.232225 | 0.612199 |
|  | rs7553212 | 0.954687 | 0.088321 | 0.6162 |
|  | rs4761097 | 0.957468 | 0.083454 | 0.61802 |
|  | rs1000579 | 1.04328 | 0.091148 | 0.627702 |
|  | rs9512637 | 1.043628 | 0.093017 | 0.631857 |
|  | rs4770403 | 0.952365 | 0.107219 | 0.664636 |
|  | rs9556711 | 0.922492 | 0.173965 | 0.668791 |
|  | rs11724320 | 1.030958 | 0.094725 | 0.740017 |
|  | rs1793257 | 1.075122 | 0.241552 | 0.747153 |
|  | rs2369955 | 1.041695 | 0.1339 | 0.750641 |
|  | rs9825310 | 0.974146 | 0.085144 | 0.764412 |
|  | rs10908907 | 0.970315 | 0.097593 | 0.764469 |
|  | rs12311304 | 0.975897 | 0.091282 | 0.794213 |
|  | rs3762894 | 1.027895 | 0.118881 | 0.81197 |
|  | rs284786 | 1.022473 | 0.09653 | 0.813899 |
|  | rs1353621 | 0.980751 | 0.088386 | 0.829246 |
|  | rs2303317 | 0.98163 | 0.085011 | 0.830478 |
|  | rs1109501 | 1.020869 | 0.101325 | 0.835156 |
|  | rs2548145 | 1.017745 | 0.088531 | 0.839752 |
|  | rs6943555 | 1.019517 | 0.10103 | 0.845351 |
|  | rs1573496 | 1.027879 | 0.146263 | 0.846768 |
|  | rs6701037 | 1.01637 | 0.088336 | 0.851798 |
|  | rs7144649 | 0.983685 | 0.102822 | 0.874952 |
|  | rs3764435 | 1.013603 | 0.088733 | 0.877337 |
|  | rs62202398 | 1.022404 | 0.181146 | 0.900479 |
|  | rs768048 | 0.985185 | 0.125021 | 0.906371 |
|  | rs2380220 | 1.013559 | 0.123159 | 0.911743 |
|  | rs2100290 | 0.991232 | 0.086128 | 0.919267 |
|  | rs3930234 | 0.990122 | 0.119824 | 0.93462 |
|  | rs4440177 | 1.004545 | 0.091905 | 0.960465 |
|  | rs3131513 | 0.996289 | 0.088586 | 0.96665 |
|  | rs2228093 | 1.004802 | 0.129414 | 0.970327 |
|  | rs1353899 | 1.001221 | 0.110472 | 0.991173 |
|  | rs1876831 | 1.00087 | 0.102806 | 0.993242 |
| antidepressant use at 3 years post baseline | Mums PGRS | 1.775703 | 1.01981 | 0.317409 |
|  | rs1824024 | 0.772918 | 0.074742 | 0.007729 |
|  | rs6716455 | 1.288491 | 0.147513 | 0.026828 |
|  | rs67031482 | 1.182569 | 0.101408 | 0.050524 |
|  | rs1864982 | 0.762708 | 0.107236 | 0.054027 |
|  | rs9656709 | 1.172825 | 0.101247 | 0.064799 |
|  | rs420817 | 1.167896 | 0.099886 | 0.069572 |
|  | rs10893366 | 1.218849 | 0.133556 | 0.0709 |
|  | rs642899 | 0.828245 | 0.087714 | 0.07517 |
|  | rs2154294 | 0.861921 | 0.073747 | 0.082446 |
|  | rs195204 | 1.171957 | 0.111661 | 0.095834 |
|  | rs9556711 | 0.708681 | 0.148128 | 0.099465 |
|  | rs2827312 | 1.155241 | 0.106394 | 0.117133 |
|  | rs10849915 | 1.145901 | 0.102008 | 0.126041 |
|  | rs8040009 | 1.176143 | 0.125918 | 0.129665 |
|  | rs12472151 | 0.711002 | 0.165785 | 0.143525 |
|  | rs4758317 | 0.881774 | 0.077363 | 0.151554 |
|  | rs3131513 | 0.88103 | 0.077935 | 0.152177 |
|  | rs750338 | 1.147474 | 0.114179 | 0.166826 |
|  | rs1042026 | 0.879365 | 0.084356 | 0.180208 |
|  | rs3819197 | 0.877026 | 0.089683 | 0.199421 |
|  | rs16985179 | 0.818776 | 0.130548 | 0.209834 |
|  | rs1229984 | 1.400911 | 0.395035 | 0.231877 |
|  | rs4293630 | 0.857533 | 0.113731 | 0.24651 |
|  | rs886205 | 0.874923 | 0.102259 | 0.252939 |
|  | rs3762894 | 1.131511 | 0.124101 | 0.259945 |
|  | rs10253361 | 1.097812 | 0.093332 | 0.272352 |
|  | rs3738443 | 0.879024 | 0.104684 | 0.278929 |
|  | rs12388359 | 1.134983 | 0.138581 | 0.299733 |
|  | rs1573496 | 0.859168 | 0.129292 | 0.313128 |
|  | rs2810114 | 0.912368 | 0.089346 | 0.348999 |
|  | rs7144649 | 0.908207 | 0.095117 | 0.35792 |
|  | rs4770403 | 0.910136 | 0.102215 | 0.401794 |
|  | rs1380131 | 1.127003 | 0.160959 | 0.402509 |
|  | rs768048 | 1.099744 | 0.132176 | 0.428901 |
|  | rs8062326 | 0.81403 | 0.215415 | 0.436841 |
|  | rs1353899 | 0.916824 | 0.102498 | 0.4373 |
|  | rs4440177 | 0.931548 | 0.08514 | 0.437851 |
|  | rs62202398 | 1.137614 | 0.18972 | 0.439453 |
|  | rs1908556 | 0.906752 | 0.115331 | 0.441541 |
|  | rs2369955 | 0.904902 | 0.120478 | 0.452923 |
|  | rs804292 | 1.074625 | 0.104783 | 0.460441 |
|  | rs1876831 | 1.075438 | 0.106948 | 0.464577 |
|  | rs1109501 | 1.073214 | 0.10398 | 0.465829 |
|  | rs13259667 | 1.110351 | 0.170718 | 0.495991 |
|  | rs12311304 | 1.063262 | 0.096519 | 0.499201 |
|  | rs59677118 | 0.904104 | 0.14003 | 0.515122 |
|  | rs36563 | 0.924694 | 0.113473 | 0.52347 |
|  | rs6943555 | 1.062412 | 0.102301 | 0.529527 |
|  | rs4761097 | 0.949377 | 0.081397 | 0.544577 |
|  | rs3930234 | 0.929993 | 0.112316 | 0.547868 |
|  | rs9825310 | 1.051617 | 0.090123 | 0.557024 |
|  | rs7590720 | 1.051551 | 0.09854 | 0.591675 |
|  | rs1230165 | 1.058249 | 0.114249 | 0.59999 |
|  | rs1353621 | 1.045928 | 0.091465 | 0.607602 |
|  | rs2303317 | 1.044214 | 0.088813 | 0.610978 |
|  | rs2228093 | 0.936613 | 0.120818 | 0.611695 |
|  | rs4478858 | 0.960505 | 0.082373 | 0.638448 |
|  | rs567926 | 0.961765 | 0.083089 | 0.6518 |
|  | rs1000579 | 0.962046 | 0.083606 | 0.656147 |
|  | rs1344694 | 1.040549 | 0.094729 | 0.662392 |
|  | rs3764435 | 1.037963 | 0.089309 | 0.664988 |
|  | rs1793257 | 1.098877 | 0.241954 | 0.668484 |
|  | rs2100290 | 1.036815 | 0.088582 | 0.67218 |
|  | rs1318937 | 1.051083 | 0.130934 | 0.689201 |
|  | rs6902771 | 0.969466 | 0.082428 | 0.715326 |
|  | rs11851015 | 0.958457 | 0.121108 | 0.737022 |
|  | rs237238 | 0.946856 | 0.163914 | 0.752425 |
|  | rs284786 | 0.971771 | 0.090825 | 0.759321 |
|  | rs36061340 | 1.05354 | 0.185506 | 0.767069 |
|  | rs9636231 | 0.972285 | 0.092771 | 0.768323 |
|  | rs1497571 | 0.977751 | 0.083161 | 0.791361 |
|  | rs1789891 | 1.029415 | 0.11727 | 0.799122 |
|  | rs933769 | 1.025297 | 0.112285 | 0.819556 |
|  | rs59972978 | 1.024909 | 0.115597 | 0.827319 |
|  | rs279861 | 0.982646 | 0.084768 | 0.839188 |
|  | rs2548145 | 1.016823 | 0.087027 | 0.84545 |
|  | rs10908907 | 0.983582 | 0.096862 | 0.866508 |
|  | rs9871864 | 1.013247 | 0.086794 | 0.877895 |
|  | rs1800759 | 1.013022 | 0.088023 | 0.881633 |
|  | rs7553212 | 1.013212 | 0.090929 | 0.883721 |
|  | rs2380220 | 0.982492 | 0.118969 | 0.884025 |
|  | rs2188561 | 0.985603 | 0.100564 | 0.886978 |
|  | rs9512637 | 1.012508 | 0.089291 | 0.887905 |
|  | rs242938 | 1.024929 | 0.179999 | 0.888497 |
|  | rs4543123 | 0.986877 | 0.100196 | 0.896478 |
|  | rs13160562 | 0.99327 | 0.090162 | 0.940701 |
|  | rs6701037 | 1.004301 | 0.085849 | 0.959953 |
|  | rs11724320 | 1.004093 | 0.091111 | 0.964099 |
|  | rs2140418 | 0.995459 | 0.107769 | 0.966464 |
| antidepressant use at 4 years post baseline | Mums PGRS | 0.96353 | 0.511268 | 0.944181 |
|  | rs195204 | 1.240243 | 0.108212 | 0.013599 |
|  | rs12472151 | 0.592723 | 0.138079 | 0.024758 |
|  | rs3762894 | 1.231063 | 0.122334 | 0.036447 |
|  | rs642899 | 0.834539 | 0.081832 | 0.065096 |
|  | rs2188561 | 1.178573 | 0.106238 | 0.068343 |
|  | rs10908907 | 0.843883 | 0.079737 | 0.072428 |
|  | rs2303317 | 0.875163 | 0.069153 | 0.091498 |
|  | rs237238 | 0.743063 | 0.131425 | 0.09314 |
|  | rs6943555 | 1.15593 | 0.100977 | 0.097156 |
|  | rs1344694 | 1.134906 | 0.093915 | 0.126194 |
|  | rs1908556 | 1.175638 | 0.126445 | 0.132461 |
|  | rs1109501 | 1.140886 | 0.100463 | 0.13444 |
|  | rs750338 | 1.141835 | 0.104899 | 0.148807 |
|  | rs9871864 | 1.119844 | 0.088824 | 0.153575 |
|  | rs10893366 | 1.155668 | 0.118929 | 0.159759 |
|  | rs1864982 | 0.841884 | 0.105784 | 0.170759 |
|  | rs7590720 | 1.122319 | 0.095587 | 0.175445 |
|  | rs1824024 | 0.889886 | 0.077271 | 0.179102 |
|  | rs4543123 | 0.88851 | 0.08541 | 0.218803 |
|  | rs62202398 | 1.203097 | 0.182063 | 0.221767 |
|  | rs1353899 | 0.881052 | 0.091979 | 0.225108 |
|  | rs10253361 | 1.098331 | 0.086126 | 0.23166 |
|  | rs4761097 | 0.910196 | 0.072203 | 0.235554 |
|  | rs2827312 | 1.10228 | 0.094295 | 0.254974 |
|  | rs16985179 | 0.847415 | 0.123276 | 0.255073 |
|  | rs13259667 | 0.836875 | 0.132597 | 0.261039 |
|  | rs886205 | 0.887785 | 0.094904 | 0.265525 |
|  | rs11724320 | 0.911333 | 0.077287 | 0.273602 |
|  | rs9636231 | 1.098654 | 0.094466 | 0.273853 |
|  | rs9656709 | 1.090031 | 0.086812 | 0.279062 |
|  | rs2369955 | 0.878836 | 0.10887 | 0.29713 |
|  | rs3764435 | 0.921612 | 0.073334 | 0.304944 |
|  | rs8040009 | 1.108134 | 0.111364 | 0.306927 |
|  | rs36563 | 1.114362 | 0.118555 | 0.308773 |
|  | rs7553212 | 1.086016 | 0.089441 | 0.316378 |
|  | rs4758317 | 0.922971 | 0.074338 | 0.31963 |
|  | rs420817 | 1.072897 | 0.084317 | 0.370604 |
|  | rs6902771 | 0.934026 | 0.073459 | 0.3855 |
|  | rs2154294 | 0.936007 | 0.073486 | 0.3996 |
|  | rs1229984 | 0.771829 | 0.266058 | 0.452454 |
|  | rs1380131 | 0.899723 | 0.127652 | 0.456406 |
|  | rs2100290 | 0.944286 | 0.074446 | 0.46714 |
|  | rs1000579 | 1.059347 | 0.084066 | 0.467531 |
|  | rs8062326 | 0.840198 | 0.205658 | 0.476871 |
|  | rs3131513 | 0.947533 | 0.076859 | 0.506425 |
|  | rs1353621 | 0.950241 | 0.077321 | 0.530496 |
|  | rs7144649 | 1.053411 | 0.098039 | 0.5761 |
|  | rs1789891 | 1.055826 | 0.109823 | 0.601492 |
|  | rs59677118 | 1.071692 | 0.143985 | 0.606309 |
|  | rs1497571 | 0.961857 | 0.07582 | 0.621766 |
|  | rs768048 | 1.05549 | 0.118003 | 0.629053 |
|  | rs36061340 | 1.079721 | 0.174552 | 0.635171 |
|  | rs13160562 | 1.039383 | 0.086769 | 0.643573 |
|  | rs2228093 | 0.949547 | 0.112486 | 0.662097 |
|  | rs67031482 | 1.034994 | 0.081857 | 0.663633 |
|  | rs3930234 | 0.954076 | 0.105765 | 0.671506 |
|  | rs9556711 | 1.071439 | 0.17552 | 0.673595 |
|  | rs1793257 | 1.090315 | 0.223875 | 0.673676 |
|  | rs11851015 | 1.046426 | 0.118837 | 0.689452 |
|  | rs10849915 | 1.03377 | 0.08614 | 0.690203 |
|  | rs1800759 | 0.969915 | 0.078362 | 0.705361 |
|  | rs4770403 | 0.963041 | 0.097959 | 0.71121 |
|  | rs279861 | 0.972287 | 0.077465 | 0.724279 |
|  | rs933769 | 0.964272 | 0.09951 | 0.724431 |
|  | rs6716455 | 1.040158 | 0.116947 | 0.726197 |
|  | rs242938 | 0.950403 | 0.160202 | 0.762819 |
|  | rs804292 | 1.024953 | 0.093546 | 0.787129 |
|  | rs9825310 | 0.97952 | 0.077639 | 0.794045 |
|  | rs1318937 | 0.973009 | 0.114789 | 0.816588 |
|  | rs2380220 | 0.975776 | 0.10908 | 0.826366 |
|  | rs1573496 | 1.028259 | 0.132256 | 0.828471 |
|  | rs1042026 | 0.981846 | 0.08468 | 0.831771 |
|  | rs12311304 | 1.017549 | 0.086119 | 0.837143 |
|  | rs4440177 | 0.985088 | 0.082021 | 0.856801 |
|  | rs284786 | 0.984986 | 0.084974 | 0.860802 |
|  | rs6701037 | 0.988079 | 0.077967 | 0.879203 |
|  | rs3819197 | 0.986672 | 0.090387 | 0.883548 |
|  | rs2548145 | 0.992379 | 0.078126 | 0.92259 |
|  | rs12388359 | 1.010351 | 0.117448 | 0.929413 |
|  | rs2140418 | 1.008643 | 0.100445 | 0.931135 |
|  | rs59972978 | 0.99313 | 0.10417 | 0.947597 |
|  | rs3738443 | 0.99314 | 0.104331 | 0.947755 |
|  | rs2810114 | 0.995605 | 0.087933 | 0.960229 |
|  | rs4478858 | 0.996474 | 0.078653 | 0.964303 |
|  | rs1876831 | 0.996773 | 0.092776 | 0.972296 |
|  | rs567926 | 1.002145 | 0.079789 | 0.978526 |
|  | rs4293630 | 0.997925 | 0.115812 | 0.985722 |
|  | rs9512637 | 0.998932 | 0.0813 | 0.989527 |
|  | rs1230165 | 1.000052 | 0.101746 | 0.999593 |
| antidepressant use at 6 years post baseline | Mums PGRS | 0.692078 | 0.350669 | 0.467597 |
|  | rs1353899 | 1.308833 | 0.118053 | 0.002846 |
|  | rs6943555 | 1.224679 | 0.100859 | 0.013854 |
|  | rs7553212 | 1.175866 | 0.090954 | 0.036223 |
|  | rs1229984 | 1.636978 | 0.386496 | 0.036848 |
|  | rs2369955 | 0.802541 | 0.096732 | 0.067999 |
|  | rs11851015 | 1.206232 | 0.125419 | 0.071337 |
|  | rs7144649 | 1.142494 | 0.100073 | 0.128299 |
|  | rs4293630 | 0.839368 | 0.097925 | 0.133374 |
|  | rs9636231 | 1.129496 | 0.092713 | 0.137939 |
|  | rs59677118 | 0.81655 | 0.115203 | 0.150863 |
|  | rs2100290 | 1.113983 | 0.083797 | 0.151297 |
|  | rs195204 | 1.12803 | 0.09552 | 0.154821 |
|  | rs4440177 | 0.892509 | 0.071718 | 0.157009 |
|  | rs2154294 | 0.899587 | 0.067338 | 0.157458 |
|  | rs2827312 | 1.119376 | 0.091659 | 0.168447 |
|  | rs13160562 | 0.89544 | 0.072829 | 0.174505 |
|  | rs6701037 | 1.104971 | 0.082863 | 0.183165 |
|  | rs567926 | 1.105456 | 0.083536 | 0.184596 |
|  | rs10893366 | 1.137467 | 0.112646 | 0.193386 |
|  | rs1000579 | 0.906821 | 0.069528 | 0.202062 |
|  | rs12472151 | 0.807305 | 0.156332 | 0.268996 |
|  | rs16985179 | 1.145711 | 0.143667 | 0.278026 |
|  | rs67031482 | 1.084922 | 0.082249 | 0.282307 |
|  | rs279861 | 1.084353 | 0.082085 | 0.28471 |
|  | rs1876831 | 1.09685 | 0.095562 | 0.28867 |
|  | rs1789891 | 0.900937 | 0.09451 | 0.320002 |
|  | rs4770403 | 0.907608 | 0.089984 | 0.328174 |
|  | rs750338 | 1.08403 | 0.096118 | 0.36283 |
|  | rs36061340 | 0.861838 | 0.144793 | 0.376146 |
|  | rs1230165 | 0.918306 | 0.090933 | 0.389423 |
|  | rs642899 | 0.925133 | 0.084014 | 0.3915 |
|  | rs1344694 | 1.070591 | 0.085309 | 0.391988 |
|  | rs4543123 | 1.077273 | 0.094248 | 0.394894 |
|  | rs12388359 | 1.089815 | 0.118495 | 0.428929 |
|  | rs237238 | 1.117845 | 0.160073 | 0.43659 |
|  | rs36563 | 0.920148 | 0.098542 | 0.43711 |
|  | rs7590720 | 1.060754 | 0.08717 | 0.472937 |
|  | rs11724320 | 1.056276 | 0.083456 | 0.488347 |
|  | rs10253361 | 1.053136 | 0.078825 | 0.48912 |
|  | rs3762894 | 0.931598 | 0.095674 | 0.490246 |
|  | rs3738443 | 1.065383 | 0.104468 | 0.518346 |
|  | rs2188561 | 1.057594 | 0.093129 | 0.524833 |
|  | rs9825310 | 0.953906 | 0.072309 | 0.533587 |
|  | rs62202398 | 0.90605 | 0.144918 | 0.537341 |
|  | rs6716455 | 0.934474 | 0.103448 | 0.54041 |
|  | rs13259667 | 0.923511 | 0.134328 | 0.584331 |
|  | rs1824024 | 1.044416 | 0.084116 | 0.589476 |
|  | rs1109501 | 1.045751 | 0.089649 | 0.601788 |
|  | rs10908907 | 0.95853 | 0.083179 | 0.625496 |
|  | rs1793257 | 1.087767 | 0.208833 | 0.661241 |
|  | rs1042026 | 0.964405 | 0.079794 | 0.661345 |
|  | rs9871864 | 1.033323 | 0.078237 | 0.665056 |
|  | rs12311304 | 0.965618 | 0.078347 | 0.666318 |
|  | rs1800759 | 0.97046 | 0.074503 | 0.696112 |
|  | rs4478858 | 1.029446 | 0.077018 | 0.698095 |
|  | rs242938 | 1.05908 | 0.161667 | 0.706894 |
|  | rs8062326 | 0.922908 | 0.204845 | 0.717763 |
|  | rs1318937 | 1.0398 | 0.112862 | 0.719172 |
|  | rs4758317 | 0.973835 | 0.074721 | 0.729685 |
|  | rs10849915 | 0.974 | 0.077899 | 0.741864 |
|  | rs2548145 | 1.022997 | 0.077123 | 0.762964 |
|  | rs886205 | 0.972075 | 0.09653 | 0.775486 |
|  | rs2228093 | 1.030546 | 0.113647 | 0.784978 |
|  | rs1864982 | 0.969677 | 0.110036 | 0.786122 |
|  | rs4761097 | 1.019799 | 0.076396 | 0.793541 |
|  | rs3930234 | 1.027422 | 0.106971 | 0.794989 |
|  | rs804292 | 0.977731 | 0.086237 | 0.798469 |
|  | rs6902771 | 1.018233 | 0.076269 | 0.80938 |
|  | rs2140418 | 0.979794 | 0.093359 | 0.830366 |
|  | rs284786 | 1.017594 | 0.083365 | 0.831409 |
|  | rs59972978 | 1.019368 | 0.101432 | 0.847125 |
|  | rs933769 | 0.981496 | 0.096119 | 0.848743 |
|  | rs768048 | 1.020742 | 0.109949 | 0.848841 |
|  | rs8040009 | 1.018344 | 0.099444 | 0.852326 |
|  | rs1497571 | 1.01287 | 0.076165 | 0.86496 |
|  | rs1573496 | 0.980438 | 0.123512 | 0.875387 |
|  | rs3131513 | 0.992192 | 0.07626 | 0.918763 |
|  | rs1380131 | 0.989149 | 0.128761 | 0.933207 |
|  | rs9512637 | 1.006367 | 0.077901 | 0.934651 |
|  | rs2810114 | 1.006441 | 0.084285 | 0.93889 |
|  | rs3819197 | 1.005723 | 0.087311 | 0.947589 |
|  | rs9656709 | 1.00415 | 0.076601 | 0.956703 |
|  | rs1353621 | 1.004121 | 0.077672 | 0.957597 |
|  | rs2380220 | 1.004308 | 0.105785 | 0.96745 |
|  | rs420817 | 1.002245 | 0.074759 | 0.976019 |
|  | rs9556711 | 1.003817 | 0.160701 | 0.981013 |
|  | rs3764435 | 1.001103 | 0.075577 | 0.988348 |
|  | rs2303317 | 1.000413 | 0.074706 | 0.995583 |
|  | rs1908556 | 1.00005 | 0.108274 | 0.99963 |
| cannabis consumption at 4 years after baseline | Mums PGRS | 5.491393 | 3.490326 | 0.00737 |
|  | rs1908556 | 0.675686 | 0.105988 | 0.012447 |
|  | rs1793257 | 1.614433 | 0.34204 | 0.023771 |
|  | rs10849915 | 1.244434 | 0.121888 | 0.025572 |
|  | rs768048 | 1.302027 | 0.163481 | 0.035555 |
|  | rs59972978 | 1.276211 | 0.150602 | 0.038755 |
|  | rs8062326 | 1.588195 | 0.357377 | 0.039801 |
|  | rs2140418 | 0.789768 | 0.101675 | 0.066761 |
|  | rs9556711 | 0.663039 | 0.158347 | 0.085318 |
|  | rs4293630 | 0.774246 | 0.117936 | 0.093005 |
|  | rs242938 | 0.678 | 0.157837 | 0.09506 |
|  | rs4478858 | 0.856949 | 0.082246 | 0.107725 |
|  | rs2380220 | 1.218594 | 0.153012 | 0.115379 |
|  | rs4770403 | 0.817494 | 0.104821 | 0.116047 |
|  | rs12311304 | 0.850189 | 0.08929 | 0.122265 |
|  | rs2188561 | 0.835515 | 0.098766 | 0.128454 |
|  | rs3930234 | 0.810207 | 0.113524 | 0.13308 |
|  | rs1229984 | 0.471655 | 0.239863 | 0.139481 |
|  | rs1000579 | 0.868444 | 0.084724 | 0.148228 |
|  | rs1353621 | 0.866919 | 0.085813 | 0.149099 |
|  | rs3819197 | 0.852134 | 0.097483 | 0.161895 |
|  | rs6943555 | 0.858594 | 0.096617 | 0.175466 |
|  | rs2548145 | 1.133115 | 0.10703 | 0.18582 |
|  | rs3131513 | 1.134076 | 0.10866 | 0.189129 |
|  | rs2100290 | 0.883806 | 0.083752 | 0.192423 |
|  | rs10253361 | 0.884382 | 0.084127 | 0.196488 |
|  | rs1789891 | 1.15389 | 0.140262 | 0.238972 |
|  | rs12472151 | 1.27195 | 0.262166 | 0.243179 |
|  | rs3762894 | 0.857741 | 0.114642 | 0.250918 |
|  | rs11851015 | 0.847652 | 0.123915 | 0.258201 |
|  | rs10908907 | 1.117506 | 0.118672 | 0.295468 |
|  | rs1318937 | 1.148574 | 0.153869 | 0.301132 |
|  | rs16985179 | 1.174708 | 0.183606 | 0.302916 |
|  | rs59677118 | 0.835115 | 0.14787 | 0.308857 |
|  | rs4758317 | 1.099184 | 0.105413 | 0.324084 |
|  | rs9512637 | 0.907279 | 0.089823 | 0.325682 |
|  | rs1109501 | 1.106638 | 0.117497 | 0.339912 |
|  | rs1042026 | 0.904775 | 0.095303 | 0.3421 |
|  | rs11724320 | 0.912184 | 0.092866 | 0.366617 |
|  | rs642899 | 1.101844 | 0.120744 | 0.376139 |
|  | rs279861 | 0.918601 | 0.088167 | 0.376368 |
|  | rs1824024 | 1.091697 | 0.110144 | 0.384534 |
|  | rs567926 | 0.920256 | 0.08846 | 0.387298 |
|  | rs8040009 | 0.897105 | 0.114329 | 0.394205 |
|  | rs4543123 | 1.097203 | 0.120283 | 0.397451 |
|  | rs1800759 | 0.924116 | 0.090101 | 0.418273 |
|  | rs4761097 | 1.079147 | 0.101775 | 0.419284 |
|  | rs804292 | 1.088173 | 0.117698 | 0.43466 |
|  | rs9636231 | 0.922586 | 0.098275 | 0.449394 |
|  | rs7144649 | 1.082928 | 0.120089 | 0.472494 |
|  | rs1876831 | 0.924266 | 0.105213 | 0.489034 |
|  | rs2228093 | 0.905427 | 0.130742 | 0.491443 |
|  | rs750338 | 0.931507 | 0.107918 | 0.540255 |
|  | rs9656709 | 1.057997 | 0.101088 | 0.555158 |
|  | rs13259667 | 1.100944 | 0.18851 | 0.57436 |
|  | rs1230165 | 0.934159 | 0.116445 | 0.5848 |
|  | rs2303317 | 1.052748 | 0.099357 | 0.585995 |
|  | rs2369955 | 0.923685 | 0.1352 | 0.587573 |
|  | rs284786 | 1.055426 | 0.108044 | 0.598221 |
|  | rs9825310 | 1.050182 | 0.099732 | 0.60614 |
|  | rs67031482 | 0.952286 | 0.090476 | 0.606842 |
|  | rs6716455 | 0.93227 | 0.130423 | 0.61615 |
|  | rs420817 | 0.956474 | 0.090228 | 0.637108 |
|  | rs3738443 | 1.057663 | 0.13072 | 0.65012 |
|  | rs1573496 | 0.936345 | 0.1497 | 0.680786 |
|  | rs36061340 | 1.074424 | 0.208577 | 0.711549 |
|  | rs933769 | 0.958489 | 0.118959 | 0.732646 |
|  | rs237238 | 0.936109 | 0.182064 | 0.734256 |
|  | rs9871864 | 1.032826 | 0.098435 | 0.734687 |
|  | rs2154294 | 1.027129 | 0.096564 | 0.77586 |
|  | rs6701037 | 0.974173 | 0.092296 | 0.782408 |
|  | rs2810114 | 0.977047 | 0.103992 | 0.827296 |
|  | rs36563 | 0.971526 | 0.129603 | 0.828566 |
|  | rs12388359 | 1.029928 | 0.142733 | 0.831497 |
|  | rs13160562 | 1.019567 | 0.10246 | 0.847097 |
|  | rs7590720 | 1.019649 | 0.105848 | 0.851312 |
|  | rs1344694 | 0.984762 | 0.099582 | 0.879307 |
|  | rs7553212 | 1.013732 | 0.101147 | 0.891276 |
|  | rs10893366 | 0.985378 | 0.127159 | 0.909123 |
|  | rs1353899 | 0.987 | 0.119877 | 0.914203 |
|  | rs1864982 | 0.986831 | 0.141157 | 0.926159 |
|  | rs6902771 | 0.991427 | 0.093306 | 0.927105 |
|  | rs886205 | 1.010801 | 0.124763 | 0.930642 |
|  | rs3764435 | 0.992609 | 0.094662 | 0.937998 |
|  | rs62202398 | 1.008571 | 0.195539 | 0.964889 |
|  | rs1497571 | 1.003586 | 0.094826 | 0.969776 |
|  | rs1380131 | 0.993815 | 0.163302 | 0.969883 |
|  | rs4440177 | 0.999124 | 0.099607 | 0.992988 |
|  | rs195204 | 0.999557 | 0.109524 | 0.996774 |
|  | rs2827312 | 0.999762 | 0.103639 | 0.998168 |
| cannabis consumption at 6 years after baseline | Mums PGRS | 7.312713 | 4.619746 | 0.001636 |
|  | rs8062326 | 2.030321 | 0.408189 | 0.000427 |
|  | rs1109501 | 1.346748 | 0.138212 | 0.003723 |
|  | rs642899 | 1.241545 | 0.131861 | 0.041638 |
|  | rs7590720 | 1.222733 | 0.123072 | 0.045734 |
|  | rs1908556 | 0.74405 | 0.112403 | 0.050344 |
|  | rs6902771 | 1.199936 | 0.11239 | 0.051657 |
|  | rs59972978 | 1.239428 | 0.147263 | 0.070826 |
|  | rs10849915 | 1.191384 | 0.116444 | 0.073185 |
|  | rs1344694 | 1.182323 | 0.116637 | 0.08956 |
|  | rs1864982 | 0.791932 | 0.121055 | 0.126985 |
|  | rs4770403 | 0.82229 | 0.105448 | 0.127063 |
|  | rs36563 | 0.813724 | 0.114285 | 0.142185 |
|  | rs4293630 | 0.805251 | 0.11988 | 0.145686 |
|  | rs10908907 | 0.85157 | 0.095491 | 0.151897 |
|  | rs2100290 | 0.878802 | 0.082925 | 0.17095 |
|  | rs1824024 | 1.144895 | 0.114065 | 0.17441 |
|  | rs16985179 | 1.228211 | 0.188239 | 0.179848 |
|  | rs1230165 | 0.843575 | 0.107718 | 0.182807 |
|  | rs1380131 | 0.798878 | 0.141356 | 0.204428 |
|  | rs242938 | 0.758923 | 0.165845 | 0.206826 |
|  | rs1229984 | 0.570307 | 0.260595 | 0.219069 |
|  | rs2810114 | 0.8767 | 0.094973 | 0.224478 |
|  | rs10893366 | 1.16023 | 0.142819 | 0.227302 |
|  | rs1318937 | 1.165337 | 0.152445 | 0.242139 |
|  | rs8040009 | 0.866793 | 0.110806 | 0.263445 |
|  | rs1800759 | 0.897734 | 0.087187 | 0.266648 |
|  | rs3762894 | 0.864564 | 0.114345 | 0.271181 |
|  | rs12311304 | 0.894774 | 0.092318 | 0.281201 |
|  | rs4478858 | 0.903795 | 0.085617 | 0.285613 |
|  | rs1793257 | 1.267152 | 0.286707 | 0.295352 |
|  | rs2188561 | 0.889063 | 0.102716 | 0.308779 |
|  | rs6943555 | 0.896795 | 0.099182 | 0.324664 |
|  | rs768048 | 1.135439 | 0.148107 | 0.330168 |
|  | rs2154294 | 1.092808 | 0.102066 | 0.341989 |
|  | rs9556711 | 0.812516 | 0.177679 | 0.342401 |
|  | rs1000579 | 0.913767 | 0.087706 | 0.347451 |
|  | rs279861 | 0.914066 | 0.087492 | 0.347867 |
|  | rs1353899 | 0.891298 | 0.110669 | 0.354033 |
|  | rs2369955 | 0.875236 | 0.128781 | 0.365101 |
|  | rs3930234 | 0.885147 | 0.12065 | 0.370753 |
|  | rs4440177 | 1.090525 | 0.106402 | 0.374444 |
|  | rs36061340 | 0.835499 | 0.178785 | 0.400967 |
|  | rs12388359 | 0.885821 | 0.129118 | 0.405536 |
|  | rs237238 | 0.852361 | 0.168713 | 0.419634 |
|  | rs2140418 | 0.908063 | 0.110955 | 0.429947 |
|  | rs420817 | 1.075783 | 0.100589 | 0.434662 |
|  | rs6701037 | 0.9295 | 0.087833 | 0.439119 |
|  | rs1789891 | 0.904958 | 0.118929 | 0.447309 |
|  | rs2827312 | 1.079352 | 0.111037 | 0.457918 |
|  | rs7144649 | 1.08378 | 0.120176 | 0.468104 |
|  | rs10253361 | 1.069748 | 0.100278 | 0.471981 |
|  | rs4758317 | 0.936612 | 0.090231 | 0.496657 |
|  | rs195204 | 1.074091 | 0.11501 | 0.504448 |
|  | rs59677118 | 0.894866 | 0.153512 | 0.517293 |
|  | rs3131513 | 1.062879 | 0.101787 | 0.524271 |
|  | rs9656709 | 1.059719 | 0.101262 | 0.543843 |
|  | rs13259667 | 0.894223 | 0.165439 | 0.545645 |
|  | rs9825310 | 1.057877 | 0.10027 | 0.552778 |
|  | rs567926 | 0.946265 | 0.090359 | 0.562984 |
|  | rs804292 | 0.938022 | 0.104692 | 0.566463 |
|  | rs13160562 | 0.943775 | 0.095284 | 0.566532 |
|  | rs2228093 | 0.921471 | 0.131838 | 0.567578 |
|  | rs1497571 | 1.054082 | 0.099334 | 0.576223 |
|  | rs3738443 | 0.931364 | 0.119336 | 0.578937 |
|  | rs12472151 | 0.880515 | 0.207193 | 0.588666 |
|  | rs9636231 | 0.944965 | 0.100185 | 0.593388 |
|  | rs4761097 | 1.044379 | 0.097923 | 0.643284 |
|  | rs3764435 | 1.041311 | 0.098499 | 0.668688 |
|  | rs2380220 | 1.053502 | 0.137032 | 0.688645 |
|  | rs11724320 | 0.960987 | 0.09636 | 0.691465 |
|  | rs1353621 | 0.962749 | 0.093791 | 0.696772 |
|  | rs4543123 | 1.037082 | 0.114427 | 0.741399 |
|  | rs62202398 | 0.943452 | 0.186426 | 0.768312 |
|  | rs1573496 | 1.045109 | 0.161187 | 0.77482 |
|  | rs6716455 | 0.963395 | 0.13251 | 0.786294 |
|  | rs9512637 | 0.975084 | 0.095005 | 0.795662 |
|  | rs9871864 | 1.024077 | 0.097206 | 0.802084 |
|  | rs284786 | 0.978314 | 0.101069 | 0.831936 |
|  | rs1042026 | 1.020848 | 0.104762 | 0.840651 |
|  | rs886205 | 1.023871 | 0.125591 | 0.847492 |
|  | rs1876831 | 0.978924 | 0.109522 | 0.848999 |
|  | rs3819197 | 0.985478 | 0.107828 | 0.893645 |
|  | rs67031482 | 1.009908 | 0.095947 | 0.917349 |
|  | rs7553212 | 1.00834 | 0.09965 | 0.933024 |
|  | rs750338 | 0.99181 | 0.112485 | 0.942194 |
|  | rs2303317 | 1.006646 | 0.09417 | 0.943548 |
|  | rs933769 | 0.993067 | 0.121504 | 0.954653 |
|  | rs11851015 | 0.99841 | 0.137623 | 0.990789 |
|  | rs2548145 | 1.000816 | 0.094552 | 0.993108 |
| cannabis consumption at 3 years post baseline | Mums PGRS | 2.516851 | 1.697022 | 0.171026 |
|  | rs8062326 | 1.83229 | 0.399397 | 0.005467 |
|  | rs9556711 | 0.424277 | 0.131073 | 0.005516 |
|  | rs4761097 | 1.2527 | 0.125017 | 0.023972 |
|  | rs4758317 | 1.227541 | 0.124929 | 0.043963 |
|  | rs1793257 | 1.517644 | 0.34464 | 0.066212 |
|  | rs1789891 | 1.257722 | 0.158817 | 0.069382 |
|  | rs1109501 | 1.207677 | 0.134261 | 0.08963 |
|  | rs1000579 | 0.839206 | 0.087271 | 0.091854 |
|  | rs59972978 | 1.228297 | 0.155033 | 0.10328 |
|  | rs2188561 | 0.815599 | 0.10312 | 0.106926 |
|  | rs1353621 | 0.844798 | 0.089347 | 0.11078 |
|  | rs1229984 | 0.395374 | 0.231347 | 0.112779 |
|  | rs3131513 | 1.17193 | 0.11855 | 0.116799 |
|  | rs1344694 | 1.17235 | 0.123442 | 0.131007 |
|  | rs2100290 | 0.859359 | 0.086453 | 0.131909 |
|  | rs750338 | 0.829001 | 0.10542 | 0.140285 |
|  | rs1230165 | 0.819551 | 0.112545 | 0.14731 |
|  | rs642899 | 1.176126 | 0.133706 | 0.153583 |
|  | rs242938 | 0.714105 | 0.170768 | 0.159103 |
|  | rs1318937 | 1.216372 | 0.169464 | 0.159746 |
|  | rs2380220 | 1.193267 | 0.159912 | 0.187336 |
|  | rs2548145 | 1.141038 | 0.114687 | 0.189292 |
|  | rs1800759 | 0.875296 | 0.090816 | 0.199236 |
|  | rs12311304 | 0.867844 | 0.095972 | 0.199936 |
|  | rs2140418 | 0.849085 | 0.113207 | 0.219818 |
|  | rs3764435 | 0.88316 | 0.089512 | 0.220238 |
|  | rs420817 | 1.128907 | 0.113277 | 0.226905 |
|  | rs4478858 | 0.884737 | 0.08976 | 0.227394 |
|  | rs10849915 | 1.128628 | 0.118128 | 0.24764 |
|  | rs2228093 | 0.834093 | 0.131356 | 0.249348 |
|  | rs36563 | 0.845515 | 0.125823 | 0.259466 |
|  | rs3930234 | 0.848666 | 0.123954 | 0.261242 |
|  | rs1908556 | 0.843371 | 0.129468 | 0.267142 |
|  | rs4293630 | 0.840008 | 0.132007 | 0.267253 |
|  | rs59677118 | 0.811365 | 0.154158 | 0.271243 |
|  | rs237238 | 0.786703 | 0.17238 | 0.273572 |
|  | rs12472151 | 1.261826 | 0.273906 | 0.28401 |
|  | rs11724320 | 0.890551 | 0.096671 | 0.285597 |
|  | rs804292 | 1.12437 | 0.127503 | 0.30127 |
|  | rs4770403 | 0.875958 | 0.116976 | 0.321325 |
|  | rs10253361 | 0.907362 | 0.091445 | 0.334742 |
|  | rs7590720 | 1.109651 | 0.12096 | 0.33984 |
|  | rs13259667 | 1.179697 | 0.207964 | 0.348531 |
|  | rs1864982 | 0.867311 | 0.136953 | 0.367302 |
|  | rs3738443 | 1.118055 | 0.144732 | 0.388666 |
|  | rs2154294 | 1.082888 | 0.108089 | 0.424995 |
|  | rs10908907 | 1.088155 | 0.122993 | 0.454791 |
|  | rs886205 | 0.903446 | 0.122848 | 0.455222 |
|  | rs10893366 | 0.905422 | 0.126911 | 0.478435 |
|  | rs16985179 | 1.125337 | 0.188717 | 0.481346 |
|  | rs9512637 | 0.933325 | 0.097767 | 0.510078 |
|  | rs6701037 | 0.935856 | 0.09423 | 0.510279 |
|  | rs11851015 | 0.906957 | 0.137216 | 0.518599 |
|  | rs2810114 | 0.932557 | 0.106762 | 0.541918 |
|  | rs8040009 | 0.922212 | 0.123488 | 0.545338 |
|  | rs4440177 | 1.061908 | 0.11159 | 0.567586 |
|  | rs933769 | 0.928163 | 0.123072 | 0.573972 |
|  | rs768048 | 1.080868 | 0.153288 | 0.583461 |
|  | rs1497571 | 1.053336 | 0.105075 | 0.602435 |
|  | rs1042026 | 0.944308 | 0.1048 | 0.605626 |
|  | rs9636231 | 0.949913 | 0.106959 | 0.648132 |
|  | rs9825310 | 1.041918 | 0.104872 | 0.683296 |
|  | rs36061340 | 0.915081 | 0.200175 | 0.684978 |
|  | rs2303317 | 0.962136 | 0.096223 | 0.699531 |
|  | rs567926 | 1.035985 | 0.104628 | 0.726301 |
|  | rs1876831 | 0.959088 | 0.115079 | 0.727734 |
|  | rs1573496 | 1.056199 | 0.172507 | 0.737803 |
|  | rs2369955 | 1.050554 | 0.156329 | 0.740328 |
|  | rs3819197 | 0.963904 | 0.11294 | 0.7537 |
|  | rs13160562 | 0.96739 | 0.103656 | 0.757005 |
|  | rs6902771 | 1.02979 | 0.10258 | 0.768227 |
|  | rs1380131 | 0.955316 | 0.170134 | 0.797423 |
|  | rs9871864 | 1.024665 | 0.103038 | 0.808543 |
|  | rs6716455 | 0.967123 | 0.142301 | 0.820271 |
|  | rs6943555 | 1.02469 | 0.116823 | 0.8306 |
|  | rs7553212 | 1.020551 | 0.107426 | 0.846759 |
|  | rs3762894 | 1.025154 | 0.136167 | 0.851634 |
|  | rs1824024 | 0.981711 | 0.106732 | 0.865183 |
|  | rs7144649 | 1.018833 | 0.121739 | 0.875915 |
|  | rs1353899 | 0.980299 | 0.126337 | 0.8773 |
|  | rs195204 | 0.983699 | 0.114502 | 0.887716 |
|  | rs2827312 | 1.014429 | 0.111073 | 0.895902 |
|  | rs62202398 | 1.024458 | 0.208925 | 0.905683 |
|  | rs279861 | 1.011979 | 0.102361 | 0.906286 |
|  | rs4543123 | 0.991248 | 0.118065 | 0.941164 |
|  | rs12388359 | 1.010438 | 0.150408 | 0.944384 |
|  | rs9656709 | 1.006262 | 0.101859 | 0.950823 |
|  | rs284786 | 0.99476 | 0.108756 | 0.96167 |
|  | rs67031482 | 0.998908 | 0.100426 | 0.991326 |
| cannabis consumption at 2 years post baseline | Mums PGRS | 3.874551 | 2.680419 | 0.05025 |
|  | rs1793257 | 1.7556 | 0.384804 | 0.010237 |
|  | rs242938 | 0.540253 | 0.148888 | 0.025471 |
|  | rs1908556 | 0.72248 | 0.121088 | 0.052437 |
|  | rs642899 | 1.246351 | 0.144057 | 0.056741 |
|  | rs10849915 | 1.224076 | 0.130166 | 0.057257 |
|  | rs4293630 | 0.736748 | 0.124789 | 0.071277 |
|  | rs8062326 | 1.543443 | 0.378708 | 0.076918 |
|  | rs12311304 | 0.816304 | 0.093721 | 0.077087 |
|  | rs12388359 | 1.267522 | 0.179679 | 0.094458 |
|  | rs7590720 | 1.195119 | 0.130549 | 0.102731 |
|  | rs59972978 | 1.232686 | 0.158835 | 0.104477 |
|  | rs9556711 | 0.661112 | 0.170575 | 0.108731 |
|  | rs1344694 | 1.18533 | 0.126783 | 0.111929 |
|  | rs279861 | 0.85029 | 0.089372 | 0.122839 |
|  | rs804292 | 1.179251 | 0.136266 | 0.153616 |
|  | rs36563 | 0.808701 | 0.124503 | 0.167847 |
|  | rs567926 | 0.86852 | 0.091297 | 0.179914 |
|  | rs1318937 | 1.206816 | 0.173003 | 0.189746 |
|  | rs9636231 | 0.861794 | 0.101519 | 0.206719 |
|  | rs2810114 | 0.861369 | 0.102563 | 0.210092 |
|  | rs9871864 | 0.877891 | 0.091589 | 0.211924 |
|  | rs1000579 | 0.876751 | 0.092677 | 0.213378 |
|  | rs13259667 | 1.246733 | 0.221203 | 0.213897 |
|  | rs1229984 | 0.559942 | 0.284923 | 0.254417 |
|  | rs6943555 | 0.870949 | 0.106443 | 0.258239 |
|  | rs1380131 | 0.801388 | 0.157517 | 0.259976 |
|  | rs12472151 | 1.275721 | 0.282795 | 0.271981 |
|  | rs10253361 | 0.892492 | 0.092418 | 0.272039 |
|  | rs1497571 | 1.11679 | 0.114865 | 0.28285 |
|  | rs237238 | 0.790294 | 0.176996 | 0.293328 |
|  | rs3131513 | 1.114797 | 0.116229 | 0.297261 |
|  | rs7553212 | 0.892365 | 0.098998 | 0.304651 |
|  | rs62202398 | 1.207712 | 0.237185 | 0.336564 |
|  | rs3764435 | 1.104001 | 0.114512 | 0.340143 |
|  | rs59677118 | 0.835106 | 0.159185 | 0.344488 |
|  | rs67031482 | 1.100108 | 0.113238 | 0.353983 |
|  | rs11851015 | 0.86643 | 0.137017 | 0.364605 |
|  | rs1109501 | 1.106089 | 0.127814 | 0.382896 |
|  | rs2303317 | 1.091257 | 0.111811 | 0.394031 |
|  | rs4758317 | 1.089478 | 0.114038 | 0.412937 |
|  | rs36061340 | 0.829157 | 0.193715 | 0.422614 |
|  | rs7144649 | 0.905637 | 0.114588 | 0.433414 |
|  | rs4770403 | 0.900977 | 0.122197 | 0.441992 |
|  | rs6716455 | 1.113332 | 0.159492 | 0.453613 |
|  | rs1789891 | 1.104158 | 0.148019 | 0.459836 |
|  | rs1353621 | 0.924113 | 0.099429 | 0.463252 |
|  | rs2228093 | 0.890401 | 0.141221 | 0.464225 |
|  | rs6701037 | 0.93007 | 0.096081 | 0.482826 |
|  | rs2140418 | 0.915836 | 0.121426 | 0.507261 |
|  | rs11724320 | 1.071209 | 0.115962 | 0.525146 |
|  | rs16985179 | 1.114513 | 0.192654 | 0.530527 |
|  | rs9825310 | 1.06237 | 0.109744 | 0.558083 |
|  | rs3930234 | 0.917892 | 0.134685 | 0.559296 |
|  | rs10908907 | 1.06907 | 0.124469 | 0.5662 |
|  | rs4761097 | 1.057702 | 0.108634 | 0.584929 |
|  | rs2548145 | 1.057485 | 0.108903 | 0.587307 |
|  | rs8040009 | 0.931825 | 0.127391 | 0.605511 |
|  | rs13160562 | 1.048065 | 0.113988 | 0.666001 |
|  | rs4478858 | 0.957357 | 0.098893 | 0.673113 |
|  | rs1573496 | 0.928833 | 0.162759 | 0.673527 |
|  | rs750338 | 0.950466 | 0.118705 | 0.684171 |
|  | rs9512637 | 1.039414 | 0.109751 | 0.714285 |
|  | rs1876831 | 1.043902 | 0.125685 | 0.721197 |
|  | rs1864982 | 0.947429 | 0.147955 | 0.729487 |
|  | rs1230165 | 1.045504 | 0.136341 | 0.732928 |
|  | rs6902771 | 1.034385 | 0.105675 | 0.740706 |
|  | rs284786 | 1.036114 | 0.115573 | 0.750442 |
|  | rs3819197 | 0.962674 | 0.11624 | 0.752731 |
|  | rs886205 | 1.035428 | 0.137416 | 0.793066 |
|  | rs2369955 | 1.038884 | 0.15823 | 0.80223 |
|  | rs3762894 | 0.9691 | 0.135354 | 0.82219 |
|  | rs768048 | 0.967663 | 0.146344 | 0.827932 |
|  | rs9656709 | 1.018014 | 0.10558 | 0.863324 |
|  | rs2154294 | 0.98465 | 0.100957 | 0.880074 |
|  | rs2827312 | 1.014853 | 0.113849 | 0.89544 |
|  | rs4543123 | 1.015322 | 0.123032 | 0.90014 |
|  | rs1353899 | 0.985789 | 0.12938 | 0.913158 |
|  | rs1042026 | 0.988808 | 0.111607 | 0.920571 |
|  | rs4440177 | 0.990001 | 0.107514 | 0.926272 |
|  | rs2100290 | 1.009391 | 0.103867 | 0.927622 |
|  | rs1824024 | 0.991493 | 0.110448 | 0.938868 |
|  | rs2380220 | 0.989699 | 0.143507 | 0.943072 |
|  | rs3738443 | 0.990404 | 0.13593 | 0.94399 |
|  | rs1800759 | 0.992914 | 0.103921 | 0.945827 |
|  | rs195204 | 0.992371 | 0.118282 | 0.948768 |
|  | rs2188561 | 1.007815 | 0.12312 | 0.949194 |
|  | rs933769 | 1.005098 | 0.134062 | 0.969589 |
|  | rs420817 | 0.998957 | 0.102708 | 0.9919 |
|  | rs10893366 | 1.000563 | 0.138639 | 0.996759 |
| cigarettes per day at 2 years post baseline | Mums PGRS | -1.0601 | 0.658355 | 0.10735 |
|  | rs1353899 | 0.410065 | 0.123868 | 0.000931 |
|  | rs7590720 | 0.307732 | 0.106779 | 0.003952 |
|  | rs1344694 | 0.290494 | 0.103695 | 0.005088 |
|  | rs237238 | -0.4339 | 0.193928 | 0.025258 |
|  | rs420817 | -0.20658 | 0.097595 | 0.034286 |
|  | rs6716455 | 0.272739 | 0.140944 | 0.052979 |
|  | rs67031482 | 0.1683 | 0.097715 | 0.085006 |
|  | rs1800759 | -0.16959 | 0.09923 | 0.087443 |
|  | rs2827312 | 0.175849 | 0.106716 | 0.099388 |
|  | rs1229984 | -0.58695 | 0.384829 | 0.127202 |
|  | rs1789891 | 0.197793 | 0.131303 | 0.131968 |
|  | rs750338 | -0.17155 | 0.117036 | 0.142694 |
|  | rs195204 | 0.16121 | 0.11279 | 0.152918 |
|  | rs1824024 | 0.149671 | 0.105662 | 0.156627 |
|  | rs4758317 | 0.131459 | 0.099781 | 0.187678 |
|  | rs4770403 | -0.16114 | 0.124892 | 0.196961 |
|  | rs2380220 | 0.157469 | 0.137236 | 0.251204 |
|  | rs6943555 | -0.12782 | 0.111925 | 0.253454 |
|  | rs2810114 | -0.1237 | 0.109261 | 0.257554 |
|  | rs1793257 | 0.289809 | 0.260514 | 0.265946 |
|  | rs4293630 | 0.159154 | 0.143892 | 0.268699 |
|  | rs1380131 | -0.18851 | 0.170996 | 0.270267 |
|  | rs3819197 | 0.123699 | 0.113408 | 0.275387 |
|  | rs1230165 | -0.13568 | 0.125445 | 0.27943 |
|  | rs59972978 | -0.13905 | 0.129441 | 0.282718 |
|  | rs2548145 | -0.10244 | 0.097894 | 0.295376 |
|  | rs3930234 | -0.14211 | 0.135908 | 0.295735 |
|  | rs13259667 | -0.18549 | 0.182977 | 0.3107 |
|  | rs2369955 | -0.14629 | 0.146657 | 0.318507 |
|  | rs1353621 | 0.10016 | 0.101006 | 0.32138 |
|  | rs9656709 | -0.09258 | 0.098657 | 0.348032 |
|  | rs59677118 | 0.157584 | 0.168838 | 0.350642 |
|  | rs9512637 | 0.091447 | 0.100807 | 0.364325 |
|  | rs642899 | 0.099685 | 0.115584 | 0.388441 |
|  | rs10253361 | 0.0839 | 0.0977 | 0.390476 |
|  | rs7144649 | -0.10011 | 0.117232 | 0.393131 |
|  | rs8040009 | 0.108307 | 0.127216 | 0.394566 |
|  | rs9556711 | -0.17391 | 0.205494 | 0.397392 |
|  | rs768048 | -0.1198 | 0.141917 | 0.398575 |
|  | rs36563 | -0.11282 | 0.135585 | 0.405353 |
|  | rs16985179 | -0.14011 | 0.170652 | 0.411617 |
|  | rs2154294 | 0.076043 | 0.097352 | 0.434734 |
|  | rs4761097 | 0.076099 | 0.09779 | 0.436457 |
|  | rs1864982 | 0.104441 | 0.145723 | 0.473553 |
|  | rs2228093 | 0.102575 | 0.145258 | 0.480092 |
|  | rs3762894 | 0.089674 | 0.130975 | 0.493555 |
|  | rs13160562 | 0.070978 | 0.104408 | 0.496627 |
|  | rs279861 | 0.067102 | 0.098759 | 0.496856 |
|  | rs36061340 | -0.13243 | 0.205465 | 0.519225 |
|  | rs3764435 | -0.06207 | 0.098551 | 0.528781 |
|  | rs1573496 | -0.10021 | 0.16193 | 0.535999 |
|  | rs1109501 | -0.06638 | 0.11214 | 0.553863 |
|  | rs9871864 | 0.058211 | 0.098461 | 0.554383 |
|  | rs62202398 | -0.11633 | 0.201301 | 0.563351 |
|  | rs804292 | 0.064836 | 0.113652 | 0.568352 |
|  | rs6902771 | -0.05209 | 0.097131 | 0.591753 |
|  | rs10893366 | -0.06425 | 0.131634 | 0.62547 |
|  | rs4478858 | 0.046388 | 0.097761 | 0.635137 |
|  | rs1042026 | 0.049754 | 0.106802 | 0.641322 |
|  | rs1000579 | -0.04343 | 0.098801 | 0.660237 |
|  | rs567926 | 0.041608 | 0.098795 | 0.673645 |
|  | rs933769 | 0.050161 | 0.126845 | 0.69251 |
|  | rs6701037 | 0.036796 | 0.097815 | 0.706784 |
|  | rs11851015 | 0.051825 | 0.142854 | 0.716765 |
|  | rs12472151 | 0.073442 | 0.231739 | 0.751306 |
|  | rs1908556 | -0.04022 | 0.141062 | 0.775555 |
|  | rs12311304 | -0.02967 | 0.104839 | 0.777167 |
|  | rs11724320 | 0.02943 | 0.104048 | 0.777293 |
|  | rs8062326 | 0.077026 | 0.281804 | 0.784597 |
|  | rs1318937 | 0.039294 | 0.145122 | 0.786572 |
|  | rs3738443 | 0.032477 | 0.129908 | 0.80259 |
|  | rs10908907 | 0.02326 | 0.112326 | 0.835951 |
|  | rs9636231 | 0.021412 | 0.108539 | 0.843611 |
|  | rs4543123 | 0.019154 | 0.115564 | 0.868357 |
|  | rs1876831 | 0.017557 | 0.115742 | 0.879428 |
|  | rs12388359 | 0.022081 | 0.145579 | 0.87944 |
|  | rs7553212 | -0.0153 | 0.103303 | 0.882285 |
|  | rs1497571 | 0.01233 | 0.097758 | 0.899634 |
|  | rs2140418 | -0.01463 | 0.122693 | 0.905082 |
|  | rs10849915 | -0.0112 | 0.103414 | 0.913741 |
|  | rs284786 | -0.01019 | 0.106445 | 0.9237 |
|  | rs242938 | -0.01521 | 0.201714 | 0.939909 |
|  | rs9825310 | -0.00611 | 0.098255 | 0.950429 |
|  | rs2100290 | 0.005461 | 0.097812 | 0.955475 |
|  | rs4440177 | 0.004699 | 0.103004 | 0.963614 |
|  | rs2303317 | -0.0022 | 0.097436 | 0.981981 |
|  | rs3131513 | -0.00156 | 0.100022 | 0.987535 |
|  | rs886205 | -0.00177 | 0.127398 | 0.988941 |
|  | rs2188561 | 0.001544 | 0.116238 | 0.989403 |
| cigarettes per day at 3 years post baseline | Mums PGRS | -0.88153 | 0.667037 | 0.186315 |
|  | rs237238 | -0.62982 | 0.19614 | 0.001322 |
|  | rs1344694 | 0.309041 | 0.106097 | 0.003582 |
|  | rs1353899 | 0.331037 | 0.126681 | 0.008971 |
|  | rs7590720 | 0.258628 | 0.109644 | 0.018334 |
|  | rs4758317 | 0.228902 | 0.101003 | 0.023434 |
|  | rs59677118 | 0.387869 | 0.173116 | 0.025057 |
|  | rs2810114 | -0.23286 | 0.111441 | 0.036659 |
|  | rs10253361 | 0.200451 | 0.098908 | 0.042699 |
|  | rs4770403 | -0.25372 | 0.126904 | 0.045571 |
|  | rs1573496 | -0.28495 | 0.164635 | 0.083488 |
|  | rs420817 | -0.15188 | 0.099108 | 0.125415 |
|  | rs642899 | 0.17333 | 0.116661 | 0.137346 |
|  | rs59972978 | -0.19412 | 0.131853 | 0.140953 |
|  | rs2188561 | -0.17114 | 0.118125 | 0.147394 |
|  | rs1789891 | 0.188623 | 0.133882 | 0.15887 |
|  | rs67031482 | 0.13057 | 0.099242 | 0.188284 |
|  | rs3764435 | -0.12853 | 0.099836 | 0.197965 |
|  | rs11724320 | 0.133586 | 0.105358 | 0.204825 |
|  | rs768048 | -0.17803 | 0.14378 | 0.215624 |
|  | rs1824024 | 0.132729 | 0.107245 | 0.215856 |
|  | rs36061340 | -0.25435 | 0.207851 | 0.221053 |
|  | rs3819197 | 0.138079 | 0.114608 | 0.228282 |
|  | rs9871864 | 0.118875 | 0.099338 | 0.231437 |
|  | rs2100290 | -0.11809 | 0.09916 | 0.233673 |
|  | rs3762894 | 0.154054 | 0.132247 | 0.244061 |
|  | rs3738443 | 0.148579 | 0.132449 | 0.261955 |
|  | rs1380131 | -0.19321 | 0.172723 | 0.263295 |
|  | rs2303317 | -0.11001 | 0.098656 | 0.264825 |
|  | rs750338 | -0.13246 | 0.119165 | 0.266315 |
|  | rs36563 | -0.15157 | 0.138481 | 0.273728 |
|  | rs1230165 | -0.13579 | 0.127344 | 0.286267 |
|  | rs195204 | 0.120628 | 0.114577 | 0.292427 |
|  | rs1793257 | 0.278225 | 0.266383 | 0.296275 |
|  | rs1864982 | 0.154783 | 0.148792 | 0.298215 |
|  | rs13160562 | 0.107991 | 0.105212 | 0.304696 |
|  | rs6716455 | 0.142821 | 0.143827 | 0.320708 |
|  | rs1800759 | -0.10018 | 0.100979 | 0.321149 |
|  | rs2154294 | 0.092116 | 0.098752 | 0.350923 |
|  | rs1042026 | 0.097641 | 0.108361 | 0.36755 |
|  | rs6943555 | -0.10029 | 0.113339 | 0.376213 |
|  | rs10908907 | 0.100478 | 0.113813 | 0.377323 |
|  | rs9636231 | -0.09682 | 0.1102 | 0.379619 |
|  | rs9556711 | -0.18482 | 0.210761 | 0.380534 |
|  | rs2140418 | -0.10978 | 0.12525 | 0.380751 |
|  | rs9825310 | -0.08703 | 0.099426 | 0.381419 |
|  | rs11851015 | -0.11884 | 0.144748 | 0.411653 |
|  | rs4478858 | 0.080198 | 0.099203 | 0.418848 |
|  | rs13259667 | -0.14605 | 0.185171 | 0.430283 |
|  | rs1497571 | -0.0739 | 0.098685 | 0.453919 |
|  | rs2369955 | -0.10926 | 0.149182 | 0.46394 |
|  | rs2827312 | 0.077096 | 0.108432 | 0.477081 |
|  | rs62202398 | -0.14128 | 0.203069 | 0.486593 |
|  | rs1229984 | -0.23476 | 0.378827 | 0.535455 |
|  | rs12388359 | -0.09142 | 0.148455 | 0.538027 |
|  | rs9656709 | -0.06145 | 0.100021 | 0.538973 |
|  | rs7553212 | 0.062824 | 0.104301 | 0.546951 |
|  | rs12472151 | 0.140187 | 0.2347 | 0.550304 |
|  | rs7144649 | -0.06891 | 0.11882 | 0.561973 |
|  | rs1876831 | 0.065439 | 0.117129 | 0.576374 |
|  | rs1000579 | -0.05457 | 0.10029 | 0.58637 |
|  | rs2380220 | -0.07365 | 0.140079 | 0.599064 |
|  | rs284786 | -0.05584 | 0.107951 | 0.604951 |
|  | rs1908556 | -0.06656 | 0.142777 | 0.641065 |
|  | rs3930234 | -0.06361 | 0.137414 | 0.643413 |
|  | rs2228093 | -0.06444 | 0.146482 | 0.659975 |
|  | rs933769 | 0.056203 | 0.12809 | 0.660824 |
|  | rs8040009 | 0.04517 | 0.129447 | 0.727132 |
|  | rs567926 | -0.03481 | 0.09986 | 0.72738 |
|  | rs6701037 | 0.034256 | 0.099222 | 0.729912 |
|  | rs1353621 | -0.03514 | 0.101932 | 0.730266 |
|  | rs6902771 | -0.03312 | 0.098576 | 0.736891 |
|  | rs804292 | -0.03806 | 0.115007 | 0.740659 |
|  | rs10849915 | -0.03457 | 0.105016 | 0.742023 |
|  | rs279861 | -0.03264 | 0.099848 | 0.743727 |
|  | rs4440177 | 0.031639 | 0.104864 | 0.762872 |
|  | rs886205 | -0.03888 | 0.130106 | 0.765058 |
|  | rs1318937 | -0.04161 | 0.146723 | 0.776744 |
|  | rs10893366 | 0.037862 | 0.134246 | 0.777918 |
|  | rs16985179 | -0.04573 | 0.172953 | 0.791445 |
|  | rs3131513 | 0.025675 | 0.101314 | 0.799946 |
|  | rs4543123 | -0.02791 | 0.117553 | 0.812354 |
|  | rs8062326 | 0.051709 | 0.281359 | 0.854185 |
|  | rs9512637 | 0.018411 | 0.102519 | 0.857475 |
|  | rs2548145 | -0.01161 | 0.099427 | 0.907071 |
|  | rs1109501 | -0.01216 | 0.114049 | 0.91512 |
|  | rs4293630 | -0.01155 | 0.146207 | 0.937012 |
|  | rs12311304 | -0.007 | 0.106443 | 0.947551 |
|  | rs242938 | 0.006913 | 0.205743 | 0.973197 |
|  | rs4761097 | -0.00068 | 0.099207 | 0.994556 |
| cigarettes per day at 4 years post baseline | Mums PGRS | -1.87201 | 0.709222 | 0.008302 |
|  | rs237238 | -0.76326 | 0.21025 | 0.000283 |
|  | rs1344694 | 0.317406 | 0.112105 | 0.004635 |
|  | rs7590720 | 0.278774 | 0.115788 | 0.016056 |
|  | rs1318937 | -0.352 | 0.155734 | 0.023805 |
|  | rs36563 | -0.32814 | 0.147245 | 0.025844 |
|  | rs420817 | -0.22492 | 0.104749 | 0.031775 |
|  | rs750338 | -0.26666 | 0.126384 | 0.034864 |
|  | rs3930234 | -0.30566 | 0.145669 | 0.035877 |
|  | rs2369955 | -0.32231 | 0.158459 | 0.041948 |
|  | rs2810114 | -0.2385 | 0.117738 | 0.042795 |
|  | rs59677118 | 0.350052 | 0.184418 | 0.057677 |
|  | rs4758317 | 0.203356 | 0.107177 | 0.057777 |
|  | rs1353899 | 0.252277 | 0.134658 | 0.061004 |
|  | rs1824024 | 0.209196 | 0.113865 | 0.066176 |
|  | rs4478858 | 0.183111 | 0.105308 | 0.082068 |
|  | rs3738443 | 0.242171 | 0.139975 | 0.083612 |
|  | rs10253361 | 0.17771 | 0.104909 | 0.090277 |
|  | rs6716455 | 0.256682 | 0.152131 | 0.091557 |
|  | rs3819197 | 0.20423 | 0.121809 | 0.093613 |
|  | rs11724320 | 0.166273 | 0.111388 | 0.135507 |
|  | rs1876831 | 0.160287 | 0.124094 | 0.196476 |
|  | rs1042026 | 0.145951 | 0.114505 | 0.202442 |
|  | rs933769 | 0.172728 | 0.136512 | 0.205765 |
|  | rs59972978 | -0.17116 | 0.139821 | 0.220909 |
|  | rs8062326 | 0.367313 | 0.304714 | 0.228034 |
|  | rs62202398 | -0.25492 | 0.216585 | 0.2392 |
|  | rs9636231 | 0.133056 | 0.11666 | 0.25406 |
|  | rs67031482 | 0.113324 | 0.105627 | 0.283329 |
|  | rs2228093 | 0.166223 | 0.156026 | 0.286714 |
|  | rs7144649 | -0.13106 | 0.125724 | 0.297195 |
|  | rs195204 | 0.125953 | 0.122046 | 0.302068 |
|  | rs2188561 | -0.12716 | 0.125782 | 0.312023 |
|  | rs2154294 | 0.104098 | 0.104492 | 0.319141 |
|  | rs1230165 | -0.13446 | 0.135877 | 0.322398 |
|  | rs13160562 | 0.10702 | 0.112209 | 0.340206 |
|  | rs36061340 | -0.20308 | 0.221158 | 0.358475 |
|  | rs1497571 | -0.09565 | 0.105186 | 0.363164 |
|  | rs1109501 | -0.10435 | 0.120923 | 0.38819 |
|  | rs8040009 | 0.110461 | 0.137313 | 0.421138 |
|  | rs284786 | -0.09049 | 0.114802 | 0.430575 |
|  | rs1908556 | 0.117748 | 0.151353 | 0.436586 |
|  | rs9656709 | -0.08149 | 0.10631 | 0.443338 |
|  | rs3764435 | -0.07834 | 0.106278 | 0.461027 |
|  | rs4440177 | -0.07875 | 0.110852 | 0.477457 |
|  | rs768048 | -0.1066 | 0.151307 | 0.481117 |
|  | rs2303317 | -0.07143 | 0.105047 | 0.496494 |
|  | rs279861 | 0.070545 | 0.106194 | 0.506494 |
|  | rs567926 | 0.068965 | 0.106347 | 0.516669 |
|  | rs4761097 | 0.065181 | 0.105317 | 0.53598 |
|  | rs1380131 | -0.11233 | 0.182014 | 0.537126 |
|  | rs13259667 | -0.1167 | 0.197677 | 0.554943 |
|  | rs4770403 | -0.07788 | 0.134181 | 0.561625 |
|  | rs4293630 | 0.086043 | 0.155002 | 0.578822 |
|  | rs2380220 | 0.078553 | 0.147873 | 0.595266 |
|  | rs1229984 | -0.21612 | 0.407169 | 0.59556 |
|  | rs11851015 | -0.08102 | 0.153965 | 0.598713 |
|  | rs886205 | -0.0717 | 0.137935 | 0.603179 |
|  | rs2827312 | 0.05908 | 0.115557 | 0.609165 |
|  | rs9512637 | -0.05348 | 0.108628 | 0.622469 |
|  | rs6902771 | -0.04997 | 0.104599 | 0.632867 |
|  | rs1573496 | -0.08182 | 0.173422 | 0.637078 |
|  | rs2548145 | -0.04758 | 0.105105 | 0.65077 |
|  | rs242938 | 0.100063 | 0.222488 | 0.652895 |
|  | rs1353621 | -0.04819 | 0.1079 | 0.655152 |
|  | rs10849915 | -0.047 | 0.111761 | 0.674115 |
|  | rs9825310 | -0.04298 | 0.105754 | 0.684461 |
|  | rs3131513 | 0.04313 | 0.107711 | 0.688844 |
|  | rs7553212 | 0.043656 | 0.111158 | 0.694512 |
|  | rs10893366 | -0.05383 | 0.143052 | 0.706714 |
|  | rs2140418 | 0.047014 | 0.133169 | 0.724056 |
|  | rs16985179 | -0.05881 | 0.184431 | 0.749843 |
|  | rs6943555 | 0.038352 | 0.120474 | 0.750226 |
|  | rs12311304 | 0.035209 | 0.113323 | 0.756031 |
|  | rs3762894 | 0.041648 | 0.141408 | 0.768359 |
|  | rs642899 | 0.036099 | 0.124864 | 0.7725 |
|  | rs2100290 | -0.0282 | 0.105209 | 0.788699 |
|  | rs1000579 | -0.02809 | 0.106735 | 0.792433 |
|  | rs804292 | 0.029332 | 0.122641 | 0.810972 |
|  | rs1789891 | 0.0331 | 0.141164 | 0.814616 |
|  | rs9556711 | 0.048086 | 0.22385 | 0.829913 |
|  | rs12388359 | 0.027861 | 0.156404 | 0.858615 |
|  | rs1864982 | 0.025141 | 0.158631 | 0.874074 |
|  | rs12472151 | -0.03511 | 0.251706 | 0.889064 |
|  | rs10908907 | 0.016626 | 0.121284 | 0.890964 |
|  | rs1800759 | -0.01292 | 0.107564 | 0.90439 |
|  | rs9871864 | 0.006259 | 0.106033 | 0.952932 |
|  | rs6701037 | 0.005415 | 0.105444 | 0.959039 |
|  | rs4543123 | 0.001619 | 0.124688 | 0.989638 |
|  | rs1793257 | -0.00346 | 0.282691 | 0.990239 |
| cigarettes per day at 6 years post baseline | Mums PGRS | -1.26945 | 0.718058 | 0.077078 |
|  | rs4758317 | 0.274554 | 0.109027 | 0.011795 |
|  | rs237238 | -0.52896 | 0.211813 | 0.012514 |
|  | rs3819197 | 0.302953 | 0.123493 | 0.014159 |
|  | rs1824024 | 0.263098 | 0.115208 | 0.022391 |
|  | rs420817 | -0.2386 | 0.106085 | 0.0245 |
|  | rs11724320 | 0.250062 | 0.113204 | 0.027178 |
|  | rs1573496 | -0.39168 | 0.177989 | 0.027767 |
|  | rs1344694 | 0.24692 | 0.114654 | 0.031272 |
|  | rs750338 | -0.24493 | 0.128342 | 0.056341 |
|  | rs1042026 | 0.218346 | 0.116748 | 0.061452 |
|  | rs36061340 | -0.41734 | 0.22538 | 0.064068 |
|  | rs7590720 | 0.215134 | 0.118308 | 0.068998 |
|  | rs10253361 | 0.18648 | 0.106619 | 0.080286 |
|  | rs59677118 | 0.289012 | 0.187051 | 0.122323 |
|  | rs8062326 | 0.458898 | 0.304782 | 0.132155 |
|  | rs10893366 | -0.21859 | 0.145633 | 0.133371 |
|  | rs12388359 | 0.234753 | 0.158838 | 0.139423 |
|  | rs13259667 | -0.29487 | 0.201371 | 0.143114 |
|  | rs36563 | -0.21552 | 0.148269 | 0.146065 |
|  | rs2810114 | -0.16079 | 0.119157 | 0.177203 |
|  | rs2369955 | -0.21148 | 0.159506 | 0.184885 |
|  | rs1353899 | 0.179818 | 0.136395 | 0.187381 |
|  | rs6943555 | -0.15565 | 0.122237 | 0.2029 |
|  | rs12311304 | 0.145946 | 0.114665 | 0.203085 |
|  | rs1800759 | -0.13836 | 0.108857 | 0.203727 |
|  | rs3762894 | 0.180294 | 0.142682 | 0.206371 |
|  | rs3930234 | -0.17596 | 0.149087 | 0.237906 |
|  | rs1109501 | -0.14417 | 0.122976 | 0.24106 |
|  | rs3764435 | -0.12477 | 0.107267 | 0.244768 |
|  | rs4770403 | -0.15443 | 0.137375 | 0.260956 |
|  | rs1793257 | 0.31212 | 0.284067 | 0.271875 |
|  | rs9556711 | -0.23864 | 0.227913 | 0.295071 |
|  | rs9512637 | -0.11515 | 0.11005 | 0.295392 |
|  | rs6716455 | 0.159114 | 0.154674 | 0.303619 |
|  | rs195204 | 0.126807 | 0.123637 | 0.305062 |
|  | rs284786 | 0.11909 | 0.116869 | 0.308201 |
|  | rs67031482 | 0.108961 | 0.107881 | 0.312493 |
|  | rs2303317 | -0.09629 | 0.106186 | 0.364502 |
|  | rs2188561 | -0.11445 | 0.127006 | 0.367533 |
|  | rs4478858 | 0.09205 | 0.106659 | 0.388117 |
|  | rs1908556 | -0.12813 | 0.154116 | 0.405758 |
|  | rs2100290 | 0.088228 | 0.10692 | 0.409271 |
|  | rs8040009 | 0.1136 | 0.139493 | 0.41543 |
|  | rs1864982 | 0.120807 | 0.159947 | 0.450074 |
|  | rs7144649 | 0.093239 | 0.128354 | 0.467581 |
|  | rs1230165 | -0.09984 | 0.137547 | 0.467921 |
|  | rs933769 | 0.094094 | 0.138936 | 0.498251 |
|  | rs1876831 | 0.08102 | 0.126395 | 0.521517 |
|  | rs62202398 | -0.13865 | 0.219584 | 0.527769 |
|  | rs1000579 | -0.06679 | 0.107885 | 0.535885 |
|  | rs16985179 | 0.109023 | 0.186169 | 0.558136 |
|  | rs6902771 | -0.0579 | 0.106553 | 0.586834 |
|  | rs59972978 | -0.07235 | 0.142315 | 0.611172 |
|  | rs9656709 | -0.05321 | 0.108495 | 0.623844 |
|  | rs279861 | -0.05259 | 0.108122 | 0.626672 |
|  | rs2228093 | 0.075936 | 0.158625 | 0.632143 |
|  | rs1497571 | -0.05078 | 0.107124 | 0.635497 |
|  | rs642899 | 0.059383 | 0.126767 | 0.639471 |
|  | rs242938 | 0.096635 | 0.220978 | 0.661888 |
|  | rs2140418 | -0.05007 | 0.134878 | 0.710474 |
|  | rs2827312 | 0.042939 | 0.117603 | 0.715024 |
|  | rs4543123 | -0.04605 | 0.126368 | 0.715529 |
|  | rs1789891 | -0.05007 | 0.144806 | 0.729512 |
|  | rs7553212 | 0.038083 | 0.112293 | 0.734504 |
|  | rs1353621 | -0.0369 | 0.109991 | 0.737243 |
|  | rs2380220 | 0.048595 | 0.149972 | 0.745918 |
|  | rs1318937 | 0.049376 | 0.156234 | 0.751975 |
|  | rs10908907 | 0.038509 | 0.122076 | 0.752418 |
|  | rs9825310 | 0.033869 | 0.107533 | 0.752791 |
|  | rs4293630 | 0.046879 | 0.156257 | 0.764167 |
|  | rs3131513 | -0.03119 | 0.109345 | 0.775476 |
|  | rs9871864 | 0.029595 | 0.107887 | 0.783841 |
|  | rs4440177 | 0.030602 | 0.112186 | 0.785026 |
|  | rs2154294 | 0.027446 | 0.106087 | 0.795857 |
|  | rs567926 | -0.02693 | 0.108078 | 0.803194 |
|  | rs3738443 | 0.031781 | 0.14179 | 0.822647 |
|  | rs11851015 | -0.0323 | 0.156282 | 0.836279 |
|  | rs9636231 | -0.02225 | 0.118897 | 0.851557 |
|  | rs1229984 | 0.075324 | 0.412088 | 0.854965 |
|  | rs1380131 | -0.03354 | 0.184734 | 0.855926 |
|  | rs6701037 | 0.01764 | 0.106951 | 0.868995 |
|  | rs4761097 | 0.016361 | 0.106737 | 0.878172 |
|  | rs10849915 | -0.01703 | 0.113434 | 0.880652 |
|  | rs768048 | -0.01805 | 0.154597 | 0.907066 |
|  | rs13160562 | 0.01177 | 0.113432 | 0.917359 |
|  | rs2548145 | -0.01038 | 0.107162 | 0.9228 |
|  | rs12472151 | -0.02086 | 0.25469 | 0.934731 |
|  | rs804292 | 0.009083 | 0.125052 | 0.942098 |
|  | rs886205 | 0.002217 | 0.140119 | 0.987374 |
| mothers education | Mums PGRS | 1.809025 | 0.431684 | 0.012986 |
|  | rs4543123 | 0.910264 | 0.037939 | 0.024081 |
|  | rs886205 | 0.902894 | 0.041592 | 0.02659 |
|  | rs2188561 | 0.913692 | 0.038252 | 0.031082 |
|  | rs1908556 | 0.896335 | 0.045583 | 0.031394 |
|  | rs1230165 | 1.090081 | 0.049251 | 0.056259 |
|  | rs284786 | 1.073364 | 0.041174 | 0.064945 |
|  | rs3819197 | 0.930407 | 0.038228 | 0.079161 |
|  | rs804292 | 1.072561 | 0.043668 | 0.085337 |
|  | rs1353621 | 1.06352 | 0.038448 | 0.08847 |
|  | rs567926 | 0.941997 | 0.033446 | 0.092384 |
|  | rs279861 | 0.942506 | 0.033381 | 0.09455 |
|  | rs11724320 | 0.943959 | 0.035645 | 0.126688 |
|  | rs3131513 | 1.05536 | 0.037855 | 0.133054 |
|  | rs10893366 | 0.932127 | 0.044408 | 0.140125 |
|  | rs2228093 | 0.926301 | 0.049028 | 0.148066 |
|  | rs62202398 | 1.106479 | 0.078312 | 0.152821 |
|  | rs1042026 | 0.948227 | 0.036665 | 0.169174 |
|  | rs2380220 | 0.934193 | 0.046616 | 0.172514 |
|  | rs6902771 | 1.047392 | 0.036528 | 0.184282 |
|  | rs2140418 | 0.943272 | 0.041527 | 0.184652 |
|  | rs2369955 | 0.933376 | 0.049419 | 0.192853 |
|  | rs13259667 | 0.926299 | 0.061199 | 0.246547 |
|  | rs11851015 | 0.942563 | 0.048765 | 0.252896 |
|  | rs8062326 | 1.120017 | 0.111543 | 0.255079 |
|  | rs1229984 | 1.163726 | 0.158776 | 0.266426 |
|  | rs2100290 | 0.961712 | 0.03385 | 0.267358 |
|  | rs2548145 | 1.037553 | 0.036473 | 0.294309 |
|  | rs237238 | 0.931254 | 0.064596 | 0.304516 |
|  | rs1789891 | 1.049203 | 0.049957 | 0.313099 |
|  | rs9636231 | 0.963895 | 0.037633 | 0.346264 |
|  | rs420817 | 0.967641 | 0.033869 | 0.34732 |
|  | rs13160562 | 0.965132 | 0.036466 | 0.347572 |
|  | rs10849915 | 0.965482 | 0.036121 | 0.347771 |
|  | rs1876831 | 0.962347 | 0.040066 | 0.356605 |
|  | rs8040009 | 0.962672 | 0.044155 | 0.406866 |
|  | rs4758317 | 0.97117 | 0.034966 | 0.416508 |
|  | rs750338 | 0.966917 | 0.040525 | 0.422144 |
|  | rs4761097 | 1.028394 | 0.03618 | 0.426119 |
|  | rs12388359 | 0.961126 | 0.05008 | 0.446685 |
|  | rs6716455 | 0.961932 | 0.049368 | 0.449506 |
|  | rs36061340 | 0.946386 | 0.069186 | 0.450985 |
|  | rs6943555 | 0.97188 | 0.039145 | 0.478846 |
|  | rs1380131 | 1.043002 | 0.06346 | 0.488943 |
|  | rs1573496 | 0.961209 | 0.057146 | 0.505752 |
|  | rs1824024 | 1.023911 | 0.039011 | 0.535127 |
|  | rs9556711 | 0.95564 | 0.071631 | 0.544955 |
|  | rs6701037 | 0.98007 | 0.034488 | 0.567271 |
|  | rs3762894 | 0.973975 | 0.045675 | 0.573904 |
|  | rs1344694 | 1.020882 | 0.03823 | 0.581022 |
|  | rs195204 | 1.022134 | 0.041237 | 0.587367 |
|  | rs7553212 | 0.980582 | 0.036508 | 0.598413 |
|  | rs1318937 | 1.025927 | 0.053017 | 0.620372 |
|  | rs4478858 | 0.983202 | 0.034704 | 0.631275 |
|  | rs1793257 | 1.045607 | 0.097958 | 0.634047 |
|  | rs1864982 | 1.024923 | 0.053738 | 0.638697 |
|  | rs4770403 | 1.020426 | 0.046038 | 0.654033 |
|  | rs59972978 | 0.980213 | 0.046105 | 0.67092 |
|  | rs1109501 | 1.017014 | 0.041074 | 0.676143 |
|  | rs12311304 | 0.984715 | 0.037184 | 0.683333 |
|  | rs36563 | 1.019465 | 0.049807 | 0.693147 |
|  | rs4293630 | 0.979866 | 0.051005 | 0.695989 |
|  | rs3738443 | 0.982229 | 0.045907 | 0.70124 |
|  | rs2827312 | 1.014867 | 0.039059 | 0.70138 |
|  | rs9825310 | 0.986931 | 0.034753 | 0.708708 |
|  | rs1000579 | 1.012607 | 0.036233 | 0.726238 |
|  | rs2810114 | 0.986588 | 0.038612 | 0.730092 |
|  | rs3930234 | 0.984771 | 0.047935 | 0.752555 |
|  | rs12472151 | 1.024779 | 0.084755 | 0.767264 |
|  | rs768048 | 0.985202 | 0.050194 | 0.769802 |
|  | rs16985179 | 1.016922 | 0.062476 | 0.78475 |
|  | rs7590720 | 1.010491 | 0.038875 | 0.786186 |
|  | rs9871864 | 0.990559 | 0.035259 | 0.78986 |
|  | rs4440177 | 1.009529 | 0.037497 | 0.79846 |
|  | rs59677118 | 1.01526 | 0.061583 | 0.802833 |
|  | rs642899 | 0.990029 | 0.040559 | 0.806766 |
|  | rs242938 | 1.017619 | 0.0748 | 0.812184 |
|  | rs9512637 | 0.991408 | 0.036385 | 0.814115 |
|  | rs1353899 | 0.989519 | 0.04436 | 0.814183 |
|  | rs3764435 | 1.00796 | 0.036101 | 0.824798 |
|  | rs2303317 | 1.007454 | 0.035633 | 0.833705 |
|  | rs7144649 | 0.991804 | 0.042008 | 0.845944 |
|  | rs10908907 | 1.007667 | 0.040828 | 0.850481 |
|  | rs2154294 | 0.993837 | 0.034713 | 0.859523 |
|  | rs67031482 | 1.005226 | 0.035562 | 0.882875 |
|  | rs1800759 | 0.994823 | 0.035583 | 0.884626 |
|  | rs1497571 | 0.994955 | 0.034999 | 0.885661 |
|  | rs10253361 | 1.00462 | 0.035506 | 0.896229 |
|  | rs933769 | 0.994414 | 0.045432 | 0.902418 |
|  | rs9656709 | 0.997248 | 0.035319 | 0.937986 |
| opiate and cocaine use at 3 years post baseline | Mums PGRS | 0.154907 | 0.444889 | 0.516109 |
|  | rs2154294 | 0.244079 | 0.134747 | 0.010633 |
|  | rs8040009 | 2.69874 | 1.228932 | 0.029246 |
|  | rs7144649 | 2.378724 | 1.039783 | 0.047429 |
|  | rs4761097 | 2.188624 | 0.966811 | 0.076206 |
|  | rs750338 | 0.166793 | 0.170812 | 0.080315 |
|  | rs2100290 | 0.465025 | 0.213309 | 0.09508 |
|  | rs9871864 | 0.472013 | 0.222576 | 0.111362 |
|  | rs10849915 | 0.421903 | 0.235283 | 0.12175 |
|  | rs1230165 | 0.215029 | 0.219778 | 0.13264 |
|  | rs36061340 | 2.485741 | 1.563771 | 0.147778 |
|  | rs2188561 | 0.347562 | 0.257385 | 0.153558 |
|  | rs10893366 | 0.236092 | 0.242361 | 0.159666 |
|  | rs3738443 | 1.886702 | 0.893333 | 0.180002 |
|  | rs3819197 | 1.789087 | 0.782951 | 0.183772 |
|  | rs6943555 | 1.711235 | 0.748708 | 0.219503 |
|  | rs195204 | 0.494829 | 0.306184 | 0.255535 |
|  | rs1109501 | 1.660617 | 0.744263 | 0.257781 |
|  | rs7590720 | 0.533125 | 0.296584 | 0.258198 |
|  | rs2369955 | 0.319078 | 0.327859 | 0.266256 |
|  | rs2303317 | 0.613823 | 0.271478 | 0.269812 |
|  | rs2548145 | 0.615949 | 0.273913 | 0.275845 |
|  | rs1000579 | 0.599295 | 0.284252 | 0.280381 |
|  | rs9636231 | 0.54916 | 0.305089 | 0.280651 |
|  | rs1876831 | 0.510789 | 0.31879 | 0.281747 |
|  | rs1497571 | 1.574446 | 0.680315 | 0.293504 |
|  | rs4478858 | 0.623234 | 0.283583 | 0.298734 |
|  | rs36563 | 1.684231 | 0.853095 | 0.303386 |
|  | rs2228093 | 1.718192 | 0.92009 | 0.312121 |
|  | rs7553212 | 0.599946 | 0.303412 | 0.312376 |
|  | rs2810114 | 1.542189 | 0.68383 | 0.328584 |
|  | rs13160562 | 1.515768 | 0.645734 | 0.328907 |
|  | rs1344694 | 0.611479 | 0.310017 | 0.331959 |
|  | rs9656709 | 1.524274 | 0.668044 | 0.336162 |
|  | rs1789891 | 0.499263 | 0.371121 | 0.350064 |
|  | rs3930234 | 0.490217 | 0.374243 | 0.350391 |
|  | rs4440177 | 1.406769 | 0.607274 | 0.429165 |
|  | rs3764435 | 0.713971 | 0.312447 | 0.441372 |
|  | rs279861 | 0.714572 | 0.318223 | 0.450459 |
|  | rs1042026 | 1.390184 | 0.61261 | 0.454712 |
|  | rs10908907 | 0.663098 | 0.36607 | 0.456768 |
|  | rs59677118 | 0.461598 | 0.479972 | 0.457199 |
|  | rs1353899 | 0.657494 | 0.411654 | 0.503025 |
|  | rs567926 | 0.74301 | 0.330127 | 0.50378 |
|  | rs1908556 | 0.629751 | 0.465029 | 0.531163 |
|  | rs768048 | 0.633372 | 0.469257 | 0.537618 |
|  | rs11851015 | 1.407881 | 0.782558 | 0.538266 |
|  | rs1800759 | 1.277015 | 0.545467 | 0.567005 |
|  | rs3131513 | 1.277477 | 0.54721 | 0.567529 |
|  | rs12388359 | 0.668014 | 0.496676 | 0.58739 |
|  | rs4758317 | 1.261554 | 0.546521 | 0.591732 |
|  | rs933769 | 0.719153 | 0.445131 | 0.594288 |
|  | rs1864982 | 0.675042 | 0.502428 | 0.597505 |
|  | rs59972978 | 1.312973 | 0.690519 | 0.604634 |
|  | rs1353621 | 0.795876 | 0.362863 | 0.616539 |
|  | rs6902771 | 0.81887 | 0.352574 | 0.642566 |
|  | rs237238 | 0.635966 | 0.655109 | 0.660383 |
|  | rs12311304 | 0.814416 | 0.389684 | 0.6679 |
|  | rs2380220 | 1.26764 | 0.7073 | 0.670809 |
|  | rs9512637 | 0.830926 | 0.378478 | 0.684282 |
|  | rs284786 | 0.834729 | 0.402659 | 0.708038 |
|  | rs8062326 | 1.445986 | 1.462202 | 0.715334 |
|  | rs6701037 | 1.162092 | 0.49557 | 0.724639 |
|  | rs242938 | 0.708692 | 0.730062 | 0.738187 |
|  | rs3762894 | 0.823398 | 0.504823 | 0.751288 |
|  | rs804292 | 0.867723 | 0.445236 | 0.782151 |
|  | rs9556711 | 0.754114 | 0.775799 | 0.783836 |
|  | rs11724320 | 0.88683 | 0.410792 | 0.795419 |
|  | rs13259667 | 1.189195 | 0.885978 | 0.816088 |
|  | rs2827312 | 0.926751 | 0.437223 | 0.871904 |
|  | rs16985179 | 0.884095 | 0.688838 | 0.87437 |
|  | rs9825310 | 0.936316 | 0.402753 | 0.878417 |
|  | rs2140418 | 0.926643 | 0.513153 | 0.890575 |
|  | rs67031482 | 1.048985 | 0.449073 | 0.911053 |
|  | rs886205 | 1.054852 | 0.581407 | 0.922818 |
|  | rs1318937 | 1.057199 | 0.656258 | 0.9286 |
|  | rs4770403 | 0.955334 | 0.529804 | 0.934333 |
|  | rs420817 | 1.030251 | 0.439841 | 0.944348 |
|  | rs10253361 | 1.025963 | 0.437219 | 0.952039 |
|  | rs4293630 | 1.03527 | 0.643098 | 0.955501 |
|  | rs1824024 | 0.977881 | 0.453238 | 0.961511 |
|  | rs12472151 | 0.958196 | 0.990056 | 0.967033 |
|  | rs642899 | 0.982718 | 0.497313 | 0.972519 |
|  | rs4543123 | 0.986569 | 0.501236 | 0.978766 |
|  | rs6716455 | 0.992216 | 0.616522 | 0.989966 |
| opiate and cocaine use at 4 years post baseline | Mums PGRS | 1.451756 | 3.838101 | 0.887869 |
|  | rs2380220 | 3.074155 | 1.290041 | 0.007447 |
|  | rs36061340 | 3.827233 | 1.939874 | 0.008098 |
|  | rs1109501 | 2.49494 | 0.989114 | 0.021103 |
|  | rs642899 | 2.472409 | 0.978421 | 0.022174 |
|  | rs4440177 | 2.334117 | 0.912206 | 0.030091 |
|  | rs2100290 | 0.447871 | 0.190645 | 0.059157 |
|  | rs13160562 | 1.881835 | 0.729934 | 0.103104 |
|  | rs10908907 | 1.872953 | 0.753604 | 0.118858 |
|  | rs11724320 | 0.461361 | 0.230171 | 0.121003 |
|  | rs6701037 | 1.853228 | 0.747223 | 0.125997 |
|  | rs8040009 | 1.875654 | 0.830192 | 0.155316 |
|  | rs3738443 | 1.826857 | 0.798864 | 0.168194 |
|  | rs4761097 | 0.561198 | 0.237653 | 0.172522 |
|  | rs4543123 | 1.752609 | 0.725545 | 0.175292 |
|  | rs933769 | 0.373692 | 0.275295 | 0.181502 |
|  | rs8062326 | 2.617001 | 1.92852 | 0.191732 |
|  | rs1497571 | 0.583978 | 0.240733 | 0.191948 |
|  | rs67031482 | 1.690682 | 0.68509 | 0.194998 |
|  | rs750338 | 0.456896 | 0.280661 | 0.202254 |
|  | rs10849915 | 0.550565 | 0.260752 | 0.20762 |
|  | rs420817 | 1.639662 | 0.660255 | 0.219445 |
|  | rs10893366 | 0.416201 | 0.307938 | 0.236108 |
|  | rs3930234 | 0.408046 | 0.309516 | 0.237315 |
|  | rs6902771 | 0.627367 | 0.257063 | 0.255193 |
|  | rs1800759 | 1.554372 | 0.609391 | 0.260572 |
|  | rs4478858 | 1.54208 | 0.602442 | 0.267561 |
|  | rs3131513 | 1.543419 | 0.606782 | 0.269624 |
|  | rs9636231 | 0.577953 | 0.289378 | 0.273515 |
|  | rs1353621 | 0.628448 | 0.276243 | 0.290634 |
|  | rs10253361 | 0.649856 | 0.266668 | 0.293564 |
|  | rs2548145 | 0.669509 | 0.269922 | 0.31966 |
|  | rs59972978 | 1.579037 | 0.730607 | 0.323496 |
|  | rs2140418 | 0.550447 | 0.337691 | 0.330469 |
|  | rs2303317 | 1.45738 | 0.578395 | 0.342611 |
|  | rs59677118 | 0.379784 | 0.396894 | 0.35423 |
|  | rs12311304 | 0.655668 | 0.305527 | 0.365021 |
|  | rs1908556 | 0.521823 | 0.382676 | 0.375116 |
|  | rs2228093 | 1.560815 | 0.792002 | 0.38028 |
|  | rs11851015 | 1.511094 | 0.758252 | 0.410666 |
|  | rs886205 | 0.607936 | 0.373301 | 0.417651 |
|  | rs1000579 | 0.717848 | 0.301478 | 0.429922 |
|  | rs6716455 | 1.478415 | 0.736343 | 0.432463 |
|  | rs9871864 | 0.727952 | 0.295624 | 0.434291 |
|  | rs2369955 | 0.560868 | 0.415358 | 0.434889 |
|  | rs9656709 | 0.761764 | 0.305961 | 0.498085 |
|  | rs2827312 | 1.315673 | 0.551943 | 0.513133 |
|  | rs1230165 | 1.35177 | 0.62663 | 0.515554 |
|  | rs804292 | 1.322907 | 0.571008 | 0.516783 |
|  | rs9512637 | 0.782528 | 0.330898 | 0.561966 |
|  | rs7553212 | 1.256988 | 0.505363 | 0.56943 |
|  | rs2188561 | 1.268751 | 0.556989 | 0.587674 |
|  | rs1876831 | 0.774212 | 0.386168 | 0.607908 |
|  | rs1353899 | 1.264283 | 0.59588 | 0.618801 |
|  | rs4770403 | 0.767529 | 0.419412 | 0.628256 |
|  | rs3764435 | 1.204844 | 0.478733 | 0.639074 |
|  | rs36563 | 0.75824 | 0.462336 | 0.649913 |
|  | rs2810114 | 0.819725 | 0.379504 | 0.667649 |
|  | rs7144649 | 0.811234 | 0.404718 | 0.674978 |
|  | rs4758317 | 1.179797 | 0.469272 | 0.67764 |
|  | rs1573496 | 0.740731 | 0.544349 | 0.682988 |
|  | rs16985179 | 0.730006 | 0.564913 | 0.684248 |
|  | rs1864982 | 1.243194 | 0.682089 | 0.691548 |
|  | rs284786 | 1.174584 | 0.490817 | 0.700173 |
|  | rs3819197 | 1.176545 | 0.51356 | 0.709543 |
|  | rs242938 | 1.284358 | 0.953141 | 0.735948 |
|  | rs12388359 | 1.197904 | 0.653696 | 0.740718 |
|  | rs567926 | 1.121644 | 0.442653 | 0.771142 |
|  | rs1380131 | 0.812612 | 0.601908 | 0.779371 |
|  | rs1824024 | 1.118737 | 0.46673 | 0.787975 |
|  | rs4293630 | 0.847773 | 0.521358 | 0.788288 |
|  | rs1344694 | 0.907065 | 0.386521 | 0.818943 |
|  | rs768048 | 1.130382 | 0.613716 | 0.82141 |
|  | rs1318937 | 0.872219 | 0.535349 | 0.823735 |
|  | rs279861 | 1.084661 | 0.428541 | 0.837032 |
|  | rs12472151 | 0.810003 | 0.834706 | 0.837978 |
|  | rs6943555 | 0.911675 | 0.420217 | 0.840995 |
|  | rs2154294 | 1.079863 | 0.421661 | 0.844007 |
|  | rs1042026 | 1.085704 | 0.457314 | 0.845222 |
|  | rs237238 | 1.142297 | 0.852964 | 0.85859 |
|  | rs1789891 | 0.90831 | 0.494362 | 0.859747 |
|  | rs7590720 | 1.065921 | 0.455709 | 0.881299 |
|  | rs195204 | 0.937375 | 0.434183 | 0.888958 |
|  | rs9825310 | 0.961555 | 0.380272 | 0.921034 |
|  | rs3762894 | 0.948197 | 0.509404 | 0.921128 |
|  | rs13259667 | 1.000273 | 0.738211 | 0.999705 |
| opiate and cocaine use at 6 years post baseline | Mums PGRS | 0.265862 | 0.637721 | 0.580748 |
|  | rs9636231 | 0.246333 | 0.150263 | 0.021628 |
|  | rs1109501 | 2.274838 | 0.822261 | 0.022974 |
|  | rs10849915 | 0.434436 | 0.19913 | 0.068931 |
|  | rs2154294 | 0.50318 | 0.191011 | 0.070412 |
|  | rs8040009 | 2.031707 | 0.798137 | 0.071155 |
|  | rs13160562 | 0.419749 | 0.202418 | 0.071836 |
|  | rs3738443 | 1.938141 | 0.753999 | 0.08895 |
|  | rs67031482 | 0.536512 | 0.201758 | 0.097765 |
|  | rs6902771 | 0.534797 | 0.203509 | 0.100031 |
|  | rs1824024 | 0.473933 | 0.216763 | 0.10256 |
|  | rs3131513 | 1.756289 | 0.62308 | 0.112397 |
|  | rs2369955 | 0.212448 | 0.216303 | 0.128146 |
|  | rs36061340 | 2.268047 | 1.221771 | 0.128459 |
|  | rs3762894 | 0.347908 | 0.252905 | 0.146382 |
|  | rs420817 | 1.692548 | 0.615547 | 0.147906 |
|  | rs2100290 | 0.595641 | 0.21814 | 0.157144 |
|  | rs1497571 | 1.622983 | 0.586061 | 0.179894 |
|  | rs2188561 | 0.493296 | 0.263306 | 0.185543 |
|  | rs9656709 | 0.618324 | 0.22944 | 0.195125 |
|  | rs10253361 | 1.581405 | 0.560839 | 0.196249 |
|  | rs933769 | 0.467904 | 0.284259 | 0.21124 |
|  | rs2548145 | 0.639073 | 0.234633 | 0.222653 |
|  | rs2303317 | 0.649567 | 0.236005 | 0.235031 |
|  | rs4478858 | 1.496349 | 0.524786 | 0.250484 |
|  | rs59677118 | 0.30969 | 0.320728 | 0.2577 |
|  | rs12388359 | 0.439017 | 0.321526 | 0.260999 |
|  | rs1789891 | 0.51712 | 0.31519 | 0.27926 |
|  | rs2380220 | 1.590893 | 0.685997 | 0.281594 |
|  | rs10893366 | 0.518787 | 0.317314 | 0.283296 |
|  | rs2228093 | 1.624506 | 0.735024 | 0.283555 |
|  | rs3930234 | 0.513883 | 0.321319 | 0.286991 |
|  | rs3819197 | 0.599864 | 0.28938 | 0.289428 |
|  | rs4543123 | 1.498671 | 0.57364 | 0.290517 |
|  | rs9825310 | 0.679737 | 0.25001 | 0.2939 |
|  | rs13259667 | 1.721351 | 0.930296 | 0.314931 |
|  | rs4770403 | 0.615015 | 0.330201 | 0.365254 |
|  | rs4440177 | 1.376211 | 0.489877 | 0.369662 |
|  | rs1042026 | 0.68599 | 0.292207 | 0.376265 |
|  | rs6943555 | 1.367161 | 0.515416 | 0.406796 |
|  | rs804292 | 0.68374 | 0.313591 | 0.407149 |
|  | rs1230165 | 0.64355 | 0.343052 | 0.408329 |
|  | rs279861 | 0.748859 | 0.275289 | 0.43145 |
|  | rs6701037 | 1.317179 | 0.466701 | 0.436847 |
|  | rs886205 | 0.66411 | 0.355798 | 0.444874 |
|  | rs62202398 | 0.457835 | 0.470223 | 0.446857 |
|  | rs11724320 | 0.740055 | 0.293839 | 0.448349 |
|  | rs7144649 | 1.34718 | 0.533397 | 0.451642 |
|  | rs12311304 | 1.314501 | 0.479057 | 0.453044 |
|  | rs242938 | 0.480982 | 0.490857 | 0.47325 |
|  | rs567926 | 0.778968 | 0.285564 | 0.495637 |
|  | rs1573496 | 0.61659 | 0.446865 | 0.504638 |
|  | rs9556711 | 0.515623 | 0.525762 | 0.515946 |
|  | rs4761097 | 1.255782 | 0.441406 | 0.517009 |
|  | rs7590720 | 1.274944 | 0.478328 | 0.51735 |
|  | rs3764435 | 0.799031 | 0.286637 | 0.531699 |
|  | rs36563 | 1.311969 | 0.587834 | 0.544503 |
|  | rs1864982 | 0.694706 | 0.423136 | 0.549803 |
|  | rs1344694 | 1.231236 | 0.454086 | 0.572732 |
|  | rs2810114 | 1.223377 | 0.463591 | 0.594694 |
|  | rs7553212 | 1.197859 | 0.434063 | 0.618333 |
|  | rs16985179 | 1.305994 | 0.732306 | 0.634001 |
|  | rs59972978 | 0.789171 | 0.400594 | 0.640899 |
|  | rs2827312 | 1.18992 | 0.456348 | 0.650256 |
|  | rs9871864 | 1.170089 | 0.417259 | 0.659583 |
|  | rs12472151 | 1.347981 | 1.000951 | 0.687584 |
|  | rs1318937 | 1.210962 | 0.585303 | 0.692083 |
|  | rs10908907 | 1.156436 | 0.453511 | 0.710921 |
|  | rs284786 | 0.866198 | 0.34509 | 0.718435 |
|  | rs11851015 | 1.177064 | 0.576366 | 0.739188 |
|  | rs4758317 | 1.125431 | 0.404974 | 0.74262 |
|  | rs768048 | 1.145701 | 0.559294 | 0.780531 |
|  | rs6716455 | 0.875902 | 0.469663 | 0.804824 |
|  | rs1876831 | 0.901367 | 0.388286 | 0.809508 |
|  | rs1000579 | 1.081174 | 0.383156 | 0.825691 |
|  | rs1793257 | 0.819443 | 0.838652 | 0.84573 |
|  | rs1908556 | 0.904831 | 0.479974 | 0.850461 |
|  | rs9512637 | 1.068961 | 0.387122 | 0.853902 |
|  | rs237238 | 0.874051 | 0.647699 | 0.855848 |
|  | rs1800759 | 0.936207 | 0.340929 | 0.856355 |
|  | rs4293630 | 0.918828 | 0.49289 | 0.874604 |
|  | rs642899 | 0.948419 | 0.404424 | 0.901162 |
|  | rs195204 | 1.048688 | 0.42467 | 0.906547 |
|  | rs1353899 | 0.959588 | 0.439519 | 0.928237 |
|  | rs2140418 | 0.973498 | 0.439109 | 0.952516 |
|  | rs1353621 | 1.019612 | 0.37139 | 0.957475 |
|  | rs750338 | 0.983795 | 0.421346 | 0.96957 |
|  | rs1380131 | 1.014807 | 0.618244 | 0.980752 |
|  | rs8062326 | 1.009572 | 1.019136 | 0.992471 |
| **Offspring** | | | | |
| ADHD at age 10 years | Offspring PGRS | 2.381227 | 2.024039 | 0.307384 |
|  | rs2548145 | 0.567054 | 0.106397 | 0.002499 |
|  | rs3738443 | 1.700746 | 0.355114 | 0.010977 |
|  | rs1908556 | 1.693564 | 0.365484 | 0.014637 |
|  | rs6902771 | 0.672117 | 0.124654 | 0.032168 |
|  | rs3819197 | 1.50306 | 0.287081 | 0.03288 |
|  | rs1789891 | 0.532434 | 0.162899 | 0.039388 |
|  | rs1109501 | 1.453592 | 0.278797 | 0.051157 |
|  | rs195204 | 0.633068 | 0.151468 | 0.056031 |
|  | rs59972978 | 0.598281 | 0.167747 | 0.066932 |
|  | rs12311304 | 1.387519 | 0.256085 | 0.075971 |
|  | rs7553212 | 0.71599 | 0.144973 | 0.098945 |
|  | rs9636231 | 1.360755 | 0.25667 | 0.10245 |
|  | rs750338 | 0.684451 | 0.164663 | 0.115035 |
|  | rs12388359 | 0.661779 | 0.184519 | 0.138714 |
|  | rs59677118 | 0.552956 | 0.22533 | 0.145967 |
|  | rs1800759 | 1.294266 | 0.23036 | 0.14727 |
|  | rs4761097 | 1.291011 | 0.230739 | 0.152966 |
|  | rs13160562 | 1.301618 | 0.245085 | 0.161515 |
|  | rs9556711 | 0.502879 | 0.257558 | 0.179546 |
|  | rs642899 | 0.741165 | 0.171283 | 0.194937 |
|  | rs16985179 | 1.415778 | 0.382186 | 0.197762 |
|  | rs12472151 | 0.469094 | 0.275757 | 0.197864 |
|  | rs4770403 | 0.724469 | 0.183666 | 0.203596 |
|  | rs2380220 | 1.339892 | 0.308555 | 0.203885 |
|  | rs242938 | 1.459133 | 0.445761 | 0.216157 |
|  | rs9825310 | 0.800472 | 0.14607 | 0.222613 |
|  | rs6943555 | 1.271233 | 0.25075 | 0.22373 |
|  | rs4293630 | 0.712191 | 0.210237 | 0.250239 |
|  | rs9656709 | 0.816984 | 0.149544 | 0.269464 |
|  | rs1793257 | 1.531206 | 0.595058 | 0.272936 |
|  | rs1042026 | 1.230062 | 0.232676 | 0.273666 |
|  | rs2140418 | 1.255692 | 0.271385 | 0.29211 |
|  | rs1353899 | 1.245301 | 0.262507 | 0.298015 |
|  | rs4543123 | 1.231736 | 0.247924 | 0.300439 |
|  | rs933769 | 1.2489 | 0.269967 | 0.303848 |
|  | rs1824024 | 0.828542 | 0.164427 | 0.343247 |
|  | rs8062326 | 0.517157 | 0.36944 | 0.355973 |
|  | rs1864982 | 0.763556 | 0.2242 | 0.358226 |
|  | rs11724320 | 0.837628 | 0.164301 | 0.36637 |
|  | rs1353621 | 1.179673 | 0.217374 | 0.369863 |
|  | rs1000579 | 1.162813 | 0.210302 | 0.404255 |
|  | rs3930234 | 1.212652 | 0.289362 | 0.419079 |
|  | rs10893366 | 0.813498 | 0.209486 | 0.42281 |
|  | rs420817 | 1.139463 | 0.204766 | 0.467526 |
|  | rs3762894 | 1.158731 | 0.2623 | 0.515161 |
|  | rs3764435 | 0.893272 | 0.159687 | 0.52781 |
|  | rs13259667 | 0.813219 | 0.299393 | 0.574394 |
|  | rs4758317 | 0.902981 | 0.165499 | 0.577653 |
|  | rs36563 | 0.873029 | 0.227448 | 0.602229 |
|  | rs886205 | 1.120837 | 0.25192 | 0.611776 |
|  | rs2154294 | 1.092455 | 0.196462 | 0.622923 |
|  | rs1876831 | 0.901971 | 0.195078 | 0.633338 |
|  | rs4440177 | 0.913191 | 0.175432 | 0.636427 |
|  | rs9512637 | 1.089452 | 0.201734 | 0.643592 |
|  | rs10849915 | 0.916145 | 0.177119 | 0.650543 |
|  | rs10253361 | 0.928361 | 0.168425 | 0.682001 |
|  | rs10908907 | 0.920329 | 0.194187 | 0.693963 |
|  | rs1229984 | 1.221023 | 0.629432 | 0.69848 |
|  | rs1318937 | 0.902721 | 0.248032 | 0.709539 |
|  | rs768048 | 1.094809 | 0.285394 | 0.728232 |
|  | rs3131513 | 1.064624 | 0.193065 | 0.729854 |
|  | rs7590720 | 1.065151 | 0.210299 | 0.74921 |
|  | rs8040009 | 1.072704 | 0.248973 | 0.762359 |
|  | rs284786 | 0.946139 | 0.186665 | 0.778995 |
|  | rs9871864 | 1.051672 | 0.188864 | 0.779062 |
|  | rs1230165 | 1.064005 | 0.241144 | 0.784283 |
|  | rs4478858 | 1.049634 | 0.190669 | 0.789724 |
|  | rs1380131 | 0.919493 | 0.292198 | 0.791687 |
|  | rs62202398 | 0.907867 | 0.360799 | 0.807838 |
|  | rs2228093 | 1.0485 | 0.279651 | 0.859062 |
|  | rs6716455 | 1.047532 | 0.27609 | 0.860146 |
|  | rs2810114 | 0.965289 | 0.19579 | 0.861729 |
|  | rs1573496 | 1.048639 | 0.311454 | 0.872955 |
|  | rs2303317 | 0.972241 | 0.173481 | 0.874637 |
|  | rs279861 | 1.026425 | 0.184675 | 0.884741 |
|  | rs6701037 | 0.975444 | 0.176125 | 0.890479 |
|  | rs7144649 | 1.028825 | 0.218889 | 0.893745 |
|  | rs11851015 | 0.96638 | 0.256027 | 0.897294 |
|  | rs804292 | 0.976985 | 0.199179 | 0.909071 |
|  | rs237238 | 1.033563 | 0.363446 | 0.925205 |
|  | rs2827312 | 1.017724 | 0.201256 | 0.929206 |
|  | rs2188561 | 1.018985 | 0.215964 | 0.929292 |
|  | rs67031482 | 0.988157 | 0.176887 | 0.946934 |
|  | rs36061340 | 1.023883 | 0.380569 | 0.949369 |
|  | rs2100290 | 1.008635 | 0.180244 | 0.961627 |
|  | rs2369955 | 0.996541 | 0.273178 | 0.989916 |
|  | rs1344694 | 1.001792 | 0.194657 | 0.99265 |
|  | rs1497571 | 0.999322 | 0.178946 | 0.996978 |
|  | rs567926 | 1.000442 | 0.180848 | 0.99805 |
| ADHD at age 13 | Offspring PGRS | 1.397999 | 1.302491 | 0.719139 |
|  | rs4761097 | 1.676773 | 0.330655 | 0.008765 |
|  | rs886205 | 1.743305 | 0.381128 | 0.011016 |
|  | rs11724320 | 0.568151 | 0.132727 | 0.015515 |
|  | rs642899 | 0.5006 | 0.143671 | 0.015909 |
|  | rs1876831 | 0.566931 | 0.154444 | 0.037229 |
|  | rs933769 | 1.5416 | 0.342706 | 0.051539 |
|  | rs2369955 | 1.639361 | 0.419673 | 0.053495 |
|  | rs1908556 | 1.586275 | 0.379666 | 0.05389 |
|  | rs3764435 | 0.721179 | 0.14297 | 0.099186 |
|  | rs1353621 | 1.376615 | 0.272353 | 0.106188 |
|  | rs2303317 | 0.728379 | 0.144561 | 0.110289 |
|  | rs242938 | 1.625873 | 0.517836 | 0.126996 |
|  | rs9656709 | 0.741466 | 0.148847 | 0.136209 |
|  | rs59972978 | 0.639598 | 0.192103 | 0.136756 |
|  | rs2548145 | 0.755988 | 0.149212 | 0.156408 |
|  | rs6943555 | 1.326332 | 0.28354 | 0.186474 |
|  | rs1380131 | 0.573256 | 0.242133 | 0.187722 |
|  | rs2100290 | 0.783042 | 0.154443 | 0.21498 |
|  | rs12388359 | 0.695519 | 0.20395 | 0.215623 |
|  | rs8062326 | 0.293647 | 0.295414 | 0.223206 |
|  | rs2810114 | 0.762202 | 0.178622 | 0.246573 |
|  | rs6902771 | 0.797183 | 0.15724 | 0.250477 |
|  | rs1864982 | 1.3395 | 0.348346 | 0.261025 |
|  | rs10253361 | 0.806228 | 0.161255 | 0.281533 |
|  | rs1824024 | 1.242144 | 0.251101 | 0.283426 |
|  | rs6716455 | 0.698894 | 0.233947 | 0.284504 |
|  | rs1353899 | 0.762231 | 0.203932 | 0.310201 |
|  | rs237238 | 0.629482 | 0.291673 | 0.317829 |
|  | rs10893366 | 1.267624 | 0.306595 | 0.32685 |
|  | rs1800759 | 1.202332 | 0.234352 | 0.344478 |
|  | rs13160562 | 1.215369 | 0.251207 | 0.345343 |
|  | rs1793257 | 0.512381 | 0.365679 | 0.348786 |
|  | rs12472151 | 1.448113 | 0.575032 | 0.351112 |
|  | rs4293630 | 1.271342 | 0.331127 | 0.35666 |
|  | rs12311304 | 0.81807 | 0.179701 | 0.360636 |
|  | rs59677118 | 0.711778 | 0.280797 | 0.388786 |
|  | rs4770403 | 1.219277 | 0.286601 | 0.398982 |
|  | rs2154294 | 0.854292 | 0.168734 | 0.425264 |
|  | rs3819197 | 1.189898 | 0.260485 | 0.427063 |
|  | rs7144649 | 1.173156 | 0.261689 | 0.474037 |
|  | rs4440177 | 0.861253 | 0.182943 | 0.481942 |
|  | rs36061340 | 0.726441 | 0.336632 | 0.490395 |
|  | rs10908907 | 1.159315 | 0.251568 | 0.495712 |
|  | rs2827312 | 1.148272 | 0.243693 | 0.514744 |
|  | rs11851015 | 1.182059 | 0.317547 | 0.533539 |
|  | rs2228093 | 1.180982 | 0.329412 | 0.550927 |
|  | rs8040009 | 0.856536 | 0.229676 | 0.563589 |
|  | rs1497571 | 0.893445 | 0.174367 | 0.563727 |
|  | rs62202398 | 0.765134 | 0.356826 | 0.565946 |
|  | rs4758317 | 1.115972 | 0.221854 | 0.580989 |
|  | rs1573496 | 0.831766 | 0.292718 | 0.600681 |
|  | rs768048 | 1.146749 | 0.318046 | 0.621506 |
|  | rs1230165 | 0.880053 | 0.229087 | 0.623534 |
|  | rs1000579 | 1.100158 | 0.218259 | 0.630415 |
|  | rs2380220 | 1.134335 | 0.299084 | 0.632611 |
|  | rs195204 | 1.107612 | 0.24406 | 0.642762 |
|  | rs1318937 | 0.867382 | 0.266079 | 0.64279 |
|  | rs1229984 | 0.720817 | 0.518522 | 0.649045 |
|  | rs3131513 | 1.091227 | 0.215067 | 0.657791 |
|  | rs9825310 | 0.921837 | 0.181118 | 0.678702 |
|  | rs420817 | 0.92263 | 0.180738 | 0.681019 |
|  | rs67031482 | 0.923289 | 0.180383 | 0.682889 |
|  | rs1109501 | 0.911423 | 0.208858 | 0.685669 |
|  | rs1789891 | 1.106734 | 0.282453 | 0.691097 |
|  | rs284786 | 0.918914 | 0.199509 | 0.696916 |
|  | rs4478858 | 1.076923 | 0.211861 | 0.706393 |
|  | rs7590720 | 0.92199 | 0.203719 | 0.713181 |
|  | rs279861 | 1.074344 | 0.209799 | 0.713459 |
|  | rs7553212 | 1.061074 | 0.21657 | 0.771472 |
|  | rs567926 | 1.057911 | 0.20734 | 0.773929 |
|  | rs3738443 | 0.934724 | 0.249322 | 0.800211 |
|  | rs10849915 | 0.948241 | 0.199868 | 0.80093 |
|  | rs9556711 | 1.103318 | 0.438531 | 0.804621 |
|  | rs2188561 | 1.043702 | 0.239685 | 0.852241 |
|  | rs3762894 | 0.958153 | 0.25342 | 0.871601 |
|  | rs1344694 | 0.968745 | 0.204713 | 0.880556 |
|  | rs9871864 | 1.029157 | 0.202436 | 0.883835 |
|  | rs6701037 | 0.972684 | 0.190841 | 0.887743 |
|  | rs4543123 | 1.032661 | 0.237041 | 0.888651 |
|  | rs3930234 | 0.966205 | 0.269388 | 0.901864 |
|  | rs804292 | 1.02606 | 0.223673 | 0.906055 |
|  | rs9512637 | 0.983463 | 0.200973 | 0.934964 |
|  | rs2140418 | 0.990657 | 0.247764 | 0.97006 |
|  | rs13259667 | 0.988958 | 0.366564 | 0.976102 |
|  | rs750338 | 0.995635 | 0.232505 | 0.985055 |
|  | rs9636231 | 0.995944 | 0.216843 | 0.985107 |
|  | rs1042026 | 0.996465 | 0.213752 | 0.98683 |
|  | rs16985179 | 1.004708 | 0.337141 | 0.988833 |
|  | rs36563 | 0.999204 | 0.27169 | 0.997662 |
| ADHD at age 15 | Offspring PGRS | 1.466928 | 1.832444 | 0.759042 |
|  | rs1908556 | 2.312034 | 0.677596 | 0.004239 |
|  | rs4761097 | 1.909487 | 0.51362 | 0.016184 |
|  | rs3762894 | 0.267732 | 0.158406 | 0.025931 |
|  | rs10253361 | 0.539193 | 0.154542 | 0.031156 |
|  | rs1318937 | 1.981445 | 0.633307 | 0.032395 |
|  | rs7553212 | 0.520658 | 0.168081 | 0.043205 |
|  | rs4293630 | 0.334732 | 0.199231 | 0.065949 |
|  | rs59972978 | 0.426542 | 0.202298 | 0.072412 |
|  | rs284786 | 0.557596 | 0.182679 | 0.074598 |
|  | rs16985179 | 1.867134 | 0.671984 | 0.082753 |
|  | rs642899 | 0.5393 | 0.20478 | 0.103913 |
|  | rs1000579 | 1.478103 | 0.392956 | 0.141604 |
|  | rs1800759 | 1.460382 | 0.377764 | 0.143195 |
|  | rs2810114 | 0.617311 | 0.207727 | 0.151711 |
|  | rs2188561 | 1.480365 | 0.421935 | 0.168713 |
|  | rs8040009 | 0.571977 | 0.234107 | 0.172277 |
|  | rs9556711 | 0.260392 | 0.263545 | 0.183693 |
|  | rs36563 | 0.537728 | 0.251494 | 0.184672 |
|  | rs1793257 | 1.946834 | 1.009355 | 0.198803 |
|  | rs4478858 | 0.699926 | 0.195963 | 0.20255 |
|  | rs1353621 | 1.38316 | 0.366366 | 0.220721 |
|  | rs1042026 | 0.6843 | 0.216621 | 0.230767 |
|  | rs7590720 | 1.390728 | 0.386993 | 0.235901 |
|  | rs2369955 | 1.480475 | 0.531471 | 0.274405 |
|  | rs6716455 | 1.468539 | 0.516906 | 0.27496 |
|  | rs12472151 | 0.346766 | 0.351592 | 0.296223 |
|  | rs9512637 | 0.738874 | 0.214234 | 0.296608 |
|  | rs11724320 | 0.737426 | 0.218765 | 0.304548 |
|  | rs13160562 | 0.730052 | 0.226322 | 0.310134 |
|  | rs1344694 | 1.316828 | 0.362848 | 0.317876 |
|  | rs3930234 | 1.392516 | 0.462613 | 0.318918 |
|  | rs3131513 | 0.756645 | 0.212546 | 0.320846 |
|  | rs2827312 | 1.320688 | 0.374688 | 0.326877 |
|  | rs3819197 | 0.7326 | 0.246313 | 0.354727 |
|  | rs420817 | 0.792101 | 0.211163 | 0.381974 |
|  | rs2100290 | 1.234217 | 0.327642 | 0.427947 |
|  | rs1824024 | 1.237955 | 0.334875 | 0.430044 |
|  | rs3764435 | 0.817344 | 0.215436 | 0.444145 |
|  | rs12311304 | 1.226886 | 0.339746 | 0.460264 |
|  | rs9636231 | 1.222752 | 0.343744 | 0.474387 |
|  | rs67031482 | 1.200937 | 0.316306 | 0.486934 |
|  | rs9656709 | 1.201428 | 0.321045 | 0.492245 |
|  | rs279861 | 1.186627 | 0.308988 | 0.511089 |
|  | rs13259667 | 0.68103 | 0.402213 | 0.515405 |
|  | rs1380131 | 0.716667 | 0.372925 | 0.522031 |
|  | rs567926 | 1.181092 | 0.308351 | 0.523786 |
|  | rs10908907 | 0.830534 | 0.262006 | 0.556124 |
|  | rs237238 | 0.707711 | 0.423116 | 0.56309 |
|  | rs933769 | 0.830324 | 0.299375 | 0.606058 |
|  | rs1789891 | 1.180279 | 0.399128 | 0.624028 |
|  | rs6701037 | 0.879244 | 0.233753 | 0.628338 |
|  | rs4440177 | 0.885337 | 0.253615 | 0.670733 |
|  | rs242938 | 0.777491 | 0.461113 | 0.671298 |
|  | rs750338 | 1.129087 | 0.347007 | 0.692812 |
|  | rs11851015 | 1.157163 | 0.430223 | 0.694603 |
|  | rs1573496 | 0.831586 | 0.393477 | 0.696715 |
|  | rs59677118 | 0.823606 | 0.413981 | 0.699435 |
|  | rs1109501 | 1.106749 | 0.324721 | 0.729573 |
|  | rs2303317 | 0.916357 | 0.240691 | 0.739471 |
|  | rs2140418 | 1.113513 | 0.364259 | 0.742396 |
|  | rs62202398 | 0.831861 | 0.501764 | 0.760215 |
|  | rs9825310 | 0.924858 | 0.244674 | 0.767785 |
|  | rs804292 | 0.913605 | 0.280077 | 0.768191 |
|  | rs3738443 | 0.899993 | 0.329107 | 0.773235 |
|  | rs7144649 | 0.912025 | 0.294772 | 0.775705 |
|  | rs1876831 | 0.91421 | 0.289027 | 0.776631 |
|  | rs886205 | 1.099157 | 0.368884 | 0.778166 |
|  | rs2548145 | 0.929768 | 0.245325 | 0.782559 |
|  | rs4770403 | 0.91378 | 0.31543 | 0.793935 |
|  | rs10893366 | 0.913626 | 0.332974 | 0.804241 |
|  | rs4758317 | 1.064536 | 0.284773 | 0.815154 |
|  | rs195204 | 1.067663 | 0.320704 | 0.827456 |
|  | rs2154294 | 0.945561 | 0.25122 | 0.833128 |
|  | rs768048 | 1.078967 | 0.420395 | 0.845339 |
|  | rs36061340 | 1.088688 | 0.573336 | 0.871815 |
|  | rs4543123 | 1.0491 | 0.323001 | 0.876283 |
|  | rs2380220 | 0.951605 | 0.360229 | 0.895744 |
|  | rs2228093 | 1.046726 | 0.413674 | 0.908007 |
|  | rs10849915 | 0.972668 | 0.275272 | 0.921994 |
|  | rs1864982 | 1.034758 | 0.392585 | 0.928241 |
|  | rs9871864 | 1.022226 | 0.269625 | 0.933578 |
|  | rs6943555 | 0.97942 | 0.301256 | 0.9461 |
|  | rs12388359 | 0.980901 | 0.304926 | 0.950538 |
|  | rs1497571 | 1.010927 | 0.265029 | 0.966933 |
|  | rs1230165 | 1.012487 | 0.340766 | 0.970588 |
|  | rs6902771 | 0.99057 | 0.26155 | 0.971376 |
|  | rs1353899 | 1.009491 | 0.327344 | 0.97676 |
| ADHD at age 7 | Offspring PGRS | 0.351254 | 0.286194 | 0.199112 |
|  | rs1109501 | 1.510729 | 0.274627 | 0.023227 |
|  | rs9512637 | 1.435243 | 0.248037 | 0.036544 |
|  | rs12311304 | 1.437199 | 0.253401 | 0.039679 |
|  | rs2154294 | 1.420228 | 0.246033 | 0.042857 |
|  | rs4543123 | 1.410395 | 0.264166 | 0.066367 |
|  | rs13160562 | 1.380473 | 0.24652 | 0.070991 |
|  | rs4440177 | 0.718903 | 0.139662 | 0.089356 |
|  | rs2369955 | 1.479217 | 0.342517 | 0.090873 |
|  | rs1497571 | 1.318361 | 0.227166 | 0.108708 |
|  | rs2303317 | 1.311624 | 0.225312 | 0.114304 |
|  | rs6902771 | 0.771603 | 0.134448 | 0.136737 |
|  | rs750338 | 0.72749 | 0.164264 | 0.158822 |
|  | rs2827312 | 1.290651 | 0.239916 | 0.169881 |
|  | rs279861 | 1.26075 | 0.216079 | 0.176398 |
|  | rs3764435 | 1.252088 | 0.214805 | 0.190053 |
|  | rs1573496 | 1.392959 | 0.356185 | 0.194924 |
|  | rs1876831 | 1.280685 | 0.244486 | 0.195003 |
|  | rs11724320 | 0.783312 | 0.148074 | 0.196377 |
|  | rs237238 | 0.583004 | 0.246173 | 0.201311 |
|  | rs1229984 | 1.707714 | 0.727894 | 0.209287 |
|  | rs1380131 | 0.651961 | 0.226152 | 0.217502 |
|  | rs420817 | 1.23601 | 0.213193 | 0.219279 |
|  | rs7144649 | 0.767126 | 0.170561 | 0.233125 |
|  | rs4761097 | 0.826146 | 0.144253 | 0.274054 |
|  | rs1042026 | 1.213924 | 0.22084 | 0.286599 |
|  | rs8062326 | 0.474584 | 0.338785 | 0.296453 |
|  | rs1793257 | 0.564123 | 0.330532 | 0.328537 |
|  | rs567926 | 1.181362 | 0.203204 | 0.33257 |
|  | rs4293630 | 0.766784 | 0.212175 | 0.337216 |
|  | rs12388359 | 0.79924 | 0.188111 | 0.341036 |
|  | rs6943555 | 1.189197 | 0.226973 | 0.363946 |
|  | rs1230165 | 1.199297 | 0.252723 | 0.388453 |
|  | rs59972978 | 0.813603 | 0.196845 | 0.393874 |
|  | rs195204 | 0.83888 | 0.175914 | 0.402143 |
|  | rs11851015 | 0.810197 | 0.21825 | 0.434599 |
|  | rs2380220 | 1.193257 | 0.272608 | 0.439292 |
|  | rs9871864 | 1.139925 | 0.195652 | 0.445449 |
|  | rs4770403 | 1.171404 | 0.24563 | 0.450568 |
|  | rs2100290 | 0.879374 | 0.150495 | 0.452584 |
|  | rs804292 | 0.862455 | 0.17377 | 0.462695 |
|  | rs1000579 | 1.12615 | 0.195783 | 0.494373 |
|  | rs768048 | 1.179071 | 0.286328 | 0.497562 |
|  | rs8040009 | 1.155975 | 0.251106 | 0.50461 |
|  | rs9636231 | 1.127208 | 0.209828 | 0.520048 |
|  | rs1864982 | 0.846174 | 0.225786 | 0.531331 |
|  | rs9656709 | 0.897659 | 0.155414 | 0.532891 |
|  | rs3819197 | 1.127462 | 0.220164 | 0.538976 |
|  | rs3131513 | 1.111221 | 0.191497 | 0.540564 |
|  | rs642899 | 0.885363 | 0.186452 | 0.563154 |
|  | rs59677118 | 0.828623 | 0.270298 | 0.564411 |
|  | rs7553212 | 1.104334 | 0.196449 | 0.57692 |
|  | rs933769 | 1.125201 | 0.240202 | 0.580552 |
|  | rs2228093 | 1.146586 | 0.284498 | 0.581438 |
|  | rs284786 | 1.102417 | 0.203273 | 0.596944 |
|  | rs4478858 | 0.912365 | 0.160188 | 0.601411 |
|  | rs9556711 | 1.189126 | 0.398001 | 0.604784 |
|  | rs36061340 | 1.182678 | 0.397918 | 0.618009 |
|  | rs1353899 | 0.902532 | 0.201424 | 0.64587 |
|  | rs3738443 | 0.901384 | 0.215575 | 0.664202 |
|  | rs9825310 | 0.929113 | 0.161089 | 0.671515 |
|  | rs36563 | 0.901396 | 0.223667 | 0.67568 |
|  | rs1824024 | 0.928877 | 0.172706 | 0.691508 |
|  | rs1789891 | 0.91033 | 0.220018 | 0.69749 |
|  | rs1800759 | 1.063597 | 0.184337 | 0.722027 |
|  | rs67031482 | 1.061601 | 0.180846 | 0.725657 |
|  | rs1353621 | 1.062871 | 0.188904 | 0.731547 |
|  | rs242938 | 0.883312 | 0.321907 | 0.733506 |
|  | rs62202398 | 0.883224 | 0.340021 | 0.747033 |
|  | rs6716455 | 0.927407 | 0.243207 | 0.773825 |
|  | rs1318937 | 0.928417 | 0.240255 | 0.774097 |
|  | rs10893366 | 0.936231 | 0.220912 | 0.780048 |
|  | rs1344694 | 0.953077 | 0.176913 | 0.795703 |
|  | rs3762894 | 1.059307 | 0.23809 | 0.797688 |
|  | rs12472151 | 0.90249 | 0.380632 | 0.807803 |
|  | rs10908907 | 0.959252 | 0.191092 | 0.834579 |
|  | rs2140418 | 1.044684 | 0.226961 | 0.840533 |
|  | rs13259667 | 0.942303 | 0.312567 | 0.857812 |
|  | rs10253361 | 0.973033 | 0.167888 | 0.874111 |
|  | rs2810114 | 1.023511 | 0.195809 | 0.903316 |
|  | rs2188561 | 0.976784 | 0.200352 | 0.908824 |
|  | rs3930234 | 1.026351 | 0.246962 | 0.913922 |
|  | rs7590720 | 0.984793 | 0.18725 | 0.935766 |
|  | rs6701037 | 0.98759 | 0.169301 | 0.941931 |
|  | rs16985179 | 1.021426 | 0.301407 | 0.942728 |
|  | rs886205 | 1.013596 | 0.225835 | 0.951669 |
|  | rs1908556 | 0.988993 | 0.243015 | 0.964074 |
|  | rs10849915 | 0.993627 | 0.182943 | 0.972301 |
|  | rs2548145 | 1.005176 | 0.172005 | 0.975934 |
|  | rs4758317 | 1.003179 | 0.174955 | 0.985481 |
| anti-social behaviour at age 11 | Offspring PGRS | 1.027403 | 0.291748 | 0.924153 |
|  | rs8062326 | 1.406739 | 0.219176 | 0.028495 |
|  | rs12311304 | 1.135013 | 0.071937 | 0.045698 |
|  | rs1497571 | 1.117751 | 0.066634 | 0.06186 |
|  | rs933769 | 1.129892 | 0.084545 | 0.102665 |
|  | rs3738443 | 0.875457 | 0.072059 | 0.106107 |
|  | rs4761097 | 1.098399 | 0.065755 | 0.116935 |
|  | rs59972978 | 0.881655 | 0.072275 | 0.124422 |
|  | rs2154294 | 0.91434 | 0.054923 | 0.136006 |
|  | rs1864982 | 1.133411 | 0.096345 | 0.140686 |
|  | rs3764435 | 1.091646 | 0.065377 | 0.14315 |
|  | rs1573496 | 1.149808 | 0.110969 | 0.14806 |
|  | rs195204 | 1.091643 | 0.074084 | 0.196342 |
|  | rs2303317 | 1.080324 | 0.064648 | 0.196674 |
|  | rs237238 | 0.853519 | 0.105619 | 0.200564 |
|  | rs16985179 | 1.134045 | 0.112467 | 0.204658 |
|  | rs1229984 | 0.769026 | 0.160076 | 0.207053 |
|  | rs4770403 | 0.906528 | 0.070848 | 0.209239 |
|  | rs1800759 | 1.078443 | 0.065313 | 0.212417 |
|  | rs6701037 | 1.073661 | 0.064123 | 0.234026 |
|  | rs1230165 | 1.092368 | 0.082212 | 0.240437 |
|  | rs2188561 | 1.085085 | 0.076087 | 0.244204 |
|  | rs2228093 | 1.104113 | 0.097848 | 0.263742 |
|  | rs1000579 | 1.068441 | 0.064793 | 0.274984 |
|  | rs242938 | 0.872773 | 0.110238 | 0.281313 |
|  | rs1876831 | 0.929154 | 0.066035 | 0.301169 |
|  | rs13259667 | 1.118748 | 0.122114 | 0.303944 |
|  | rs4440177 | 0.939102 | 0.060054 | 0.325839 |
|  | rs2380220 | 1.081017 | 0.089247 | 0.345372 |
|  | rs886205 | 1.075007 | 0.082487 | 0.345891 |
|  | rs12388359 | 1.067321 | 0.074861 | 0.352943 |
|  | rs1042026 | 0.94181 | 0.062553 | 0.366708 |
|  | rs10849915 | 1.05604 | 0.066936 | 0.389647 |
|  | rs2827312 | 0.944825 | 0.062793 | 0.393111 |
|  | rs420817 | 0.951489 | 0.056733 | 0.40428 |
|  | rs59677118 | 1.088185 | 0.111589 | 0.409864 |
|  | rs1789891 | 1.066301 | 0.08491 | 0.420144 |
|  | rs10908907 | 0.94564 | 0.065667 | 0.420885 |
|  | rs4758317 | 0.953508 | 0.058076 | 0.43443 |
|  | rs9871864 | 1.046784 | 0.062812 | 0.446073 |
|  | rs768048 | 0.933897 | 0.084696 | 0.450797 |
|  | rs750338 | 1.054846 | 0.074785 | 0.451366 |
|  | rs3819197 | 0.949181 | 0.067277 | 0.461829 |
|  | rs1380131 | 1.075221 | 0.108418 | 0.471972 |
|  | rs9825310 | 0.959131 | 0.057688 | 0.487829 |
|  | rs2548145 | 1.041237 | 0.062157 | 0.498454 |
|  | rs1908556 | 1.058212 | 0.089077 | 0.501476 |
|  | rs3930234 | 0.946551 | 0.080815 | 0.519984 |
|  | rs6943555 | 1.044567 | 0.071533 | 0.524314 |
|  | rs3762894 | 1.048828 | 0.082395 | 0.543956 |
|  | rs9656709 | 0.965093 | 0.058538 | 0.558026 |
|  | rs284786 | 0.963269 | 0.0632 | 0.568423 |
|  | rs567926 | 0.966256 | 0.058504 | 0.570756 |
|  | rs4478858 | 0.966721 | 0.058676 | 0.577101 |
|  | rs7553212 | 0.965624 | 0.061235 | 0.581213 |
|  | rs62202398 | 1.069259 | 0.132236 | 0.588176 |
|  | rs3131513 | 1.032863 | 0.062527 | 0.593255 |
|  | rs10893366 | 1.039781 | 0.082176 | 0.621586 |
|  | rs13160562 | 1.031831 | 0.067109 | 0.629952 |
|  | rs7144649 | 0.96749 | 0.069866 | 0.647187 |
|  | rs2140418 | 1.035196 | 0.078558 | 0.648518 |
|  | rs1353621 | 1.027676 | 0.063969 | 0.66097 |
|  | rs804292 | 1.02916 | 0.06885 | 0.667457 |
|  | rs8040009 | 0.967168 | 0.076022 | 0.671052 |
|  | rs1353899 | 0.969419 | 0.073444 | 0.681841 |
|  | rs1344694 | 0.974745 | 0.062846 | 0.691562 |
|  | rs642899 | 0.972326 | 0.069001 | 0.692501 |
|  | rs2100290 | 0.978481 | 0.058385 | 0.715431 |
|  | rs7590720 | 0.9779 | 0.065078 | 0.737012 |
|  | rs9556711 | 1.041837 | 0.128622 | 0.739901 |
|  | rs11724320 | 1.020674 | 0.064347 | 0.745499 |
|  | rs6716455 | 0.972132 | 0.088054 | 0.755014 |
|  | rs12472151 | 1.040017 | 0.145096 | 0.778525 |
|  | rs10253361 | 0.985247 | 0.059344 | 0.805101 |
|  | rs4543123 | 1.01653 | 0.071726 | 0.816267 |
|  | rs1793257 | 0.969253 | 0.155309 | 0.845473 |
|  | rs6902771 | 0.989075 | 0.058864 | 0.853554 |
|  | rs4293630 | 0.987914 | 0.086653 | 0.889744 |
|  | rs11851015 | 0.988037 | 0.086621 | 0.890815 |
|  | rs9512637 | 0.993152 | 0.062309 | 0.912786 |
|  | rs1824024 | 1.007019 | 0.064354 | 0.912842 |
|  | rs279861 | 0.993669 | 0.059924 | 0.916126 |
|  | rs36563 | 0.991381 | 0.082409 | 0.917059 |
|  | rs67031482 | 1.005896 | 0.060005 | 0.921503 |
|  | rs2369955 | 1.008085 | 0.091656 | 0.92943 |
|  | rs36061340 | 0.991003 | 0.124397 | 0.942605 |
|  | rs1318937 | 1.004156 | 0.089042 | 0.962693 |
|  | rs9636231 | 1.001661 | 0.066151 | 0.979953 |
|  | rs2810114 | 0.998725 | 0.066936 | 0.984815 |
|  | rs1109501 | 1.00079 | 0.068903 | 0.990845 |
| anti-social behaviour at age 13 | Offspring PGRS | 0.968721 | 0.182957 | 0.866377 |
|  | rs279861 | 0.859432 | 0.039513 | 0.000985 |
|  | rs567926 | 0.873133 | 0.040222 | 0.003229 |
|  | rs1497571 | 1.114245 | 0.04419 | 0.006379 |
|  | rs1864982 | 1.168111 | 0.067269 | 0.00697 |
|  | rs12388359 | 1.130749 | 0.053215 | 0.009027 |
|  | rs1042026 | 1.118734 | 0.055026 | 0.022543 |
|  | rs2810114 | 0.895432 | 0.046084 | 0.031866 |
|  | rs2380220 | 0.891193 | 0.05073 | 0.043005 |
|  | rs2228093 | 1.144888 | 0.0769 | 0.043962 |
|  | rs4543123 | 0.908736 | 0.043172 | 0.043964 |
|  | rs4770403 | 0.889437 | 0.052451 | 0.04694 |
|  | rs2380220 | 1.124864 | 0.070226 | 0.059472 |
|  | rs36563 | 0.901413 | 0.050354 | 0.063165 |
|  | rs67031482 | 1.075964 | 0.042685 | 0.064951 |
|  | rs4478858 | 0.929051 | 0.037495 | 0.068238 |
|  | rs9656709 | 0.930572 | 0.037534 | 0.074426 |
|  | rs4293630 | 1.122133 | 0.072648 | 0.075095 |
|  | rs3131513 | 0.930983 | 0.037675 | 0.077198 |
|  | rs1800759 | 1.073932 | 0.043407 | 0.077613 |
|  | rs933769 | 1.093254 | 0.055548 | 0.0793 |
|  | rs36563 | 0.895837 | 0.05754 | 0.0868 |
|  | rs804292 | 1.078936 | 0.04798 | 0.087546 |
|  | rs1109501 | 1.090679 | 0.0562 | 0.092076 |
|  | rs13259667 | 0.879899 | 0.067428 | 0.094987 |
|  | rs4761097 | 1.076593 | 0.04884 | 0.103773 |
|  | rs1344694 | 0.936616 | 0.040166 | 0.126774 |
|  | rs4761097 | 0.940991 | 0.037609 | 0.128068 |
|  | rs62202398 | 0.879219 | 0.075338 | 0.133038 |
|  | rs8040009 | 1.087507 | 0.063423 | 0.150314 |
|  | rs9556711 | 0.887997 | 0.075214 | 0.160788 |
|  | rs9871864 | 0.947266 | 0.037869 | 0.175366 |
|  | rs3819197 | 1.071902 | 0.056326 | 0.186383 |
|  | rs1229984 | 0.820495 | 0.12365 | 0.189238 |
|  | rs1318937 | 1.088376 | 0.071894 | 0.199827 |
|  | rs933769 | 1.076028 | 0.061982 | 0.203332 |
|  | rs7553212 | 0.9492 | 0.039948 | 0.21542 |
|  | rs4440177 | 1.060062 | 0.050525 | 0.221041 |
|  | rs12472151 | 1.134518 | 0.117875 | 0.22447 |
|  | rs8062326 | 1.146841 | 0.131219 | 0.231125 |
|  | rs2228093 | 1.074033 | 0.064333 | 0.23312 |
|  | rs7553212 | 0.944649 | 0.045383 | 0.235919 |
|  | rs59972978 | 1.072977 | 0.063853 | 0.236561 |
|  | rs7590720 | 0.949566 | 0.04201 | 0.242111 |
|  | rs1230165 | 1.059166 | 0.053778 | 0.257591 |
|  | rs4770403 | 0.944632 | 0.048146 | 0.263755 |
|  | rs6701037 | 0.950906 | 0.043113 | 0.266861 |
|  | rs8062326 | 1.152204 | 0.147622 | 0.268815 |
|  | rs12311304 | 1.054899 | 0.051164 | 0.270492 |
|  | rs1229984 | 1.142751 | 0.140604 | 0.278138 |
|  | rs11724320 | 1.052054 | 0.050111 | 0.286716 |
|  | rs9825310 | 0.958942 | 0.038316 | 0.29407 |
|  | rs6902771 | 1.042202 | 0.041201 | 0.295736 |
|  | rs237238 | 1.095412 | 0.095478 | 0.295776 |
|  | rs67031482 | 0.954204 | 0.043102 | 0.299364 |
|  | rs1864982 | 1.070447 | 0.070344 | 0.300229 |
|  | rs59972978 | 1.055129 | 0.055496 | 0.307598 |
|  | rs36061340 | 1.086969 | 0.089477 | 0.311033 |
|  | rs1793257 | 1.110743 | 0.115246 | 0.311406 |
|  | rs9512637 | 0.959319 | 0.040058 | 0.319927 |
|  | rs2100290 | 1.04537 | 0.047211 | 0.325866 |
|  | rs10849915 | 1.042331 | 0.044106 | 0.327196 |
|  | rs2810114 | 0.957809 | 0.0428 | 0.334706 |
|  | rs768048 | 1.065363 | 0.070919 | 0.341529 |
|  | rs1353621 | 0.956319 | 0.045313 | 0.345873 |
|  | rs3930234 | 0.941622 | 0.060664 | 0.350473 |
|  | rs10253361 | 1.043143 | 0.047472 | 0.353327 |
|  | rs804292 | 0.953736 | 0.048896 | 0.355518 |
|  | rs2188561 | 1.044623 | 0.049375 | 0.355679 |
|  | rs768048 | 0.946591 | 0.056365 | 0.35664 |
|  | rs2369955 | 0.946468 | 0.05771 | 0.366886 |
|  | rs1876831 | 1.048205 | 0.05513 | 0.370711 |
|  | rs13259667 | 0.926725 | 0.080782 | 0.382664 |
|  | rs9512637 | 1.042159 | 0.049304 | 0.382742 |
|  | rs4440177 | 1.037284 | 0.043579 | 0.383587 |
|  | rs1573496 | 1.058526 | 0.070281 | 0.391633 |
|  | rs2827312 | 1.03766 | 0.045533 | 0.399517 |
|  | rs886205 | 1.04394 | 0.053962 | 0.405454 |
|  | rs642899 | 0.962245 | 0.045277 | 0.4134 |
|  | rs2100290 | 0.970212 | 0.038482 | 0.445799 |
|  | rs3738443 | 0.961831 | 0.051008 | 0.463059 |
|  | rs1109501 | 0.966919 | 0.044385 | 0.463645 |
|  | rs11724320 | 0.970257 | 0.040826 | 0.473013 |
|  | rs6716455 | 1.04232 | 0.061957 | 0.485609 |
|  | rs9871864 | 1.031758 | 0.046883 | 0.491436 |
|  | rs10253361 | 1.02782 | 0.041108 | 0.492654 |
|  | rs3930234 | 0.962289 | 0.054062 | 0.493834 |
|  | rs2548145 | 1.030143 | 0.046546 | 0.511011 |
|  | rs3131513 | 0.97037 | 0.044656 | 0.513374 |
|  | rs1497571 | 0.970955 | 0.043827 | 0.513752 |
|  | rs6902771 | 1.029793 | 0.046341 | 0.514152 |
|  | rs12472151 | 1.061125 | 0.098915 | 0.524469 |
|  | rs36061340 | 0.941542 | 0.090465 | 0.530708 |
|  | rs237238 | 1.049669 | 0.081469 | 0.532255 |
|  | rs1793257 | 0.927034 | 0.113421 | 0.535743 |
|  | rs9556711 | 0.945165 | 0.090757 | 0.55699 |
|  | rs567926 | 1.023769 | 0.041107 | 0.558519 |
|  | rs13160562 | 0.971542 | 0.048177 | 0.560431 |
|  | rs11851015 | 1.033969 | 0.059833 | 0.563764 |
|  | rs284786 | 1.025232 | 0.044401 | 0.565034 |
|  | rs10893366 | 0.966508 | 0.058756 | 0.575225 |
|  | rs1800759 | 1.02589 | 0.047233 | 0.578789 |
|  | rs2369955 | 1.03827 | 0.071081 | 0.583302 |
|  | rs2154294 | 1.02121 | 0.040677 | 0.598249 |
|  | rs13160562 | 0.977446 | 0.042501 | 0.599837 |
|  | rs1380131 | 1.041298 | 0.080521 | 0.600743 |
|  | rs16985179 | 0.964889 | 0.066454 | 0.603783 |
|  | rs284786 | 1.025507 | 0.050512 | 0.6091 |
|  | rs2140418 | 0.971534 | 0.056539 | 0.619732 |
|  | rs420817 | 1.019654 | 0.040384 | 0.62312 |
|  | rs62202398 | 1.046964 | 0.099094 | 0.627754 |
|  | rs1789891 | 0.971171 | 0.059806 | 0.634764 |
|  | rs10908907 | 1.02147 | 0.046546 | 0.641081 |
|  | rs1000579 | 1.021397 | 0.047058 | 0.645865 |
|  | rs1344694 | 0.978727 | 0.047695 | 0.659031 |
|  | rs6943555 | 1.023209 | 0.053306 | 0.659644 |
|  | rs886205 | 0.974246 | 0.05786 | 0.660429 |
|  | rs1353899 | 1.024436 | 0.058053 | 0.670086 |
|  | rs1042026 | 0.981802 | 0.042961 | 0.674697 |
|  | rs242938 | 0.962157 | 0.088568 | 0.675152 |
|  | rs6701037 | 0.983617 | 0.039121 | 0.677906 |
|  | rs4478858 | 0.981271 | 0.045019 | 0.680264 |
|  | rs9636231 | 0.982552 | 0.043197 | 0.688883 |
|  | rs11851015 | 0.973796 | 0.064737 | 0.689579 |
|  | rs7590720 | 0.980487 | 0.049316 | 0.695213 |
|  | rs1380131 | 1.026653 | 0.07011 | 0.7001 |
|  | rs2188561 | 0.979504 | 0.053103 | 0.702475 |
|  | rs242938 | 0.970685 | 0.078073 | 0.711437 |
|  | rs2154294 | 1.016752 | 0.046108 | 0.714109 |
|  | rs2303317 | 1.016574 | 0.046046 | 0.716667 |
|  | rs279861 | 1.013001 | 0.040585 | 0.747147 |
|  | rs3764435 | 1.011573 | 0.040245 | 0.772404 |
|  | rs59677118 | 1.020178 | 0.071219 | 0.774749 |
|  | rs1230165 | 0.983521 | 0.057323 | 0.775567 |
|  | rs1908556 | 0.984472 | 0.056091 | 0.783564 |
|  | rs2303317 | 1.010361 | 0.040198 | 0.795572 |
|  | rs3762894 | 0.98664 | 0.052247 | 0.799497 |
|  | rs1573496 | 1.01805 | 0.077193 | 0.813493 |
|  | rs195204 | 1.012079 | 0.052782 | 0.81791 |
|  | rs1318937 | 1.012735 | 0.059672 | 0.829941 |
|  | rs3738443 | 0.987196 | 0.059492 | 0.830669 |
|  | rs3819197 | 1.009935 | 0.047022 | 0.831851 |
|  | rs1876831 | 0.990344 | 0.046093 | 0.834854 |
|  | rs3762894 | 0.987713 | 0.059602 | 0.837672 |
|  | rs2140418 | 0.990133 | 0.050405 | 0.845552 |
|  | rs16985179 | 1.01516 | 0.078944 | 0.846586 |
|  | rs4758317 | 0.991561 | 0.045623 | 0.853872 |
|  | rs59677118 | 1.014336 | 0.080574 | 0.857788 |
|  | rs12388359 | 0.990407 | 0.054342 | 0.860543 |
|  | rs10893366 | 0.990947 | 0.052625 | 0.864027 |
|  | rs1824024 | 0.992861 | 0.042231 | 0.866229 |
|  | rs8040009 | 1.008467 | 0.052261 | 0.870759 |
|  | rs1353899 | 0.992627 | 0.049645 | 0.88237 |
|  | rs195204 | 0.993486 | 0.045611 | 0.886801 |
|  | rs2827312 | 1.006941 | 0.050348 | 0.889978 |
|  | rs6716455 | 0.99115 | 0.067519 | 0.896176 |
|  | rs1353621 | 0.994742 | 0.04127 | 0.898881 |
|  | rs12311304 | 1.004791 | 0.04301 | 0.911096 |
|  | rs7144649 | 0.994227 | 0.053998 | 0.915099 |
|  | rs420817 | 0.995807 | 0.044907 | 0.925769 |
|  | rs1000579 | 0.996245 | 0.040369 | 0.926028 |
|  | rs10908907 | 1.00426 | 0.05216 | 0.934769 |
|  | rs4543123 | 1.004079 | 0.053767 | 0.939405 |
|  | rs642899 | 0.996113 | 0.053205 | 0.941879 |
|  | rs2548145 | 1.002768 | 0.039801 | 0.944484 |
|  | rs1824024 | 0.99685 | 0.048271 | 0.948055 |
|  | rs4293630 | 0.996521 | 0.057926 | 0.952191 |
|  | rs750338 | 1.002784 | 0.047777 | 0.953472 |
|  | rs10849915 | 0.997322 | 0.048151 | 0.9557 |
|  | rs1908556 | 1.003481 | 0.064924 | 0.957164 |
|  | rs4758317 | 1.002105 | 0.040486 | 0.958498 |
|  | rs9636231 | 0.99767 | 0.049891 | 0.962788 |
|  | rs6943555 | 1.002049 | 0.045988 | 0.964426 |
|  | rs1789891 | 0.998033 | 0.053697 | 0.970812 |
|  | rs3764435 | 1.001551 | 0.045367 | 0.972701 |
|  | rs7144649 | 0.998837 | 0.047608 | 0.98052 |
|  | rs9656709 | 0.999054 | 0.045836 | 0.983548 |
|  | rs9825310 | 0.999306 | 0.045435 | 0.98781 |
|  | rs750338 | 0.999417 | 0.054233 | 0.991419 |
| anti-social behaviour at age 15 | Offspring PGRS | 1.377311 | 0.263521 | 0.094288 |
|  | rs2827312 | 0.903242 | 0.04036 | 0.022758 |
|  | rs195204 | 1.097573 | 0.050562 | 0.043278 |
|  | rs1353899 | 1.100727 | 0.055126 | 0.055325 |
|  | rs8040009 | 1.100093 | 0.057226 | 0.066677 |
|  | rs13160562 | 0.924309 | 0.040839 | 0.074845 |
|  | rs4478858 | 0.931671 | 0.038039 | 0.083014 |
|  | rs2154294 | 1.070524 | 0.043145 | 0.090851 |
|  | rs9512637 | 1.072656 | 0.045095 | 0.09525 |
|  | rs8062326 | 0.821136 | 0.100574 | 0.107627 |
|  | rs11724320 | 1.069161 | 0.04529 | 0.114409 |
|  | rs279861 | 0.940912 | 0.038208 | 0.133655 |
|  | rs567926 | 0.943689 | 0.038416 | 0.154515 |
|  | rs1497571 | 0.946355 | 0.037966 | 0.169327 |
|  | rs1573496 | 1.09597 | 0.073248 | 0.170327 |
|  | rs59972978 | 0.930996 | 0.050183 | 0.184684 |
|  | rs1109501 | 1.062747 | 0.048963 | 0.186526 |
|  | rs10893366 | 0.936844 | 0.050691 | 0.227934 |
|  | rs1353621 | 1.051008 | 0.044019 | 0.234899 |
|  | rs3738443 | 0.938971 | 0.05055 | 0.242126 |
|  | rs750338 | 0.94603 | 0.045874 | 0.252561 |
|  | rs6902771 | 1.044087 | 0.041753 | 0.280649 |
|  | rs2228093 | 0.93593 | 0.057547 | 0.281525 |
|  | rs13259667 | 0.925302 | 0.071231 | 0.313218 |
|  | rs12311304 | 0.959907 | 0.041697 | 0.346201 |
|  | rs1864982 | 1.052246 | 0.061881 | 0.386506 |
|  | rs2100290 | 1.035018 | 0.041529 | 0.390986 |
|  | rs9871864 | 1.033506 | 0.041736 | 0.414436 |
|  | rs2810114 | 0.965343 | 0.04362 | 0.435054 |
|  | rs9656709 | 1.032251 | 0.042076 | 0.436145 |
|  | rs6716455 | 0.955123 | 0.058062 | 0.450069 |
|  | rs2303317 | 1.030358 | 0.041469 | 0.457436 |
|  | rs420817 | 0.97139 | 0.038928 | 0.468866 |
|  | rs284786 | 1.032142 | 0.045196 | 0.469993 |
|  | rs9556711 | 1.062087 | 0.088591 | 0.470204 |
|  | rs1230165 | 0.963998 | 0.049984 | 0.479476 |
|  | rs768048 | 0.958343 | 0.057666 | 0.479489 |
|  | rs9825310 | 0.972093 | 0.039284 | 0.48369 |
|  | rs886205 | 1.03717 | 0.054249 | 0.485333 |
|  | rs1318937 | 1.041959 | 0.061874 | 0.48883 |
|  | rs10849915 | 1.029102 | 0.04407 | 0.502934 |
|  | rs1042026 | 1.029595 | 0.045424 | 0.50856 |
|  | rs12472151 | 1.063568 | 0.100183 | 0.512935 |
|  | rs9636231 | 1.027717 | 0.045561 | 0.537427 |
|  | rs3764435 | 1.023461 | 0.041192 | 0.564485 |
|  | rs804292 | 0.9764 | 0.044266 | 0.598341 |
|  | rs1000579 | 0.978942 | 0.040159 | 0.603899 |
|  | rs1229984 | 0.939332 | 0.12039 | 0.62532 |
|  | rs3131513 | 1.019341 | 0.041572 | 0.63856 |
|  | rs4440177 | 0.980625 | 0.041819 | 0.646383 |
|  | rs10253361 | 1.018358 | 0.041206 | 0.653009 |
|  | rs6943555 | 0.979329 | 0.045563 | 0.65346 |
|  | rs642899 | 0.979529 | 0.046554 | 0.663428 |
|  | rs1800759 | 1.017708 | 0.041669 | 0.668131 |
|  | rs7144649 | 1.020585 | 0.049111 | 0.671981 |
|  | rs1908556 | 1.022816 | 0.058666 | 0.694081 |
|  | rs36061340 | 1.033379 | 0.086556 | 0.695062 |
|  | rs1380131 | 0.974158 | 0.067777 | 0.706683 |
|  | rs1824024 | 0.984365 | 0.042381 | 0.714344 |
|  | rs7590720 | 0.983793 | 0.043918 | 0.714344 |
|  | rs62202398 | 1.031438 | 0.087272 | 0.714486 |
|  | rs3930234 | 0.979601 | 0.055572 | 0.716373 |
|  | rs2548145 | 0.988787 | 0.039706 | 0.77885 |
|  | rs6701037 | 0.989819 | 0.039821 | 0.799224 |
|  | rs16985179 | 1.017569 | 0.070373 | 0.801162 |
|  | rs2380220 | 0.985886 | 0.056021 | 0.80246 |
|  | rs1876831 | 0.988543 | 0.046556 | 0.806706 |
|  | rs10908907 | 0.989702 | 0.045741 | 0.822775 |
|  | rs4543123 | 1.010469 | 0.048058 | 0.826663 |
|  | rs1793257 | 1.021319 | 0.108412 | 0.842475 |
|  | rs1344694 | 0.991793 | 0.042879 | 0.848831 |
|  | rs12388359 | 0.990935 | 0.048246 | 0.85164 |
|  | rs59677118 | 1.012482 | 0.071563 | 0.860683 |
|  | rs36563 | 1.009316 | 0.056214 | 0.86777 |
|  | rs4758317 | 0.993691 | 0.040618 | 0.87696 |
|  | rs1789891 | 0.992023 | 0.054033 | 0.883094 |
|  | rs3762894 | 0.993221 | 0.05317 | 0.898895 |
|  | rs4770403 | 0.994116 | 0.050968 | 0.90836 |
|  | rs4293630 | 0.993642 | 0.058454 | 0.913663 |
|  | rs2188561 | 1.004651 | 0.048204 | 0.922962 |
|  | rs3819197 | 1.004517 | 0.047333 | 0.923795 |
|  | rs4761097 | 0.996185 | 0.040219 | 0.924566 |
|  | rs237238 | 1.00718 | 0.079488 | 0.927774 |
|  | rs7553212 | 1.003703 | 0.042585 | 0.930572 |
|  | rs242938 | 0.994139 | 0.080615 | 0.942209 |
|  | rs67031482 | 1.00259 | 0.040217 | 0.948588 |
|  | rs11851015 | 0.996282 | 0.058565 | 0.949473 |
|  | rs933769 | 1.002431 | 0.051955 | 0.962632 |
|  | rs2369955 | 1.002221 | 0.061391 | 0.971114 |
|  | rs2140418 | 0.998248 | 0.051367 | 0.972814 |
| anti-social behaviour at age 18 | Offspring PGRS | 2.021649 | 0.547207 | 0.009306 |
|  | rs4761097 | 1.153665 | 0.065274 | 0.011523 |
|  | rs1318937 | 1.214321 | 0.096846 | 0.014899 |
|  | rs8040009 | 1.170066 | 0.083708 | 0.028137 |
|  | rs6716455 | 0.834678 | 0.074677 | 0.043401 |
|  | rs4770403 | 1.150732 | 0.080326 | 0.044292 |
|  | rs2380220 | 1.164365 | 0.089303 | 0.047241 |
|  | rs3930234 | 1.15803 | 0.089036 | 0.056353 |
|  | rs4440177 | 1.110649 | 0.065628 | 0.075732 |
|  | rs1000579 | 0.909591 | 0.052889 | 0.103169 |
|  | rs279861 | 0.912969 | 0.052297 | 0.111936 |
|  | rs36061340 | 0.822186 | 0.103663 | 0.120456 |
|  | rs567926 | 0.919749 | 0.052814 | 0.14516 |
|  | rs16985179 | 1.144146 | 0.107191 | 0.150625 |
|  | rs9556711 | 0.836328 | 0.105018 | 0.154623 |
|  | rs10253361 | 0.925414 | 0.052869 | 0.174844 |
|  | rs1908556 | 1.111505 | 0.087379 | 0.178704 |
|  | rs2154294 | 0.92707 | 0.052624 | 0.18219 |
|  | rs1876831 | 0.914145 | 0.061606 | 0.182858 |
|  | rs7590720 | 0.921401 | 0.058536 | 0.197555 |
|  | rs420817 | 1.074104 | 0.060523 | 0.20456 |
|  | rs67031482 | 0.937219 | 0.052914 | 0.250789 |
|  | rs642899 | 1.07825 | 0.070846 | 0.251533 |
|  | rs1230165 | 1.083093 | 0.077275 | 0.263233 |
|  | rs195204 | 1.074424 | 0.069186 | 0.264941 |
|  | rs1380131 | 0.894544 | 0.090205 | 0.269098 |
|  | rs9512637 | 1.066198 | 0.062825 | 0.276674 |
|  | rs1789891 | 0.921542 | 0.071993 | 0.295613 |
|  | rs768048 | 1.088405 | 0.089664 | 0.303799 |
|  | rs2303317 | 1.054444 | 0.059657 | 0.348748 |
|  | rs2188561 | 0.937962 | 0.064235 | 0.349682 |
|  | rs11724320 | 1.055594 | 0.062697 | 0.362348 |
|  | rs9871864 | 1.051942 | 0.059679 | 0.372082 |
|  | rs62202398 | 0.900486 | 0.111363 | 0.396669 |
|  | rs3764435 | 1.047527 | 0.059285 | 0.411974 |
|  | rs1800759 | 1.047503 | 0.060122 | 0.418749 |
|  | rs10849915 | 0.953143 | 0.057815 | 0.428838 |
|  | rs933769 | 1.058291 | 0.076098 | 0.430751 |
|  | rs1109501 | 1.050025 | 0.067755 | 0.449355 |
|  | rs1353899 | 1.053385 | 0.074005 | 0.459122 |
|  | rs11851015 | 1.058575 | 0.086185 | 0.484443 |
|  | rs2100290 | 1.039884 | 0.058668 | 0.488187 |
|  | rs10908907 | 0.955653 | 0.062588 | 0.488556 |
|  | rs1793257 | 0.897836 | 0.139756 | 0.488726 |
|  | rs2228093 | 1.058594 | 0.089625 | 0.501229 |
|  | rs13259667 | 0.93036 | 0.101792 | 0.509417 |
|  | rs12311304 | 0.963889 | 0.059011 | 0.548008 |
|  | rs2140418 | 1.04252 | 0.074709 | 0.561187 |
|  | rs1344694 | 0.965539 | 0.058923 | 0.565519 |
|  | rs9656709 | 1.033428 | 0.05922 | 0.566104 |
|  | rs7144649 | 1.039148 | 0.069908 | 0.568127 |
|  | rs1864982 | 1.047266 | 0.086062 | 0.574119 |
|  | rs6701037 | 0.971399 | 0.054997 | 0.608274 |
|  | rs12388359 | 0.965522 | 0.067036 | 0.61331 |
|  | rs3762894 | 1.037286 | 0.077297 | 0.623245 |
|  | rs2810114 | 0.973217 | 0.061936 | 0.669678 |
|  | rs9636231 | 0.973807 | 0.061082 | 0.67218 |
|  | rs3738443 | 0.97106 | 0.073469 | 0.697905 |
|  | rs4758317 | 0.979281 | 0.056324 | 0.71585 |
|  | rs1824024 | 0.980809 | 0.059469 | 0.749278 |
|  | rs750338 | 1.021135 | 0.068925 | 0.756672 |
|  | rs12472151 | 0.959 | 0.129976 | 0.757408 |
|  | rs886205 | 1.022481 | 0.075103 | 0.762138 |
|  | rs2827312 | 1.018566 | 0.063556 | 0.768138 |
|  | rs242938 | 0.968273 | 0.111472 | 0.779438 |
|  | rs1042026 | 0.982883 | 0.061266 | 0.781793 |
|  | rs8062326 | 1.037868 | 0.169906 | 0.820388 |
|  | rs59972978 | 1.016631 | 0.076154 | 0.825721 |
|  | rs284786 | 0.986619 | 0.060981 | 0.827461 |
|  | rs3819197 | 1.013578 | 0.067007 | 0.83835 |
|  | rs7553212 | 1.011951 | 0.060295 | 0.841963 |
|  | rs59677118 | 0.981119 | 0.098261 | 0.849052 |
|  | rs2548145 | 1.01009 | 0.057022 | 0.858853 |
|  | rs13160562 | 0.990964 | 0.061282 | 0.883298 |
|  | rs4293630 | 1.011516 | 0.083347 | 0.889484 |
|  | rs4543123 | 0.990763 | 0.066447 | 0.889944 |
|  | rs4478858 | 1.007741 | 0.057692 | 0.89285 |
|  | rs9825310 | 0.992385 | 0.056379 | 0.892962 |
|  | rs1353621 | 1.007551 | 0.059407 | 0.898471 |
|  | rs804292 | 0.99278 | 0.063234 | 0.909419 |
|  | rs36563 | 1.008802 | 0.07891 | 0.910793 |
|  | rs10893366 | 0.992889 | 0.075061 | 0.924793 |
|  | rs2369955 | 1.007257 | 0.086615 | 0.932989 |
|  | rs6902771 | 0.995335 | 0.055996 | 0.933754 |
|  | rs1497571 | 0.995452 | 0.056116 | 0.935547 |
|  | rs237238 | 1.008158 | 0.111716 | 0.941548 |
|  | rs1229984 | 0.992004 | 0.177251 | 0.964162 |
|  | rs1573496 | 1.001396 | 0.095194 | 0.988292 |
|  | rs6943555 | 0.999328 | 0.065257 | 0.991787 |
|  | rs3131513 | 0.999646 | 0.057366 | 0.995082 |
| anti-social behaviour at age 19 | Offspring PGRS | 2.999597 | 0.923059 | 0.000357 |
|  | rs1908556 | 1.2922 | 0.110181 | 0.002643 |
|  | rs279861 | 0.843402 | 0.05507 | 0.009098 |
|  | rs567926 | 0.846014 | 0.055418 | 0.010687 |
|  | rs12472151 | 1.350948 | 0.184856 | 0.027926 |
|  | rs8040009 | 1.188949 | 0.095747 | 0.031626 |
|  | rs59677118 | 1.253082 | 0.131547 | 0.031629 |
|  | rs2188561 | 0.842791 | 0.06729 | 0.032177 |
|  | rs13160562 | 0.860358 | 0.061688 | 0.035929 |
|  | rs1230165 | 1.178831 | 0.093322 | 0.037687 |
|  | rs10253361 | 0.879602 | 0.05712 | 0.048211 |
|  | rs1864982 | 0.824555 | 0.082601 | 0.05414 |
|  | rs1800759 | 1.131036 | 0.073084 | 0.056702 |
|  | rs1000579 | 0.888106 | 0.05868 | 0.072501 |
|  | rs2827312 | 1.108019 | 0.07775 | 0.143797 |
|  | rs1497571 | 0.917828 | 0.058721 | 0.18017 |
|  | rs12388359 | 0.89773 | 0.073354 | 0.186718 |
|  | rs933769 | 1.109269 | 0.089158 | 0.196976 |
|  | rs2548145 | 1.082612 | 0.069202 | 0.214315 |
|  | rs2369955 | 1.124348 | 0.106188 | 0.214612 |
|  | rs3738443 | 1.107862 | 0.091795 | 0.216371 |
|  | rs1876831 | 1.09456 | 0.080489 | 0.219187 |
|  | rs4758317 | 0.923038 | 0.060307 | 0.220293 |
|  | rs1318937 | 1.11809 | 0.102831 | 0.224873 |
|  | rs10849915 | 1.085279 | 0.073414 | 0.226356 |
|  | rs62202398 | 0.840173 | 0.120978 | 0.2265 |
|  | rs10908907 | 1.087296 | 0.078663 | 0.247339 |
|  | rs242938 | 1.151449 | 0.141543 | 0.251296 |
|  | rs4293630 | 1.107176 | 0.100697 | 0.26295 |
|  | rs9512637 | 0.926985 | 0.062784 | 0.262957 |
|  | rs2810114 | 0.923867 | 0.06726 | 0.276733 |
|  | rs284786 | 1.075732 | 0.074383 | 0.291083 |
|  | rs16985179 | 1.1112 | 0.118551 | 0.323002 |
|  | rs2380220 | 1.085894 | 0.095781 | 0.350188 |
|  | rs2154294 | 0.942615 | 0.060568 | 0.357713 |
|  | rs1789891 | 0.923212 | 0.081771 | 0.367031 |
|  | rs6943555 | 0.935223 | 0.07014 | 0.371879 |
|  | rs9656709 | 1.058815 | 0.068708 | 0.378474 |
|  | rs7553212 | 0.942647 | 0.064249 | 0.38618 |
|  | rs11851015 | 1.082473 | 0.099072 | 0.38656 |
|  | rs6701037 | 0.950367 | 0.060996 | 0.42767 |
|  | rs1380131 | 0.914419 | 0.103935 | 0.431206 |
|  | rs8062326 | 0.857955 | 0.171156 | 0.442508 |
|  | rs3762894 | 1.06424 | 0.089125 | 0.457203 |
|  | rs2303317 | 0.95377 | 0.061187 | 0.460631 |
|  | rs804292 | 0.9495 | 0.069145 | 0.476722 |
|  | rs1109501 | 1.050844 | 0.076718 | 0.496944 |
|  | rs7590720 | 0.956145 | 0.068406 | 0.530768 |
|  | rs4440177 | 1.043043 | 0.070299 | 0.531785 |
|  | rs2228093 | 1.061603 | 0.101598 | 0.532207 |
|  | rs1573496 | 0.935827 | 0.102977 | 0.54668 |
|  | rs36061340 | 1.081807 | 0.141239 | 0.546987 |
|  | rs2100290 | 0.965805 | 0.061713 | 0.586085 |
|  | rs3131513 | 0.967031 | 0.063035 | 0.607032 |
|  | rs4770403 | 1.041738 | 0.084107 | 0.612527 |
|  | rs4543123 | 0.965976 | 0.073792 | 0.650441 |
|  | rs6902771 | 1.028971 | 0.065494 | 0.653651 |
|  | rs9556711 | 0.941636 | 0.128868 | 0.66036 |
|  | rs1344694 | 0.970974 | 0.067075 | 0.669824 |
|  | rs67031482 | 0.973344 | 0.062191 | 0.672407 |
|  | rs7144649 | 1.030699 | 0.07861 | 0.691764 |
|  | rs237238 | 1.047207 | 0.129767 | 0.709715 |
|  | rs4761097 | 0.977002 | 0.062874 | 0.7177 |
|  | rs59972978 | 0.969672 | 0.083179 | 0.719578 |
|  | rs195204 | 1.026605 | 0.075463 | 0.720941 |
|  | rs6716455 | 0.969019 | 0.094101 | 0.745882 |
|  | rs1824024 | 1.020523 | 0.0697 | 0.766117 |
|  | rs420817 | 1.018185 | 0.064955 | 0.777569 |
|  | rs4478858 | 0.98248 | 0.063789 | 0.785447 |
|  | rs9636231 | 0.981431 | 0.069644 | 0.791673 |
|  | rs1042026 | 1.018659 | 0.071489 | 0.792223 |
|  | rs886205 | 1.021878 | 0.084976 | 0.794669 |
|  | rs2140418 | 0.980087 | 0.080698 | 0.80701 |
|  | rs1793257 | 1.040021 | 0.173806 | 0.814357 |
|  | rs10893366 | 1.019333 | 0.086672 | 0.821822 |
|  | rs1353899 | 1.017664 | 0.081569 | 0.827076 |
|  | rs1229984 | 0.95672 | 0.196242 | 0.829221 |
|  | rs12311304 | 0.989759 | 0.068371 | 0.881547 |
|  | rs750338 | 1.010539 | 0.077395 | 0.891116 |
|  | rs3930234 | 0.988119 | 0.089327 | 0.894814 |
|  | rs9825310 | 0.992136 | 0.063832 | 0.902339 |
|  | rs13259667 | 1.012002 | 0.122061 | 0.921208 |
|  | rs11724320 | 0.993413 | 0.06728 | 0.922261 |
|  | rs642899 | 1.005566 | 0.07585 | 0.941338 |
|  | rs1353621 | 0.996125 | 0.066584 | 0.953682 |
|  | rs3819197 | 0.996375 | 0.07485 | 0.961441 |
|  | rs768048 | 0.996658 | 0.09507 | 0.972006 |
|  | rs3764435 | 1.002014 | 0.064216 | 0.974951 |
|  | rs9871864 | 0.999331 | 0.064286 | 0.991694 |
|  | rs36563 | 0.999738 | 0.088774 | 0.997647 |
| anti-social behaviour at age 21 | Offspring PGRS | 1.348567 | 0.369015 | 0.274458 |
|  | rs1000579 | 0.809952 | 0.048554 | 0.000438 |
|  | rs6716455 | 1.195623 | 0.09858 | 0.030239 |
|  | rs242938 | 1.223486 | 0.133015 | 0.063554 |
|  | rs9556711 | 1.229913 | 0.138767 | 0.066629 |
|  | rs2827312 | 1.121452 | 0.070634 | 0.068776 |
|  | rs36563 | 1.141675 | 0.087944 | 0.085424 |
|  | rs62202398 | 0.798965 | 0.104707 | 0.086792 |
|  | rs1109501 | 1.113998 | 0.072324 | 0.096349 |
|  | rs2188561 | 0.890317 | 0.06278 | 0.099438 |
|  | rs6943555 | 0.894754 | 0.06075 | 0.10144 |
|  | rs1344694 | 0.903168 | 0.056547 | 0.103803 |
|  | rs7590720 | 0.902308 | 0.058499 | 0.112827 |
|  | rs9636231 | 1.103469 | 0.069049 | 0.115612 |
|  | rs237238 | 0.833572 | 0.099798 | 0.128395 |
|  | rs1864982 | 1.131752 | 0.092603 | 0.130374 |
|  | rs1824024 | 0.91059 | 0.056733 | 0.132759 |
|  | rs2369955 | 1.132863 | 0.096087 | 0.141354 |
|  | rs10849915 | 1.089176 | 0.066163 | 0.159663 |
|  | rs1318937 | 0.886518 | 0.078394 | 0.173154 |
|  | rs2380220 | 1.112922 | 0.087713 | 0.174624 |
|  | rs1573496 | 0.878679 | 0.088409 | 0.198639 |
|  | rs13160562 | 1.082435 | 0.067269 | 0.202439 |
|  | rs1789891 | 1.100574 | 0.083655 | 0.207388 |
|  | rs1800759 | 1.074113 | 0.062557 | 0.219606 |
|  | rs3764435 | 0.933321 | 0.053743 | 0.230771 |
|  | rs4543123 | 0.920654 | 0.063762 | 0.232604 |
|  | rs886205 | 1.090594 | 0.080214 | 0.238365 |
|  | rs2303317 | 0.934716 | 0.053859 | 0.241325 |
|  | rs3930234 | 1.096018 | 0.086713 | 0.246519 |
|  | rs6902771 | 1.065624 | 0.060855 | 0.265708 |
|  | rs6701037 | 0.938207 | 0.054082 | 0.268499 |
|  | rs4440177 | 1.068321 | 0.064467 | 0.273428 |
|  | rs8040009 | 1.081469 | 0.079811 | 0.288573 |
|  | rs16985179 | 1.107121 | 0.106403 | 0.289674 |
|  | rs36061340 | 1.128859 | 0.130857 | 0.295741 |
|  | rs2228093 | 0.912466 | 0.081379 | 0.304365 |
|  | rs1380131 | 0.901077 | 0.0922 | 0.308674 |
|  | rs1230165 | 1.075706 | 0.078121 | 0.314957 |
|  | rs7553212 | 0.943489 | 0.057691 | 0.341436 |
|  | rs11851015 | 0.923197 | 0.079247 | 0.35188 |
|  | rs10908907 | 1.062039 | 0.069336 | 0.356552 |
|  | rs10893366 | 1.071199 | 0.08082 | 0.36198 |
|  | rs1229984 | 0.844667 | 0.162705 | 0.380828 |
|  | rs8062326 | 1.144371 | 0.184046 | 0.401743 |
|  | rs1908556 | 1.069684 | 0.086336 | 0.403936 |
|  | rs768048 | 1.070362 | 0.089991 | 0.418652 |
|  | rs1042026 | 0.95009 | 0.060549 | 0.421756 |
|  | rs3131513 | 0.954194 | 0.055894 | 0.423448 |
|  | rs4478858 | 0.955002 | 0.055757 | 0.430345 |
|  | rs59677118 | 0.921947 | 0.095759 | 0.433966 |
|  | rs9871864 | 0.957483 | 0.055381 | 0.452554 |
|  | rs4770403 | 1.054675 | 0.076276 | 0.461695 |
|  | rs10253361 | 0.958243 | 0.055551 | 0.461864 |
|  | rs2810114 | 1.047637 | 0.066977 | 0.466668 |
|  | rs3762894 | 0.945548 | 0.073376 | 0.470595 |
|  | rs284786 | 1.045292 | 0.065168 | 0.47739 |
|  | rs12388359 | 0.950977 | 0.067619 | 0.479615 |
|  | rs642899 | 1.045044 | 0.07022 | 0.512009 |
|  | rs2140418 | 0.953272 | 0.070908 | 0.519999 |
|  | rs2100290 | 0.964986 | 0.055354 | 0.534371 |
|  | rs567926 | 0.965054 | 0.056167 | 0.541084 |
|  | rs9512637 | 1.03638 | 0.062237 | 0.551808 |
|  | rs279861 | 0.966824 | 0.056117 | 0.561058 |
|  | rs195204 | 1.037027 | 0.068326 | 0.581067 |
|  | rs9825310 | 0.970489 | 0.056086 | 0.604221 |
|  | rs7144649 | 0.965138 | 0.067015 | 0.609331 |
|  | rs3819197 | 0.968219 | 0.065674 | 0.633967 |
|  | rs804292 | 1.030522 | 0.066261 | 0.640082 |
|  | rs1876831 | 0.968977 | 0.065583 | 0.64149 |
|  | rs750338 | 1.031896 | 0.070654 | 0.646549 |
|  | rs4761097 | 1.02568 | 0.059133 | 0.660078 |
|  | rs2548145 | 0.978087 | 0.056159 | 0.699581 |
|  | rs12472151 | 0.949918 | 0.13133 | 0.710169 |
|  | rs67031482 | 1.016455 | 0.058281 | 0.775914 |
|  | rs1497571 | 1.015097 | 0.05816 | 0.793692 |
|  | rs1353621 | 0.986735 | 0.059262 | 0.824051 |
|  | rs4758317 | 0.98756 | 0.057726 | 0.830425 |
|  | rs11724320 | 1.012955 | 0.061442 | 0.831945 |
|  | rs1353899 | 1.015262 | 0.073115 | 0.833417 |
|  | rs1793257 | 1.03167 | 0.155368 | 0.835986 |
|  | rs59972978 | 1.014011 | 0.077261 | 0.855108 |
|  | rs420817 | 1.006752 | 0.05766 | 0.906468 |
|  | rs13259667 | 0.987569 | 0.107788 | 0.908755 |
|  | rs933769 | 1.00851 | 0.074583 | 0.908777 |
|  | rs9656709 | 0.993843 | 0.057916 | 0.915599 |
|  | rs4293630 | 1.007402 | 0.084478 | 0.929921 |
|  | rs2154294 | 1.004709 | 0.057873 | 0.935002 |
|  | rs3738443 | 0.994358 | 0.076036 | 0.94102 |
|  | rs12311304 | 0.996842 | 0.061749 | 0.959273 |
| anxiety at age 10 | Offspring PGRS | 1.033496 | 0.754538 | 0.964006 |
|  | rs67031482 | 0.697469 | 0.109688 | 0.021962 |
|  | rs1353899 | 1.464899 | 0.256039 | 0.028936 |
|  | rs1573496 | 0.495957 | 0.172417 | 0.043675 |
|  | rs1824024 | 0.701182 | 0.12475 | 0.046011 |
|  | rs13259667 | 0.434272 | 0.181565 | 0.046044 |
|  | rs13160562 | 1.369261 | 0.221275 | 0.051808 |
|  | rs3819197 | 1.375067 | 0.230565 | 0.057497 |
|  | rs4478858 | 0.770888 | 0.124521 | 0.107196 |
|  | rs2100290 | 1.28142 | 0.199037 | 0.110388 |
|  | rs2228093 | 0.652549 | 0.174946 | 0.111336 |
|  | rs1497571 | 0.780158 | 0.121973 | 0.112308 |
|  | rs10849915 | 0.766534 | 0.132043 | 0.12272 |
|  | rs284786 | 1.270708 | 0.206733 | 0.140867 |
|  | rs1000579 | 1.256383 | 0.195202 | 0.141832 |
|  | rs6943555 | 0.758357 | 0.146691 | 0.152728 |
|  | rs6716455 | 1.333215 | 0.280171 | 0.171145 |
|  | rs886205 | 0.740387 | 0.164223 | 0.175368 |
|  | rs1876831 | 1.253103 | 0.216168 | 0.190903 |
|  | rs1800759 | 0.81337 | 0.131214 | 0.200377 |
|  | rs237238 | 0.655247 | 0.240373 | 0.249165 |
|  | rs1042026 | 1.198958 | 0.19668 | 0.268669 |
|  | rs6701037 | 0.845218 | 0.132874 | 0.284766 |
|  | rs7553212 | 0.839601 | 0.141538 | 0.299698 |
|  | rs1908556 | 1.228924 | 0.253127 | 0.316926 |
|  | rs3764435 | 0.857898 | 0.132497 | 0.321003 |
|  | rs567926 | 0.855058 | 0.13505 | 0.321487 |
|  | rs4758317 | 0.854866 | 0.135722 | 0.323304 |
|  | rs7144649 | 0.82695 | 0.161417 | 0.330336 |
|  | rs12472151 | 1.362032 | 0.432639 | 0.330692 |
|  | rs4543123 | 0.832988 | 0.160404 | 0.342642 |
|  | rs1793257 | 0.62507 | 0.316604 | 0.353561 |
|  | rs2380220 | 0.801725 | 0.19099 | 0.353588 |
|  | rs2140418 | 0.825034 | 0.174634 | 0.363538 |
|  | rs3930234 | 1.201103 | 0.248194 | 0.375204 |
|  | rs933769 | 0.828166 | 0.176173 | 0.37545 |
|  | rs2188561 | 1.167618 | 0.205706 | 0.379071 |
|  | rs3762894 | 1.185562 | 0.229943 | 0.380149 |
|  | rs279861 | 0.871567 | 0.136793 | 0.38112 |
|  | rs11724320 | 1.149028 | 0.184922 | 0.388043 |
|  | rs1230165 | 0.839851 | 0.176228 | 0.405545 |
|  | rs36563 | 0.832084 | 0.190388 | 0.42175 |
|  | rs1789891 | 0.838695 | 0.187166 | 0.430552 |
|  | rs768048 | 1.186033 | 0.25948 | 0.435482 |
|  | rs9556711 | 0.763407 | 0.280204 | 0.46203 |
|  | rs1864982 | 0.83678 | 0.204655 | 0.466254 |
|  | rs2154294 | 1.117886 | 0.173554 | 0.472881 |
|  | rs62202398 | 1.241458 | 0.37582 | 0.47494 |
|  | rs4761097 | 0.894636 | 0.139754 | 0.476012 |
|  | rs4440177 | 1.11946 | 0.178889 | 0.480078 |
|  | rs8040009 | 1.136409 | 0.224448 | 0.517349 |
|  | rs2827312 | 0.900094 | 0.155934 | 0.543472 |
|  | rs10893366 | 0.881982 | 0.190691 | 0.561341 |
|  | rs9656709 | 1.093743 | 0.1715 | 0.567689 |
|  | rs1353621 | 0.913132 | 0.149514 | 0.578892 |
|  | rs12388359 | 1.10149 | 0.193095 | 0.581355 |
|  | rs420817 | 1.085353 | 0.168181 | 0.597098 |
|  | rs6902771 | 0.924362 | 0.143424 | 0.612221 |
|  | rs59972978 | 0.900528 | 0.190387 | 0.620191 |
|  | rs9636231 | 1.08263 | 0.183153 | 0.638857 |
|  | rs3738443 | 1.098611 | 0.221887 | 0.64147 |
|  | rs36061340 | 1.147191 | 0.351803 | 0.654318 |
|  | rs1109501 | 1.0799 | 0.189412 | 0.661205 |
|  | rs750338 | 0.922749 | 0.174857 | 0.671364 |
|  | rs9825310 | 1.065095 | 0.1653 | 0.684488 |
|  | rs195204 | 1.073019 | 0.189887 | 0.690448 |
|  | rs12311304 | 1.066355 | 0.175978 | 0.697048 |
|  | rs2548145 | 1.061222 | 0.163605 | 0.699917 |
|  | rs4770403 | 0.930171 | 0.186622 | 0.718252 |
|  | rs2810114 | 0.938492 | 0.165369 | 0.71865 |
|  | rs10908907 | 0.93698 | 0.16971 | 0.719306 |
|  | rs2303317 | 0.949005 | 0.146201 | 0.734041 |
|  | rs4293630 | 0.926231 | 0.214115 | 0.740269 |
|  | rs804292 | 0.944714 | 0.167685 | 0.748652 |
|  | rs1344694 | 0.956946 | 0.161663 | 0.794475 |
|  | rs1318937 | 0.943265 | 0.220072 | 0.802321 |
|  | rs1229984 | 0.884935 | 0.453584 | 0.811501 |
|  | rs59677118 | 1.056321 | 0.282019 | 0.837396 |
|  | rs3131513 | 0.969266 | 0.153202 | 0.843438 |
|  | rs7590720 | 0.968587 | 0.168164 | 0.854143 |
|  | rs10253361 | 1.026811 | 0.159712 | 0.864928 |
|  | rs242938 | 1.046172 | 0.315981 | 0.881201 |
|  | rs11851015 | 0.970272 | 0.221428 | 0.894793 |
|  | rs16985179 | 1.027185 | 0.270871 | 0.918983 |
|  | rs9871864 | 0.986215 | 0.15324 | 0.928815 |
|  | rs2369955 | 1.020983 | 0.239695 | 0.929518 |
|  | rs9512637 | 0.988663 | 0.159969 | 0.943821 |
|  | rs8062326 | 0.974409 | 0.445125 | 0.954744 |
|  | rs642899 | 0.989891 | 0.181446 | 0.955796 |
|  | rs1380131 | 1.000495 | 0.265387 | 0.998511 |
| anxiety at age 13 | Offspring PGRS | 1.095724 | 1.048528 | 0.923894 |
|  | rs1230165 | 0.464017 | 0.15511 | 0.021618 |
|  | rs9871864 | 1.589447 | 0.321147 | 0.021823 |
|  | rs1793257 | 2.266217 | 0.835632 | 0.026507 |
|  | rs768048 | 0.390228 | 0.165735 | 0.026714 |
|  | rs4758317 | 1.517756 | 0.309159 | 0.040528 |
|  | rs242938 | 1.866867 | 0.574326 | 0.04244 |
|  | rs1573496 | 0.362788 | 0.186527 | 0.048602 |
|  | rs1824024 | 1.439802 | 0.292437 | 0.072713 |
|  | rs1497571 | 0.696906 | 0.141393 | 0.075102 |
|  | rs12472151 | 1.755646 | 0.658228 | 0.1333 |
|  | rs1229984 | 1.971049 | 0.92178 | 0.146785 |
|  | rs4440177 | 1.33869 | 0.269762 | 0.147752 |
|  | rs9512637 | 0.730971 | 0.160128 | 0.152555 |
|  | rs7590720 | 1.349232 | 0.283879 | 0.154549 |
|  | rs59677118 | 1.489167 | 0.445007 | 0.182667 |
|  | rs1876831 | 1.335095 | 0.291375 | 0.185428 |
|  | rs1864982 | 0.63081 | 0.220863 | 0.188188 |
|  | rs2827312 | 0.761672 | 0.173781 | 0.232787 |
|  | rs7144649 | 1.300074 | 0.288728 | 0.237356 |
|  | rs6701037 | 0.788064 | 0.16003 | 0.24084 |
|  | rs1318937 | 1.359236 | 0.36117 | 0.248057 |
|  | rs1800759 | 0.787871 | 0.164661 | 0.253954 |
|  | rs12388359 | 0.717321 | 0.210237 | 0.256977 |
|  | rs2548145 | 1.248871 | 0.24843 | 0.263902 |
|  | rs1042026 | 1.263315 | 0.264596 | 0.264428 |
|  | rs3131513 | 0.805857 | 0.168018 | 0.300544 |
|  | rs1789891 | 0.734983 | 0.219599 | 0.302754 |
|  | rs13160562 | 1.236219 | 0.259894 | 0.313129 |
|  | rs11724320 | 0.812803 | 0.177612 | 0.342869 |
|  | rs2369955 | 1.29596 | 0.361986 | 0.353327 |
|  | rs10908907 | 1.224558 | 0.267794 | 0.354264 |
|  | rs933769 | 0.77935 | 0.217443 | 0.371583 |
|  | rs1344694 | 1.20209 | 0.251166 | 0.378357 |
|  | rs3738443 | 0.775868 | 0.224736 | 0.380967 |
|  | rs13259667 | 1.322544 | 0.444901 | 0.405956 |
|  | rs4543123 | 0.815166 | 0.203826 | 0.413748 |
|  | rs2228093 | 1.255146 | 0.350721 | 0.416059 |
|  | rs3819197 | 1.196224 | 0.266353 | 0.421006 |
|  | rs2140418 | 1.176288 | 0.285501 | 0.503525 |
|  | rs8062326 | 0.62071 | 0.444288 | 0.505245 |
|  | rs9825310 | 0.875172 | 0.175733 | 0.506675 |
|  | rs2380220 | 1.189912 | 0.314805 | 0.511029 |
|  | rs279861 | 0.876486 | 0.176648 | 0.513027 |
|  | rs16985179 | 1.226465 | 0.387894 | 0.518637 |
|  | rs2188561 | 1.156581 | 0.263122 | 0.522549 |
|  | rs1109501 | 0.860756 | 0.204163 | 0.527277 |
|  | rs4293630 | 0.823362 | 0.254338 | 0.529222 |
|  | rs567926 | 0.881384 | 0.178347 | 0.532639 |
|  | rs6716455 | 1.166301 | 0.329596 | 0.586189 |
|  | rs1380131 | 0.818592 | 0.303351 | 0.589088 |
|  | rs2100290 | 1.111291 | 0.221482 | 0.596488 |
|  | rs1353621 | 0.894655 | 0.188485 | 0.59724 |
|  | rs2303317 | 1.110024 | 0.220522 | 0.599293 |
|  | rs284786 | 1.111749 | 0.238038 | 0.620765 |
|  | rs4478858 | 0.906182 | 0.184108 | 0.627753 |
|  | rs1353899 | 0.883201 | 0.228771 | 0.631583 |
|  | rs7553212 | 0.905773 | 0.193599 | 0.643345 |
|  | rs10849915 | 1.102011 | 0.231678 | 0.644049 |
|  | rs10253361 | 1.095076 | 0.218857 | 0.649508 |
|  | rs8040009 | 1.119253 | 0.28482 | 0.657966 |
|  | rs67031482 | 0.918919 | 0.182875 | 0.670919 |
|  | rs1908556 | 0.884996 | 0.262109 | 0.679968 |
|  | rs12311304 | 1.091241 | 0.231051 | 0.680057 |
|  | rs11851015 | 0.898516 | 0.270033 | 0.721788 |
|  | rs886205 | 0.923887 | 0.245798 | 0.766039 |
|  | rs3764435 | 1.058389 | 0.210716 | 0.775617 |
|  | rs9656709 | 1.058015 | 0.213352 | 0.779738 |
|  | rs6943555 | 0.937639 | 0.220699 | 0.78442 |
|  | rs750338 | 0.936192 | 0.226586 | 0.785297 |
|  | rs237238 | 1.100114 | 0.412554 | 0.799164 |
|  | rs9636231 | 0.951838 | 0.213204 | 0.825584 |
|  | rs1000579 | 1.041955 | 0.211691 | 0.839688 |
|  | rs2810114 | 1.041137 | 0.231184 | 0.855935 |
|  | rs642899 | 0.959249 | 0.227208 | 0.860568 |
|  | rs36563 | 1.048411 | 0.286007 | 0.862417 |
|  | rs10893366 | 1.035319 | 0.271152 | 0.894565 |
|  | rs59972978 | 0.968176 | 0.259066 | 0.903798 |
|  | rs36061340 | 0.95154 | 0.400854 | 0.906135 |
|  | rs3930234 | 0.973184 | 0.275082 | 0.92339 |
|  | rs804292 | 0.979516 | 0.2203 | 0.92668 |
|  | rs9556711 | 0.967545 | 0.413082 | 0.938401 |
|  | rs4761097 | 1.012911 | 0.20195 | 0.948697 |
|  | rs420817 | 0.991398 | 0.197689 | 0.965444 |
|  | rs2154294 | 0.992476 | 0.198646 | 0.969899 |
|  | rs4770403 | 1.007378 | 0.255489 | 0.976879 |
|  | rs6902771 | 0.997052 | 0.197567 | 0.988112 |
|  | rs62202398 | 1.00615 | 0.426695 | 0.988465 |
|  | rs3762894 | 1.003425 | 0.266411 | 0.989724 |
|  | rs195204 | 1.001654 | 0.230684 | 0.994274 |
| anxiety at age 15 | Offspring PGRS | 0.991813 | 0.770581 | 0.991558 |
|  | rs3131513 | 1.561904 | 0.255203 | 0.006352 |
|  | rs7144649 | 1.5309 | 0.271353 | 0.016281 |
|  | rs6943555 | 1.476144 | 0.259633 | 0.02682 |
|  | rs7553212 | 0.761704 | 0.138317 | 0.13388 |
|  | rs420817 | 0.780817 | 0.129143 | 0.134678 |
|  | rs2228093 | 0.658923 | 0.188413 | 0.144603 |
|  | rs1344694 | 1.268234 | 0.217933 | 0.166716 |
|  | rs195204 | 0.756554 | 0.154199 | 0.171066 |
|  | rs933769 | 1.30713 | 0.256207 | 0.171798 |
|  | rs3930234 | 0.717923 | 0.18551 | 0.199672 |
|  | rs2548145 | 1.234012 | 0.202479 | 0.20002 |
|  | rs1000579 | 0.804242 | 0.139383 | 0.208745 |
|  | rs3738443 | 1.290151 | 0.26522 | 0.215248 |
|  | rs12472151 | 0.533007 | 0.272885 | 0.219067 |
|  | rs567926 | 1.215288 | 0.197662 | 0.230603 |
|  | rs8040009 | 1.266915 | 0.255945 | 0.241564 |
|  | rs7590720 | 1.226881 | 0.215976 | 0.245418 |
|  | rs2100290 | 1.207059 | 0.197978 | 0.25123 |
|  | rs12388359 | 0.776453 | 0.175899 | 0.264049 |
|  | rs1789891 | 0.765365 | 0.185374 | 0.269576 |
|  | rs2140418 | 0.790459 | 0.178363 | 0.297372 |
|  | rs1908556 | 0.766959 | 0.198293 | 0.304791 |
|  | rs9871864 | 1.17185 | 0.190739 | 0.329908 |
|  | rs2154294 | 0.852199 | 0.141018 | 0.333781 |
|  | rs279861 | 1.168151 | 0.189282 | 0.337465 |
|  | rs4478858 | 0.852546 | 0.144603 | 0.34694 |
|  | rs4761097 | 0.856906 | 0.142274 | 0.352317 |
|  | rs36061340 | 0.703574 | 0.275364 | 0.369017 |
|  | rs3819197 | 0.838031 | 0.16795 | 0.377942 |
|  | rs2188561 | 0.836161 | 0.17245 | 0.385614 |
|  | rs1800759 | 1.140279 | 0.18626 | 0.421597 |
|  | rs9825310 | 1.13774 | 0.185279 | 0.428117 |
|  | rs6701037 | 1.137867 | 0.186345 | 0.430314 |
|  | rs1864982 | 0.81775 | 0.211714 | 0.43708 |
|  | rs4293630 | 0.822673 | 0.209146 | 0.442605 |
|  | rs59972978 | 0.838345 | 0.193936 | 0.445929 |
|  | rs10908907 | 0.866023 | 0.167117 | 0.456021 |
|  | rs237238 | 1.246001 | 0.369911 | 0.458791 |
|  | rs59677118 | 0.790691 | 0.251987 | 0.461176 |
|  | rs6716455 | 1.185279 | 0.2774 | 0.467663 |
|  | rs3764435 | 0.896471 | 0.145436 | 0.500526 |
|  | rs1497571 | 0.895985 | 0.146508 | 0.501782 |
|  | rs886205 | 1.138353 | 0.233887 | 0.528242 |
|  | rs10893366 | 0.86803 | 0.197785 | 0.534511 |
|  | rs1318937 | 1.156705 | 0.271204 | 0.534671 |
|  | rs4440177 | 1.110002 | 0.189771 | 0.541577 |
|  | rs62202398 | 0.813977 | 0.306708 | 0.584903 |
|  | rs9636231 | 0.906243 | 0.16745 | 0.594171 |
|  | rs9556711 | 1.183434 | 0.376166 | 0.596211 |
|  | rs13160562 | 0.907687 | 0.166095 | 0.596597 |
|  | rs3762894 | 1.112905 | 0.232755 | 0.609011 |
|  | rs1230165 | 1.109887 | 0.226388 | 0.609256 |
|  | rs1876831 | 1.097753 | 0.20585 | 0.618932 |
|  | rs4770403 | 0.906356 | 0.194892 | 0.647486 |
|  | rs2380220 | 0.897957 | 0.215455 | 0.653731 |
|  | rs11724320 | 1.077397 | 0.18647 | 0.666668 |
|  | rs13259667 | 0.871196 | 0.286454 | 0.674952 |
|  | rs67031482 | 0.935656 | 0.152701 | 0.683629 |
|  | rs12311304 | 0.930297 | 0.16583 | 0.685237 |
|  | rs16985179 | 1.116266 | 0.303288 | 0.68561 |
|  | rs1229984 | 0.78944 | 0.464781 | 0.687991 |
|  | rs750338 | 0.924194 | 0.184571 | 0.693037 |
|  | rs10253361 | 0.940047 | 0.155607 | 0.708776 |
|  | rs242938 | 1.117414 | 0.353272 | 0.725473 |
|  | rs642899 | 0.933636 | 0.184096 | 0.727653 |
|  | rs1109501 | 1.06008 | 0.195373 | 0.751567 |
|  | rs4758317 | 0.949608 | 0.158202 | 0.756284 |
|  | rs768048 | 0.931191 | 0.233282 | 0.775973 |
|  | rs1380131 | 1.077417 | 0.296395 | 0.78635 |
|  | rs6902771 | 0.956771 | 0.156367 | 0.786859 |
|  | rs8062326 | 1.118552 | 0.510581 | 0.806116 |
|  | rs4543123 | 0.954881 | 0.187798 | 0.814405 |
|  | rs1824024 | 1.040506 | 0.179724 | 0.818185 |
|  | rs2369955 | 1.049431 | 0.258591 | 0.844763 |
|  | rs11851015 | 0.95315 | 0.23412 | 0.845119 |
|  | rs1793257 | 1.067766 | 0.448641 | 0.875991 |
|  | rs9512637 | 0.973624 | 0.167309 | 0.876388 |
|  | rs2810114 | 1.027187 | 0.187763 | 0.883331 |
|  | rs10849915 | 1.021855 | 0.177912 | 0.901177 |
|  | rs1042026 | 0.978516 | 0.176722 | 0.90428 |
|  | rs9656709 | 0.980395 | 0.162592 | 0.904968 |
|  | rs2827312 | 1.018226 | 0.183831 | 0.92031 |
|  | rs1353621 | 0.9871 | 0.168153 | 0.939247 |
|  | rs1573496 | 0.980105 | 0.269935 | 0.941834 |
|  | rs2303317 | 1.007985 | 0.163379 | 0.960864 |
|  | rs284786 | 1.006353 | 0.179367 | 0.971656 |
|  | rs36563 | 0.993149 | 0.22687 | 0.975991 |
|  | rs804292 | 1.003972 | 0.184592 | 0.982798 |
|  | rs1353899 | 0.997892 | 0.20311 | 0.991729 |
| Anxiety at age 7 | Offspring PGRS | 1.137239 | 0.944873 | 0.876991 |
|  | rs4293630 | 0.439567 | 0.152845 | 0.018084 |
|  | rs9825310 | 0.656283 | 0.11826 | 0.019426 |
|  | rs10849915 | 0.651313 | 0.130732 | 0.032669 |
|  | rs10908907 | 0.658269 | 0.146764 | 0.06073 |
|  | rs195204 | 1.412509 | 0.263362 | 0.063978 |
|  | rs1000579 | 1.345805 | 0.233397 | 0.086804 |
|  | rs59972978 | 1.425343 | 0.298513 | 0.090598 |
|  | rs1789891 | 1.402309 | 0.301381 | 0.11566 |
|  | rs9512637 | 0.751805 | 0.141632 | 0.129949 |
|  | rs2369955 | 1.428561 | 0.336616 | 0.130113 |
|  | rs1497571 | 0.770231 | 0.134707 | 0.135511 |
|  | rs242938 | 1.516635 | 0.44306 | 0.153955 |
|  | rs13259667 | 0.551708 | 0.231886 | 0.157065 |
|  | rs4770403 | 0.712777 | 0.176712 | 0.172032 |
|  | rs3930234 | 0.687687 | 0.19081 | 0.177199 |
|  | rs36563 | 1.3405 | 0.294102 | 0.181657 |
|  | rs4440177 | 1.259989 | 0.221231 | 0.188103 |
|  | rs2228093 | 1.36762 | 0.325495 | 0.188368 |
|  | rs9871864 | 1.240961 | 0.21462 | 0.211928 |
|  | rs4758317 | 1.240748 | 0.216804 | 0.217013 |
|  | rs1318937 | 0.698801 | 0.203683 | 0.218858 |
|  | rs1109501 | 1.256622 | 0.237693 | 0.227187 |
|  | rs567926 | 1.225414 | 0.212052 | 0.24011 |
|  | rs6902771 | 0.815837 | 0.142366 | 0.243453 |
|  | rs1864982 | 0.739115 | 0.209278 | 0.285677 |
|  | rs420817 | 0.839986 | 0.146137 | 0.316213 |
|  | rs6943555 | 0.812384 | 0.171404 | 0.324721 |
|  | rs1793257 | 0.570775 | 0.334506 | 0.33865 |
|  | rs7553212 | 0.836519 | 0.157467 | 0.342985 |
|  | rs933769 | 0.798622 | 0.192515 | 0.350906 |
|  | rs9636231 | 1.188247 | 0.220338 | 0.352292 |
|  | rs36061340 | 0.683394 | 0.29021 | 0.370016 |
|  | rs4478858 | 1.160603 | 0.202172 | 0.392544 |
|  | rs1800759 | 1.156919 | 0.2004 | 0.400078 |
|  | rs279861 | 1.155197 | 0.199522 | 0.403548 |
|  | rs1876831 | 1.171307 | 0.229563 | 0.41979 |
|  | rs62202398 | 0.715463 | 0.303935 | 0.430592 |
|  | rs12311304 | 0.860534 | 0.164974 | 0.433344 |
|  | rs237238 | 1.272205 | 0.393321 | 0.436147 |
|  | rs9656709 | 1.139395 | 0.198209 | 0.453157 |
|  | rs12388359 | 1.152577 | 0.219707 | 0.456315 |
|  | rs16985179 | 0.786779 | 0.259825 | 0.467739 |
|  | rs750338 | 0.856684 | 0.185193 | 0.474263 |
|  | rs67031482 | 0.891417 | 0.153298 | 0.503888 |
|  | rs1380131 | 0.825242 | 0.261432 | 0.544304 |
|  | rs3762894 | 0.866716 | 0.209895 | 0.554742 |
|  | rs9556711 | 1.205283 | 0.403774 | 0.577287 |
|  | rs8062326 | 0.728862 | 0.42689 | 0.589202 |
|  | rs642899 | 0.899762 | 0.189808 | 0.616579 |
|  | rs2154294 | 1.08827 | 0.188172 | 0.624692 |
|  | rs2548145 | 0.920397 | 0.15897 | 0.63104 |
|  | rs3764435 | 1.083102 | 0.186267 | 0.642512 |
|  | rs59677118 | 0.862116 | 0.278995 | 0.646622 |
|  | rs10893366 | 0.900453 | 0.216769 | 0.663145 |
|  | rs2810114 | 1.084486 | 0.206649 | 0.67037 |
|  | rs2188561 | 1.08134 | 0.217207 | 0.697042 |
|  | rs1824024 | 1.073338 | 0.19627 | 0.698728 |
|  | rs3738443 | 0.914491 | 0.219297 | 0.709331 |
|  | rs886205 | 1.084342 | 0.238484 | 0.712748 |
|  | rs1042026 | 0.937073 | 0.180285 | 0.735496 |
|  | rs10253361 | 1.060174 | 0.18354 | 0.735723 |
|  | rs4543123 | 1.066616 | 0.214303 | 0.748225 |
|  | rs8040009 | 1.071634 | 0.238389 | 0.755795 |
|  | rs284786 | 0.945405 | 0.180307 | 0.768475 |
|  | rs3131513 | 0.950132 | 0.167497 | 0.771683 |
|  | rs7144649 | 0.941739 | 0.198509 | 0.775818 |
|  | rs1230165 | 1.063272 | 0.232939 | 0.779445 |
|  | rs2100290 | 0.957401 | 0.164655 | 0.800173 |
|  | rs1344694 | 1.046933 | 0.193011 | 0.803531 |
|  | rs804292 | 0.952716 | 0.18847 | 0.806567 |
|  | rs1908556 | 0.943052 | 0.237486 | 0.815892 |
|  | rs1229984 | 1.125418 | 0.579193 | 0.818415 |
|  | rs11724320 | 1.042356 | 0.188857 | 0.818899 |
|  | rs6716455 | 0.945384 | 0.248171 | 0.830583 |
|  | rs2303317 | 1.037081 | 0.178466 | 0.832434 |
|  | rs12472151 | 0.917016 | 0.386912 | 0.837321 |
|  | rs2140418 | 0.965849 | 0.216374 | 0.876736 |
|  | rs2380220 | 0.969236 | 0.239743 | 0.899473 |
|  | rs1573496 | 1.029344 | 0.295528 | 0.919759 |
|  | rs3819197 | 0.980278 | 0.199474 | 0.922022 |
|  | rs6701037 | 0.984633 | 0.169955 | 0.928508 |
|  | rs1353899 | 1.018391 | 0.220689 | 0.932982 |
|  | rs4761097 | 1.014284 | 0.175658 | 0.934731 |
|  | rs1353621 | 0.989336 | 0.178534 | 0.952623 |
|  | rs13160562 | 1.009656 | 0.189942 | 0.95926 |
|  | rs11851015 | 1.011543 | 0.253967 | 0.96354 |
|  | rs7590720 | 1.005356 | 0.191863 | 0.977669 |
|  | rs768048 | 0.994637 | 0.257165 | 0.983407 |
|  | rs2827312 | 1.003114 | 0.192036 | 0.987043 |
| binge eating at age 13 | Offspring PGRS | 1.220283 | 0.840875 | 0.77265 |
|  | rs1109501 | 0.560005 | 0.110161 | 0.003204 |
|  | rs1042026 | 1.397535 | 0.210925 | 0.026575 |
|  | rs1908556 | 0.614826 | 0.151907 | 0.048986 |
|  | rs9871864 | 1.306722 | 0.188889 | 0.064213 |
|  | rs9556711 | 1.59989 | 0.407906 | 0.065303 |
|  | rs7553212 | 0.747391 | 0.120528 | 0.070992 |
|  | rs804292 | 1.311961 | 0.20088 | 0.076173 |
|  | rs9825310 | 0.785026 | 0.115335 | 0.099471 |
|  | rs2140418 | 0.732132 | 0.14953 | 0.126857 |
|  | rs10908907 | 0.773345 | 0.137617 | 0.14863 |
|  | rs642899 | 0.771679 | 0.141168 | 0.156538 |
|  | rs10849915 | 1.234215 | 0.185302 | 0.16103 |
|  | rs2380220 | 1.296677 | 0.24302 | 0.165675 |
|  | rs7590720 | 1.235025 | 0.191214 | 0.172755 |
|  | rs886205 | 1.262867 | 0.221729 | 0.183765 |
|  | rs8040009 | 1.253853 | 0.224274 | 0.205964 |
|  | rs3819197 | 1.220292 | 0.198512 | 0.221011 |
|  | rs7144649 | 0.801981 | 0.147664 | 0.230727 |
|  | rs2827312 | 1.207091 | 0.189678 | 0.231007 |
|  | rs4440177 | 0.829658 | 0.131036 | 0.23706 |
|  | rs1353621 | 0.836715 | 0.128996 | 0.247544 |
|  | rs1344694 | 1.187761 | 0.180773 | 0.258232 |
|  | rs12472151 | 1.395249 | 0.411574 | 0.258844 |
|  | rs62202398 | 0.667736 | 0.242531 | 0.266176 |
|  | rs3762894 | 1.213303 | 0.220323 | 0.286991 |
|  | rs768048 | 1.239142 | 0.249655 | 0.287216 |
|  | rs13160562 | 1.1768 | 0.181468 | 0.291091 |
|  | rs3930234 | 0.799471 | 0.175145 | 0.306976 |
|  | rs750338 | 1.176319 | 0.194505 | 0.326052 |
|  | rs1229984 | 0.561802 | 0.329954 | 0.326213 |
|  | rs1789891 | 0.814221 | 0.171314 | 0.328663 |
|  | rs2188561 | 1.16663 | 0.191397 | 0.347519 |
|  | rs284786 | 0.859323 | 0.139515 | 0.350395 |
|  | rs59972978 | 1.182049 | 0.216242 | 0.360593 |
|  | rs279861 | 0.874997 | 0.128447 | 0.363003 |
|  | rs1824024 | 0.866868 | 0.13703 | 0.3661 |
|  | rs3131513 | 1.134059 | 0.164538 | 0.385896 |
|  | rs6943555 | 1.150683 | 0.186686 | 0.386975 |
|  | rs567926 | 0.881709 | 0.130168 | 0.393799 |
|  | rs8062326 | 0.652781 | 0.33163 | 0.401161 |
|  | rs1793257 | 0.683948 | 0.310304 | 0.402431 |
|  | rs59677118 | 0.793666 | 0.222322 | 0.409385 |
|  | rs2228093 | 1.18557 | 0.244872 | 0.409854 |
|  | rs36563 | 1.165097 | 0.21997 | 0.418317 |
|  | rs1497571 | 0.889914 | 0.128365 | 0.418765 |
|  | rs13259667 | 1.219293 | 0.307139 | 0.431221 |
|  | rs9512637 | 0.88723 | 0.135932 | 0.434822 |
|  | rs2303317 | 0.899681 | 0.129684 | 0.463318 |
|  | rs4758317 | 1.112603 | 0.162579 | 0.465259 |
|  | rs2154294 | 1.111134 | 0.160995 | 0.467039 |
|  | rs6716455 | 1.157553 | 0.236691 | 0.474281 |
|  | rs420817 | 0.909515 | 0.131421 | 0.511582 |
|  | rs237238 | 1.182913 | 0.31714 | 0.530951 |
|  | rs2100290 | 0.91565 | 0.133071 | 0.544277 |
|  | rs11724320 | 1.08317 | 0.16338 | 0.596345 |
|  | rs67031482 | 1.078496 | 0.156019 | 0.601412 |
|  | rs16985179 | 0.875091 | 0.227497 | 0.607783 |
|  | rs1000579 | 1.077612 | 0.157979 | 0.610144 |
|  | rs4761097 | 0.929925 | 0.134714 | 0.61601 |
|  | rs10893366 | 1.096009 | 0.204721 | 0.623569 |
|  | rs2548145 | 0.933345 | 0.134529 | 0.632241 |
|  | rs4293630 | 0.902215 | 0.195793 | 0.635374 |
|  | rs1318937 | 1.099479 | 0.229143 | 0.649077 |
|  | rs1573496 | 0.891914 | 0.224418 | 0.649392 |
|  | rs11851015 | 0.907951 | 0.197797 | 0.657573 |
|  | rs6902771 | 0.940314 | 0.135848 | 0.670126 |
|  | rs1864982 | 0.916147 | 0.2008 | 0.689469 |
|  | rs12388359 | 0.930497 | 0.168498 | 0.690771 |
|  | rs9636231 | 1.062715 | 0.168567 | 0.701365 |
|  | rs1876831 | 0.937659 | 0.160424 | 0.706747 |
|  | rs4770403 | 1.059895 | 0.191693 | 0.747735 |
|  | rs1230165 | 1.054881 | 0.191179 | 0.768144 |
|  | rs4543123 | 0.9513 | 0.166205 | 0.775063 |
|  | rs9656709 | 0.959591 | 0.140268 | 0.777804 |
|  | rs4478858 | 1.041846 | 0.152302 | 0.77915 |
|  | rs1353899 | 0.953581 | 0.174132 | 0.79464 |
|  | rs36061340 | 1.072254 | 0.312804 | 0.810997 |
|  | rs2369955 | 0.953987 | 0.2137 | 0.833447 |
|  | rs933769 | 1.038391 | 0.189826 | 0.836731 |
|  | rs1800759 | 0.971852 | 0.142617 | 0.845733 |
|  | rs3764435 | 0.973439 | 0.13999 | 0.851508 |
|  | rs12311304 | 1.027917 | 0.15902 | 0.858736 |
|  | rs1380131 | 1.039515 | 0.254761 | 0.874355 |
|  | rs2810114 | 0.9833 | 0.159926 | 0.91753 |
|  | rs195204 | 1.009577 | 0.168956 | 0.954583 |
|  | rs242938 | 0.985628 | 0.284623 | 0.960018 |
|  | rs3738443 | 0.995246 | 0.192977 | 0.980393 |
|  | rs10253361 | 0.996879 | 0.144824 | 0.982831 |
|  | rs6701037 | 1.000325 | 0.145091 | 0.998214 |
| binge eating at age 14 | Offspring PGRS | 1.291018 | 0.37227 | 0.375712 |
|  | rs1353899 | 1.264517 | 0.091486 | 0.001179 |
|  | rs59972978 | 1.25831 | 0.097933 | 0.003155 |
|  | rs13259667 | 0.741409 | 0.092449 | 0.016418 |
|  | rs1353621 | 1.161972 | 0.073034 | 0.016922 |
|  | rs16985179 | 1.23227 | 0.122666 | 0.035893 |
|  | rs1230165 | 1.172419 | 0.088927 | 0.035977 |
|  | rs9512637 | 0.890565 | 0.057599 | 0.073138 |
|  | rs3930234 | 0.865232 | 0.076737 | 0.102641 |
|  | rs1793257 | 1.26696 | 0.186883 | 0.108681 |
|  | rs9656709 | 1.101432 | 0.067693 | 0.11596 |
|  | rs2380220 | 0.873437 | 0.077785 | 0.12864 |
|  | rs6701037 | 1.094228 | 0.066498 | 0.138407 |
|  | rs10893366 | 0.888432 | 0.073936 | 0.155176 |
|  | rs1864982 | 1.128715 | 0.096902 | 0.158441 |
|  | rs9871864 | 1.090101 | 0.066713 | 0.158641 |
|  | rs4543123 | 0.904222 | 0.066347 | 0.17002 |
|  | rs9556711 | 1.171688 | 0.142427 | 0.192417 |
|  | rs642899 | 0.911804 | 0.066007 | 0.202158 |
|  | rs1109501 | 1.091689 | 0.075121 | 0.202355 |
|  | rs2303317 | 1.078257 | 0.065217 | 0.212864 |
|  | rs11851015 | 0.895566 | 0.081514 | 0.225583 |
|  | rs1380131 | 0.881379 | 0.094636 | 0.239605 |
|  | rs1229984 | 0.779538 | 0.16593 | 0.241979 |
|  | rs237238 | 1.142456 | 0.131495 | 0.247234 |
|  | rs3738443 | 1.08894 | 0.087208 | 0.287364 |
|  | rs1908556 | 0.910211 | 0.081028 | 0.290599 |
|  | rs1573496 | 0.902585 | 0.093525 | 0.322599 |
|  | rs13160562 | 1.066361 | 0.070641 | 0.332092 |
|  | rs2810114 | 0.936346 | 0.064378 | 0.338778 |
|  | rs195204 | 1.062541 | 0.074324 | 0.385807 |
|  | rs4293630 | 0.925652 | 0.083496 | 0.391728 |
|  | rs804292 | 0.944105 | 0.064786 | 0.401926 |
|  | rs1789891 | 0.933041 | 0.077831 | 0.406056 |
|  | rs9636231 | 1.056648 | 0.070678 | 0.410063 |
|  | rs768048 | 0.928936 | 0.085705 | 0.424301 |
|  | rs2369955 | 0.926824 | 0.08837 | 0.425456 |
|  | rs59677118 | 0.916805 | 0.100894 | 0.429943 |
|  | rs3819197 | 0.945425 | 0.068117 | 0.436022 |
|  | rs7553212 | 1.050433 | 0.067243 | 0.442129 |
|  | rs36563 | 0.938342 | 0.080718 | 0.45941 |
|  | rs4770403 | 0.944359 | 0.074413 | 0.467513 |
|  | rs2228093 | 1.066973 | 0.097069 | 0.476118 |
|  | rs3131513 | 0.959917 | 0.059523 | 0.509429 |
|  | rs1042026 | 0.958037 | 0.064373 | 0.52347 |
|  | rs9825310 | 1.038262 | 0.063245 | 0.537624 |
|  | rs10908907 | 1.043331 | 0.07204 | 0.538989 |
|  | rs62202398 | 0.924552 | 0.121698 | 0.5512 |
|  | rs1344694 | 0.961772 | 0.063663 | 0.555962 |
|  | rs12472151 | 0.919067 | 0.136748 | 0.570568 |
|  | rs3764435 | 1.032257 | 0.062232 | 0.598468 |
|  | rs420817 | 1.028773 | 0.062473 | 0.6404 |
|  | rs6943555 | 1.032768 | 0.072149 | 0.644414 |
|  | rs2140418 | 0.964997 | 0.076544 | 0.653288 |
|  | rs1876831 | 0.969073 | 0.069193 | 0.659946 |
|  | rs10849915 | 0.972122 | 0.06247 | 0.659954 |
|  | rs7144649 | 1.031499 | 0.074873 | 0.669195 |
|  | rs4761097 | 0.974595 | 0.05891 | 0.670306 |
|  | rs284786 | 1.025577 | 0.067872 | 0.702743 |
|  | rs567926 | 0.977788 | 0.059877 | 0.713766 |
|  | rs2100290 | 0.979764 | 0.059667 | 0.737097 |
|  | rs1000579 | 1.020664 | 0.063213 | 0.741207 |
|  | rs67031482 | 1.018743 | 0.062006 | 0.760302 |
|  | rs2827312 | 0.980704 | 0.065897 | 0.771831 |
|  | rs2188561 | 0.979617 | 0.071388 | 0.777491 |
|  | rs36061340 | 1.034246 | 0.127036 | 0.783979 |
|  | rs279861 | 0.984209 | 0.060102 | 0.794364 |
|  | rs3762894 | 1.020988 | 0.081856 | 0.79558 |
|  | rs10253361 | 1.015851 | 0.06247 | 0.798153 |
|  | rs1497571 | 0.984813 | 0.059462 | 0.79991 |
|  | rs1824024 | 1.015341 | 0.065786 | 0.81423 |
|  | rs2548145 | 1.013256 | 0.061552 | 0.82838 |
|  | rs2154294 | 1.013004 | 0.061912 | 0.832573 |
|  | rs242938 | 1.024817 | 0.122731 | 0.837808 |
|  | rs750338 | 0.986772 | 0.071582 | 0.854352 |
|  | rs4440177 | 0.98847 | 0.063618 | 0.857006 |
|  | rs886205 | 1.013001 | 0.080097 | 0.870234 |
|  | rs4758317 | 0.992078 | 0.06132 | 0.89761 |
|  | rs8040009 | 1.008206 | 0.080264 | 0.918235 |
|  | rs6902771 | 1.005691 | 0.061057 | 0.925525 |
|  | rs1318937 | 0.992256 | 0.089697 | 0.93147 |
|  | rs8062326 | 0.985542 | 0.17438 | 0.9344 |
|  | rs7590720 | 1.002219 | 0.068148 | 0.973999 |
|  | rs6716455 | 0.997381 | 0.09043 | 0.976926 |
|  | rs4478858 | 0.998617 | 0.061757 | 0.98215 |
|  | rs12311304 | 1.001249 | 0.065519 | 0.984784 |
|  | rs11724320 | 0.999141 | 0.064307 | 0.989347 |
|  | rs1800759 | 1.000485 | 0.061434 | 0.993693 |
|  | rs12388359 | 0.999428 | 0.072441 | 0.993697 |
|  | rs933769 | 0.999566 | 0.077397 | 0.99553 |
| binge eating at age 16 | Offspring PGRS | 1.398356 | 0.357019 | 0.189089 |
|  | rs3819197 | 0.830838 | 0.05463 | 0.004826 |
|  | rs2827312 | 1.168493 | 0.068849 | 0.008223 |
|  | rs67031482 | 0.868137 | 0.0473 | 0.00945 |
|  | rs4770403 | 0.830845 | 0.059761 | 0.009985 |
|  | rs4293630 | 0.815141 | 0.066301 | 0.011973 |
|  | rs3762894 | 0.839084 | 0.062045 | 0.01766 |
|  | rs1042026 | 0.877295 | 0.053079 | 0.030486 |
|  | rs7553212 | 0.883525 | 0.050617 | 0.030651 |
|  | rs1864982 | 1.160527 | 0.088972 | 0.052152 |
|  | rs1380131 | 1.16758 | 0.106598 | 0.089697 |
|  | rs9636231 | 1.106979 | 0.066321 | 0.089808 |
|  | rs13259667 | 0.839077 | 0.086936 | 0.090378 |
|  | rs2369955 | 1.1429 | 0.093959 | 0.104227 |
|  | rs9556711 | 1.188007 | 0.129358 | 0.11361 |
|  | rs2303317 | 1.087678 | 0.058645 | 0.119054 |
|  | rs4440177 | 1.09074 | 0.062982 | 0.132528 |
|  | rs1876831 | 1.096354 | 0.068103 | 0.13863 |
|  | rs62202398 | 0.842427 | 0.100784 | 0.151786 |
|  | rs2140418 | 0.906825 | 0.064233 | 0.167345 |
|  | rs3764435 | 1.075729 | 0.058087 | 0.176417 |
|  | rs10908907 | 1.085093 | 0.067251 | 0.187612 |
|  | rs7144649 | 0.918383 | 0.059943 | 0.192084 |
|  | rs59972978 | 1.087083 | 0.077601 | 0.242123 |
|  | rs4758317 | 0.937597 | 0.051768 | 0.243206 |
|  | rs12311304 | 0.934006 | 0.055197 | 0.247986 |
|  | rs1000579 | 0.93829 | 0.052379 | 0.253864 |
|  | rs1109501 | 0.931469 | 0.059152 | 0.263604 |
|  | rs12388359 | 1.072645 | 0.069875 | 0.281693 |
|  | rs1229984 | 0.819509 | 0.151605 | 0.281938 |
|  | rs1573496 | 0.908509 | 0.083046 | 0.293863 |
|  | rs4543123 | 0.935268 | 0.060885 | 0.303944 |
|  | rs195204 | 1.065726 | 0.066634 | 0.308635 |
|  | rs642899 | 0.9375 | 0.060019 | 0.313402 |
|  | rs1230165 | 1.070073 | 0.072854 | 0.31985 |
|  | rs9512637 | 0.945057 | 0.054174 | 0.324223 |
|  | rs1318937 | 1.075545 | 0.085363 | 0.358828 |
|  | rs8062326 | 0.860024 | 0.142243 | 0.36191 |
|  | rs3930234 | 0.935414 | 0.071442 | 0.382018 |
|  | rs420817 | 1.046529 | 0.05638 | 0.398561 |
|  | rs1353621 | 1.046939 | 0.058661 | 0.412969 |
|  | rs933769 | 1.057716 | 0.073197 | 0.417464 |
|  | rs13160562 | 0.953193 | 0.056459 | 0.418327 |
|  | rs6716455 | 0.93516 | 0.077633 | 0.419365 |
|  | rs804292 | 0.952772 | 0.057963 | 0.42647 |
|  | rs10253361 | 1.042595 | 0.056377 | 0.440464 |
|  | rs1789891 | 1.055825 | 0.077674 | 0.460264 |
|  | rs2548145 | 1.038463 | 0.056368 | 0.486857 |
|  | rs9656709 | 1.038591 | 0.056753 | 0.488348 |
|  | rs1824024 | 0.96108 | 0.05611 | 0.496535 |
|  | rs16985179 | 1.06479 | 0.098489 | 0.497326 |
|  | rs4761097 | 1.036896 | 0.05584 | 0.501084 |
|  | rs7590720 | 1.041458 | 0.062947 | 0.501527 |
|  | rs11851015 | 1.053743 | 0.082158 | 0.501961 |
|  | rs242938 | 0.930506 | 0.10131 | 0.508264 |
|  | rs2100290 | 0.965543 | 0.052042 | 0.515338 |
|  | rs1497571 | 1.035322 | 0.055448 | 0.516893 |
|  | rs36061340 | 1.072158 | 0.116274 | 0.520573 |
|  | rs1344694 | 1.035878 | 0.060837 | 0.548373 |
|  | rs2810114 | 0.964582 | 0.05876 | 0.553879 |
|  | rs2228093 | 1.048807 | 0.085059 | 0.556816 |
|  | rs10849915 | 0.96755 | 0.055578 | 0.565781 |
|  | rs237238 | 1.061894 | 0.111396 | 0.567003 |
|  | rs36563 | 1.042838 | 0.078544 | 0.577582 |
|  | rs3131513 | 0.970425 | 0.053309 | 0.584727 |
|  | rs8040009 | 1.039096 | 0.073077 | 0.585527 |
|  | rs6943555 | 1.034215 | 0.064309 | 0.588481 |
|  | rs1908556 | 0.958817 | 0.074741 | 0.589542 |
|  | rs12472151 | 0.934712 | 0.124838 | 0.61319 |
|  | rs11724320 | 1.028124 | 0.058619 | 0.626644 |
|  | rs10893366 | 0.966314 | 0.071606 | 0.643776 |
|  | rs4478858 | 1.024619 | 0.056173 | 0.657312 |
|  | rs9871864 | 1.023511 | 0.055636 | 0.669005 |
|  | rs284786 | 0.976008 | 0.057866 | 0.6821 |
|  | rs9825310 | 0.97973 | 0.053628 | 0.708315 |
|  | rs750338 | 1.02397 | 0.067245 | 0.71833 |
|  | rs1800759 | 0.982307 | 0.053737 | 0.744184 |
|  | rs59677118 | 1.02923 | 0.097805 | 0.761745 |
|  | rs2380220 | 0.980529 | 0.074383 | 0.795478 |
|  | rs2154294 | 1.013934 | 0.055324 | 0.7998 |
|  | rs3738443 | 1.017272 | 0.073397 | 0.812388 |
|  | rs1353899 | 1.015416 | 0.068466 | 0.820512 |
|  | rs567926 | 1.012193 | 0.055372 | 0.824675 |
|  | rs768048 | 0.984939 | 0.079956 | 0.851706 |
|  | rs6902771 | 0.993329 | 0.05335 | 0.900827 |
|  | rs886205 | 1.008005 | 0.070615 | 0.90939 |
|  | rs1793257 | 0.987279 | 0.138266 | 0.927161 |
|  | rs279861 | 1.003036 | 0.054806 | 0.955753 |
|  | rs6701037 | 0.998209 | 0.054391 | 0.973754 |
|  | rs2188561 | 1.001567 | 0.065208 | 0.980816 |
| binge eating at age 18 | Offspring PGRS | 1.186213 | 0.330091 | 0.539437 |
|  | rs4440177 | 0.859057 | 0.053661 | 0.015012 |
|  | rs1353621 | 1.142787 | 0.070202 | 0.029802 |
|  | rs3930234 | 0.838825 | 0.069274 | 0.033324 |
|  | rs7144649 | 0.864938 | 0.060563 | 0.038246 |
|  | rs12388359 | 1.15708 | 0.08488 | 0.046713 |
|  | rs3764435 | 1.12098 | 0.065162 | 0.049456 |
|  | rs4543123 | 0.877263 | 0.06193 | 0.063607 |
|  | rs933769 | 0.871131 | 0.065889 | 0.068146 |
|  | rs1230165 | 1.144376 | 0.084818 | 0.068829 |
|  | rs3762894 | 0.869523 | 0.067517 | 0.071772 |
|  | rs1800759 | 1.106083 | 0.065321 | 0.087769 |
|  | rs284786 | 0.900851 | 0.057728 | 0.103224 |
|  | rs2228093 | 0.863959 | 0.078006 | 0.105321 |
|  | rs2548145 | 1.099806 | 0.064688 | 0.105784 |
|  | rs2140418 | 1.127292 | 0.084943 | 0.111803 |
|  | rs10253361 | 1.096731 | 0.06433 | 0.115453 |
|  | rs6902771 | 0.912345 | 0.053208 | 0.115722 |
|  | rs642899 | 0.898379 | 0.061695 | 0.118651 |
|  | rs13259667 | 0.853 | 0.09344 | 0.146654 |
|  | rs237238 | 1.184544 | 0.138693 | 0.148052 |
|  | rs2369955 | 0.879254 | 0.079811 | 0.156295 |
|  | rs1876831 | 0.912802 | 0.061977 | 0.179033 |
|  | rs1864982 | 0.891196 | 0.077626 | 0.186009 |
|  | rs1042026 | 0.92107 | 0.059041 | 0.199611 |
|  | rs3819197 | 0.916911 | 0.062703 | 0.204622 |
|  | rs1000579 | 0.928487 | 0.056137 | 0.219736 |
|  | rs4758317 | 0.934753 | 0.055945 | 0.259586 |
|  | rs8062326 | 0.818856 | 0.146434 | 0.263765 |
|  | rs2827312 | 0.930391 | 0.060111 | 0.264113 |
|  | rs4293630 | 0.910982 | 0.07833 | 0.278233 |
|  | rs1318937 | 0.908227 | 0.081043 | 0.280691 |
|  | rs1353899 | 0.926256 | 0.068803 | 0.30241 |
|  | rs195204 | 0.932244 | 0.065012 | 0.314384 |
|  | rs36061340 | 1.1229 | 0.132497 | 0.325919 |
|  | rs279861 | 1.059305 | 0.062687 | 0.330273 |
|  | rs2188561 | 0.933688 | 0.066558 | 0.335787 |
|  | rs9512637 | 0.942805 | 0.058173 | 0.339817 |
|  | rs1229984 | 0.833432 | 0.159472 | 0.340981 |
|  | rs67031482 | 0.948928 | 0.055404 | 0.369253 |
|  | rs242938 | 0.901196 | 0.105572 | 0.374513 |
|  | rs6716455 | 1.078078 | 0.095053 | 0.39384 |
|  | rs1497571 | 0.953762 | 0.055282 | 0.414063 |
|  | rs1109501 | 0.946252 | 0.064805 | 0.419854 |
|  | rs11724320 | 0.951696 | 0.059135 | 0.425574 |
|  | rs2810114 | 0.94996 | 0.062107 | 0.432335 |
|  | rs1824024 | 0.951723 | 0.060017 | 0.432656 |
|  | rs567926 | 1.046893 | 0.06218 | 0.440377 |
|  | rs36563 | 0.939129 | 0.078397 | 0.451859 |
|  | rs1573496 | 0.931324 | 0.092381 | 0.47321 |
|  | rs768048 | 1.061985 | 0.091531 | 0.485321 |
|  | rs2303317 | 1.040619 | 0.060559 | 0.493856 |
|  | rs1344694 | 0.957544 | 0.06096 | 0.495586 |
|  | rs1908556 | 0.944519 | 0.079609 | 0.498267 |
|  | rs11851015 | 1.057531 | 0.090194 | 0.511912 |
|  | rs420817 | 0.963424 | 0.055882 | 0.520608 |
|  | rs59972978 | 1.049474 | 0.082011 | 0.536617 |
|  | rs9825310 | 0.964312 | 0.057104 | 0.539428 |
|  | rs1380131 | 1.059188 | 0.107234 | 0.570057 |
|  | rs4770403 | 0.957726 | 0.073234 | 0.572161 |
|  | rs62202398 | 0.933151 | 0.115841 | 0.577294 |
|  | rs7590720 | 0.965234 | 0.063658 | 0.59159 |
|  | rs750338 | 0.96292 | 0.068052 | 0.592892 |
|  | rs3131513 | 1.032018 | 0.061521 | 0.59703 |
|  | rs4761097 | 1.030597 | 0.060036 | 0.6049 |
|  | rs13160562 | 1.030972 | 0.065572 | 0.631527 |
|  | rs2100290 | 1.027809 | 0.05963 | 0.636364 |
|  | rs9636231 | 1.028858 | 0.067665 | 0.66532 |
|  | rs4478858 | 0.974584 | 0.058028 | 0.66547 |
|  | rs886205 | 1.032619 | 0.080308 | 0.679807 |
|  | rs16985179 | 1.040913 | 0.105245 | 0.691676 |
|  | rs9656709 | 0.980727 | 0.058494 | 0.744209 |
|  | rs2380220 | 1.027265 | 0.08506 | 0.745283 |
|  | rs6701037 | 1.019174 | 0.059705 | 0.745779 |
|  | rs59677118 | 1.031367 | 0.104212 | 0.759862 |
|  | rs10908907 | 1.020667 | 0.06911 | 0.762563 |
|  | rs9871864 | 0.983593 | 0.057841 | 0.77847 |
|  | rs9556711 | 0.967062 | 0.119172 | 0.785784 |
|  | rs6943555 | 0.982377 | 0.066993 | 0.794302 |
|  | rs1789891 | 0.98358 | 0.078941 | 0.836565 |
|  | rs3738443 | 0.985925 | 0.076435 | 0.854922 |
|  | rs10849915 | 0.989493 | 0.060638 | 0.863148 |
|  | rs1793257 | 0.976933 | 0.152067 | 0.880824 |
|  | rs2154294 | 0.99127 | 0.058813 | 0.882513 |
|  | rs10893366 | 1.007833 | 0.078565 | 0.920269 |
|  | rs7553212 | 1.005129 | 0.062776 | 0.934713 |
|  | rs12472151 | 1.01003 | 0.143707 | 0.944078 |
|  | rs12311304 | 0.996978 | 0.063148 | 0.961886 |
|  | rs804292 | 1.002 | 0.066173 | 0.975868 |
|  | rs8040009 | 1.000675 | 0.075592 | 0.992877 |
| conduct disorder at age 10 | Offspring PGRS | 0.275343 | 0.422309 | 0.400403 |
|  | rs1344694 | 0.361307 | 0.163466 | 0.02444 |
|  | rs195204 | 0.273046 | 0.164084 | 0.030761 |
|  | rs7590720 | 0.367871 | 0.1776 | 0.038322 |
|  | rs750338 | 0.293851 | 0.177007 | 0.042042 |
|  | rs10893366 | 0.269286 | 0.19625 | 0.071822 |
|  | rs279861 | 1.727012 | 0.568069 | 0.096691 |
|  | rs4761097 | 1.720213 | 0.564784 | 0.098497 |
|  | rs3738443 | 1.779315 | 0.663364 | 0.122203 |
|  | rs2827312 | 0.568981 | 0.224307 | 0.152596 |
|  | rs567926 | 1.59438 | 0.521064 | 0.153472 |
|  | rs3762894 | 0.428743 | 0.256943 | 0.157608 |
|  | rs11724320 | 1.579699 | 0.517898 | 0.163118 |
|  | rs1789891 | 0.434061 | 0.262119 | 0.166964 |
|  | rs3930234 | 0.434811 | 0.274218 | 0.186638 |
|  | rs4543123 | 1.542875 | 0.53806 | 0.213693 |
|  | rs9871864 | 1.479431 | 0.483634 | 0.230888 |
|  | rs2380220 | 1.589132 | 0.627304 | 0.240643 |
|  | rs4770403 | 1.528521 | 0.556153 | 0.243558 |
|  | rs9556711 | 1.863064 | 1.000889 | 0.246778 |
|  | rs13259667 | 0.323073 | 0.327912 | 0.265621 |
|  | rs2548145 | 0.691397 | 0.229339 | 0.265896 |
|  | rs2369955 | 1.582389 | 0.680857 | 0.286143 |
|  | rs1353621 | 1.408138 | 0.46429 | 0.299243 |
|  | rs2188561 | 1.436236 | 0.503506 | 0.301759 |
|  | rs6943555 | 1.412328 | 0.493569 | 0.323207 |
|  | rs10253361 | 0.724646 | 0.244325 | 0.339459 |
|  | rs768048 | 1.493531 | 0.639674 | 0.348963 |
|  | rs933769 | 1.418804 | 0.53592 | 0.354392 |
|  | rs242938 | 1.625405 | 0.859243 | 0.358151 |
|  | rs2140418 | 0.661532 | 0.318352 | 0.390551 |
|  | rs2303317 | 1.316407 | 0.427532 | 0.397298 |
|  | rs1353899 | 1.358565 | 0.50667 | 0.411278 |
|  | rs11851015 | 1.412609 | 0.598462 | 0.414858 |
|  | rs1824024 | 0.740665 | 0.273284 | 0.415856 |
|  | rs804292 | 1.320411 | 0.45724 | 0.422182 |
|  | rs1380131 | 1.46024 | 0.702865 | 0.431538 |
|  | rs36563 | 0.66337 | 0.349402 | 0.435848 |
|  | rs9512637 | 1.28664 | 0.424386 | 0.444803 |
|  | rs2228093 | 1.365787 | 0.611882 | 0.486543 |
|  | rs4478858 | 1.222465 | 0.400505 | 0.539801 |
|  | rs7144649 | 0.778409 | 0.32582 | 0.549526 |
|  | rs2154294 | 1.214832 | 0.396677 | 0.551186 |
|  | rs4293630 | 0.7317 | 0.388104 | 0.555899 |
|  | rs1497571 | 1.190168 | 0.386717 | 0.592098 |
|  | rs1864982 | 1.244767 | 0.552195 | 0.621619 |
|  | rs6902771 | 0.852881 | 0.279462 | 0.627208 |
|  | rs9656709 | 0.855354 | 0.282807 | 0.636535 |
|  | rs886205 | 1.205701 | 0.47966 | 0.638207 |
|  | rs6701037 | 0.85809 | 0.283123 | 0.642752 |
|  | rs13160562 | 1.173224 | 0.405973 | 0.644312 |
|  | rs6716455 | 0.785382 | 0.416956 | 0.649072 |
|  | rs9636231 | 1.169313 | 0.4098 | 0.655371 |
|  | rs12388359 | 0.822963 | 0.361727 | 0.657557 |
|  | rs1876831 | 0.839669 | 0.335566 | 0.661922 |
|  | rs36061340 | 1.293059 | 0.789903 | 0.673958 |
|  | rs4440177 | 0.865778 | 0.30568 | 0.683121 |
|  | rs237238 | 0.74283 | 0.544168 | 0.684875 |
|  | rs1573496 | 0.782077 | 0.474801 | 0.685568 |
|  | rs67031482 | 1.137067 | 0.36992 | 0.692962 |
|  | rs1793257 | 0.700802 | 0.707324 | 0.72465 |
|  | rs2100290 | 1.118205 | 0.362416 | 0.730308 |
|  | rs642899 | 0.88419 | 0.350705 | 0.756321 |
|  | rs1000579 | 0.902928 | 0.303256 | 0.761101 |
|  | rs10849915 | 1.103532 | 0.376619 | 0.772839 |
|  | rs3131513 | 0.912671 | 0.305232 | 0.784673 |
|  | rs1042026 | 1.096149 | 0.382867 | 0.792679 |
|  | rs9825310 | 0.923765 | 0.302075 | 0.808395 |
|  | rs16985179 | 1.127693 | 0.601689 | 0.821799 |
|  | rs62202398 | 0.851205 | 0.628897 | 0.82739 |
|  | rs2810114 | 1.068194 | 0.38425 | 0.854492 |
|  | rs1109501 | 1.066909 | 0.394202 | 0.860853 |
|  | rs8040009 | 0.932354 | 0.406754 | 0.872447 |
|  | rs10908907 | 1.052914 | 0.389203 | 0.889064 |
|  | rs7553212 | 1.048341 | 0.356163 | 0.889484 |
|  | rs8062326 | 0.870199 | 0.881197 | 0.890795 |
|  | rs4758317 | 1.044582 | 0.34461 | 0.894817 |
|  | rs12472151 | 1.091953 | 0.800931 | 0.904537 |
|  | rs1908556 | 0.946511 | 0.449794 | 0.907906 |
|  | rs59677118 | 1.064051 | 0.593756 | 0.911412 |
|  | rs420817 | 1.033975 | 0.336204 | 0.918159 |
|  | rs59972978 | 1.040162 | 0.442996 | 0.926335 |
|  | rs3819197 | 0.96874 | 0.368819 | 0.933519 |
|  | rs1800759 | 1.025315 | 0.336916 | 0.939355 |
|  | rs1230165 | 0.969248 | 0.408734 | 0.940957 |
|  | rs3764435 | 1.013 | 0.327565 | 0.968137 |
|  | rs284786 | 1.011869 | 0.357489 | 0.973359 |
|  | rs1318937 | 1.015556 | 0.484651 | 0.974196 |
|  | rs12311304 | 1.003132 | 0.351291 | 0.992875 |
| conduct disorder at age 13 | Offspring PGRS | 3.197754 | 3.593352 | 0.300915 |
|  | rs9871864 | 1.772606 | 0.412683 | 0.013938 |
|  | rs279861 | 0.592191 | 0.143877 | 0.031048 |
|  | rs567926 | 0.605309 | 0.147575 | 0.039482 |
|  | rs1824024 | 1.59289 | 0.366973 | 0.043303 |
|  | rs2228093 | 1.739288 | 0.507377 | 0.057786 |
|  | rs4761097 | 1.437087 | 0.326735 | 0.110733 |
|  | rs9556711 | 1.834656 | 0.704662 | 0.114105 |
|  | rs2188561 | 1.443554 | 0.354772 | 0.135241 |
|  | rs768048 | 0.527344 | 0.226469 | 0.136214 |
|  | rs1344694 | 1.399824 | 0.329382 | 0.152883 |
|  | rs2810114 | 0.670738 | 0.189902 | 0.158361 |
|  | rs11851015 | 1.486994 | 0.433194 | 0.173225 |
|  | rs3738443 | 0.634388 | 0.226065 | 0.201568 |
|  | rs3762894 | 1.398829 | 0.383303 | 0.220624 |
|  | rs16985179 | 0.546513 | 0.275375 | 0.230489 |
|  | rs36563 | 0.642858 | 0.240325 | 0.237254 |
|  | rs7590720 | 1.311726 | 0.316729 | 0.261113 |
|  | rs6902771 | 0.773071 | 0.178117 | 0.263948 |
|  | rs1109501 | 1.307301 | 0.320953 | 0.275066 |
|  | rs1042026 | 1.278883 | 0.305597 | 0.30328 |
|  | rs2154294 | 1.262209 | 0.289011 | 0.309158 |
|  | rs420817 | 0.794005 | 0.182329 | 0.315136 |
|  | rs2369955 | 0.668167 | 0.268483 | 0.315631 |
|  | rs3930234 | 0.712367 | 0.257376 | 0.347866 |
|  | rs8062326 | 0.40114 | 0.404126 | 0.364568 |
|  | rs1380131 | 0.657158 | 0.304993 | 0.365679 |
|  | rs1864982 | 1.310276 | 0.398954 | 0.37479 |
|  | rs12311304 | 0.798755 | 0.205241 | 0.381853 |
|  | rs11724320 | 1.220471 | 0.284872 | 0.393335 |
|  | rs2548145 | 0.82384 | 0.187848 | 0.395408 |
|  | rs6943555 | 0.788126 | 0.222558 | 0.399144 |
|  | rs1573496 | 1.330139 | 0.453527 | 0.402761 |
|  | rs8040009 | 0.764221 | 0.246734 | 0.404916 |
|  | rs1000579 | 0.819607 | 0.195809 | 0.405031 |
|  | rs4543123 | 1.237175 | 0.316424 | 0.40533 |
|  | rs804292 | 0.799812 | 0.216931 | 0.410176 |
|  | rs10893366 | 1.253455 | 0.353932 | 0.423686 |
|  | rs1230165 | 0.781192 | 0.24625 | 0.433413 |
|  | rs1497571 | 0.836696 | 0.190786 | 0.434265 |
|  | rs6701037 | 1.184704 | 0.269773 | 0.456681 |
|  | rs9636231 | 1.190486 | 0.290948 | 0.475571 |
|  | rs4293630 | 1.241666 | 0.379218 | 0.478492 |
|  | rs1793257 | 1.43375 | 0.733981 | 0.481561 |
|  | rs6716455 | 0.769018 | 0.28875 | 0.48425 |
|  | rs4770403 | 0.803608 | 0.251223 | 0.484305 |
|  | rs1229984 | 1.515464 | 0.904087 | 0.485898 |
|  | rs12388359 | 0.806247 | 0.250347 | 0.487942 |
|  | rs2827312 | 0.840597 | 0.215503 | 0.498204 |
|  | rs9825310 | 1.160515 | 0.263579 | 0.512187 |
|  | rs4478858 | 1.161453 | 0.265274 | 0.512267 |
|  | rs9512637 | 1.16389 | 0.270961 | 0.514463 |
|  | rs750338 | 1.179511 | 0.307382 | 0.526384 |
|  | rs886205 | 1.193681 | 0.334213 | 0.527174 |
|  | rs1800759 | 1.149303 | 0.261665 | 0.54106 |
|  | rs10908907 | 1.15447 | 0.29228 | 0.570466 |
|  | rs7553212 | 0.87793 | 0.215482 | 0.595819 |
|  | rs4758317 | 1.104481 | 0.255974 | 0.668078 |
|  | rs7144649 | 0.888046 | 0.249079 | 0.672064 |
|  | rs1876831 | 0.89354 | 0.245738 | 0.682318 |
|  | rs59972978 | 0.880934 | 0.277041 | 0.686867 |
|  | rs13259667 | 0.829515 | 0.385987 | 0.68791 |
|  | rs2380220 | 0.874548 | 0.295103 | 0.691179 |
|  | rs3764435 | 0.917829 | 0.208952 | 0.706447 |
|  | rs62202398 | 0.831032 | 0.434099 | 0.723094 |
|  | rs1908556 | 0.890045 | 0.300821 | 0.730364 |
|  | rs933769 | 0.903521 | 0.273578 | 0.737573 |
|  | rs4440177 | 0.923229 | 0.225006 | 0.743103 |
|  | rs3819197 | 1.087618 | 0.28267 | 0.746571 |
|  | rs642899 | 0.91764 | 0.251056 | 0.753402 |
|  | rs237238 | 0.873801 | 0.409528 | 0.77347 |
|  | rs2303317 | 1.066622 | 0.241703 | 0.775935 |
|  | rs195204 | 0.92799 | 0.249021 | 0.780628 |
|  | rs284786 | 0.93237 | 0.234803 | 0.780965 |
|  | rs1789891 | 0.921161 | 0.290785 | 0.794752 |
|  | rs9656709 | 1.058567 | 0.243802 | 0.80481 |
|  | rs10253361 | 1.056902 | 0.241508 | 0.808631 |
|  | rs1353621 | 0.958175 | 0.228299 | 0.85769 |
|  | rs12472151 | 1.091828 | 0.56624 | 0.865482 |
|  | rs1353899 | 0.958184 | 0.276167 | 0.882182 |
|  | rs2100290 | 0.970613 | 0.220892 | 0.895723 |
|  | rs67031482 | 0.981923 | 0.222828 | 0.935928 |
|  | rs242938 | 0.963735 | 0.444666 | 0.93619 |
|  | rs3131513 | 1.014576 | 0.234432 | 0.950063 |
|  | rs2140418 | 1.015238 | 0.293327 | 0.958254 |
|  | rs10849915 | 1.006446 | 0.244713 | 0.978917 |
|  | rs1318937 | 1.00487 | 0.339945 | 0.988543 |
|  | rs36061340 | 1.006336 | 0.471309 | 0.98924 |
|  | rs13160562 | 1.003287 | 0.24902 | 0.989451 |
|  | rs59677118 | 0.997084 | 0.397468 | 0.994155 |
| conduct disorder at age 15 | Offspring PGRS | 0.880541 | 0.819311 | 0.891247 |
|  | rs10253361 | 0.588375 | 0.123202 | 0.011309 |
|  | rs10849915 | 0.628314 | 0.14344 | 0.041791 |
|  | rs1864982 | 1.600996 | 0.390967 | 0.053955 |
|  | rs1824024 | 1.453156 | 0.287065 | 0.058504 |
|  | rs9636231 | 1.466322 | 0.298095 | 0.059731 |
|  | rs6902771 | 0.686287 | 0.138424 | 0.06198 |
|  | rs4761097 | 1.407385 | 0.275226 | 0.080556 |
|  | rs12472151 | 0.186361 | 0.187914 | 0.095677 |
|  | rs4758317 | 0.727916 | 0.148718 | 0.120094 |
|  | rs7553212 | 0.712523 | 0.156568 | 0.122956 |
|  | rs420817 | 0.741248 | 0.147445 | 0.132256 |
|  | rs3131513 | 0.732084 | 0.153472 | 0.136853 |
|  | rs1353621 | 1.305956 | 0.257732 | 0.176186 |
|  | rs6716455 | 0.643306 | 0.226188 | 0.209609 |
|  | rs642899 | 0.732641 | 0.185335 | 0.218774 |
|  | rs2303317 | 1.271069 | 0.248393 | 0.219673 |
|  | rs1573496 | 0.628009 | 0.247715 | 0.238247 |
|  | rs67031482 | 0.800197 | 0.157382 | 0.257088 |
|  | rs6943555 | 1.273047 | 0.273733 | 0.261548 |
|  | rs2188561 | 1.266276 | 0.277895 | 0.282044 |
|  | rs13160562 | 0.785614 | 0.177484 | 0.285499 |
|  | rs59677118 | 1.37769 | 0.417805 | 0.290726 |
|  | rs1229984 | 0.37078 | 0.373812 | 0.325066 |
|  | rs1789891 | 0.763226 | 0.221022 | 0.350795 |
|  | rs2827312 | 0.813119 | 0.180404 | 0.351108 |
|  | rs16985179 | 0.702612 | 0.273894 | 0.365246 |
|  | rs2810114 | 0.815612 | 0.188272 | 0.377262 |
|  | rs886205 | 1.233719 | 0.297459 | 0.383689 |
|  | rs279861 | 1.179587 | 0.228094 | 0.393023 |
|  | rs9871864 | 1.174903 | 0.229577 | 0.40943 |
|  | rs4770403 | 1.210398 | 0.283983 | 0.415721 |
|  | rs768048 | 0.769978 | 0.25027 | 0.421281 |
|  | rs9512637 | 1.174974 | 0.235609 | 0.421324 |
|  | rs1800759 | 1.162538 | 0.225578 | 0.437653 |
|  | rs9656709 | 0.85762 | 0.170415 | 0.439542 |
|  | rs284786 | 0.848141 | 0.185808 | 0.452153 |
|  | rs11851015 | 0.793818 | 0.248559 | 0.460864 |
|  | rs36061340 | 0.720989 | 0.334658 | 0.480951 |
|  | rs13259667 | 0.746796 | 0.313371 | 0.486567 |
|  | rs4440177 | 1.139115 | 0.231212 | 0.521061 |
|  | rs3819197 | 0.86832 | 0.205993 | 0.551725 |
|  | rs9556711 | 1.244174 | 0.465549 | 0.559313 |
|  | rs2100290 | 1.121015 | 0.220059 | 0.560613 |
|  | rs7144649 | 1.135579 | 0.256352 | 0.573291 |
|  | rs237238 | 1.218689 | 0.432035 | 0.576921 |
|  | rs59972978 | 1.140774 | 0.289825 | 0.604172 |
|  | rs10908907 | 0.887574 | 0.204248 | 0.604273 |
|  | rs36563 | 0.867261 | 0.249518 | 0.620602 |
|  | rs12311304 | 1.108417 | 0.231201 | 0.621676 |
|  | rs2380220 | 1.134973 | 0.299449 | 0.631318 |
|  | rs1109501 | 0.897548 | 0.205623 | 0.637064 |
|  | rs1344694 | 0.906417 | 0.194831 | 0.647588 |
|  | rs7590720 | 1.102482 | 0.236175 | 0.648794 |
|  | rs10893366 | 0.883357 | 0.24152 | 0.650101 |
|  | rs933769 | 1.116546 | 0.271479 | 0.650261 |
|  | rs1793257 | 0.768525 | 0.451131 | 0.653782 |
|  | rs1497571 | 1.083655 | 0.210817 | 0.679629 |
|  | rs11724320 | 0.916975 | 0.193235 | 0.680847 |
|  | rs1380131 | 1.132277 | 0.363889 | 0.699085 |
|  | rs3764435 | 0.931963 | 0.181424 | 0.717383 |
|  | rs3930234 | 0.903598 | 0.255745 | 0.72022 |
|  | rs2154294 | 0.933313 | 0.184152 | 0.726506 |
|  | rs4478858 | 1.071186 | 0.213065 | 0.729551 |
|  | rs1353899 | 0.924207 | 0.228534 | 0.749916 |
|  | rs4543123 | 1.070419 | 0.243315 | 0.764654 |
|  | rs1042026 | 0.939666 | 0.20441 | 0.774823 |
|  | rs9825310 | 1.057457 | 0.206813 | 0.775145 |
|  | rs3762894 | 0.927541 | 0.244849 | 0.775687 |
|  | rs8040009 | 1.072028 | 0.269872 | 0.782329 |
|  | rs1908556 | 0.928631 | 0.266346 | 0.796285 |
|  | rs3738443 | 0.934247 | 0.250208 | 0.799528 |
|  | rs12388359 | 0.947925 | 0.22302 | 0.820183 |
|  | rs62202398 | 1.086323 | 0.43488 | 0.836143 |
|  | rs750338 | 1.038608 | 0.242135 | 0.87092 |
|  | rs567926 | 1.028964 | 0.200643 | 0.883583 |
|  | rs2548145 | 1.028779 | 0.200884 | 0.884471 |
|  | rs8062326 | 0.927867 | 0.543356 | 0.89827 |
|  | rs2228093 | 0.9638 | 0.290228 | 0.902548 |
|  | rs1876831 | 0.975285 | 0.224808 | 0.913543 |
|  | rs2369955 | 0.968006 | 0.294425 | 0.914862 |
|  | rs1318937 | 1.029326 | 0.298839 | 0.920695 |
|  | rs4293630 | 1.027462 | 0.290099 | 0.92356 |
|  | rs804292 | 0.979925 | 0.218929 | 0.927676 |
|  | rs1000579 | 0.991279 | 0.200577 | 0.96547 |
|  | rs2140418 | 0.992014 | 0.249062 | 0.974524 |
|  | rs242938 | 1.012113 | 0.396988 | 0.975512 |
|  | rs1230165 | 1.00386 | 0.251278 | 0.987721 |
|  | rs6701037 | 0.997079 | 0.195321 | 0.988087 |
|  | rs195204 | 0.997083 | 0.226301 | 0.989729 |
| conduct disorder at age 7 | Offspring PGRS | 0.055929 | 0.084226 | 0.055511 |
|  | rs2228093 | 2.38334 | 0.909066 | 0.022787 |
|  | rs9556711 | 2.770084 | 1.259477 | 0.025032 |
|  | rs36061340 | 2.773083 | 1.273724 | 0.026378 |
|  | rs1229984 | 3.100366 | 1.900589 | 0.06492 |
|  | rs567926 | 1.766354 | 0.567289 | 0.07649 |
|  | rs4543123 | 1.782208 | 0.590067 | 0.08093 |
|  | rs1824024 | 1.710702 | 0.547264 | 0.093286 |
|  | rs279861 | 1.702208 | 0.545332 | 0.096842 |
|  | rs10253361 | 1.665436 | 0.533121 | 0.111053 |
|  | rs1800759 | 0.592991 | 0.209047 | 0.138245 |
|  | rs2827312 | 1.647102 | 0.559171 | 0.141586 |
|  | rs6716455 | 0.344699 | 0.250831 | 0.143284 |
|  | rs1230165 | 0.467482 | 0.248261 | 0.152189 |
|  | rs6902771 | 0.630148 | 0.20833 | 0.162462 |
|  | rs1876831 | 1.555222 | 0.52721 | 0.192666 |
|  | rs933769 | 0.519614 | 0.272195 | 0.211392 |
|  | rs2154294 | 1.474107 | 0.472964 | 0.226487 |
|  | rs1042026 | 1.456164 | 0.476538 | 0.250822 |
|  | rs13160562 | 0.654279 | 0.249389 | 0.265726 |
|  | rs750338 | 0.610676 | 0.270736 | 0.265947 |
|  | rs9871864 | 0.700809 | 0.229645 | 0.277946 |
|  | rs768048 | 0.519484 | 0.314948 | 0.280034 |
|  | rs9656709 | 0.711011 | 0.231034 | 0.293882 |
|  | rs3131513 | 1.376314 | 0.432695 | 0.309642 |
|  | rs2140418 | 1.444268 | 0.531419 | 0.317769 |
|  | rs2100290 | 1.374691 | 0.438708 | 0.318682 |
|  | rs7590720 | 0.699333 | 0.266752 | 0.34846 |
|  | rs62202398 | 0.389073 | 0.399032 | 0.357349 |
|  | rs36563 | 1.411499 | 0.557564 | 0.382935 |
|  | rs1497571 | 1.308065 | 0.416238 | 0.398704 |
|  | rs6701037 | 1.298238 | 0.410326 | 0.408913 |
|  | rs1318937 | 1.401241 | 0.578956 | 0.414212 |
|  | rs9512637 | 1.293909 | 0.415472 | 0.422288 |
|  | rs7144649 | 1.325069 | 0.469814 | 0.427285 |
|  | rs8040009 | 0.696066 | 0.322298 | 0.433931 |
|  | rs2188561 | 1.313564 | 0.459147 | 0.435222 |
|  | rs11724320 | 1.284707 | 0.416617 | 0.439787 |
|  | rs2548145 | 0.788783 | 0.251877 | 0.457468 |
|  | rs8062326 | 1.710699 | 1.238702 | 0.4584 |
|  | rs6943555 | 0.755713 | 0.299119 | 0.479165 |
|  | rs16985179 | 0.626201 | 0.418742 | 0.483935 |
|  | rs1908556 | 0.69336 | 0.362866 | 0.484089 |
|  | rs10849915 | 0.784103 | 0.277985 | 0.492696 |
|  | rs2810114 | 1.255321 | 0.427051 | 0.503867 |
|  | rs4478858 | 0.802849 | 0.264221 | 0.504623 |
|  | rs3930234 | 1.313747 | 0.541021 | 0.507563 |
|  | rs12472151 | 0.511701 | 0.520388 | 0.510005 |
|  | rs4770403 | 1.273294 | 0.48127 | 0.522681 |
|  | rs13259667 | 0.636298 | 0.463539 | 0.534877 |
|  | rs9825310 | 0.842894 | 0.27152 | 0.595712 |
|  | rs2303317 | 1.179836 | 0.37277 | 0.600682 |
|  | rs284786 | 1.192424 | 0.401015 | 0.600763 |
|  | rs886205 | 0.806812 | 0.358025 | 0.628564 |
|  | rs2369955 | 1.223762 | 0.552644 | 0.654768 |
|  | rs1380131 | 0.764711 | 0.460327 | 0.655859 |
|  | rs10908907 | 0.851418 | 0.323343 | 0.671893 |
|  | rs1000579 | 1.145451 | 0.367427 | 0.672039 |
|  | rs67031482 | 1.140769 | 0.35959 | 0.676082 |
|  | rs1793257 | 1.337842 | 0.97176 | 0.688637 |
|  | rs242938 | 0.759264 | 0.547952 | 0.702748 |
|  | rs4440177 | 1.126321 | 0.368392 | 0.716083 |
|  | rs1109501 | 0.873524 | 0.330391 | 0.720711 |
|  | rs4758317 | 0.899731 | 0.291957 | 0.744716 |
|  | rs10893366 | 0.866911 | 0.387508 | 0.749343 |
|  | rs3738443 | 0.873638 | 0.390249 | 0.762333 |
|  | rs642899 | 1.11314 | 0.407257 | 0.769549 |
|  | rs1864982 | 1.134351 | 0.501445 | 0.775513 |
|  | rs3762894 | 0.886763 | 0.391029 | 0.78521 |
|  | rs4293630 | 1.117033 | 0.499088 | 0.804359 |
|  | rs12311304 | 0.922059 | 0.320317 | 0.815308 |
|  | rs1789891 | 0.900966 | 0.404392 | 0.816268 |
|  | rs11851015 | 1.107587 | 0.495605 | 0.819364 |
|  | rs12388359 | 1.085789 | 0.392825 | 0.820034 |
|  | rs3819197 | 0.926196 | 0.351392 | 0.83985 |
|  | rs9636231 | 0.943193 | 0.336697 | 0.869863 |
|  | rs1344694 | 0.954537 | 0.327711 | 0.892195 |
|  | rs804292 | 0.952593 | 0.345731 | 0.893545 |
|  | rs195204 | 1.047185 | 0.381847 | 0.899383 |
|  | rs2380220 | 1.051642 | 0.463555 | 0.909054 |
|  | rs7553212 | 0.963812 | 0.323668 | 0.9126 |
|  | rs420817 | 1.035412 | 0.328876 | 0.912757 |
|  | rs4761097 | 1.024096 | 0.325553 | 0.940295 |
|  | rs3764435 | 1.020744 | 0.32156 | 0.948035 |
|  | rs1353621 | 1.018113 | 0.33564 | 0.956575 |
|  | rs59972978 | 0.97927 | 0.41409 | 0.960489 |
|  | rs59677118 | 0.985479 | 0.552867 | 0.979198 |
|  | rs1353899 | 0.997298 | 0.398943 | 0.994604 |
|  | rs1573496 | 1.003245 | 0.533924 | 0.995142 |
| Depressive symptoms at age 10 | Offspring PGRS | 0.617322 | 0.680836 | 0.661846 |
|  | rs1789891 | 2.067264 | 0.543032 | 0.005698 |
|  | rs2380220 | 1.924712 | 0.5175 | 0.01488 |
|  | rs768048 | 1.998772 | 0.568679 | 0.014929 |
|  | rs12472151 | 2.42133 | 0.924742 | 0.020587 |
|  | rs2548145 | 1.640272 | 0.390492 | 0.037647 |
|  | rs2188561 | 1.589934 | 0.392626 | 0.06042 |
|  | rs8062326 | 2.32272 | 1.08041 | 0.070023 |
|  | rs9512637 | 1.510463 | 0.355255 | 0.079517 |
|  | rs9871864 | 1.487044 | 0.349361 | 0.091234 |
|  | rs7590720 | 0.6185 | 0.180647 | 0.099971 |
|  | rs10253361 | 1.466862 | 0.344073 | 0.102395 |
|  | rs59972978 | 1.560838 | 0.430154 | 0.106198 |
|  | rs2140418 | 1.522318 | 0.407489 | 0.116431 |
|  | rs1864982 | 0.482983 | 0.223936 | 0.116495 |
|  | rs7144649 | 0.59834 | 0.196056 | 0.117013 |
|  | rs1380131 | 0.40388 | 0.238548 | 0.124782 |
|  | rs195204 | 0.619441 | 0.195039 | 0.128236 |
|  | rs4770403 | 1.48353 | 0.391256 | 0.134773 |
|  | rs3764435 | 0.729892 | 0.17172 | 0.1808 |
|  | rs2369955 | 1.481621 | 0.465768 | 0.211088 |
|  | rs9556711 | 1.655046 | 0.671179 | 0.214096 |
|  | rs11724320 | 0.724833 | 0.190824 | 0.221559 |
|  | rs62202398 | 0.414536 | 0.301584 | 0.226123 |
|  | rs1109501 | 0.700724 | 0.208496 | 0.231987 |
|  | rs1344694 | 0.731958 | 0.196087 | 0.244116 |
|  | rs2303317 | 0.770437 | 0.181338 | 0.267847 |
|  | rs2154294 | 1.286871 | 0.301853 | 0.282265 |
|  | rs67031482 | 0.776893 | 0.182722 | 0.283104 |
|  | rs886205 | 1.341653 | 0.372494 | 0.28979 |
|  | rs13160562 | 1.293966 | 0.316546 | 0.292128 |
|  | rs36563 | 0.675564 | 0.252346 | 0.293722 |
|  | rs1876831 | 0.73568 | 0.219486 | 0.303538 |
|  | rs1800759 | 1.260065 | 0.292028 | 0.31855 |
|  | rs8040009 | 1.32859 | 0.380344 | 0.320973 |
|  | rs2100290 | 1.254533 | 0.293542 | 0.332477 |
|  | rs4293630 | 1.337939 | 0.412156 | 0.344626 |
|  | rs6902771 | 0.800212 | 0.189383 | 0.346326 |
|  | rs1573496 | 0.646536 | 0.303399 | 0.352695 |
|  | rs2810114 | 0.772795 | 0.215128 | 0.354512 |
|  | rs10908907 | 1.24825 | 0.319214 | 0.385887 |
|  | rs9825310 | 0.819568 | 0.194122 | 0.40087 |
|  | rs12388359 | 0.759743 | 0.252767 | 0.408865 |
|  | rs4543123 | 1.22962 | 0.321556 | 0.429274 |
|  | rs9636231 | 1.210912 | 0.302747 | 0.444006 |
|  | rs3131513 | 1.195158 | 0.279414 | 0.445723 |
|  | rs3762894 | 0.773461 | 0.261908 | 0.448084 |
|  | rs1042026 | 0.820598 | 0.219204 | 0.459191 |
|  | rs1000579 | 1.184184 | 0.278181 | 0.471745 |
|  | rs16985179 | 1.292016 | 0.470462 | 0.481679 |
|  | rs1229984 | 0.49902 | 0.504831 | 0.492015 |
|  | rs36061340 | 1.340783 | 0.580797 | 0.498417 |
|  | rs10849915 | 1.17595 | 0.285614 | 0.504571 |
|  | rs2228093 | 1.226008 | 0.405523 | 0.537873 |
|  | rs1908556 | 1.207561 | 0.377906 | 0.546733 |
|  | rs1230165 | 1.183348 | 0.337841 | 0.555414 |
|  | rs11851015 | 1.205087 | 0.385224 | 0.559499 |
|  | rs6716455 | 0.808758 | 0.304621 | 0.573073 |
|  | rs804292 | 0.858022 | 0.235636 | 0.577133 |
|  | rs1353621 | 0.875118 | 0.2175 | 0.591457 |
|  | rs237238 | 0.762962 | 0.396225 | 0.602395 |
|  | rs642899 | 1.146862 | 0.304688 | 0.606004 |
|  | rs1497571 | 1.125128 | 0.261973 | 0.612612 |
|  | rs3930234 | 0.840159 | 0.294493 | 0.619278 |
|  | rs2827312 | 1.133266 | 0.288458 | 0.623076 |
|  | rs1318937 | 1.159941 | 0.377188 | 0.648196 |
|  | rs242938 | 0.794316 | 0.40895 | 0.654682 |
|  | rs750338 | 0.878106 | 0.255269 | 0.654769 |
|  | rs59677118 | 0.828741 | 0.368321 | 0.672538 |
|  | rs7553212 | 0.911854 | 0.228209 | 0.712349 |
|  | rs284786 | 1.093284 | 0.273766 | 0.721717 |
|  | rs4478858 | 0.92386 | 0.220385 | 0.7399 |
|  | rs13259667 | 0.874247 | 0.407473 | 0.773084 |
|  | rs9656709 | 1.070325 | 0.253023 | 0.773735 |
|  | rs1824024 | 0.937925 | 0.236548 | 0.799418 |
|  | rs3819197 | 0.932479 | 0.257761 | 0.800344 |
|  | rs6701037 | 1.060105 | 0.248148 | 0.803087 |
|  | rs4761097 | 0.951471 | 0.223443 | 0.832238 |
|  | rs4440177 | 0.95182 | 0.236021 | 0.842157 |
|  | rs6943555 | 1.052189 | 0.281981 | 0.849446 |
|  | rs3738443 | 0.949709 | 0.302886 | 0.87147 |
|  | rs279861 | 0.964848 | 0.226868 | 0.879036 |
|  | rs10893366 | 1.04789 | 0.323849 | 0.879688 |
|  | rs420817 | 1.035574 | 0.242034 | 0.88111 |
|  | rs4758317 | 1.021961 | 0.242151 | 0.926952 |
|  | rs12311304 | 0.977188 | 0.246922 | 0.927234 |
|  | rs933769 | 0.974381 | 0.295247 | 0.931744 |
|  | rs567926 | 0.983383 | 0.231521 | 0.943258 |
|  | rs1353899 | 1.010541 | 0.293822 | 0.971231 |
| Depressive symptoms at age 13 | Offspring PGRS | 4.746878 | 6.271271 | 0.238438 |
|  | rs1230165 | 2.156549 | 0.626606 | 0.008171 |
|  | rs1864982 | 2.072679 | 0.663358 | 0.022769 |
|  | rs1793257 | 2.718323 | 1.267184 | 0.031937 |
|  | rs2810114 | 0.520683 | 0.191505 | 0.075998 |
|  | rs8040009 | 1.72714 | 0.547326 | 0.084631 |
|  | rs11724320 | 1.556312 | 0.42697 | 0.106906 |
|  | rs6902771 | 0.637145 | 0.180873 | 0.112321 |
|  | rs1824024 | 1.531015 | 0.423864 | 0.123931 |
|  | rs1573496 | 0.342827 | 0.248843 | 0.140253 |
|  | rs3930234 | 0.480759 | 0.242121 | 0.145879 |
|  | rs9825310 | 0.679835 | 0.191911 | 0.17161 |
|  | rs2188561 | 1.485286 | 0.43473 | 0.176497 |
|  | rs4761097 | 1.431832 | 0.390928 | 0.188602 |
|  | rs1000579 | 0.708077 | 0.208313 | 0.240645 |
|  | rs1353621 | 1.379496 | 0.38103 | 0.244117 |
|  | rs6943555 | 1.406724 | 0.415225 | 0.247618 |
|  | rs1789891 | 1.448097 | 0.477077 | 0.261081 |
|  | rs2303317 | 0.733266 | 0.202965 | 0.262351 |
|  | rs4478858 | 1.356828 | 0.370603 | 0.263911 |
|  | rs13259667 | 0.465468 | 0.336179 | 0.289688 |
|  | rs4770403 | 1.388134 | 0.439648 | 0.300438 |
|  | rs279861 | 0.74791 | 0.210017 | 0.300937 |
|  | rs2228093 | 1.455089 | 0.533471 | 0.306294 |
|  | rs567926 | 0.755512 | 0.213156 | 0.320365 |
|  | rs1353899 | 0.686837 | 0.266313 | 0.332623 |
|  | rs59972978 | 1.358776 | 0.452578 | 0.357334 |
|  | rs7553212 | 0.761992 | 0.231764 | 0.371491 |
|  | rs36061340 | 1.521665 | 0.725656 | 0.378691 |
|  | rs1344694 | 1.283456 | 0.365287 | 0.38058 |
|  | rs59677118 | 0.592659 | 0.354778 | 0.382172 |
|  | rs2548145 | 0.791584 | 0.217272 | 0.394488 |
|  | rs10893366 | 0.713862 | 0.29041 | 0.40736 |
|  | rs1800759 | 1.249745 | 0.339348 | 0.411625 |
|  | rs2154294 | 1.252292 | 0.344263 | 0.413146 |
|  | rs284786 | 0.773539 | 0.243796 | 0.415225 |
|  | rs1497571 | 0.799903 | 0.219447 | 0.415748 |
|  | rs36563 | 0.703287 | 0.304442 | 0.416145 |
|  | rs2369955 | 0.687981 | 0.327925 | 0.432669 |
|  | rs2140418 | 0.742417 | 0.285244 | 0.438214 |
|  | rs242938 | 1.430928 | 0.668175 | 0.442864 |
|  | rs1318937 | 0.700536 | 0.328061 | 0.447252 |
|  | rs9656709 | 1.233824 | 0.34224 | 0.448746 |
|  | rs9556711 | 0.586191 | 0.426082 | 0.462454 |
|  | rs4440177 | 0.803175 | 0.242174 | 0.467272 |
|  | rs13160562 | 1.230621 | 0.354637 | 0.471458 |
|  | rs10908907 | 1.237777 | 0.37012 | 0.475607 |
|  | rs420817 | 0.823589 | 0.226595 | 0.480549 |
|  | rs768048 | 1.298142 | 0.483073 | 0.48318 |
|  | rs9512637 | 1.213094 | 0.337953 | 0.488055 |
|  | rs3819197 | 0.813321 | 0.27539 | 0.541698 |
|  | rs12311304 | 1.182934 | 0.339112 | 0.557854 |
|  | rs886205 | 0.800968 | 0.306163 | 0.561501 |
|  | rs7144649 | 1.196593 | 0.371305 | 0.562995 |
|  | rs11851015 | 1.236525 | 0.456373 | 0.565134 |
|  | rs195204 | 1.189576 | 0.360967 | 0.56726 |
|  | rs12388359 | 1.184864 | 0.351997 | 0.568007 |
|  | rs4293630 | 1.232245 | 0.451282 | 0.568514 |
|  | rs1042026 | 0.850091 | 0.264503 | 0.601686 |
|  | rs10253361 | 1.146858 | 0.313747 | 0.616454 |
|  | rs2380220 | 1.192077 | 0.431947 | 0.627758 |
|  | rs237238 | 0.753635 | 0.452458 | 0.637553 |
|  | rs4543123 | 0.857777 | 0.289829 | 0.649804 |
|  | rs62202398 | 1.260531 | 0.66894 | 0.662623 |
|  | rs7590720 | 1.136561 | 0.337083 | 0.666026 |
|  | rs3738443 | 0.848529 | 0.326136 | 0.669129 |
|  | rs1109501 | 1.136829 | 0.344971 | 0.672576 |
|  | rs67031482 | 0.893371 | 0.243744 | 0.679412 |
|  | rs1229984 | 0.696754 | 0.707182 | 0.721844 |
|  | rs6701037 | 1.097159 | 0.299858 | 0.734406 |
|  | rs3762894 | 0.882734 | 0.337176 | 0.744009 |
|  | rs12472151 | 1.198259 | 0.71834 | 0.762875 |
|  | rs804292 | 0.912451 | 0.286924 | 0.770773 |
|  | rs933769 | 0.907616 | 0.330275 | 0.789947 |
|  | rs1908556 | 0.904795 | 0.364962 | 0.80411 |
|  | rs8062326 | 1.195633 | 0.8622 | 0.804309 |
|  | rs10849915 | 0.930962 | 0.275253 | 0.808818 |
|  | rs750338 | 1.075196 | 0.343373 | 0.820403 |
|  | rs3764435 | 0.942905 | 0.257167 | 0.829335 |
|  | rs3131513 | 1.055727 | 0.291467 | 0.844276 |
|  | rs2100290 | 0.948814 | 0.25924 | 0.847503 |
|  | rs9636231 | 1.058166 | 0.317583 | 0.85058 |
|  | rs16985179 | 0.914385 | 0.445819 | 0.854348 |
|  | rs642899 | 1.052737 | 0.333686 | 0.871196 |
|  | rs2827312 | 1.037209 | 0.3105 | 0.902868 |
|  | rs9871864 | 1.031577 | 0.283448 | 0.909917 |
|  | rs4758317 | 1.01597 | 0.283123 | 0.95466 |
|  | rs1380131 | 0.983769 | 0.463546 | 0.972296 |
|  | rs1876831 | 1.010625 | 0.322078 | 0.973543 |
|  | rs6716455 | 1.0032 | 0.408799 | 0.993745 |
| Depressive symptoms at age 15 | Offspring PGRS | 1.088355 | 0.901054 | 0.918544 |
|  | rs3930234 | 0.493366 | 0.1556 | 0.025082 |
|  | rs750338 | 1.500614 | 0.281735 | 0.030632 |
|  | rs10253361 | 1.432313 | 0.249096 | 0.038834 |
|  | rs9825310 | 1.401157 | 0.242436 | 0.051246 |
|  | rs284786 | 1.406742 | 0.253187 | 0.057937 |
|  | rs10893366 | 1.447522 | 0.29845 | 0.072839 |
|  | rs12472151 | 0.292773 | 0.209721 | 0.086382 |
|  | rs1793257 | 0.189861 | 0.190864 | 0.098386 |
|  | rs13259667 | 0.470268 | 0.214844 | 0.098656 |
|  | rs8040009 | 0.664103 | 0.169564 | 0.10891 |
|  | rs62202398 | 0.450771 | 0.232587 | 0.122528 |
|  | rs10908907 | 0.717379 | 0.154331 | 0.122602 |
|  | rs237238 | 0.494139 | 0.227434 | 0.125622 |
|  | rs3738443 | 1.366852 | 0.292123 | 0.143675 |
|  | rs11724320 | 1.292387 | 0.231368 | 0.151938 |
|  | rs242938 | 1.521291 | 0.448993 | 0.155154 |
|  | rs36563 | 0.686634 | 0.189192 | 0.172426 |
|  | rs1573496 | 0.624548 | 0.218207 | 0.177881 |
|  | rs886205 | 1.312245 | 0.273562 | 0.192403 |
|  | rs1908556 | 0.698561 | 0.197836 | 0.205266 |
|  | rs1824024 | 1.2361 | 0.220139 | 0.233976 |
|  | rs1353621 | 1.228441 | 0.216297 | 0.242598 |
|  | rs1876831 | 0.783106 | 0.169621 | 0.259003 |
|  | rs6943555 | 1.241089 | 0.237883 | 0.259799 |
|  | rs16985179 | 1.335535 | 0.358495 | 0.281089 |
|  | rs1800759 | 1.200291 | 0.206265 | 0.288066 |
|  | rs1789891 | 0.770782 | 0.196717 | 0.307676 |
|  | rs8062326 | 1.52008 | 0.637291 | 0.317871 |
|  | rs12311304 | 0.829973 | 0.159918 | 0.333437 |
|  | rs2548145 | 1.180827 | 0.20439 | 0.336915 |
|  | rs36061340 | 0.669594 | 0.282831 | 0.342338 |
|  | rs7590720 | 0.828023 | 0.165654 | 0.345533 |
|  | rs567926 | 0.852723 | 0.149761 | 0.364323 |
|  | rs1353899 | 0.827131 | 0.188698 | 0.405451 |
|  | rs10849915 | 0.859195 | 0.162357 | 0.421912 |
|  | rs1497571 | 1.145465 | 0.197099 | 0.429946 |
|  | rs768048 | 0.803442 | 0.223979 | 0.432429 |
|  | rs4293630 | 0.81738 | 0.220082 | 0.453901 |
|  | rs59972978 | 1.17816 | 0.26167 | 0.460394 |
|  | rs4770403 | 0.84784 | 0.196939 | 0.477322 |
|  | rs1344694 | 0.874153 | 0.166809 | 0.480909 |
|  | rs9636231 | 1.140309 | 0.212602 | 0.481288 |
|  | rs2100290 | 0.889003 | 0.153609 | 0.495921 |
|  | rs9656709 | 0.888329 | 0.155941 | 0.499964 |
|  | rs279861 | 0.889378 | 0.154575 | 0.49998 |
|  | rs3131513 | 1.122104 | 0.195894 | 0.509312 |
|  | rs7553212 | 1.124328 | 0.200794 | 0.511714 |
|  | rs804292 | 1.129117 | 0.213489 | 0.520703 |
|  | rs642899 | 0.873183 | 0.185391 | 0.523008 |
|  | rs7144649 | 0.87245 | 0.187809 | 0.52617 |
|  | rs1318937 | 0.839337 | 0.232647 | 0.527469 |
|  | rs4761097 | 0.898254 | 0.15686 | 0.538909 |
|  | rs9871864 | 1.104858 | 0.190015 | 0.562043 |
|  | rs13160562 | 1.108367 | 0.206549 | 0.580875 |
|  | rs6716455 | 0.860939 | 0.238281 | 0.58851 |
|  | rs1109501 | 1.108809 | 0.213571 | 0.591796 |
|  | rs12388359 | 0.889269 | 0.195469 | 0.593411 |
|  | rs4758317 | 0.911258 | 0.160722 | 0.598274 |
|  | rs9556711 | 0.815383 | 0.319692 | 0.602677 |
|  | rs4478858 | 0.914629 | 0.162734 | 0.615989 |
|  | rs2228093 | 1.131665 | 0.286839 | 0.625554 |
|  | rs1864982 | 1.123797 | 0.273854 | 0.631975 |
|  | rs1380131 | 0.861959 | 0.273456 | 0.639617 |
|  | rs67031482 | 0.924279 | 0.159349 | 0.647867 |
|  | rs2827312 | 0.92598 | 0.178638 | 0.690168 |
|  | rs2154294 | 0.935395 | 0.16274 | 0.70107 |
|  | rs1042026 | 0.930213 | 0.179312 | 0.707448 |
|  | rs2380220 | 1.081742 | 0.256448 | 0.740318 |
|  | rs1230165 | 1.072521 | 0.233279 | 0.747538 |
|  | rs1000579 | 1.057555 | 0.187261 | 0.75198 |
|  | rs59677118 | 0.906184 | 0.288216 | 0.756761 |
|  | rs4440177 | 0.954089 | 0.176865 | 0.799857 |
|  | rs3819197 | 1.050708 | 0.209557 | 0.804127 |
|  | rs3762894 | 1.055753 | 0.23712 | 0.80912 |
|  | rs2140418 | 1.05309 | 0.230026 | 0.812797 |
|  | rs2188561 | 1.047161 | 0.213504 | 0.821187 |
|  | rs3764435 | 0.964075 | 0.164867 | 0.830594 |
|  | rs6701037 | 1.03658 | 0.179536 | 0.835675 |
|  | rs1229984 | 0.88644 | 0.522614 | 0.837994 |
|  | rs11851015 | 0.96013 | 0.248459 | 0.875067 |
|  | rs9512637 | 1.028399 | 0.18531 | 0.876501 |
|  | rs2810114 | 0.971279 | 0.189723 | 0.881404 |
|  | rs933769 | 0.969148 | 0.21895 | 0.889678 |
|  | rs6902771 | 1.022947 | 0.176142 | 0.895176 |
|  | rs4543123 | 0.978644 | 0.20196 | 0.916687 |
|  | rs195204 | 0.985424 | 0.196909 | 0.941421 |
|  | rs420817 | 0.988912 | 0.170978 | 0.948581 |
|  | rs2369955 | 0.986101 | 0.261996 | 0.957987 |
|  | rs2303317 | 1.001784 | 0.171484 | 0.991693 |
| Depressive symptoms at age 7 | Offspring PGRS | 4.858887 | 6.943516 | 0.268636 |
|  | rs13160562 | 0.33364 | 0.146902 | 0.012665 |
|  | rs2140418 | 2.103363 | 0.667267 | 0.019089 |
|  | rs2154294 | 1.862519 | 0.571619 | 0.042719 |
|  | rs6902771 | 0.564571 | 0.176996 | 0.068222 |
|  | rs2827312 | 0.52759 | 0.193021 | 0.080501 |
|  | rs4758317 | 1.665761 | 0.504191 | 0.091818 |
|  | rs36563 | 1.778809 | 0.614447 | 0.095446 |
|  | rs6716455 | 0.299677 | 0.217459 | 0.096782 |
|  | rs1800759 | 1.549469 | 0.453801 | 0.134858 |
|  | rs59677118 | 1.850238 | 0.768944 | 0.138721 |
|  | rs1908556 | 1.685238 | 0.594204 | 0.138822 |
|  | rs9512637 | 0.615293 | 0.207454 | 0.149748 |
|  | rs9871864 | 1.522971 | 0.453904 | 0.158115 |
|  | rs2810114 | 1.536326 | 0.4733 | 0.163376 |
|  | rs195204 | 0.566931 | 0.232576 | 0.166546 |
|  | rs1789891 | 1.622525 | 0.573557 | 0.170958 |
|  | rs804292 | 1.516657 | 0.466061 | 0.175289 |
|  | rs12388359 | 1.465855 | 0.422094 | 0.184132 |
|  | rs9825310 | 0.665293 | 0.20475 | 0.185444 |
|  | rs420817 | 1.47068 | 0.442062 | 0.199404 |
|  | rs12472151 | 1.940125 | 1.02551 | 0.209901 |
|  | rs9636231 | 1.458068 | 0.448622 | 0.220329 |
|  | rs8040009 | 1.527508 | 0.534535 | 0.226047 |
|  | rs642899 | 1.458659 | 0.469048 | 0.240389 |
|  | rs16985179 | 1.64265 | 0.695754 | 0.241288 |
|  | rs7590720 | 1.418938 | 0.437401 | 0.256328 |
|  | rs10908907 | 1.429243 | 0.451869 | 0.25863 |
|  | rs10893366 | 0.600606 | 0.286641 | 0.285418 |
|  | rs12311304 | 1.383326 | 0.420782 | 0.286077 |
|  | rs67031482 | 0.728421 | 0.217158 | 0.287824 |
|  | rs62202398 | 0.336676 | 0.344994 | 0.28806 |
|  | rs933769 | 1.43503 | 0.490488 | 0.290635 |
|  | rs7553212 | 0.709143 | 0.237238 | 0.304245 |
|  | rs3738443 | 1.428353 | 0.513346 | 0.321197 |
|  | rs7144649 | 1.375566 | 0.452352 | 0.332224 |
|  | rs4770403 | 0.660599 | 0.289109 | 0.343457 |
|  | rs1864982 | 0.617047 | 0.322613 | 0.355774 |
|  | rs4543123 | 0.700653 | 0.272604 | 0.36054 |
|  | rs3930234 | 0.646206 | 0.314759 | 0.370026 |
|  | rs3131513 | 1.287032 | 0.378943 | 0.391426 |
|  | rs6943555 | 0.730067 | 0.272181 | 0.398726 |
|  | rs1109501 | 0.732852 | 0.272439 | 0.403114 |
|  | rs768048 | 1.39114 | 0.552757 | 0.406068 |
|  | rs750338 | 0.731089 | 0.285151 | 0.421943 |
|  | rs1344694 | 1.276331 | 0.392562 | 0.427615 |
|  | rs1573496 | 1.356848 | 0.600313 | 0.490356 |
|  | rs279861 | 0.815112 | 0.245978 | 0.498132 |
|  | rs2303317 | 0.822117 | 0.244302 | 0.509803 |
|  | rs3764435 | 1.214629 | 0.358785 | 0.510377 |
|  | rs1497571 | 0.822227 | 0.244757 | 0.510821 |
|  | rs9656709 | 0.820846 | 0.246479 | 0.510881 |
|  | rs10849915 | 0.822206 | 0.269482 | 0.550313 |
|  | rs567926 | 0.836474 | 0.253075 | 0.555068 |
|  | rs2380220 | 1.249185 | 0.48278 | 0.564823 |
|  | rs4293630 | 0.760355 | 0.363021 | 0.566079 |
|  | rs1793257 | 0.566367 | 0.572989 | 0.574155 |
|  | rs36061340 | 0.679702 | 0.497724 | 0.598008 |
|  | rs10253361 | 0.861988 | 0.258771 | 0.620804 |
|  | rs1230165 | 1.193484 | 0.432371 | 0.625382 |
|  | rs4761097 | 0.872924 | 0.261475 | 0.65003 |
|  | rs1876831 | 1.158222 | 0.38941 | 0.662196 |
|  | rs59972978 | 1.176077 | 0.441887 | 0.665994 |
|  | rs2188561 | 0.857874 | 0.315438 | 0.676741 |
|  | rs237238 | 1.246429 | 0.66258 | 0.678589 |
|  | rs1353899 | 1.154666 | 0.412985 | 0.687624 |
|  | rs4440177 | 1.122196 | 0.342584 | 0.705693 |
|  | rs2369955 | 0.836586 | 0.402156 | 0.710511 |
|  | rs3819197 | 1.131738 | 0.381739 | 0.713698 |
|  | rs1824024 | 1.109115 | 0.345478 | 0.739531 |
|  | rs8062326 | 0.722653 | 0.730122 | 0.747829 |
|  | rs1353621 | 1.094502 | 0.334009 | 0.767308 |
|  | rs13259667 | 0.844405 | 0.506406 | 0.777941 |
|  | rs6701037 | 1.07825 | 0.317707 | 0.798189 |
|  | rs11724320 | 0.924246 | 0.291845 | 0.802991 |
|  | rs2228093 | 0.898695 | 0.413373 | 0.816371 |
|  | rs1380131 | 0.897259 | 0.471166 | 0.836439 |
|  | rs1229984 | 0.815317 | 0.82952 | 0.840947 |
|  | rs1042026 | 1.063837 | 0.342065 | 0.847385 |
|  | rs3762894 | 1.066644 | 0.413336 | 0.86777 |
|  | rs11851015 | 0.934625 | 0.413282 | 0.878479 |
|  | rs4478858 | 1.030155 | 0.309007 | 0.921105 |
|  | rs9556711 | 1.058971 | 0.639788 | 0.924443 |
|  | rs886205 | 0.96404 | 0.376164 | 0.925222 |
|  | rs2548145 | 0.977243 | 0.287719 | 0.937678 |
|  | rs2100290 | 1.021107 | 0.300345 | 0.943388 |
|  | rs284786 | 1.018964 | 0.328026 | 0.953463 |
|  | rs242938 | 1.014687 | 0.604636 | 0.980479 |
|  | rs1318937 | 0.990529 | 0.43071 | 0.982539 |
|  | rs1000579 | 0.996263 | 0.301518 | 0.99013 |
| Mother’s ethnicity | Offspring PGRS | 0.001405 | 0.004422 | 0.036895 |
|  | rs59677118 | 6.866373 | 4.484738 | 0.00318 |
|  | rs11724320 | 7.625883 | 6.0414 | 0.010337 |
|  | rs1864982 | 4.298761 | 2.770439 | 0.023647 |
|  | rs279861 | 4.993441 | 3.956883 | 0.042418 |
|  | rs7144649 | 3.455676 | 2.193323 | 0.050736 |
|  | rs67031482 | 4.156157 | 3.280759 | 0.07112 |
|  | rs2100290 | 0.254991 | 0.201323 | 0.083485 |
|  | rs9556711 | 3.917696 | 3.191286 | 0.093675 |
|  | rs9825310 | 0.278967 | 0.221483 | 0.107833 |
|  | rs567926 | 3.002639 | 2.073231 | 0.111299 |
|  | rs2369955 | 3.090733 | 2.194442 | 0.111994 |
|  | rs1353899 | 2.719098 | 1.741109 | 0.118247 |
|  | rs2154294 | 2.536674 | 1.754163 | 0.178272 |
|  | rs16985179 | 2.375837 | 1.8908 | 0.276889 |
|  | rs4478858 | 2.018249 | 1.307363 | 0.278333 |
|  | rs10893366 | 2.114946 | 1.465161 | 0.279601 |
|  | rs10849915 | 1.941296 | 1.246726 | 0.301641 |
|  | rs6943555 | 0.337303 | 0.356008 | 0.303164 |
|  | rs1793257 | 2.859296 | 3.004174 | 0.317354 |
|  | rs886205 | 1.990931 | 1.374349 | 0.318506 |
|  | rs6902771 | 0.508398 | 0.349221 | 0.324704 |
|  | rs6701037 | 0.508452 | 0.350037 | 0.325857 |
|  | rs1876831 | 0.36071 | 0.37981 | 0.332844 |
|  | rs4761097 | 1.858222 | 1.196956 | 0.336083 |
|  | rs10253361 | 0.52774 | 0.364383 | 0.354607 |
|  | rs4758317 | 1.765159 | 1.148719 | 0.382567 |
|  | rs7553212 | 0.503553 | 0.397029 | 0.384224 |
|  | rs1353621 | 1.690608 | 1.075515 | 0.40915 |
|  | rs1042026 | 1.628195 | 1.050361 | 0.449863 |
|  | rs1230165 | 0.468951 | 0.495639 | 0.473694 |
|  | rs2140418 | 0.472875 | 0.499271 | 0.47812 |
|  | rs4770403 | 0.48283 | 0.507118 | 0.488172 |
|  | rs1318937 | 1.643047 | 1.288347 | 0.526564 |
|  | rs284786 | 1.484807 | 0.970655 | 0.545401 |
|  | rs3764435 | 0.680164 | 0.439889 | 0.551212 |
|  | rs2188561 | 1.492799 | 1.015913 | 0.556045 |
|  | rs3738443 | 0.543503 | 0.573391 | 0.563306 |
|  | rs1800759 | 0.677003 | 0.463401 | 0.568756 |
|  | rs750338 | 1.479426 | 1.017758 | 0.569144 |
|  | rs1789891 | 0.5558 | 0.588165 | 0.578876 |
|  | rs62202398 | 1.792659 | 1.910382 | 0.583877 |
|  | rs2303317 | 0.712837 | 0.45985 | 0.599771 |
|  | rs59972978 | 1.476758 | 1.121541 | 0.607725 |
|  | rs1824024 | 1.386021 | 0.898191 | 0.614449 |
|  | rs12388359 | 1.376187 | 0.878879 | 0.617075 |
|  | rs4543123 | 1.406477 | 0.972516 | 0.621808 |
|  | rs3819197 | 1.385181 | 0.954748 | 0.636408 |
|  | rs36563 | 0.63479 | 0.668455 | 0.666051 |
|  | rs804292 | 0.721703 | 0.564397 | 0.676648 |
|  | rs1344694 | 1.30054 | 0.853884 | 0.688983 |
|  | rs10908907 | 0.731481 | 0.579324 | 0.692984 |
|  | rs3930234 | 1.360539 | 1.106346 | 0.704971 |
|  | rs1109501 | 0.744545 | 0.589724 | 0.709577 |
|  | rs2827312 | 1.276625 | 0.873378 | 0.721108 |
|  | rs11851015 | 0.694435 | 0.737813 | 0.731435 |
|  | rs195204 | 0.776713 | 0.610486 | 0.747841 |
|  | rs768048 | 0.72234 | 0.771475 | 0.760714 |
|  | rs3762894 | 1.252212 | 0.9797 | 0.773751 |
|  | rs6716455 | 0.738782 | 0.782206 | 0.77492 |
|  | rs2228093 | 0.759132 | 0.797581 | 0.793094 |
|  | rs9871864 | 1.174767 | 0.745891 | 0.799741 |
|  | rs9512637 | 1.174461 | 0.764822 | 0.804955 |
|  | rs8040009 | 0.804253 | 0.710081 | 0.805115 |
|  | rs642899 | 0.833427 | 0.65735 | 0.817302 |
|  | rs2810114 | 1.115613 | 0.774167 | 0.874728 |
|  | rs933769 | 1.130868 | 0.885336 | 0.875171 |
|  | rs4440177 | 0.914755 | 0.622603 | 0.895848 |
|  | rs1497571 | 1.07407 | 0.677604 | 0.909821 |
|  | rs9656709 | 1.070116 | 0.683417 | 0.915493 |
|  | rs2548145 | 1.068843 | 0.67272 | 0.915757 |
|  | rs12311304 | 0.929884 | 0.643647 | 0.916357 |
|  | rs36061340 | 9.18E-06 | 0.001224 | 0.930684 |
|  | rs9636231 | 1.05701 | 0.731913 | 0.936181 |
|  | rs1380131 | 1.069888 | 1.131547 | 0.949071 |
|  | rs13160562 | 0.95754 | 0.664416 | 0.950141 |
|  | rs7590720 | 1.042147 | 0.724336 | 0.952637 |
|  | rs1000579 | 1.031731 | 0.665499 | 0.961375 |
|  | rs420817 | 1.030758 | 0.653294 | 0.961877 |
|  | rs3131513 | 1.020382 | 0.653656 | 0.974873 |
|  | rs1573496 | 0.996773 | 1.056741 | 0.997567 |
| Offspring’s ethnicity | Offspring PGRS | 0.100905 | 0.186846 | 0.215483 |
|  | rs1229984 | 7.388332 | 4.218931 | 0.000461 |
|  | rs567926 | 2.437105 | 1.00581 | 0.030892 |
|  | rs279861 | 2.363252 | 0.974616 | 0.037031 |
|  | rs9825310 | 0.413083 | 0.183793 | 0.046915 |
|  | rs1864982 | 2.393613 | 1.058556 | 0.048428 |
|  | rs7144649 | 2.16131 | 0.873741 | 0.05659 |
|  | rs59677118 | 2.430182 | 1.217719 | 0.076378 |
|  | rs1000579 | 0.464602 | 0.216333 | 0.099699 |
|  | rs12388359 | 1.762185 | 0.623838 | 0.109515 |
|  | rs6902771 | 0.527332 | 0.223364 | 0.130845 |
|  | rs6716455 | 2.022417 | 0.951584 | 0.134433 |
|  | rs7553212 | 0.480867 | 0.238772 | 0.140342 |
|  | rs1353899 | 1.825936 | 0.772275 | 0.154573 |
|  | rs10253361 | 0.548579 | 0.233115 | 0.157671 |
|  | rs2140418 | 0.355683 | 0.262189 | 0.160818 |
|  | rs2810114 | 0.470468 | 0.25681 | 0.16717 |
|  | rs9656709 | 1.7338 | 0.705408 | 0.176182 |
|  | rs10893366 | 1.821729 | 0.812207 | 0.178534 |
|  | rs3738443 | 1.813891 | 0.80463 | 0.17947 |
|  | rs67031482 | 1.67227 | 0.67321 | 0.201518 |
|  | rs886205 | 1.710036 | 0.757542 | 0.225858 |
|  | rs4758317 | 0.612382 | 0.257877 | 0.244199 |
|  | rs933769 | 1.663623 | 0.728604 | 0.245156 |
|  | rs6701037 | 0.623795 | 0.256947 | 0.251911 |
|  | rs2100290 | 0.635454 | 0.255675 | 0.259777 |
|  | rs3764435 | 0.636652 | 0.257595 | 0.264433 |
|  | rs9636231 | 0.58743 | 0.293164 | 0.286426 |
|  | rs2188561 | 1.549341 | 0.649878 | 0.296574 |
|  | rs1793257 | 2.140733 | 1.573735 | 0.300491 |
|  | rs1380131 | 1.759304 | 0.961303 | 0.301196 |
|  | rs8040009 | 1.595222 | 0.732648 | 0.309227 |
|  | rs36563 | 0.47413 | 0.349008 | 0.310669 |
|  | rs750338 | 1.533963 | 0.65154 | 0.313778 |
|  | rs2369955 | 1.666247 | 0.84728 | 0.315338 |
|  | rs2380220 | 0.498099 | 0.365155 | 0.341756 |
|  | rs1344694 | 1.460657 | 0.589851 | 0.34812 |
|  | rs4770403 | 0.564807 | 0.345741 | 0.350698 |
|  | rs1318937 | 1.570567 | 0.777686 | 0.36193 |
|  | rs768048 | 1.587772 | 0.809498 | 0.364497 |
|  | rs1573496 | 1.638034 | 0.896182 | 0.367052 |
|  | rs4293630 | 0.525034 | 0.387543 | 0.382733 |
|  | rs13160562 | 0.667171 | 0.312237 | 0.387171 |
|  | rs10849915 | 1.412685 | 0.570844 | 0.392551 |
|  | rs11724320 | 1.398239 | 0.558775 | 0.401574 |
|  | rs284786 | 1.387172 | 0.567074 | 0.423388 |
|  | rs4478858 | 1.339966 | 0.527555 | 0.457298 |
|  | rs2228093 | 0.593864 | 0.422131 | 0.463495 |
|  | rs1789891 | 0.653274 | 0.403448 | 0.490571 |
|  | rs59972978 | 0.664057 | 0.395856 | 0.492237 |
|  | rs1109501 | 1.322417 | 0.564158 | 0.512422 |
|  | rs6943555 | 0.72444 | 0.361462 | 0.518238 |
|  | rs1042026 | 1.294751 | 0.533226 | 0.530506 |
|  | rs10908907 | 1.305415 | 0.556263 | 0.531668 |
|  | rs7590720 | 1.291959 | 0.537427 | 0.538027 |
|  | rs2827312 | 1.297754 | 0.551079 | 0.539362 |
|  | rs2303317 | 0.784634 | 0.311809 | 0.541649 |
|  | rs2154294 | 1.267754 | 0.500282 | 0.547705 |
|  | rs1497571 | 1.251802 | 0.491236 | 0.567118 |
|  | rs3819197 | 0.771181 | 0.383473 | 0.601298 |
|  | rs1230165 | 1.273034 | 0.596945 | 0.606685 |
|  | rs1876831 | 0.776454 | 0.384813 | 0.609683 |
|  | rs642899 | 1.229295 | 0.54185 | 0.639533 |
|  | rs62202398 | 0.630565 | 0.649158 | 0.654203 |
|  | rs4543123 | 1.209797 | 0.536735 | 0.66772 |
|  | rs9871864 | 1.178153 | 0.464518 | 0.677543 |
|  | rs3930234 | 0.786061 | 0.47359 | 0.68949 |
|  | rs3762894 | 1.197592 | 0.590581 | 0.714632 |
|  | rs1908556 | 0.81067 | 0.494692 | 0.730875 |
|  | rs16985179 | 1.236306 | 0.770174 | 0.733469 |
|  | rs12311304 | 1.149794 | 0.476175 | 0.736084 |
|  | rs36061340 | 1.266998 | 0.95022 | 0.752349 |
|  | rs9556711 | 1.242908 | 0.926481 | 0.7705 |
|  | rs3131513 | 1.119351 | 0.440854 | 0.774668 |
|  | rs11851015 | 1.143176 | 0.629186 | 0.807912 |
|  | rs9512637 | 1.10207 | 0.447853 | 0.810978 |
|  | rs4761097 | 0.910952 | 0.361094 | 0.813987 |
|  | rs1824024 | 1.102151 | 0.456823 | 0.814471 |
|  | rs2548145 | 0.918153 | 0.359787 | 0.827497 |
|  | rs195204 | 0.929145 | 0.430513 | 0.873977 |
|  | rs237238 | 1.102249 | 0.823923 | 0.896377 |
|  | rs1353621 | 1.053112 | 0.427031 | 0.898449 |
|  | rs4440177 | 0.948687 | 0.398151 | 0.900117 |
|  | rs804292 | 1.053255 | 0.459848 | 0.905402 |
|  | rs420817 | 1.025635 | 0.403686 | 0.948724 |
|  | rs1800759 | 0.982316 | 0.392574 | 0.96439 |
|  | rs13259667 | 1.003701 | 0.741057 | 0.996007 |
