# Supplementary material for: Exploration of a Polygenic Risk Score for Alcohol Consumption: A Longitudinal Analysis from the ALSPAC Cohort
Source: PLoS One. 2016 Nov 30;11(11):e0167360. doi: 10.1371/journal.pone.0167360 (PMC5130278; doi:10.1371/journal.pone.0167360)
Supplement: S7 Table — (DOCX) [file pone.0167360.s007.docx]

**S7 Table: Sensitivity analysis for repeated measures analysis in ALSPAC mothers**

| **Excluding pregnant women** | | | | | **Excluding time points 4, 7, 8 and 12** | | | | |
| --- | --- | --- | --- | --- | --- | --- | --- | --- | --- |
| **SNP** | **Effect size** | **SE** | ***t*** | **p-value** | **SNP** | **Effect size** | **SE** | ***t*** | **p-value** |
| **rs1229984** | -0.15919 | 0.041744 | -3.8136 | 0.000137 | **rs1229984** | -0.14867 | 0.039253 | -3.78735 | 0.000152 |
| **rs11724320** | -0.02863 | 0.011459 | -2.49862 | 0.012468 | **rs1318937** | 0.036543 | 0.014992 | 2.437443 | 0.014792 |
| **rs1318937** | 0.037773 | 0.015905 | 2.37499 | 0.017549 | **rs4770403** | -0.03048 | 0.012996 | -2.34546 | 0.019004 |
| **rs1876831** | -0.02889 | 0.012744 | -2.26672 | 0.023407 | **rs59972978** | 0.026553 | 0.013457 | 1.973194 | 0.048473 |
| **rs2100290** | -0.02273 | 0.010752 | -2.11371 | 0.03454 | **rs11724320** | -0.02108 | 0.010797 | -1.95278 | 0.050846 |
| **rs1380131** | 0.037511 | 0.018742 | 2.001422 | 0.045347 | **rs1876831** | -0.02329 | 0.012003 | -1.94077 | 0.052287 |
| **rs6902771** | 0.019699 | 0.010711 | 1.839078 | 0.065904 | **rs6902771** | 0.019412 | 0.010094 | 1.923104 | 0.054467 |
| **rs3131513** | 0.020248 | 0.011022 | 1.837034 | 0.066205 | **rs1380131** | 0.032684 | 0.017668 | 1.849914 | 0.064326 |
| **rs4770403** | -0.02502 | 0.013802 | -1.81256 | 0.069899 | **rs2100290** | -0.01846 | 0.010127 | -1.82301 | 0.068302 |
| **rs4478858** | -0.01954 | 0.010802 | -1.80925 | 0.070412 | **rs642899** | 0.021584 | 0.012022 | 1.795415 | 0.072588 |
| **rs1497571** | 0.018851 | 0.01079 | 1.747124 | 0.080616 | **rs3131513** | 0.018296 | 0.010382 | 1.762181 | 0.078039 |
| **rs642899** | 0.020502 | 0.012757 | 1.607184 | 0.108014 | **rs4478858** | -0.01685 | 0.010177 | -1.65561 | 0.097802 |
| **rs750338** | 0.02021 | 0.012947 | 1.560962 | 0.118533 | **rs1230165** | 0.020956 | 0.013005 | 1.611428 | 0.107087 |
| **rs59972978** | 0.02206 | 0.014282 | 1.544651 | 0.122431 | **rs3764435** | 0.016495 | 0.010299 | 1.601597 | 0.109245 |
| **rs1353899** | -0.02079 | 0.013681 | -1.51964 | 0.128602 | **rs750338** | 0.018585 | 0.012192 | 1.524345 | 0.127422 |
| **rs933769** | -0.02053 | 0.013936 | -1.4733 | 0.140672 | **rs1497571** | 0.014948 | 0.010171 | 1.469781 | 0.141621 |
| **rs11851015** | -0.01948 | 0.015865 | -1.22778 | 0.21953 | **rs6943555** | -0.01562 | 0.011689 | -1.33644 | 0.181405 |
| **rs9656709** | 0.013344 | 0.010881 | 1.226415 | 0.220043 | **rs13160562** | -0.0144 | 0.010803 | -1.33333 | 0.182425 |
| **rs2810114** | -0.01464 | 0.012081 | -1.21153 | 0.225693 | **rs9656709** | 0.012888 | 0.010246 | 1.257813 | 0.208459 |
| **rs1230165** | 0.016095 | 0.013808 | 1.165671 | 0.243748 | **rs12311304** | -0.01355 | 0.010918 | -1.24145 | 0.214438 |
| **rs4293630** | -0.01777 | 0.015896 | -1.11759 | 0.263744 | **rs2810114** | -0.01413 | 0.01138 | -1.2414 | 0.214458 |
| **rs12311304** | -0.01276 | 0.011584 | -1.10194 | 0.270488 | **rs1789891** | 0.016954 | 0.013707 | 1.236892 | 0.216127 |
| **rs1864982** | -0.01715 | 0.016146 | -1.06212 | 0.288182 | **rs62202398** | -0.0257 | 0.020942 | -1.22709 | 0.21979 |
| **rs3764435** | 0.0115 | 0.010926 | 1.052495 | 0.292573 | **rs36563** | -0.01735 | 0.014156 | -1.22559 | 0.220355 |
| **rs242938** | -0.02265 | 0.022389 | -1.01148 | 0.311786 | **rs1042026** | -0.01359 | 0.01114 | -1.21992 | 0.222496 |
| **rs62202398** | -0.02173 | 0.022224 | -0.97774 | 0.328204 | **rs1353899** | -0.01556 | 0.012885 | -1.20767 | 0.227174 |
| **rs36563** | -0.01456 | 0.015035 | -0.96833 | 0.332882 | **rs933769** | -0.01567 | 0.01313 | -1.1935 | 0.232675 |
| **rs1042026** | -0.01137 | 0.011826 | -0.96162 | 0.336241 | **rs9556711** | 0.023593 | 0.021447 | 1.100076 | 0.271299 |
| **rs2140418** | -0.01294 | 0.013592 | -0.95197 | 0.341112 | **rs2303317** | 0.011127 | 0.010211 | 1.089701 | 0.275845 |
| **rs13160562** | -0.01076 | 0.011467 | -0.93861 | 0.34793 | **rs1908556** | -0.01456 | 0.014633 | -0.99513 | 0.319673 |
| **rs1800759** | 0.009983 | 0.010985 | 0.908812 | 0.363449 | **rs4440177** | 0.010481 | 0.01073 | 0.976806 | 0.328665 |
| **rs1789891** | 0.012909 | 0.014543 | 0.887638 | 0.374736 | **rs11851015** | -0.01442 | 0.014957 | -0.96401 | 0.33504 |
| **rs10893366** | 0.012471 | 0.014538 | 0.857808 | 0.390998 | **rs12388359** | 0.014444 | 0.015024 | 0.961401 | 0.336351 |
| **rs6943555** | -0.0106 | 0.012406 | -0.85476 | 0.392684 | **rs4293630** | -0.01432 | 0.014974 | -0.95633 | 0.338905 |
| **rs9556711** | 0.018373 | 0.022773 | 0.806776 | 0.419796 | **rs3819197** | -0.01072 | 0.011829 | -0.90658 | 0.364629 |
| **rs1353621** | 0.008642 | 0.011121 | 0.777025 | 0.437144 | **rs2140418** | -0.01156 | 0.012807 | -0.90246 | 0.366811 |
| **rs8040009** | -0.00997 | 0.014056 | -0.70919 | 0.478205 | **rs242938** | -0.01903 | 0.021093 | -0.902 | 0.367058 |
| **rs886205** | -0.00991 | 0.014127 | -0.70152 | 0.482978 | **rs1353621** | 0.008174 | 0.010477 | 0.780138 | 0.43531 |
| **rs2303317** | 0.007217 | 0.010833 | 0.666176 | 0.505299 | **rs279861** | -0.00784 | 0.010254 | -0.76426 | 0.44471 |
| **rs4543123** | -0.00825 | 0.012811 | -0.64418 | 0.519458 | **rs6701037** | 0.007362 | 0.010195 | 0.72209 | 0.47024 |
| **rs10908907** | -0.00795 | 0.012406 | -0.64091 | 0.521579 | **rs1800759** | 0.00737 | 0.010341 | 0.712718 | 0.476021 |
| **rs67031482** | 0.006025 | 0.010811 | 0.557333 | 0.5773 | **rs3930234** | 0.009787 | 0.014173 | 0.690535 | 0.489858 |
| **rs9512637** | -0.00616 | 0.011126 | -0.5539 | 0.579648 | **rs4543123** | -0.0083 | 0.012072 | -0.68744 | 0.491808 |
| **rs4440177** | 0.006007 | 0.011392 | 0.527305 | 0.597982 | **rs420817** | 0.006436 | 0.010154 | 0.633792 | 0.526216 |
| **rs1908556** | -0.0081 | 0.015533 | -0.52123 | 0.602205 | **rs1864982** | -0.00956 | 0.015215 | -0.62859 | 0.529616 |
| **rs9636231** | -0.00543 | 0.011989 | -0.45258 | 0.650849 | **rs9512637** | -0.00631 | 0.010476 | -0.60208 | 0.547121 |
| **rs2827312** | -0.00524 | 0.011787 | -0.4445 | 0.656681 | **rs8062326** | -0.01722 | 0.028907 | -0.59562 | 0.55143 |
| **rs768048** | -0.00695 | 0.015672 | -0.44352 | 0.657389 | **rs1000579** | 0.005888 | 0.010279 | 0.572822 | 0.566765 |
| **rs1573496** | -0.00746 | 0.01802 | -0.41416 | 0.678757 | **rs10893366** | 0.007788 | 0.013691 | 0.568832 | 0.56947 |
| **rs2154294** | 0.004408 | 0.010761 | 0.40964 | 0.68207 | **rs10908907** | -0.00659 | 0.011688 | -0.56365 | 0.572991 |
| **rs7553212** | 0.004656 | 0.011414 | 0.407938 | 0.683319 | **rs10849915** | 0.005809 | 0.010741 | 0.540818 | 0.588633 |
| **rs10253361** | 0.004291 | 0.010791 | 0.397583 | 0.690937 | **rs13259667** | -0.0102 | 0.01908 | -0.53436 | 0.59309 |
| **rs420817** | 0.004084 | 0.010772 | 0.379158 | 0.70457 | **rs4758317** | 0.005524 | 0.010361 | 0.533145 | 0.593933 |
| **rs12472151** | 0.009739 | 0.025787 | 0.377693 | 0.705659 | **rs567926** | -0.00533 | 0.010254 | -0.51968 | 0.603286 |
| **rs9871864** | 0.0041 | 0.010858 | 0.377645 | 0.705694 | **rs1344694** | -0.00558 | 0.010818 | -0.5155 | 0.606206 |
| **rs4758317** | -0.00412 | 0.01099 | -0.37472 | 0.707866 | **rs2154294** | 0.005099 | 0.010135 | 0.503104 | 0.614891 |
| **rs6701037** | 0.003992 | 0.010817 | 0.369046 | 0.712093 | **rs2380220** | 0.006997 | 0.014298 | 0.489365 | 0.624583 |
| **rs8062326** | -0.0112 | 0.030739 | -0.36445 | 0.71552 | **rs3762894** | -0.00652 | 0.013663 | -0.47737 | 0.633101 |
| **rs1824024** | 0.004161 | 0.011663 | 0.356717 | 0.721303 | **rs2827312** | -0.00511 | 0.011103 | -0.46026 | 0.645329 |
| **rs13259667** | -0.0069 | 0.020259 | -0.34082 | 0.733237 | **rs7590720** | -0.00503 | 0.011172 | -0.45012 | 0.652626 |
| **rs195204** | 0.004148 | 0.012433 | 0.333625 | 0.738663 | **rs3738443** | -0.00593 | 0.013528 | -0.43814 | 0.661288 |
| **rs6716455** | 0.005147 | 0.015538 | 0.331265 | 0.740444 | **rs36061340** | 0.008708 | 0.021354 | 0.407794 | 0.683425 |
| **rs279861** | -0.00353 | 0.010882 | -0.32447 | 0.74558 | **rs7553212** | 0.004202 | 0.010757 | 0.390616 | 0.696081 |
| **rs2380220** | 0.004811 | 0.015175 | 0.317006 | 0.751239 | **rs804292** | 0.004583 | 0.01184 | 0.387078 | 0.698699 |
| **rs10849915** | 0.003524 | 0.011404 | 0.309017 | 0.757308 | **rs9825310** | 0.003791 | 0.010196 | 0.371779 | 0.710058 |
| **rs2548145** | -0.00331 | 0.010783 | -0.30703 | 0.75882 | **rs2369955** | -0.00565 | 0.015244 | -0.37049 | 0.711017 |
| **rs3930234** | 0.004575 | 0.015038 | 0.304242 | 0.760943 | **rs6716455** | 0.004397 | 0.014636 | 0.30044 | 0.763842 |
| **rs804292** | 0.003709 | 0.012565 | 0.295161 | 0.767871 | **rs195204** | 0.003398 | 0.011714 | 0.290057 | 0.771773 |
| **rs12388359** | 0.004708 | 0.015971 | 0.294777 | 0.768164 | **rs10253361** | 0.002528 | 0.010163 | 0.24875 | 0.803555 |
| **rs237238** | -0.00605 | 0.021457 | -0.28176 | 0.778131 | **rs2188561** | -0.00297 | 0.012065 | -0.24616 | 0.805561 |
| **rs1109501** | 0.003314 | 0.012393 | 0.267393 | 0.789167 | **rs2548145** | -0.00232 | 0.010159 | -0.22884 | 0.818991 |
| **rs3762894** | -0.00373 | 0.014521 | -0.25717 | 0.797047 | **rs9636231** | -0.00255 | 0.011301 | -0.22592 | 0.821263 |
| **rs3819197** | -0.0031 | 0.012559 | -0.24652 | 0.805278 | **rs16985179** | -0.00374 | 0.017774 | -0.21015 | 0.833549 |
| **rs567926** | -0.00249 | 0.010883 | -0.2285 | 0.819259 | **rs1793257** | 0.00511 | 0.027356 | 0.186801 | 0.851817 |
| **rs3738443** | -0.00325 | 0.01437 | -0.22637 | 0.820917 | **rs7144649** | 0.002173 | 0.012191 | 0.178216 | 0.858554 |
| **rs1793257** | -0.00656 | 0.029016 | -0.22601 | 0.821196 | **rs67031482** | 0.001701 | 0.010191 | 0.166899 | 0.867449 |
| **rs7590720** | 0.00243 | 0.011857 | 0.204906 | 0.837645 | **rs1109501** | -0.00182 | 0.011671 | -0.15635 | 0.875759 |
| **rs59677118** | 0.003221 | 0.018776 | 0.17153 | 0.863807 | **rs284786** | 0.001164 | 0.011068 | 0.105154 | 0.916254 |
| **rs1000579** | 0.001678 | 0.010908 | 0.153869 | 0.877713 | **rs237238** | -0.00211 | 0.020201 | -0.10443 | 0.916825 |
| **rs9825310** | 0.001424 | 0.010817 | 0.13166 | 0.895254 | **rs1824024** | 0.001013 | 0.010988 | 0.092223 | 0.926521 |
| **rs4761097** | -0.00134 | 0.010812 | -0.12427 | 0.901098 | **rs59677118** | 0.001392 | 0.017689 | 0.078677 | 0.93729 |
| **rs2369955** | 0.001726 | 0.016188 | 0.106644 | 0.915071 | **rs4761097** | -0.00075 | 0.010186 | -0.07407 | 0.940957 |
| **rs16985179** | 0.002008 | 0.018869 | 0.106394 | 0.91527 | **rs9871864** | 0.000702 | 0.010229 | 0.068639 | 0.945277 |
| **rs2228093** | -0.00145 | 0.015943 | -0.09095 | 0.927532 | **rs8040009** | 0.000556 | 0.013239 | 0.041971 | 0.966522 |
| **rs1344694** | 0.001033 | 0.011481 | 0.089947 | 0.92833 | **rs12472151** | 0.000989 | 0.024218 | 0.040818 | 0.967441 |
| **rs36061340** | 0.001922 | 0.022661 | 0.084832 | 0.932395 | **rs768048** | 0.000514 | 0.014772 | 0.034782 | 0.972253 |
| **rs7144649** | 0.000912 | 0.012926 | 0.070546 | 0.943759 | **rs1573496** | -0.00049 | 0.016979 | -0.02874 | 0.977069 |
| **rs284786** | -0.00042 | 0.011741 | -0.03615 | 0.971162 | **rs2228093** | 0.000181 | 0.015023 | 0.012041 | 0.990393 |
| **rs2188561** | 0.000416 | 0.012797 | 0.032476 | 0.974092 | **rs886205** | 0.000104 | 0.013302 | 0.007795 | 0.993781 |
